# Catalogue of socioeconomic disparities and characteristics of 199+ chronic conditions—A nationwide register-based population study

**DOI:** 10.1371/journal.pone.0278380

**Published:** 2022-12-30

**Authors:** Michael Falk Hvidberg, Anne Frølich, Sanne Lykke Lundstrøm

**Affiliations:** 1 Innovation and Research Centre for Multimorbidity, Slagelse Hospital, Slagelse, Denmark; 2 University of York, York, United Kingdom; 3 The Research Unit for General Practice and Section of General Practice, Department of Public Health, University of Copenhagen, Copenhagen, Denmark; 4 Center for Clinical Research and Prevention, Bispebjerg and Frederiksberg Hospital, Copenhagen, The Capital Region of Denmark; Kasturba Medical College Mangalore, Manipal Academy of Higher Education, INDIA

## Abstract

**Background:**

Real-world information on socioeconomic differences within and between chronic conditions represents an important data source for treatments and decision-makers executing and prioritising healthcare resources.

**Aims:**

The aim of this study was to estimate the prevalence and mean of socioeconomic disparities from educational, income, and socioeconomic positions of 199 chronic conditions and disease groups, including sex and age group estimates, for use in planning of care services and prioritisation, by healthcare professionals, decision-makers and researchers.

**Methods:**

The study population includes all Danish residents 16 years and above, alive on 1 January 2013 (n = 4,555,439). The data was established by linking seven national registers encompassing educational achievements, incomes, socioeconomic positions, hospital- and general practice services, and filled-in out-of-hospital prescriptions. The health register data were used to identify the 199+ chronic conditions. Socioeconomic differences were primarily measured as differences in educational prevalence levels from low to high educational achievements using a ratio. Furthermore, multiple binary logistic regression models were carried out to control for potential confounding and residual correlations of the crude estimates.

**Results:**

The prevalence of having one or more chronic conditions for patients with no educational achievement was 768 per thousand compared to 601.3 for patients with higher educational achievement (ratio 1.3). Across disease groups, the highest educational differences were found within disease group F–mental and behavioural (ratio 2.5), E–endocrine, nutritional and metabolic disease (ratio 2.4), I–diseases of the circulatory system (ratio 2.1) and, K–diseases of the digestive system (ratio 2.1). The highest educational differences among the 29 common diseases were found among schizophrenia (ratio 5.9), hyperkinetic disorders (ratio 5.2), dementia (ratio 4.9), osteoporosis (ratio 3.9), type 2 diabetes (ratio 3.8), chronic obstructive pulmonary disease COPD (ratio 3.3), heart conditions and stroke (ratios ranging from 2.3–3.1).

**Conclusions:**

A nationwide catalogue of socioeconomic disparities for 199+ chronic conditions and disease groups is catalogued and provided. The catalogue findings underline a large scope of socioeconomic disparities that exist across most chronic conditions. The data offer essential information on the socioeconomic disparities to inform future socially differentiated treatments, healthcare planning, etiological, economic, and other research areas.

## Introduction

The socioeconomic differences in morbidity and mortality, in particular across educational levels, have been widening in high-income countries for decades [1–8]. A wealth of research has linked socioeconomic inequality to reduced population health [2, 9–11]. Wilkinson’s (1996) influential work first demonstrated that it is not necessarily the wealthiest countries with the most healthy populations, but countries with the smallest income differences within society [9, 10]. More unequal societies show shorter life expectancy and higher prevalence rates of obesity, HIV infections, and mental illness, among others [9, 12–23]. Further, social disparities in multimorbidity are inversely related to educational achievement [24, 25]. Recently, high-income countries have seen increased income inequality [2]. In Europe, one out of four adults is now at risk of poverty or social exclusion [2, 26, 27]. Moreover, the fallout from the COVID-19 pandemic has particularly affected the socioeconomic low-income groups with chronic conditions such as heart diseases, diabetes, and chronic obstructive pulmonary disease (COPD) [28].

Along with increased income inequality [2], the disease burden of chronic conditions and multimorbidity is increasing [29, 30]. Consequently, the expenditures also increase worldwide along with ageing populations [31–40]. These expenditures have earlier been shown to account for up to 80 per cent of healthcare costs [41–44]; and to be positively correlated with multimorbidity and socioeconomic factors [45]. To control the growing cost of healthcare, decision-makers require access to reliable, real-world evidence of disease and treatment patterns [46, 47]. Real-world evidence of disease prevalence is essential for estimating the burden of disease, cost of illness, and budget impact on new health technologies [48, 49]. Thus, the World Health Organization (WHO) has long recommended improvement in data surveillance of chronic diseases [37]; and other studies have criticised and recommended additional methodological improvements measuring disease burden [50–53]. Hvidberg et al. (2016, 2019) earlier replied to these calls [54–58]. While most prevalence studies usually included a single or a limited number of chronic conditions [44, 59–66], Hvidberg et al. (2019) encompassed the full-sized disease burden of chronic conditions based on uniform methodology physician-reported conditions covering the whole adult population of Denmark [56, 57]. Besides the completeness, the advantages of this single-study approach were reliable, and comparable prevalence rates were estimated across conditions which often cannot be achieved in other studies caused by biases from comparing different, heterogenic data [54]. The study comprised 199 chronic conditions and found that a record high of two-thirds of the population had at least one chronic condition [56]. However, while sex and age characteristics were identified, the studies did not investigate the socioeconomic inequalities across the conditions. Comparable knowledge about socioeconomic differences within and between chronic conditions would, for example, support decision-makers or healthcare professionals’ evidence according to service needs and treatment potentials for future prioritisation and socially differential clinical treatments, as socioeconomic larger disparities could indicate treatment potentials or patient needs [24, 67–72]. This has long been used to inform and target populations within the treatment of health behaviours like smoking, exercise, obesity etc. [67], but less so within chronic diseases, although numerous studies have identified disparities within single diseases [25, 56]. Thus, comparable estimates of socioeconomic disparities across a long range of chronic conditions could provide new insights and practical usefulness and move future prioritisation and socially differentiated treatment forward.

The present study estimates the national prevalence rates of socioeconomic inequalities regarding educational levels, income, and socioeconomic position of 199 chronic conditions of the whole adult Danish population. The study provides an off-the-shelf catalogue and a comparative overview of socioeconomic inequalities across chronic conditions for healthcare professionals, decision-makers, researchers, and clinicians to use, for example, in practical treatment, targeting low-income patients of interest. We also want to provide concrete information for future prioritisation for specific chronic conditions based on the identified socioeconomic differences and size of prevalence. To the best of the authors’ knowledge, the present study is the first and most comprehensive, independent register-based attempt to estimate socioeconomic inequalities associated with the disease burden of a whole nationwide population using a comparable, uniform methodology.

## Methods

### Population

The nationwide study population comprised 4,555,439 Danish residents aged 16 years or older, alive on 1 January 2013. Hereof, 45.2% were 16–44 years old, 45.9% were 45–74 years old, and 8.9% were 75 years or older. The population had a mean age of 46.7, and 49.2% were men.

### The registers

Seven registers from Statistics Denmark were employed in the current study (Table 1). The registers have been recorded for public administration, for example, for claims and management, surveillance, tax, and control functions by government officials at the individual level [73].

**Table 1:** Summary of the registers used and characteristics of the selected population.

Registry	Years	Population	Contains
The Population’s Education Register (PER) [74]	2012	Entire population aged 16+ resident in Denmark	Education levels completed, educational level time completed, type and name, enrolment, time of enrolment, and length. The register only provides information about the education of more than 80 hours’ duration authorized by the Danish Ministry of Education.
			An eight-digit code enables transformation into the International Standard Classification of Education (ISCED).
The Income Statistics Register (ISR) [75]	2012	Entire population aged 16+ resident in Denmark	The register contains more than 160 variables, including salaries, capital income, taxes, entrepreneurial income, private pension contributions, public transfer payments, payouts, and socio-economic status or position.
The National Patient Register (NPR) [76]	1994–2012	All records of somatic hospital-treated in/outpatients. Primary, secondary and additional diagnoses for patients aged 16+	ICD-10 diagnosis codes for every public and private hospital-treated patient including every contact and treatment for the whole population, as well as a civil registration number.
			Moreover, data from all hospital treatments/procedures/operations for all hospital-treated patients as well as a civil registration number.
The Danish Psychiatric Central Research Register [77]	1995–2012	All psychiatric hospital-treated in/outpatients. Primary, secondary, and additional diagnoses for patients aged 16+	ICD-10 diagnosis codes for every public hospital-treated patient (no private psychiatric hospital exists) for all contacts and treatments for the whole population, as well as a civil registration number.
The National Health Service Registry (NHSR) [78]	2000–2012	All patients in primary care aged 16+	All GP services for the entire population and all consultations based on civil registration number. While the register does not contain information on diagnosis, many services are disease-specific, thus they can be used for identifying chronic conditions.
The Danish National Prescription Register (TNPR) [79]	1995–2012	All patients aged 16+ in primary care with a prescription	All Danish medicine prescriptions are sold for the entire population using 6-digit ATC codes as well as a civil registration number.
The Danish Civil Registration System [80]	2013	The whole population aged 16+ resident in Denmark on 1st January 2013	Data regarding birth date, gender, age etc., for the whole population as well as a civil registration number.

Partly adapted from Hvidberg et al. (2019) [56].

The Population’s Education Register (PER) [74] and The Income Statistics Register (ISR) [75] were used for identifying socioeconomic disparities regarding educational achievements, income level, and socioeconomic position. The Danish National Patient Register (NPR) [76], the Danish Psychiatric Central Research Register [77], The National Health Service Register (NHSR) [78] and The National Prescription Register (TNPR) [79] contained information on physician-reported ICD-10 hospital diagnoses, prescriptions, and general practice services. These data were used to identify the 199 chronic conditions by clinical recommendation [54, 55, 58]. Finally, sex, birth date, and other information originated from the Danish Civil Registration System [80]. The registers were linked together by the unique personal identification number assigned to every person [81]. The employed registers and quality are described elsewhere [54–56].

### Socioeconomic measures

Three measures of socioeconomic disparities were used: educational achievements, personal income, and socioeconomic position in society. All three variables were derived from Statistics Denmark PER and ISR registers (coming variable names are in brackets) for the year 2012 on 31 December. Education was measured as the highest archived education based on the International Standard Classification of Education, ISCED2011 (AUDD). Educational achievements were divided into five levels: 1. no education or training, 2. student, 3. shorter, 4. middle, and 5. higher education. Please note that students, who did not have any other achieved education, were classified separately using the information of their socioeconomic position (SOCIO02), into category 2 (student). This was done as students are usually younger, healthier, and thus have different health conditions than people with no education. Income was measured as personal income (DKR) after tax (DISPON_NY). Income was divided into quartiles to better allow for international, relative comparisons and reported as means. Finally, the socioeconomic position was measured based on standard classification (SOCIO02) into the following seven categories: 1. Retired, age, 2. Retirement, early retirement (health), 3. Sick leave/leave, 4. Unemployed/social benefits, 5. Unemployed minimum six months, 7. In training/study, 8. Employed, and 9. Others not in the workforce.

### Defining ’chronic condition’

A detailed description of the phases and methods used to define the 199 chronic conditions are provided elsewhere [54, 55, 58]. In summary, we defined a ’chronic condition’ according to the definition if the *’…condition had lasted or was expected to last twelve or more months and resulted in functional limitations and/or the need for functional limitations and/or the need for ongoing medical care’* [56, 82–84]. A clinical expert panel identified the ICD-10 diagnosis considered ’chronic’ based on the above definition [55]. The experts aggregated the ICD-10 diagnoses to 199 conditions, where several conditions included subgroups of different ICD-10 diagnoses; thus, some defined conditions contained several different conditions within related disease groups. Subsequently, all ICD-10 diseases considered chronic by definition included the full-population burden of chronic conditions [55, 56].

### The data algorithms identifying the chronic conditions in registers

Several chronic conditions do not last for a lifetime, although they last longer than the defined 12 months. The differing ’chronicity’ of the identified chronic conditions were divided into four groups of severity depending on how long they were expected to last [55]:

- **Category I**: Stationary to progressive chronic conditions (no time limit equals inclusion time going back from the time of interest for as long as valid data were available. In the current study, this starting point was defined by the introduction of the ICD-10 diagnosis coding in Denmark in 1994);
- **Category II**: Stationary to diminishing chronic conditions (10 years from register inclusion time to the time of interest);
- **Category III**: Diminishing chronic conditions (5 years from register inclusion time to the time of interest); and
- **Category IV**: Borderline chronic conditions (2 years from register inclusion time to the time of interest).

Adapted from Hvidberg et al. (2016, 2019) [55, 56].

This approach was developed to handle a well-known challenge of register research: if a condition is only identified once, for example, 10 or 25 years earlier, can researchers then be sure that the patient still suffers from this condition at present. Thus, to address this, all 199 chronic conditions were allocated into one of the four chronicity categories by medical experts. The allocation was based on a clinical judgment of how long the different chronic conditions with the best possible clinical certainty would still suffer from the condition from the time of interest, 1 January 2013. This new approach can also be used at different times of interest and as a proxy for severity.

An algorithm was employed based on the clinicians’ definitions and allocation of the 199 chronic conditions and related ICD-10 codes into one of the four categories. However, 35 chronic conditions were not judged by the medical experts to be appropriately representative using exclusively ICD-10 diagnosis. Thus, 35 more complex disease algorithms were suggested and employed using multiple registers, including medicine, hospital treatments, and general practice services. Furthermore, details of the 199 distinctive definitions, the medical experts and panel process, the allocation of conditions into the four categories, and the detailed algorithms for replication can be found in references [55, 58].

### Statistical analysis

Disease prevalence rates were calculated per thousand subjects, i.e., the number of conditions identified, divided by the total population (N = 4,555,439) multiplied by 1000. Hence, prevalence rates were calculated from a specific point in time, 1 January 2013, thus comprising conditions dating back from this point of time-based on the four inclusion time periods. Disease prevalence within educational levels, sex, or age were calculated as the number of conditions within the socioeconomic variable of interest, divided by the total of the socioeconomic variables, multiplied by 1000 (Tables 2 and 3 and S1 Table in S1 File). Ratios were calculated by dividing no educational achievement with high educational achievement prevalence rates. Per cent proportions within the diseases were calculated for levels of education, income quartiles, and socioeconomic position (Table 4, S1 and S2 Tables in S1 File) as the number of patients for each level of the socioeconomic variable of interest, divided by the total number of patients within the disease, multiplied by 100. Finally, nine multiple binary logistic regression models were carried out to control for potential confounding and residual correlations of the crude estimates (Table 5). This was done for the two key socioeconomic measures, educational levels, and income, hence one binary model for each of the five education levels and the income quartiles. All regression models included the 199 chronic conditions, gender and age as predictors.

**Table 2:** Overview of disease groups and medicine prevalence in Denmark: Number of patients, prevalence rates per thousands of the total population, prevalence rates per thousand within educational levels, age, and sex, on 1 January 2013.

		N, Prevalence and Ratios																			
Name of condition	ICD-10 code / definition	Total population*			Disease prevalence by education levels							Disease prevalence by sex and age–within no education or training									
					No education or training			Higher (MSc degree or doctorate)				Female		Male		Age 16–44		Age 45–74		Age 75+	
		*N*	*Per Thousand*		*N*	*Per Thousand*		*N*	*Per Thousand*		*Ratio*	*Per Thousand*				*Per Thousand*					
B–Viral hepatitis and human immunodeficiency virus [HIV] disease	B18, B20–B24	8,500	1.9	(1.9)	3,091	2.7	(3.1)	488	1.5	(0.8)	*1*.*8*	1.9		3.6		3.7		3.0		0.2	
C–Malignant neoplasms	C00–C99; D32–D33; D35.2–D35.4; D42–D44	229,331	50.3	(50.4)	76,501	67.8	(48.5)	13,755	42.8	(52.8)	*1*.*6*	79.2		55.2		10.0		76.5		145.1	
D–In situ and benign neoplasms, and neoplasms of unc. or unk. behaviour and diseases of the blood and blood-forming organs and diso. inv. the immune mechanism	D00–D09; D55–D59; D60–D67; D80–D89	116,560	25.6	(25.7)	40,941	36.3	(29.6)	6,187	19.3	(21.5)	*1*.*9*	45.5		26.0		16.4		33.0		80.3	
E–Endocrine, nutritional and metabolic diseases	E00–E14; E20–E29; E31–35; E70–E78; E84–E85; E88–E89	877,433	192.6	(192.7)	323,816	286.9	(225.8)	38,834	120.9	(146.8)	*2*.*4*	332.0		236.9		56.4		348.8		519.9	
G–Diseases of the nervous system	G00–G14; G20–G32; G35–G37; G40–47; G50–64; G70–73; G80–G83; G90–G99	561,054	123.2	(123.5)	186,070	164.9	(152.2)	28,699	89.3	(87.2)	*1*.*8*	192.7		134.0		108.2		192.5		187.4	
H–Diseases of the eye and adnexa and diseases of the ear and mastoid process	H02–H06; H17–H18; H25–H28; H31–H32; H34–H36; H40–55; H57; H80; H810; H93, H90–H93	448,176	98.4	(98.6)	162,019	143.6	(101.4)	24,682	76.8	(97.1)	*1*.*9*	163.1		121.8		33.4		128.5		379.4	
I–Diseases of the circulatory system	I05–I06; I10–28; I30–33; I36–141; I44–I52; I60–I88; I90–I94; I96–I99	1,254,427	275.4	(275.5)	444,581	393.9	(308.9)	60,629	188.7	(221.7)	*2*.*1*	453.8		327.4		87.3		453.2		768.1	
J–Diseases of the respiratory system	J30.1; J40–J47; J60–J84; J95, J97–J99	1,210,598	265.7	(266.3)	336,462	298.1	(278.0)	88,121	274.3	(275.5)	*1*.*1*	348.9		241.8		218.5		318.2		382.2	
K–Diseases of the digestive system	K25–K27; K40, K43, K50–52; K58–K59; K71-K77; K86-K87	329,337	72.3	(72.6)	113,926	100.9	(88.5)	15,438	48.1	(50.7)	*2*.*1*	114.1		86.3		57.8		107.2		159.4	
L–Diseases of the skin and subcut. tissue	L40	65,469	14.4	(14.5)	18,801	16.7	(14.7)	4,593	14.3	(14.4)	*1*.*2*	18.4		14.7		8.8		20.4		20.1	
M–Diseases of the musculoskeletal system and connective tissue	M01–M25; M30–M36; M40–M54; M60.1–M99	1,032,808	226.7	(227.1)	332,557	294.7	(246.7)	51,276	159.6	(179.6)	*1*.*8*	353.4		229.4		137.4		328.9		476.0	
N–Diseases of the genitourinary system	N18	20,162	4.4	(4.5)	8,621	7.6	(5.9)	713	2.2	(2.8)	*3*.*4*	7.0		8.4		1.7		6.8		20.5	
Q–Congenital malform., def & chrom abn	Q00–Q56; Q60–Q99	124,898	27.4	(27.5)	33,012	29.3	(32.6)	9,581	29.8	(26.4)	*1*.*0*	31.4		26.8		43.7		24.9		16.0	
F–Mental and behavioural disorders	F00–99	683,194	150.0	(150.7)	254,454	225.5	(222.3)	28,936	90.1	(85.4)	*2*.*5*	251.9		196.1		273.4		191.2		236.7	
Depression medicine ^c^ **	ATC: N06A.	529,918	116.3	(116.7)	191,590	70.9	(160.4)	22,768	169.8	(70.0)	2.4	212.1		122.8		161.0		158.4		216.9	
Antipsychotic medicine ^c^ **	ATC: N05A.	138,625	30.4	(30.6)	64,514	13.2	(56.7)	4,225	57.2	(11.8)	4.3	59.3		54.8		74.6		48.9		49.6	
Indication prescribed anxiety medicine ^c^ **	All prescribe. w. indication codes 163 (for anxiety) or 371 (for anxiety, addictive)	102,568	22.5	(22.6)	37,917	13.7	(33.3)	4,400	33.6	(12.7)	2.5	42.5		23.7		39.5		30.5		31.8	
Heart failure medication ^c^ **	ATC: C01AA05, C03, C07 or C09A with indication code 430 (for heart failure)	7,468	1.6	(1.7)	3,127	1.0	(2.0)	311	2.8	(1.2)	2.9	2.3		3.3		0.3		2.7		7.5	
Ischaemic heart medication ^c^ **	ATC: C01A, C01B, C01D, C01E.	129,484	28.4	(28.5)	57,571	13.5	(35.2)	4,324	51.0	(20.9)	3.8	54.6		47.0		2.4		45.3		152.3	
**All of the five types of medicine above**		**688,006**	**151.0**	**(151.6)**	**262,536**	**88.1**	**(92.4)**	**28,310**	**232.6**	**(211.0)**	**2.6**	**272.6**		**188.2**		**194.2**		**215.1**		**349.1**	
**Having one or more chronic conditions**		**2,989,441**	**656.2**	**(657.2)**	867,345	768.5	(713.2)	193,164	601.3	(604.2)	*1*.*3*	833.8		696.1		582.0		819.5		954.6	
**Mean numb. of chronic conditions [SD]**		**2.2**	**(2.2)**	**[2,8]**	**3.1**	**(2.6)**	**[3.3]**	**1.6**	**(1.7)**	**[2.1]**	**1.6**	**2.4**	**[2.9]**	**2.0**	**[2.6]**	**1.1**	**[1.6]**	**2.7**	**[2.8]**	**5.3**	**[3.6]**
**Total**		**4,555,439**	**-**	**-**	**1,128,588**	**1,000**	**N/A**	**321,254**	**1,000**	**N/A**	***1*.*0***	**1,000**		**1,000**		**1,000**		**1,000**		**1,000**	

Age and gender standardized estimates in brackets. [SD] = Standard Deviation.

ICD-10 International Statistical Classification of Diseases, 10^th^ Revision

^c^ = complex defined conditions, see reference for further details [55].

* Total population frequencies and prevalence adapted from Hvidberg et al. 2019 [56]

** 2-year prevalence.

**Table 3:** Overview of disease prevalence for 29 common conditions and overweight: Number of patients, overall prevalence rate per thousand, and estimates by educational level, age, and sex in Denmark on 1 January 2013.

		N and Prevalence														
Name of condition	ICD-10 code / definition	Total population*			Disease prevalence by education							Disease prevalence by sex and age—within no education or training				
					No Education or Training			Higher (MSc degree or doctorate)				Female	Male	Age 16–44	Age 45–74	Age 75+
		*N*	*Per thousand*		*N*	*Per thousand*		*N*	*Per Thousand*		*Ratio*	*Per thousand*		*Per thousand*		
Cancers	C00–C99; D32–D33; D35.2–D35.4; D42–D44	229,331	50.3	(50.4)	76,501	67.8	(48.5)	13,755	42.8	(52.8)	*1*.*6*	79.2	55.2	10.0	76.5	145.1
Type 1 diabetes ^c^	E10	23,062	5.1	(5.1)	6,012	5.3	(5.5)	1,541	4.8	(4.4)	*1*.*1*	4.5	6.2	6.1	5.5	3.4
Type 2 diabetes ^c^	E11	242,177	53.2	(53.3)	102,366	90.7	(73.6)	7,735	24.1	(29.1)	*3*.*8*	93.9	87.2	14.1	112.7	164.1
Migraine ^c^	G43	149,866	32.9	(33.0)	38,243	33.9	(34.9)	10,093	31.4	(29.6)	*1*.*1*	52.5	13.2	27.4	44.5	15.6
Other headache syndromes	G44	16,469	3.6	(3.6)	4,690	4.2	(4.6)	828	2.6	(2.0)	*1*.*6*	5.0	3.2	5.7	4.0	1.9
Diseases of the eye lens (cataracts)	H25–H28	68,009	14.9	(15.1)	29,764	26.4	(16.3)	2,743	8.5	(14.3)	*3*.*1*	34.1	17.8	0.8	20.8	87.0
Tinnitus	H931	40,124	8.8	(8.7)	12,187	10.8	(8.9)	2,657	8.3	(8.6)	*1*.*3*	9.7	12.0	2.5	13.8	17.0
Ischaemic Heart Diseases broad	I05-I06; I11-I13; I20-I28; I30-I52	315,901	69.3	(69.3)	120,791	107.0	(78.8)	15,151	47.2	(58.6)	*2*.*3*	106.9	107.2	18.2	104.7	269.9
Hypertensive diseases ^c^	I10–I15	1,060,046	232.7	(232.7)	391,287	346.7	(263.7)	47,086	146.6	(182.6)	*2*.*4*	408.6	278.0	51.5	401.4	713.8
Heart failure ^c^	I11.0, I13.0, I13.2, I42.0, I42.6, I42.7, I42.9, I50.0, I50.1, I50.9	37,540	8.2	(8.3)	15,906	14.1	(10.2)	1,482	4.6	(6.1)	*3*.*1*	11.9	16.5	1.3	13.1	39.3
Ischaemic heart diseases specific	I20–I25	139,173	30.6	(30.7)	56,244	49.8	(37.7)	5,320	16.6	(20.9)	*3*.*0*	45.5	54.7	4.7	54.8	115.3
Angina pectoris	I20	78,476	17.2	(17.3)	30,898	27.4	(21.4)	3,136	9.8	(11.8)	*2*.*8*	25.6	29.4	3.0	33.4	53.6
Stroke	I60, I61, I63–I64, Z501 (rehabilitation)	72,606	15.9	(16.0)	29,883	26.5	(19.2)	2,783	8.7	(11.7)	*3*.*1*	26.9	26.0	2.7	26.6	68.0
Respiratory allergy ^c^	J30, except J30.0	841,685	184.8	(185.2)	207,831	184.2	(172.6)	70,172	218.4	(218.2)	*0*.*8*	221.9	142.2	142.2	195.3	226.9
Chronic lower respiratory diseases ^c^	J40–J43, J47	418,120	91.8	(92.0)	136,583	121.0	(110.3)	27,404	85.3	(83.4)	*1*.*4*	147.2	92.0	68.5	137.0	169.0
Chronic obstructive lung disease (COPD) ^c^	J44, J96, J13–J18	216,184	47.5	(47.6)	88,085	78.0	(64.7)	7,600	23.7	(30.1)	*3*.*3*	89.3	65.5	23.9	88.6	143.9
Asthma, status asthmaticus ^c^	J45–J46	361,129	79.3	(79.4)	115,623	102.4	(96.5)	21,606	67.3	(69.4)	*1*.*5*	120.8	82.1	77.6	107.5	132.2
Arthritis	M1-M3; M5-M9; M7-M14; M15-M20; M45	505,792	111.0	(111.0)	175,514	155.5	(120.9)	23,321	72.6	(87.3)	*2*.*1*	183.1	124.9	34.0	183.2	292.1
Inflammatory polyarthropathies and ankylosing spondylitis ^c^	M05–M14, M45	165,944	36.4	(36.5)	55,657	49.3	(40.8)	9,676	30.1	(31.9)	*1*.*6*	50.8	47.6	13.9	59.2	84.2
Rheumatoid arthritis ^c^	M05, M06, M07.1, M07.2, M07.3, M08, M09	77,345	17.0	(17.0)	24,192	21.4	(18.0)	5,714	17.8	(19.1)	*1*.*2*	29.2	12.8	7.6	26.5	31.8
Arthrosis	M15-M19	338,166	74.2	(74.2)	125,325	111.0	(81.8)	12,528	39.0	(53.3)	*2*.*8*	134.6	84.8	14.6	128.7	231.3
Gonarthrosis [arthrosis of knee]	M17	178,811	39.3	(39.4)	64,860	57.5	(42.4)	6,420	20.0	(27.3)	*2*.*9*	71.1	42.3	6.4	69.2	114.5
Back conditions	M32-34; M41-M43; M46-49; M50-51; M53-54	212,948	46.7	(46.7)	70,622	62.6	(55.8)	9,814	30.5	(31.1)	*2*.*0*	70.6	53.6	36.2	72.0	82.8
Osteoporosis ^c^	M80–M81	158,813	34.9	(34.8)	68,692	60.9	(36.0)	5,055	15.7	(33.2)	*3*.*9*	101.6	15.6	1.1	56.9	177.0
Dementia ^c^	F00, G30, F01, F02.0, F03.9, G31.8B, G31.8E, G31.9, G31.0B	36,803	8.1	(8.1)	18,319	16.2	(8.8)	1,066	3.3	(7.5)	*4*.*9*	22.0	9.8	0.1	5.5	74.5
Schizophrenia ^c^	F20	29,422	6.5	(6.5)	15,920	14.1	(15.3)	776	2.4	(0.8)	*5*.*8*	11.1	17.5	24.2	11.8	2.8
Depression ^c^	F32, F33, F34.1, F06.32	454,933	99.9	(100.2)	161,524	143.1	(136.8)	19,825	61.7	(59.3)	*2*.*3*	180.4	101.7	142.5	130.6	179.1
Other anxiety disorders	F41	38,079	8.4	(8.4)	14,581	12.9	(14.2)	1,686	5.2	(3.8)	*2*.*5*	16.2	9.3	22.0	9.8	5.8
Hyperkinetic disorders (ADHD) ^c^	F90	42,908	9.4	(9.5)	19,548	17.3	(19.7)	1,048	3.3	(3.6)	*5*.*3*	12.0	23.3	47.7	4.1	0.9
Overweight, clinical (BMI>35)	E66	220,928	48.5	(48.5)	68,152	60.4	(67.3)	8,875	27.6	(14.7)	*2*.*2*	88.5	29.2	72.1	62.8	33.0
**All the above conditions**	**-**	**2,564,764**	**563.0**	**(563.0)**	**160,348**	**679.3**	**(616.0)**	**766,694**	**499.1**	**(506.5)**	***1*.*4***	**762.4**	**587.2**	**457.8**	**866.2**	**1386.4**

Age and gender standardized estimates in brackets.

ICD-10 International Statistical Classification of Diseases, 10^th^ Revision

^c^ = complex defined conditions, see reference for further details [55].

* Total population frequencies and prevalence adapted from Hvidberg et al. 2019 [56].

**Table 4:** Catalogue of socioeconomic differences of 199 chronic conditions: Prevalence rates per hundred of educational and income levels (per cent within conditions) and mean incomes, in Denmark on 1 January 2013.

No.	Name of condition	ICD-10 code / definition	N total*	Prevalence of education levels–within conditions											Prevalence of Income quartiles–within conditions								Mean income–within conditions		
				No education or training		Students or in training		Shorter		Middle (BSc or equal)		Higher (MSc degree or doctorate)			1st quartile		2nd quartile		3rd quartile		4th quartile				
				%	Std	%	Std	%	Std	%	Std	%	Std	Rank	%	Std	%	Std	%	Std	%	Std	Mean	Std mean	SD
	**B–Viral hepatitis and human immunodeficiency virus [HIV] disease**	**B18, B20–B24**	**8,500**	**36.4**	**(36.9)**	**0.9**	(**3.7)**	**37.2**	(**32.9)**	**10.2**	(**10.5)**	**5.7**	(**4.7)**	**86**	**22.1**	(**31.8)**	**39.6**	**(36.6)**	**22.3**	(**19.3)**	**14.5**	(**10.6)**	**185,881.4**	**(165,718.5)**	**115,321**
1	Chronic viral hepatitis	B18	4,584	46.5	(43.6)	1.1	(4.0)	28.9	(27.4)	8.4	(8.4)	3.5	(3.1)	13	26.5	(35.5)	46.2	(41.0)	17.8	(15.0)	8.2	(6.7)	166,002.3	(153,636.2)	149,137
2	Human immunodeficiency virus [HIV] disease	B20–24	4,229	25.4	(29.9)	0.5	(3.2)	45.9	(38.7)	11.9	(13.2)	8.2	(6.3)	196	17.6	(27.0)	32.7	(32.2)	26.8	(24.3)	21.1	(14.5)	209,183.8	(182,093.1)	182,701
	**C–Malignant neoplasms**	**C00–C99; D32–D33; D35.2–D35.4; D42–D44**	**229,331**	**33.4**	(**25.7)**	**0.2**	(**3.4)**	**42.7**	(**46.0)**	**14.8**	(**15.3)**	**6.0**	(**7.5)**	**124**	**20.2**	(**23.2)**	**34.8**	(**26.7)**	**23.1**	(**24.7)**	**21.8**	(**24.9)**	**209,459.6**	(**210,490.5)**	**187,044**
3	Malignant neoplasms of other and unspecified localizations	C00–C14; C30–C33; C37–C42; C45–C49; C69; C73–74; C754–C759	20,557	32.6	(26.2)	0.4	(3.7)	44.3	(45.7)	13.7	(14.5)	5.9	(6.8)	135	19.0	(24.3)	35.4	(28.8)	23.4	(24.1)	21.9	(22.3)	210,585.2	(203,185.7)	198,068
4	Malignant neoplasms of digestive organs	C15–C17; C22–C26	4,839	37.7	(32.7)	0.2	(5.4)	43.2	(42.0)	11.0	(13.8)	4.4	(4.0)	65	23.0	(25.7)	39.4	(31.9)	20.6	(23.9)	16.7	(17.9)	194,684.2	(190,194.8)	157,317
5	Malignant neoplasm of colon	C18	18,826	38.4	(25.2)	0.1	(4.4)	40.8	(46.3)	11.8	(13.9)	5.0	(7.4)	61	22.3	(25.0)	39.6	(28.1)	20.0	(22.4)	17.9	(24.3)	197,588.8	(204,384.1)	171,911
6	Malignant neoplasms of rectosigmoid junction, rectum, anus and anal canal	C19–C21	10,680	37.6	(28.9)	0.0	(0.0)	42.7	(47.9)	11.8	(13.1)	5.0	(7.9)	67	20.9	(27.4)	38.5	(26.9)	21.2	(21.4)	19.3	(24.0)	206,324.2	(206,536.1)	247,900
7	Malignant neoplasm of bronchus and lung	C34	14,762	42.4	(35.6)	0.0	(4.7)	41.9	(42.6)	9.7	(10.3)	3.3	(3.8)	31	24.0	(26.2)	42.4	(32.8)	20.1	(24.5)	13.4	(16.4)	184,048.4	(186,639.4)	149,064
8	Malignant melanoma of skin	C43	19,636	23.0	(19.8)	0.3	(2.3)	46.4	(48.5)	19.1	(18.2)	9.1	(9.8)	206	15.3	(20.0)	25.9	(22.2)	26.2	(25.4)	32.5	(32.1)	243,621.5	(235,782.6)	238,641
9	Other malignant neoplasms of skin	C44	15,597	33.4	(22.7)	0.0	(2.1)	41.2	(47.7)	13.6	(16.7)	6.6	(8.4)	125	20.0	(21.3)	35.5	(23.6)	21.9	(26.1)	22.5	(28.9)	215,542.8	(222,082.0)	211,225
10	Malignant neoplasm of breast	C50	50,687	34.3	(22.4)	0.0	(0.7)	39.8	(48.4)	18.7	(15.5)	4.8	(5.4)	115	22.0	(12.5)	35.2	(27.6)	23.8	(31.2)	18.8	(23.2)	195,647.7	(210,432.2)	132,603
11	Malignant neoplasms of female genital organs	C51–C52; C56–C58	7,245	36.9	(26.2)	0.2	(2.3)	38.4	(38.0)	17.0	(10.3)	4.9	(2.4)	77	21.9	(30.0)	36.6	(25.6)	23.9	(14.5)	17.3	(9.9)	192,190.6	(134,658.5)	135,117
12	Malignant neoplasm of cervix uteri, corpus uteri and part unspecified	C53–C55	11,608	37.5	(13.6)	0.0	(0.1)	38.4	(22.1)	17.1	(10.1)	4.3	(2.6)	70	21.8	(11.2)	36.1	(14.9)	24.7	(13.6)	17.2	(9.4)	191,219.6	(95,452.2)	108,491
13	Malignant tumour of male genitalia	C60, C62–C63	5,194	21.5	(20.1)	0.5	(1.1)	54.2	(41.4)	11.7	(12.5)	10.3	(4.3)	214	12.9	(27.6)	20.2	(17.9)	27.6	(15.4)	38.9	(19.2)	260,128.0	(162,140.6)	236,578
14	Malignant neoplasm of prostate	C61	26,697	30.5	(29.5)	0.0	(0.0)	46.1	(34.8)	12.4	(11.1)	8.3	(4.2)	158	17.4	(15.6)	34.1	(20.3)	22.0	(26.9)	26.3	(17.3)	231,862.4	(180,963.1)	245,928
15	Malignant neoplasms of urinary tract	C64–C68	10,319	38.4	(29.7)	0.1	(2.4)	43.6	(44.8)	9.9	(13.5)	4.6	(7.1)	62	22.5	(27.5)	38.7	(29.7)	21.1	(21.7)	17.5	(20.8)	196,074.2	(194,578.5)	149,060
16	Brain cancer ^c^	C71, C75.1–C75.3, D33.0–D33.2, D35.2–D35.4, D43.0–D43.2, D44.3–D44.5 (brain). C70, D32, D42 (brain membrane). C72, D33.3–D33.9, D43.3–D43.9 (cranial nerve, spinal cord)	15,310	31.7	(30.0)	0.8	(4.0)	44.6	(43.3)	14.2	(14.1)	6.3	(6.6)	146	19.1	(23.7)	34.2	(30.6)	24.0	(23.8)	22.4	(21.4)	210,068.5	(199,939.6)	161,877
17	Malignant neoplasms of ill-defined, secondary and unspecified sites, and of independent (primary) multiple sites	C76–C80, C97	25,619	34.1	(26.8)	0.2	(2.7)	43.0	(46.7)	14.8	(14.7)	5.6	(6.9)	118	20.6	(23.9)	35.3	(28.3)	23.1	(24.6)	20.8	(22.7)	205,785.3	(204,595.3)	177,825
18	Malignant neoplasms, stated or presumed to be primary, of lymphoid, haematopoietic and related tissue	C81–C96	19,712	33.1	(26.2)	0.9	(3.9)	42.8	(45.1)	14.1	(15.5)	6.5	(7.3)	129	21.7	(24.5)	33.8	(27.5)	22.8	(24.8)	21.4	(22.6)	208,860.4	(204,521.4)	187,060
	**D–In situ and benign neoplasms, and neoplasms of uncertain or unknown behaviour and diseases of the blood and blood-forming organs and certain disorders involving the immune mechanism**	**D00–D09; D55–D59; D60–D67; D80–D89**	**116,560**	**35.1**	(**30.0)**	**0.8**	(**3.6)**	**40.5**	(**44.3)**	**13.5**	(**13.1)**	**5.3**	(**5.9)**	**107**	**19.8**	(**24.2)**	**37.6**	(**30.9)**	**24.1**	(**24.3)**	**18.2**	(**20.1)**	**198,011.7**	**196,845.8)**	**158,521**
19	In situ neoplasms	D00–D09	19,810	26.8	(24.2)	0.1	(0.8)	44.9	(49.3)	19.0	(15.4)	7.0	(8.3)	186	15.7	(18.2)	30.8	(27.4)	28.9	(25.7)	24.5	(27.9)	216,497.4	(226,442.2)	147,992
20	Haemolytic anaemias	D55–D59	3,055	32.6	(30.8)	2.8	(4.8)	39.2	(40.8)	12.7	(11.5)	6.4	(6.7)	136	25.7	(27.8)	33.6	(31.6)	24.7	(23.2)	15.3	(16.4)	184,357.0	(185,217.7)	129,266
21	Aplastic and other anaemias	D60–D63	14,918	40.5	(33.2)	0.4	(4.3)	37.3	(41.5)	11.2	(12.2)	4.1	(4.7)	39	21.0	(25.7)	43.1	(34.0)	21.5	(23.1)	14.1	(16.6)	186,736.3	(185,062.3)	132,903
22	Other anaemias	D64	46,613	44.2	(37.6)	0.3	(3.1)	35.1	(40.7)	9.7	(10.3)	3.2	(3.9)	18	21.9	(26.3)	46.7	(37.5)	19.9	(22.0)	11.3	(13.7)	179,721.0	(179,593.3)	140,017
23	Coagulation defects, purpura and other haemorrhagic conditions	D65–D69	25,376	27.0	(27.6)	1.9	(3.6)	45.0	(45.7)	16.2	(14.1)	7.5	(6.8)	185	19.2	(23.8)	29.3	(28.2)	27.3	(24.9)	23.8	(22.7)	210,815.8	(203,148.6)	163,700
24	Other diseases of blood and blood-forming organs	D70–D77	8,896	34.8	(32.0)	0.8	(3.8)	42.9	(43.7)	13.8	(13.0)	5.1	(5.1)	108	20.8	(25.9)	35.2	(31.0)	24.7	(24.7)	19.2	(18.1)	203,800.9	(193,482.6)	229,667
25	Certain disorders involving the immune mechanism	D80–D89	7,660	27.7	(28.6)	1.3	(4.0)	46.5	(45.1)	15.3	(14.0)	7.3	(6.4)	182	17.1	(23.2)	27.9	(28.1)	28.1	(25.8)	26.6	(22.5)	221,264.4	(203,467.9)	165,269
	**E–Endocrine, nutritional and metabolic diseases**	**E00–E14; E20–E29; E31–35; E70–E78; E84–E85; E88–E89**	**877,433**	**36.9**	(**30.6)**	**0.4**	(**4.4)**	**42.9**	(**44.4)**	**12.4**	(**12.6)**	**4.4**	(**5.2)**	**78**	**21.3**	(**25.2)**	**36.4**	(**29.6)**	**22.9**	(**24.7)**	**19.1**	(**20.0)**	**201,407.0**	(**195,975.4)**	**185,470**
26	Diseases of the thyroid ^c^	E00–E04, E06, E07	131,908	33.5	(28.0)	0.6	(4.3)	40.1	(42.8)	16.9	(14.2)	5.3	(6.8)	121	20.2	(23.9)	34.3	(28.0)	25.5	(25.4)	19.7	(22.0)	200,252.3	(202,142.7)	156,674
27	Thyrotoxicosis ^c^	E05	41,374	39.1	(29.6)	0.4	(3.6)	38.6	(44.6)	13.7	(13.0)	4.5	(5.8)	55	20.8	(24.5)	38.0	(28.8)	23.8	(25.1)	17.3	(21.1)	192,409.8	(197,446.5)	121,776
28	Type 1 diabetes ^c^	E10	23,062	26.1	(27.1)	4.1	(5.0)	47.8	(46.3)	13.8	(13.8)	6.7	(6.2)	194	21.7	(24.7)	27.5	(28.4)	26.5	(25.4)	23.9	(21.0)	209,460.0	(198,589.9)	179,737
29	Type 2 diabetes ^c^	E11	242,177	42.3	(38.9)	0.0	(2.8)	41.3	(41.6)	9.4	(9.8)	3.2	(3.4)	33	23.0	(25.6)	41.0	(35.4)	21.1	(23.7)	14.6	(14.8)	187,307.6	(182,176.4)	165,960
30	Diabetes others ^c^	E12–E14	1,117	34.6	(32.0)	3.1	(7.6)	41.5	(41.5)	11.5	(11.0)	4.7	(4.1)	111	24.4	(29.9)	36.3	(32.4)	23.7	(23.0)	15.0	(14.1)	193,832.0	(179,037.5)	357,817
31	Disorders of other endocrine glands	E20–E35, except E30	28,650	28.9	(29.1)	3.9	(5.1)	41.9	(43.2)	16.3	(13.9)	6.3	(6.2)	170	24.7	(25.7)	32.2	(30.4)	25.3	(24.2)	17.3	(19.2)	188,791.1	(192,042.4)	125,418
32	Metabolic disorders	E70–E77; E79–E83; E85, E88–E89;	23,690	33.2	(31.7)	1.4	(3.6)	41.7	(42.7)	14.6	(13.2)	5.9	(5.9)	127	20.9	(24.7)	34.4	(31.8)	25.0	(23.7)	19.4	(19.3)	200,403.0	(195,800.9)	166,430
33	Disturbances in lipoprotein circulation and other lipids ^c^	E78	652,242	38.7	(33.7)	0.0	(3.0)	43.5	(45.6)	11.3	(11.4)	3.9	(3.9)	58	21.9	(24.3)	37.5	(32.4)	21.9	(24.4)	18.6	(18.5)	201,180.6	(194,711.7)	192,923
34	Cystic fibrosis ^c^	E84	947	24.7	(30.2)	6.1	(5.2)	36.0	(37.3)	16.5	(13.7)	14.8	(11.5)	201	22.1	(23.7)	26.7	(30.1)	25.5	(22.6)	25.6	(23.2)	220,652.4	(219,314.1)	197,699
	**G–Diseases of the nervous system**	**G00–G14; G20–G32; G35–G37; G40–47; G50–64; G70–73; G80–G83; G90–G99**	**561,054**	**33.2**	(**32.5)**	**0.9**	(**3.7)**	**43.9**	(**44.0)**	**14.2**	(**12.4)**	**5.1**	(**4.9)**	**128**	**17.9**	(**23.3)**	**33.9**	(**31.6)**	**27.2**	(**25.6)**	**20.8**	(**19.1)**	**206,768.8**	(**196,126.1)**	**168,239**
35	Inflammatory diseases of the central nervous system	G00–G09	7,642	30.0	(30.1)	1.1	(3.3)	44.9	(44.5)	14.8	(13.7)	6.8	(6.2)	162	17.9	(22.6)	30.8	(29.6)	27.3	(25.8)	23.6	(21.4)	215,477.5	(203,767.3)	162,961
36	Systemic atrophies primarily affecting the central nervous system and other degenerative diseases	G10–G14, G30–G32	10,401	40.5	(36.3)	0.2	(2.8)	38.1	(39.6)	10.6	(11.9)	5.2	(6.2)	40	18.0	(17.6)	44.7	(36.9)	23.5	(26.9)	13.6	(18.1)	191,221.2	(200,525.2)	125,966
37	Parkinson’s disease ^c^	G20, G21, G22, F02.3	57,583	47.1	(51.2)	0.1	(1.8)	35.8	(32.6)	9.8	(7.8)	3.3	(2.9)	12	19.0	(21.3)	46.3	(45.8)	24.1	(23.5)	10.4	(9.2)	182,597.1	(174,997.3)	124,107
38	Extrapyramidal and movement disorders	G23–G26	10,837	36.9	(34.3)	0.8	(3.2)	40.1	(41.5)	13.8	(13.1)	5.2	(5.5)	79	20.8	(23.6)	38.2	(34.0)	24.5	(25.4)	16.3	(16.7)	196,091.1	(191,902.3)	235,993
39	Sclerosis	G35	13,284	26.6	(28.3)	0.2	(2.1)	47.3	(47.9)	17.7	(13.9)	7.0	(6.3)	188	11.1	(19.1)	29.9	(30.1)	33.7	(29.4)	25.1	(21.0)	222,561.2	(206,034.8)	178,878
40	Demyelinating diseases of the central nervous system	G36–G37	4,571	25.3	(28.4)	0.6	(2.8)	49.0	(47.4)	16.6	(13.6)	7.2	(6.3)	198	13.2	(21.3)	28.0	(29.5)	31.9	(26.7)	26.5	(22.2)	224,055.6	(206,896.1)	148,961
41	Epilepsy ^c^	G40–G41	61,695	43.5	(43.3)	2.3	(4.4)	36.5	(35.4)	10.3	(9.7)	3.6	(3.4)	22	20.6	(23.9)	41.7	(40.1)	25.9	(24.9)	11.5	(10.8)	181,782.4	(175,203.8)	111,502
42	Migraine ^c^	G43	149,866	25.5	(26.8)	1.1	(3.7)	45.7	(46.5)	19.3	(14.5)	6.7	(6.6)	195	16.5	(23.9)	27.7	(27.1)	29.8	(24.9)	25.8	(23.6)	218,092.9	(208,560.9)	163,491
43	Other headache syndromes	G44	16,469	28.5	(30.8)	2.9	(4.1)	46.1	(45.1)	14.8	(12.7)	5.0	(4.5)	173	22.1	(26.7)	29.9	(29.8)	26.5	(23.5)	21.2	(19.6)	203,001.0	(196,174.4)	175,122
44	Transient cerebral ischaemic attacks and related syndromes and vascular syndromes of brain in cerebrovascular diseases	G45–G46	43,977	37.5	(32.4)	0.0	(2.2)	41.9	(45.1)	12.0	(13.0)	4.7	(4.9)	71	20.7	(22.0)	38.7	(31.0)	21.6	(25.3)	18.8	(21.4)	202,406.6	(203,666.0)	191,294
45	Sleep disorders	G47	36,806	26.5	(28.7)	0.7	(3.9)	51.0	(46.4)	13.6	(13.7)	6.1	(5.4)	191	15.2	(23.7)	28.2	(29.6)	27.8	(26.1)	28.7	(20.4)	231,721.9	(199,498.8)	227,421
46	Disorders of trigeminal nerve and facial nerve disorders	G50–G51	21,488	31.4	(29.6)	0.7	(3.3)	44.9	(46.0)	14.5	(13.1)	5.6	(5.5)	150	18.9	(24.4)	31.9	(28.7)	25.6	(24.5)	23.3	(21.7)	213,377.8	(202,591.8)	192,801
47	Disorders of other cranial nerves, cranial nerve disorders in diseases classified elsewhere, nerve root and plexus disorders and nerve root and plexus compressions in diseases classified elsewhere	G52–G55	12,429	31.6	(30.6)	0.2	(2.1)	47.5	(48.9)	13.2	(11.7)	5.1	(4.7)	147	16.2	(23.8)	33.6	(31.5)	27.3	(25.0)	22.6	(19.3)	215,625.1	(197,938.9)	176,042
48	Mononeuropathies of upper limb	G56	122,395	35.3	(33.1)	0.2	(2.1)	46.3	(49.0)	12.5	(10.4)	3.2	(2.9)	105	17.1	(22.4)	35.4	(32.8)	28.6	(27.5)	18.7	(17.0)	201,466.0	(191,491.8)	147,007
49	Mononeuropathies of lower limb, other mononeuropathies and mononeuropathy in diseases classified elsewhere	G57–G59	18,627	30.9	(28.7)	0.3	(2.7)	47.2	(48.3)	14.9	(13.6)	4.8	(4.8)	154	17.1	(23.4)	31.9	(29.0)	26.8	(25.4)	24.0	(21.8)	216,078.3	(202,252.5)	157,883
50	Polyneuropathies and other disorders of the peripheral nervous system	G60–G64	30,289	36.1	(33.5)	0.3	(3.4)	43.4	(43.8)	12.1	(11.7)	5.0	(5.0)	91	19.3	(23.5)	39.7	(34.9)	23.4	(24.7)	17.4	(16.6)	201,276.6	(191,316.9)	201,814
51	Diseases of myoneural junction and muscle	G70–G73	5,758	33.0	(33.1)	1.8	(3.9)	42.8	(41.9)	14.0	(13.0)	6.7	(6.4)	130	17.8	(20.6)	32.3	(31.0)	27.6	(27.0)	21.9	(21.0)	211,707.7	(207,704.3)	177,215
52	Cerebral palsy and other paralytic syndromes	G80–G83	14,410	47.5	(48.2)	3.0	(3.9)	32.2	(31.0)	9.4	(9.2)	3.7	(3.6)	11	15.3	(16.5)	36.7	(37.1)	31.1	(30.4)	16.7	(15.8)	200,721.5	(196,850.6)	134,307
53	Other disorders of the nervous system	G90–G99	44,394	33.3	(33.1)	1.3	(3.7)	43.8	(43.1)	13.3	(12.2)	5.5	(5.3)	126	19.3	(23.4)	34.8	(32.4)	26.0	(25.2)	19.6	(18.7)	202,203.8	(193,950.3)	164,778
	**H–Diseases of the eye and adnexa and diseases of the ear and mastoid process**	**H02–H06; H17–H18; H25–H28; H31–H32; H34–H36; H40–55; H57; H80, H810; H93, H90–H93**	**448,176**	**36.2**	(**28.6)**	**0.5**	(**3.9)**	**40.2**	(**43.6)**	**13.0**	(**14.1)**	**5.5**	(**7.0)**	**88**	**21.1**	(**23.6)**	**37.5**	(**28.5)**	**22.1**	(**24.8)**	**19.1**	(**22.6)**	**201,140.2**	(**204,253.6)**	**179,853**
54	Disorders of eyelid, lacrimal system and orbit	H02–H06	13,191	32.9	(27.1)	0.5	(4.3)	42.5	(45.2)	15.1	(14.1)	5.6	(6.4)	131	19.1	(23.7)	33.2	(26.0)	24.2	(24.9)	23.2	(24.8)	218,165.1	(215,514.6)	235,844
55	Corneal scars and opacities	H17	2,173	33.8	(30.8)	0.6	(2.2)	44.8	(46.8)	11.2	(12.0)	4.6	(4.7)	120	22.5	(24.2)	32.9	(29.4)	23.2	(25.5)	20.8	(20.2)	199,686.1	(196,307.5)	158,416
56	Other disorders of cornea	H18	9,473	32.4	(26.0)	0.5	(2.9)	40.8	(44.5)	14.2	(15.1)	6.9	(8.0)	139	20.3	(23.1)	33.9	(26.4)	23.3	(24.7)	22.2	(25.2)	211,249.9	(214,018.0)	237,806
57	Diseases of the eye lens (cataracts)	H25–H28	68,009	43.8	(32.3)	0.1	(4.7)	37.0	(41.4)	11.2	(12.3)	4.0	(5.2)	21	25.8	(25.6)	42.4	(31.5)	18.5	(24.7)	13.1	(17.7)	182,302.8	(190,558.8)	167,124
58	Disorders of the choroid and retina	H31–H32	1,900	30.6	(28.0)	0.7	(3.2)	42.7	(43.2)	15.7	(16.2)	6.1	(6.6)	156	20.8	(23.5)	31.8	(28.2)	24.3	(24.8)	23.2	(23.2)	210,410.9	(210,438.8)	345,010
59	Retinal vascular occlusions	H34	10,358	39.1	(28.1)	0.1	(3.4)	39.3	(44.7)	11.4	(14.6)	5.5	(6.5)	56	21.8	(25.3)	40.5	(28.7)	20.0	(23.4)	17.6	(22.4)	198,213.7	(201,452.3)	184,876
60	Other retinal disorders	H35	68,485	40.5	(28.7)	0.1	(3.3)	36.2	(44.1)	11.2	(14.0)	4.7	(7.1)	41	21.8	(23.2)	42.4	(28.6)	19.9	(24.6)	15.8	(23.2)	192,829.7	(206,354.6)	186,243
61	Retinal disorders in diseases classified elsewhere	H36	19,279	35.7	(31.9)	0.4	(2.8)	44.4	(45.7)	11.4	(12.1)	4.9	(5.1)	97	21.6	(25.9)	36.9	(32.8)	23.2	(24.2)	18.0	(16.8)	195,883.3	(186,071.6)	143,600
62	Glaucoma ^c^	H40–H42	67,310	38.5	(28.4)	0.2	(4.8)	38.9	(43.6)	12.1	(13.3)	5.0	(6.8)	59	22.3	(23.3)	39.7	(29.3)	20.8	(25.0)	17.0	(21.8)	196,806.7	(205,907.8)	181,431
63	Disorders of the vitreous body and globe	H43–H45	7,572	26.6	(22.6)	0.6	(3.3)	42.4	(45.4)	17.8	(16.4)	9.8	(9.8)	189	18.6	(22.6)	29.6	(26.0)	24.4	(24.4)	27.1	(26.4)	226,202.8	(217,127.6)	183,622
64	Disorders of optic nerve and visual pathways	H46–H48	6,184	29.2	(31.5)	1.3	(2.9)	45.4	(44.1)	15.1	(13.0)	6.6	(5.9)	166	16.8	(21.2)	32.4	(32.4)	28.0	(25.8)	22.6	(20.3)	214,777.9	(204,663.7)	239,339
65	Disorders of ocular muscles, binocular movement, accommodation and refraction	H49–H52	18,247	21.6	(23.8)	2.6	(3.9)	45.7	(45.0)	17.0	(15.3)	11.1	(9.8)	213	17.5	(21.7)	23.0	(24.4)	26.8	(24.5)	32.3	(29.0)	236,638.5	(225,324.3)	214,224
66	Visual disturbances	H53	22,232	34.0	(31.5)	0.9	(3.5)	41.6	(42.6)	14.1	(13.6)	6.2	(6.2)	119	19.4	(23.5)	34.9	(30.7)	24.6	(24.5)	20.9	(21.0)	207,270.6	(201,428.0)	203,147
67	Blindness and partial sight	H54	6,614	43.2	(43.3)	0.9	(3.0)	33.9	(35.2)	9.3	(9.1)	4.2	(4.6)	26	19.3	(21.0)	42.9	(38.0)	24.6	(27.0)	12.9	(13.7)	186,664.3	(186,743.7)	109,931
68	Nystagmus and other irregular eye movements and other disorders of eye and adnexa	H55, H57	11,133	29.0	(28.2)	1.9	(4.3)	41.2	(41.2)	16.5	(15.2)	8.3	(8.3)	168	20.2	(24.0)	29.6	(27.2)	25.3	(24.5)	24.5	(23.9)	214,563.5	(208,663.6)	183,153
69	Otosclerosis	H80	10,360	31.5	(25.1)	0.6	(4.8)	42.1	(44.8)	16.4	(15.1)	6.2	(7.5)	148	19.0	(24.1)	32.7	(25.5)	24.9	(25.2)	23.2	(24.5)	210,338.0	(205,889.7)	142,402
70	Ménière’s disease ^c^	H810	10,003	35.6	(27.2)	0.1	(2.4)	42.5	(48.3)	13.8	(13.9)	4.9	(6.4)	100	20.0	(23.5)	36.2	(26.6)	23.3	(25.6)	20.4	(23.9)	207,492.0	(209,921.9)	190,808
71	Other diseases of the inner ear	H83	29,865	35.8	(31.4)	0.1	(3.2)	50.8	(46.2)	7.8	(11.5)	2.9	(5.5)	95	20.1	(23.2)	39.7	(30.7)	22.7	(25.2)	17.3	(20.3)	195,326.1	(195,450.1)	135,395
72	Conductive and sensorineural hearing loss	H90	43,238	35.7	(31.4)	1.4	(4.2)	40.2	(41.9)	13.2	(13.5)	5.3	(5.8)	98	21.8	(24.0)	36.1	(29.9)	22.8	(25.1)	19.1	(20.6)	198,734.8	(197,728.3)	148,477
73	Other hearing loss and other disorders of ear, not elsewhere classified	H910, H912, H913, H918, H930, H932, H933	8,306	36.0	(34.1)	1.0	(4.1)	40.9	(39.8)	13.5	(13.7)	4.9	(5.3)	93	20.2	(23.4)	36.1	(30.9)	24.5	(26.3)	19.1	(19.2)	202,992.9	(195,813.1)	195,115
74	Presbycusis (age-related hearing loss)	H911	80,659	44.0	(31.9)	0.0	(2.1)	32.5	(44.1)	9.8	(12.8)	3.6	(5.3)	19	23.1	(23.4)	47.4	(30.3)	17.9	(24.6)	11.5	(21.4)	178,694.7	(200,012.1)	130,395
75	Hearing loss, unspecified	H919	87,806	35.5	(30.5)	0.4	(4.3)	41.3	(42.4)	13.1	(13.7)	5.1	(5.9)	101	20.7	(24.0)	37.8	(29.6)	22.4	(25.5)	19.0	(20.6)	199,504.9	(197,443.4)	141,665
76	Tinnitus	H931	40,124	30.4	(25.5)	0.2	(3.5)	44.7	(43.9)	15.3	(16.5)	6.6	(8.3)	159	17.7	(25.0)	33.8	(28.0)	24.2	(24.0)	24.1	(22.7)	216,349.9	(202,839.9)	193,053
77	Other specified disorders of ear	H938	20,537	36.9	(29.8)	0.5	(4.1)	40.7	(44.6)	14.5	(13.8)	4.6	(5.7)	80	21.5	(23.5)	38.0	(29.0)	23.1	(27.0)	17.3	(20.1)	193,841.4	(195,362.3)	138,134
	**I–Diseases of the circulatory system**	**I05–I06; I10–28; I30–33; I36–141; I44–I52; I60–I88; I90–I94; I96–I99**	**1,254,427**	**35.4**	(**29.2)**	**0.2**	(**2.8)**	**43.2**	(**46.8)**	**12.9**	(**13.1)**	**4.8**	(**5.4)**	**103**	**19.8**	(**23.5)**	**35.5**	(**29.2)**	**23.8**	(**25.5)**	**20.6**	(**21.4)**	**206,330.1**	(**202,252.4)**	**185,543**
78	Aortic and mitral valve disease ^c^	I05, I06, I34, I35	30,123	43.4	(31.3)	0.2	(3.5)	36.8	(43.3)	10.0	(12.2)	4.4	(6.1)	23	23.3	(25.0)	42.9	(31.6)	18.7	(22.7)	15.0	(19.9)	190,094.4	(194,849.7)	195,987
79	Hypertensive diseases ^c^	I10–I15	1,060,046	36.9	(29.9)	0.1	(2.3)	42.8	(47.5)	12.4	(12.7)	4.4	(5.1)	81	20.3	(23.2)	36.8	(30.3)	23.2	(25.6)	19.5	(20.5)	203,263.9	(200,577.2)	180,504
80	Heart failure ^c^	I11.0, I13.0, I13.2, I42.0, I42.6, I42.7, I42.9, I50.0, I50.1, I50.9	37,540	42.4	(37.3)	0.1	(2.7)	39.6	(41.9)	8.7	(10.2)	3.9	(4.1)	32	22.1	(24.0)	44.3	(36.5)	19.3	(23.8)	14.1	(15.3)	189,258.2	(186,267.4)	187,532
80A	Ischaemic heart diseases	I20–I25	139,173	40.4	(36.8)	0.0	(2.4)	41.7	(43.1)	9.9	(10.5)	3.8	(3.8)	45	22.3	(25.8)	40.5	(32.8)	20.5	(23.7)	16.5	(17.3)	194,429.6	(188,574.6)	183,894
81	Angina pectoris	I20	78,476	39.4	(36.9)	0.0	(2.1)	42.5	(43.1)	10.6	(10.8)	4.0	(3.8)	52	22.3	(26.3)	39.0	(32.4)	21.0	(23.5)	17.4	(17.3)	197,173.6	(188,491.2)	194,370
82	Acute myocardial infarction and subsequent myocardial infarction	I21–I22	36,654	40.5	(39.1)	0.0	(2.3)	42.4	(42.3)	9.0	(9.6)	3.6	(3.1)	42	21.4	(26.0)	40.5	(32.5)	20.9	(25.1)	17.1	(16.1)	194,936.8	(186,024.7)	183,171
83	AMI complex/other	I23–I24	2,969	40.2	(35.4)	0.0	(1.3)	41.3	(45.2)	10.7	(11.3)	3.3	(3.0)	47	22.5	(25.5)	40.5	(32.5)	20.5	(23.8)	16.3	(17.9)	197,090.5	(192,599.4)	263,837
84	Chronic ischaemic heart disease	I25	84,592	42.0	(37.5)	0.0	(3.9)	41.1	(42.3)	8.8	(9.8)	3.6	(3.6)	34	23.2	(25.9)	42.6	(34.9)	19.3	(22.9)	14.7	(15.7)	189,164.3	(183,267.3)	177,003
85	Pulmonary heart disease and diseases of pulmonary circulation	I26–I28	15,352	39.9	(33.2)	0.5	(3.0)	40.3	(44.3)	11.0	(11.7)	4.2	(5.0)	48	21.9	(25.2)	41.0	(32.4)	20.9	(23.1)	16.1	(19.0)	192,577.9	(193,983.0)	152,267
86	Acute pericarditis	I30	5,563	28.5	(28.1)	1.3	(3.0)	48.5	(46.0)	12.0	(13.2)	6.7	(6.5)	174	19.6	(24.7)	27.6	(27.8)	25.3	(24.4)	27.0	(22.6)	226,031.7	(205,792.8)	197,524
87	Other forms of heart disease	I31–I43, except I34–I35 and I42	8,119	36.1	(33.1)	0.7	(2.5)	42.2	(43.4)	11.4	(12.3)	5.8	(5.6)	92	21.5	(24.7)	35.3	(31.1)	22.3	(23.6)	20.8	(20.2)	208,007.6	(198,854.3)	226,712
88	Atrioventricular and left bundle branch block	I44	14,604	40.4	(31.2)	0.1	(2.2)	37.4	(44.0)	9.7	(13.6)	4.6	(5.5)	46	22.1	(23.9)	42.7	(29.6)	20.1	(25.4)	14.9	(20.5)	192,043.0	(204,944.3)	181,989
89	Other conduction disorders	I45–46	11,823	35.4	(29.6)	0.9	(3.8)	42.5	(44.9)	10.9	(12.6)	5.6	(6.2)	104	20.7	(23.9)	37.1	(30.1)	22.4	(24.3)	19.5	(21.1)	202,901.2	(200,481.3)	189,274
90	Paroxysmal tachycardia	I47	39,510	32.5	(27.7)	0.8	(3.7)	42.9	(45.6)	14.7	(14.6)	6.1	(6.3)	138	20.5	(24.0)	34.3	(28.3)	23.1	(24.4)	21.9	(22.9)	209,359.8	(204,507.9)	209,887
91	Atrial fibrillation and flutter	I48	112,342	39.8	(29.8)	0.0	(2.1)	38.5	(45.3)	11.1	(14.0)	5.1	(6.2)	49	21.5	(23.3)	40.8	(28.6)	19.8	(25.0)	17.7	(22.7)	201,082.8	(207,206.3)	202,743
92	Other cardiac arrhythmias	I49	34,418	34.5	(27.0)	0.3	(2.9)	40.6	(45.6)	14.5	(15.7)	6.1	(6.5)	113	20.0	(23.0)	35.8	(27.5)	22.7	(25.0)	21.4	(24.2)	210,149.2	(211,236.2)	217,948
93	Complications and ill-defined descriptions of heart disease and other heart disorders in diseases classified elsewhere	I51–52	7,337	36.2	(31.4)	0.5	(2.8)	40.0	(41.8)	12.9	(13.4)	6.2	(7.3)	89	21.3	(24.3)	37.6	(31.6)	21.5	(22.6)	19.4	(21.1)	207,945.8	(207,738.2)	205,049
94	Stroke	I60, I61, I63–I64, Z501 (rehabilitation)	72,606	41.2	(35.6)	0.1	(2.5)	40.6	(45.0)	9.7	(10.5)	3.8	(3.8)	36	21.0	(23.3)	43.4	(35.0)	20.9	(25.1)	14.6	(16.3)	190,624.9	(189,249.6)	166,002
95	Cerebrovascular diseases	I62, I65–I68	17,308	37.9	(31.9)	0.3	(3.4)	41.8	(44.0)	11.8	(12.4)	5.2	(5.2)	64	21.0	(24.3)	39.6	(32.2)	21.2	(23.4)	17.9	(19.8)	200,913.0	(198,301.7)	202,349
96	Sequelae of cerebrovascular disease	I69	50,952	43.9	(39.9)	0.0	(2.6)	38.6	(42.0)	8.9	(9.4)	3.3	(3.1)	20	21.2	(21.4)	47.0	(40.0)	20.3	(25.2)	11.4	(13.1)	182,158.5	(182,804.7)	153,066
97	Atherosclerosis	I70	32,064	47.7	(41.0)	0.0	(2.0)	38.7	(42.8)	7.1	(8.3)	2.2	(3.0)	10	24.2	(25.3)	48.5	(39.0)	18.0	(21.9)	9.2	(13.6)	173,209.6	(179,064.2)	130,694
98	Aortic aneurysm and aortic dissection	I71	10,296	38.5	(28.9)	0.1	(3.4)	44.1	(44.2)	10.0	(14.1)	4.5	(6.9)	60	23.0	(24.1)	41.0	(30.1)	20.2	(22.5)	15.7	(23.1)	192,608.8	(202,594.4)	141,262
99	Diseases of arteries, arterioles and capillaries	I72, I74, I77–I79	11,830	31.8	(27.9)	0.5	(2.9)	43.6	(45.6)	14.9	(14.7)	6.4	(6.8)	145	19.4	(24.7)	33.9	(28.5)	23.8	(23.8)	22.7	(22.7)	213,898.0	(208,014.5)	297,631
100	Other peripheral vascular diseases	I73	28,508	44.4	(33.8)	0.3	(4.7)	41.8	(45.5)	7.9	(9.8)	2.6	(3.9)	17	23.9	(26.3)	45.4	(33.6)	19.0	(24.4)	11.5	(15.5)	178,817.1	(181,736.3)	150,027
101	Phlebitis, thrombosis of the portal vein and others	I80–I82	37,388	36.8	(32.7)	0.5	(3.3)	43.3	(45.2)	11.3	(11.2)	4.6	(4.9)	83	19.8	(24.5)	37.0	(31.6)	23.8	(24.5)	19.2	(19.1)	201,392.9	(195,004.0)	152,460
102	Varicose veins of lower extremities	I83	23,530	28.0	(26.0)	0.3	(2.4)	47.0	(50.1)	17.3	(13.9)	5.0	(4.6)	178	15.5	(21.3)	29.0	(26.8)	30.2	(28.3)	25.1	(23.3)	217,565.3	(208,063.1)	191,287
103	Haemorrhoids ^c^	I84	74,285	25.2	(26.0)	0.8	(2.4)	45.7	(46.7)	17.9	(15.4)	7.1	(6.2)	199	16.6	(22.3)	27.6	(26.9)	28.6	(25.7)	26.8	(24.6)	222,783.5	(211,880.4)	180,208
104	Oesophageal varices (chronic), varicose veins of other sites, other disorders of veins, non-specific lymphadenitis, other non-infective disorders of lymphatic vessels and lymph nodes and other and unspecified disorders of the circulatory system	I85–I99, except I89 and I95	15,194	32.7	(29.4)	1.9	(3.6)	44.0	(45.0)	12.6	(13.2)	5.5	(5.9)	132	22.9	(25.4)	34.0	(30.4)	23.3	(23.9)	19.6	(19.9)	198,980.6	(196,054.5)	189,873
	**J–Diseases of the respiratory system**	**J30.1; J40–J47; J60–J84; J95, J97–J99**	**1,210,598**	**27.8**	(**25.9)**	**2.2**	(**4.2)**	**44.3**	(**45.5)**	**15.9**	(**14.9)**	**7.3**	(**7.3)**	**180**	**20.6**	(**24.1)**	**28.9**	(**26.6)**	**25.6**	(**24.6)**	**24.5**	(**24.2)**	**213,419.8**	(**208,610.5)**	**191,819**
105	Respiratory allergy ^c^	J30, except J30.0	841,685	24.7	(23.4)	2.1	(4.0)	45.1	(46.2)	17.4	(16.1)	8.3	(8.2)	202	19.5	(23.3)	27.0	(25.2)	26.2	(24.8)	26.9	(26.3)	220,822.8	(215,395.1)	200,950
105A	Chronic lower respiratory diseases ^c^	J40–J43, J47	418,120	32.7	(29.1)	0.7	(3.5)	42.5	(44.6)	15.4	(14.2)	6.6	(6.7)	133	18.8	(24.6)	32.7	(28.8)	25.6	(24.2)	22.6	(22.0)	211,660.1	(203,729.6)	195,079
106	Bronchitis, not specified as acute or chronic, simple and mucopurulent chronic bronchitis and unspecified chronic bronchitis	J40–J42	12,790	50.4	(43.9)	0.1	(2.2)	35.2	(39.6)	8.0	(7.9)	2.2	(2.9)	4	24.8	(26.9)	48.6	(40.3)	18.1	(22.7)	8.3	(9.7)	171,943.8	(174,930.9)	151,491
107	Emphysema	J43	5,557	44.8	(40.4)	0.1	(2.9)	40.0	(40.6)	9.3	(10.0)	2.8	(3.1)	16	21.9	(24.8)	45.3	(39.8)	21.6	(24.1)	11.2	(11.2)	179,844.6	(175,200.0)	126,553
108	Chronic obstructive lung disease (COPD) ^c^	J44, J96, J13–J18	216,184	40.7	(32.8)	1.0	(3.2)	41.1	(45.1)	10.7	(12.0)	3.5	(4.6)	37	23.7	(26.1)	40.1	(32.6)	21.5	(23.9)	14.5	(17.1)	185,490.5	(188,049.7)	148,123
109	Asthma, status asthmaticus ^c^	J45–J46	361,129	32.0	(29.5)	3.2	(4.7)	42.0	(43.3)	14.5	(14.1)	6.0	(6.3)	142	23.7	(25.5)	31.6	(29.0)	23.8	(23.7)	20.5	(21.2)	199,808.7	(199,611.7)	196,814
110	Bronchiectasis	J47	4,362	31.1	(26.2)	0.5	(4.3)	41.6	(43.2)	17.9	(16.6)	6.3	(7.0)	153	20.2	(24.0)	34.1	(29.0)	24.4	(24.8)	20.9	(21.6)	208,653.1	(205,610.9)	188,879
111	Other diseases of the respiratory system	J60–J84; J95, J97–J99	21,993	39.2	(35.8)	0.6	(3.9)	41.9	(42.5)	10.5	(10.4)	3.7	(3.9)	53	21.5	(24.1)	40.0	(33.7)	22.7	(24.9)	15.5	(16.7)	191,907.5	(190,393.8)	149,165
	**K–Diseases of the digestive system**	**K25–K27; K40, K43, K50–52; K58–K59; K71–K77; K86–K87**	**329,337**	**34.6**	**(31.2)**	**1.2**	**(3.4)**	**42.9**	**(44.7)**	**12.8**	**(12.5)**	**4.7**	**(4.8)**	**112**	**21.3**	**(25.2)**	**35.7**	**(31.2)**	**24.0**	**(24.3)**	**18.7**	**(18.8)**	**198,643.1**	**(193,619.0)**	**163,696**
112	Ulcers ^c^	K25–K27	157,379	39.0	(35.0)	1.0	(3.6)	40.4	(42.4)	11.0	(10.5)	3.7	(3.8)	57	22.6	(26.6)	39.3	(33.7)	22.4	(23.4)	15.3	(15.6)	189,749.3	(184,915.1)	156,587
113	Inguinal hernia	K40	25,032	27.6	(26.1)	0.6	(3.9)	49.0	(46.3)	12.3	(14.8)	7.5	(6.0)	183	16.1	(22.9)	27.6	(25.5)	26.0	(27.2)	30.1	(24.1)	236,936.1	(209,391.7)	221,825
114	Ventral hernia	K43	7,941	37.3	(35.5)	0.4	(3.4)	43.2	(43.5)	12.3	(11.0)	3.9	(3.8)	74	19.5	(23.4)	37.5	(34.1)	25.0	(25.3)	17.8	(16.8)	197,931.9	(190,849.0)	180,137
115	Crohn’s disease	K50	18,913	26.7	(27.9)	2.0	(3.3)	47.7	(47.5)	15.4	(13.6)	6.4	(5.8)	187	19.7	(23.9)	29.2	(29.1)	27.4	(24.7)	23.2	(21.8)	209,532.3	(201,829.3)	162,426
116	Ulcerative colitis	K51	29,538	25.4	(24.7)	1.2	(3.1)	47.8	(48.6)	16.6	(15.2)	7.2	(6.7)	197	17.8	(23.1)	27.6	(26.3)	27.4	(25.4)	26.8	(24.8)	219,262.5	(208,635.1)	160,979
117	Other non-infective gastroenteritis and colitis	K52	20,844	37.1	(31.0)	1.4	(3.5)	41.6	(45.8)	12.5	(12.4)	3.9	(4.6)	76	23.6	(24.9)	38.8	(32.5)	22.4	(24.2)	15.0	(18.0)	187,639.2	(193,366.5)	141,202
118	Irritable bowel syndrome (IBS)	K58	37,593	28.0	(28.0)	1.2	(2.5)	46.1	(47.4)	17.3	(14.8)	5.4	(5.4)	179	21.2	(24.5)	31.1	(28.9)	26.7	(24.6)	20.9	(21.7)	203,029.9	(202,383.4)	150,613
119	Other functional intestinal disorders	K59	51,933	37.6	(33.2)	1.5	(3.7)	39.7	(43.4)	12.2	(12.0)	4.1	(4.4)	68	22.8	(24.7)	39.0	(33.3)	23.0	(24.3)	15.0	(17.3)	188,382.5	(190,772.4)	158,264
120	Diseases of liver, biliary tract and pancreas	K71–K77; K86–K87	26,956	36.2	(34.7)	0.6	(3.8)	44.3	(43.2)	11.6	(11.0)	4.1	(3.9)	90	21.9	(27.8)	39.6	(35.1)	22.5	(22.0)	15.6	(14.5)	189,259.5	(178,925.8)	150,370
	**L–Diseases of the skin and subcutaneous tissue**	**L40**	65,469	**28.7**	(**26.4)**	**0.8**	(**3.4)**	**45.9**	(**46.6)**	**15.2**	(**14.5)**	**7.0**	(**6.9)**	**171**	**17.2**	(**22.7)**	**29.8**	(**26.8)**	**26.1**	(**25.5)**	**26.6**	(**24.6)**	**223,937.7**	(**211,259.0)**	**196,774**
121	Psoriasis ^c^	L40	65,469	28.7	(26.4)	0.8	(3.4)	45.9	(46.6)	15.2	(14.5)	7.0	(6.9)	172	17.2	(22.7)	29.8	(26.8)	26.1	(25.5)	26.6	(24.6)	223,937.7	(211,259.0)	196,774
	**M–Diseases of the musculoskeletal system and connective tissue**	**M01–M25; M30–M36; M40–M54; M60.1–M99**	**1,032,808**	**32.2**	(**27.7)**	**1.4**	(**4.3)**	**44.5**	(**46.7)**	**14.1**	(**13.6)**	**5.0**	(**5.4)**	**140**	**20.7**	(**24.2)**	**32.6**	(**28.0)**	**24.7**	(**25.2)**	**21.8**	(**22.2)**	**207,898.7**	(**203,403.2)**	**192,082**
122	Infectious arthropathies	M01–M03	9,402	24.6	(25.1)	1.0	(2.7)	48.1	(48.2)	16.5	(15.2)	7.7	(6.7)	203	17.5	(23.5)	26.2	(26.2)	26.2	(24.0)	29.8	(26.0)	228,329.5	(212,370.0)	204,720
122A	Inflammatory polyarthropathies and ankylosing spondylitis ^c^	M05–M14, M45	165,944	33.5	(28.2)	0.5	(3.7)	44.6	(45.7)	12.8	(13.6)	5.8	(6.7)	122	19.2	(24.2)	34.5	(28.7)	23.9	(24.6)	22.1	(22.1)	212,831.2	(203,828.4)	205,567
123	Rheumatoid arthritis ^c^	M05, M06, M07.1, M07.2, M07.3, M08, M09	77,345	31.3	(26.0)	0.8	(4.3)	42.5	(44.4)	16.0	(15.1)	7.4	(8.5)	152	19.3	(24.3)	32.1	(26.9)	24.5	(23.7)	23.8	(24.7)	215,782.6	(211,142.4)	186,887
124	Inflammatory polyarthropathies–except rheumatoid arthritis ^c^	M074–M079, M10–M14, M45	115,945	34.4	(29.2)	0.2	(2.6)	45.8	(47.4)	11.7	(13.2)	4.9	(5.5)	114	19.1	(23.7)	35.7	(30.1)	24.1	(25.4)	21.0	(20.4)	209,620.0	(199,544.5)	200,184
125	Polyarthrosis [arthrosis]	M15	16,935	39.5	(29.0)	0.0	(2.6)	40.1	(50.2)	13.7	(12.5)	3.2	(4.1)	51	24.8	(25.1)	40.8	(31.2)	20.5	(24.0)	13.8	(19.6)	185,372.7	(197,730.4)	192,801
126	Coxarthrosis [arthrosis of hip]	M16	104,115	42.5	(30.6)	0.1	(2.9)	38.1	(46.1)	11.7	(13.2)	3.7	(4.7)	30	22.7	(22.8)	40.7	(30.6)	20.4	(25.7)	16.0	(20.5)	193,738.4	(202,550.8)	183,754
127	Gonarthrosis [arthrosis of knee]	M17	178,811	36.3	(29.0)	0.0	(2.4)	44.0	(49.5)	13.0	(13.1)	3.6	(4.0)	87	20.4	(22.1)	35.9	(29.3)	23.2	(26.6)	20.3	(21.7)	205,368.5	(205,249.8)	191,733
128	Arthrosis of first carpometacarpal joint and other arthrosis	M18–M19	91,101	35.3	(29.4)	0.1	(2.3)	45.1	(50.1)	13.2	(12.5)	3.5	(3.6)	106	20.1	(23.4)	36.0	(30.5)	24.4	(26.2)	19.4	(19.4)	202,099.8	(196,701.1)	165,932
129	Acquired deformities of fingers and toes	M20	55,730	29.3	(26.2)	0.9	(3.7)	45.7	(48.6)	17.5	(14.4)	4.7	(5.3)	165	19.4	(22.5)	31.1	(26.6)	27.0	(26.9)	22.4	(23.7)	207,987.9	(207,765.6)	165,023
130	Other acquired deformities of limbs	M21	20,584	31.9	(28.7)	2.1	(4.1)	44.5	(46.4)	15.4	(14.1)	4.2	(4.7)	143	21.7	(23.9)	32.4	(29.1)	26.1	(26.2)	19.5	(20.5)	199,959.6	(199,430.5)	148,960
131	Disorders of patella (kneecap)	M22	38,999	22.6	(26.6)	6.3	(3.7)	49.5	(48.3)	14.7	(14.4)	5.8	(5.5)	209	27.8	(23.4)	24.5	(27.6)	26.5	(25.5)	20.8	(23.2)	194,315.3	(206,749.7)	164,676
132	Internal derangement of knee	M230, M231, M233, M235, M236, M238	9,192	19.3	(23.0)	6.3	(4.4)	49.0	(47.0)	15.9	(16.5)	7.4	(7.1)	215	27.8	(24.7)	19.5	(22.6)	25.4	(25.6)	26.9	(26.6)	214,648.5	(222,447.7)	296,851
133	Derangement of meniscus due to old tear or injury	M232	36,374	22.2	(23.8)	3.2	(4.6)	50.3	(48.3)	15.9	(15.2)	6.8	(6.2)	211	18.3	(23.7)	21.4	(23.3)	27.6	(25.9)	32.3	(26.8)	239,195.3	(219,540.6)	247,263
134	Internal derangement of knee, unspecified	M239	28,206	23.0	(24.9)	6.4	(5.6)	48.7	(47.4)	14.6	(14.6)	5.5	(5.4)	207	23.4	(23.9)	22.0	(24.4)	26.5	(25.6)	27.7	(25.7)	217,585.8	(213,151.9)	200,094
135	Other specific joint derangements	M24, except M240–M241	5,923	22.4	(24.7)	7.4	(4.5)	47.8	(46.9)	13.2	(14.5)	6.8	(6.6)	210	31.2	(24.6)	21.1	(24.1)	22.7	(23.9)	24.3	(26.9)	202,470.0	(219,362.3)	186,298
136	Other joint disorders, not elsewhere classified	M25	12,043	27.8	(28.3)	3.5	(4.9)	46.7	(46.5)	14.7	(12.9)	4.9	(5.0)	181	21.9	(25.6)	29.4	(28.5)	27.0	(24.9)	21.4	(20.7)	201,737.0	(196,518.1)	133,897
137	Systemic connective tissue disorders	M30–M36, except M32, M34	42,631	36.6	(29.4)	0.7	(3.7)	39.6	(45.0)	15.1	(14.0)	4.6	(5.9)	84	22.3	(25.1)	38.0	(30.8)	23.2	(23.6)	16.3	(20.2)	191,438.0	(198,438.7)	151,635
138	Systemic lupus erythematosus	M32	3,376	30.8	(31.2)	0.9	(5.1)	41.5	(40.9)	19.2	(14.5)	5.4	(6.0)	155	19.7	(26.5)	33.3	(31.5)	27.6	(23.0)	18.9	(18.0)	200,376.5	(192,127.0)	145,294
139	Dermatopolymyositis	M33	1,137	33.5	(29.4)	0.7	(2.2)	43.1	(43.7)	13.8	(14.4)	6.3	(7.6)	123	20.2	(23.5)	32.5	(27.4)	26.4	(27.4)	20.9	(21.2)	207,965.0	(203,705.7)	167,493
140	Systemic sclerosis	M34	1,675	36.5	(31.7)	0.7	(5.6)	42.5	(45.1)	15.4	(11.6)	3.9	(5.0)	85	21.8	(25.1)	34.6	(30.0)	26.1	(25.2)	17.4	(19.0)	194,555.7	(190,308.8)	133,339
141	Kyphosis, lordosis	M40	4,160	31.9	(32.2)	2.1	(4.5)	41.1	(40.6)	15.6	(14.1)	7.0	(6.4)	144	20.8	(26.2)	32.2	(31.3)	25.1	(23.3)	21.4	(18.6)	202,823.9	(189,789.5)	138,776
142	Scoliosis	M41	17,686	29.9	(31.2)	8.3	(4.9)	39.7	(41.1)	14.1	(13.8)	6.0	(6.6)	163	32.5	(25.2)	29.8	(30.9)	22.8	(24.9)	14.5	(18.6)	172,617.4	(191,552.1)	128,957
143	Spinal osteochondrosis	M42	8,034	32.6	(32.4)	1.5	(4.0)	46.4	(44.4)	12.4	(12.9)	5.3	(4.6)	137	19.4	(25.9)	33.0	(32.4)	26.7	(24.7)	20.6	(16.7)	204,371.5	(186,887.1)	146,715
144	Other deforming dorsopathies	M43	23,756	34.3	(29.9)	0.5	(3.7)	44.3	(46.9)	13.9	(12.8)	4.3	(4.6)	116	19.9	(25.1)	35.0	(30.7)	24.7	(24.4)	20.3	(19.4)	207,124.2	(197,278.0)	260,719
145	Other inflammatory spondylopathies	M46	7,086	30.3	(29.5)	0.8	(3.0)	46.4	(46.8)	14.5	(13.0)	5.8	(5.5)	160	17.0	(23.6)	33.3	(31.4)	27.5	(25.1)	21.8	(19.5)	207,618.2	(194,660.3)	137,350
146	Spondylosis	M47	61,999	37.7	(31.7)	0.0	(2.5)	44.0	(48.9)	12.0	(11.1)	3.4	(3.4)	66	20.1	(23.4)	38.2	(33.2)	23.3	(25.2)	18.1	(17.9)	201,047.4	(194,885.6)	204,090
147	Other spondylopathies and spondylopathies in diseases classified elsewhere	M48, M49	50,805	39.2	(32.6)	0.0	(2.3)	41.9	(47.9)	11.8	(11.0)	3.9	(3.6)	54	22.5	(23.7)	40.2	(34.4)	20.9	(24.3)	16.2	(17.4)	194,168.3	(191,906.1)	175,865
148	Cervical disc disorders	M50	11,476	26.6	(28.8)	0.1	(2.1)	51.2	(49.6)	14.8	(12.8)	5.3	(4.7)	190	12.7	(21.9)	27.7	(29.7)	29.4	(24.8)	30.0	(23.2)	239,345.7	(210,468.8)	227,615
149	Other intervertebral disc disorders	M51	40,161	29.1	(29.7)	0.5	(2.8)	49.1	(48.2)	13.9	(12.6)	4.8	(4.2)	167	17.0	(23.9)	30.7	(30.6)	27.2	(24.7)	24.9	(20.5)	219,345.8	(200,481.9)	222,707
150	Other dorsopathies, not elsewhere classified	M53	7,246	28.4	(29.0)	2.2	(5.4)	48.8	(47.1)	13.6	(11.7)	4.6	(4.4)	176	17.9	(24.3)	29.4	(28.5)	27.8	(24.6)	24.6	(22.1)	223,180.6	(208,983.7)	272,920
151	Dorsalgia	M54	40,780	31.5	(31.2)	1.8	(4.4)	46.2	(45.6)	12.6	(11.4)	4.5	(4.2)	149	19.6	(25.0)	32.9	(31.4)	25.8	(23.9)	21.4	(19.4)	210,603.8	(199,156.2)	243,249
152	Soft tissue disorders	M60–M63, except M60.0	13,422	30.6	(32.0)	3.5	(4.1)	44.2	(44.5)	13.9	(11.7)	4.5	(4.4)	157	23.2	(25.5)	32.1	(31.6)	26.4	(24.4)	17.8	(18.0)	192,898.9	(192,128.4)	140,081
153	Synovitis and tenosynovitis	M65	19,104	28.1	(26.7)	3.1	(4.6)	46.9	(47.8)	14.9	(13.5)	5.1	(5.3)	177	20.8	(23.9)	27.8	(26.3)	27.7	(26.4)	23.4	(23.0)	212,294.4	(208,001.6)	207,866
154	Disorders of synovium and tendon	M66–68	19,669	23.7	(25.0)	5.8	(5.1)	48.5	(48.3)	14.7	(14.1)	5.4	(5.4)	204	23.5	(23.5)	24.2	(25.3)	27.4	(26.6)	24.5	(24.2)	208,141.8	(208,416.2)	165,410
155	Soft tissue disorders related to use, overuse and pressure	M70	11,090	29.5	(26.3)	3.0	(5.5)	46.7	(47.7)	14.6	(14.1)	4.6	(5.0)	164	20.7	(23.7)	29.1	(26.5)	26.7	(26.6)	23.3	(22.9)	209,489.5	(204,844.6)	176,380
156	Fibroblastic disorders	M72	43,600	30.1	(25.5)	0.2	(3.6)	47.4	(48.6)	14.4	(14.6)	5.7	(5.9)	161	16.9	(21.8)	31.5	(26.3)	25.6	(26.8)	25.9	(24.7)	225,565.9	(211,841.5)	225,351
157	Shoulder lesions	M75	58,112	28.5	(28.1)	1.0	(3.8)	50.4	(49.7)	13.9	(12.6)	4.2	(3.8)	175	16.2	(24.2)	28.5	(28.3)	28.6	(25.9)	26.5	(21.4)	223,017.7	(201,283.0)	204,824
158	Enthesopathies of lower limb, excluding foot	M76	11,223	17.8	(20.0)	5.3	(5.4)	47.2	(46.3)	18.9	(18.0)	9.5	(8.8)	216	21.4	(23.7)	18.7	(21.1)	25.4	(24.1)	34.3	(30.9)	238,662.6	(229,940.0)	234,549
159	Other enthesopathies	M77	10,500	24.8	(25.2)	1.7	(4.3)	50.5	(48.8)	15.1	(13.8)	6.1	(5.9)	200	14.9	(23.3)	25.4	(25.5)	29.9	(25.8)	29.6	(25.0)	232,334.6	(213,503.2)	226,500
160	Rheumatism, unspecified	M790	6,852	40.7	(37.0)	0.2	(1.5)	41.8	(45.3)	12.4	(10.4)	2.7	(3.4)	38	22.9	(29.3)	42.3	(35.6)	23.3	(20.6)	11.3	(14.3)	179,788.1	(182,066.0)	136,756
161	Myalgia	M791	10,168	36.0	(34.1)	0.6	(3.0)	44.4	(46.0)	12.2	(10.5)	3.5	(3.4)	94	19.9	(25.6)	36.3	(33.2)	25.6	(24.1)	17.9	(16.8)	196,567.6	(188,210.8)	149,246
162	Other soft tissue disorders, not elsewhere classified	M792– M794; M798–M799	7,939	29.0	(28.5)	0.9	(2.7)	45.7	(47.1)	15.5	(13.1)	6.5	(6.3)	169	18.9	(24.7)	32.3	(30.5)	26.8	(24.1)	21.6	(20.4)	206,636.9	(197,940.7)	135,993
163	Other soft tissue disorders, not elsewhere classified: pain in limb	M796	22,201	31.4	(29.8)	3.9	(5.4)	44.6	(45.3)	13.2	(12.6)	4.1	(4.2)	151	22.7	(25.1)	31.0	(29.5)	25.2	(24.7)	20.7	(20.4)	200,364.0	(196,775.0)	156,863
164	Fibromyalgia	M797	3,399	37.4	(36.6)	0.4	(1.3)	44.0	(46.3)	14.4	(9.4)	1.9	(3.5)	72	21.3	(30.6)	46.2	(38.9)	24.3	(21.7)	7.9	(8.6)	170,222.3	(159,950.5)	67,717
165	Osteoporosis ^c^	M80–M81	158,813	43.3	(33.1)	0.0	(3.1)	35.6	(41.1)	13.7	(14.0)	3.2	(5.9)	25	24.9	(19.3)	42.4	(35.0)	19.7	(26.1)	12.9	(19.2)	181,115.2	(200,729.1)	148,814
166	Osteoporosis in diseases classified elsewhere	M82	1,007	39.8	(36.3)	0.8	(5.4)	40.1	(40.0)	12.8	(11.3)	4.2	(4.8)	50	22.2	(20.7)	40.2	(35.2)	23.2	(25.0)	14.5	(19.1)	188,257.4	(219,018.0)	155,948
167	Adult osteomalacia and other disorders of bone density and structure	M83, M85, except M833	43,271	34.2	(27.0)	0.3	(3.3)	41.5	(47.2)	17.5	(14.9)	4.5	(5.7)	117	21.5	(23.5)	35.4	(29.3)	24.4	(25.6)	18.5	(21.2)	197,049.2	(199,957.0)	169,997
168	Disorders of continuity of bone	M84	1,865	34.7	(33.1)	4.7	(4.4)	42.3	(43.2)	11.7	(12.6)	3.8	(3.8)	110	29.6	(28.6)	32.2	(30.9)	19.1	(20.2)	19.2	(20.0)	194,711.8	(196,186.7)	286,038
169	Other osteopathies	M86–M90	24,251	37.4	(31.8)	0.8	(4.0)	42.4	(45.4)	13.1	(12.6)	3.7	(4.1)	73	22.2	(25.0)	37.4	(31.9)	23.2	(24.3)	16.9	(18.3)	193,819.5	(192,030.7)	183,059
170	Other disorders of the musculoskeletal system and connective tissue	M95–M99	30,038	32.2	(31.1)	1.4	(3.1)	46.0	(46.3)	13.0	(12.7)	4.7	(4.4)	141	21.0	(25.7)	32.6	(31.0)	25.4	(24.1)	20.7	(18.8)	202,414.7	(192,249.9)	176,603
	**N–Diseases of the genitourinary system**	**N18**	**20,162**	**42.8**	**(36.2)**	**0.4**	**(3.7)**	**39.5**	**(42.3)**	**8.8**	**(10.4)**	**3.5**	**(4.0)**	**28**	**22.6**	**(25.0)**	**44.1**	**(35.9)**	**20.1**	**(24.1)**	**13.1**	**(14.8)**	**185,507.6**	**(183,509.0)**	**208,833**
171	Chronic renal failure (CRF) ^c^	N18	20,162	42.8	(36.2)	0.4	(3.7)	39.5	(42.3)	8.8	(10.4)	3.5	(4.0)	29	22.6	(25.0)	44.1	(35.9)	20.1	(24.1)	13.1	(14.8)	185,507.6	(183,509.0)	208,833
	**Q–Congenital malformations, deformations and chromosomal abnormalities**	**Q00–Q56; Q60–Q99**	124,898	**26.4**	(**28.8)**	**3.7**	(**4.2)**	**43.6**	(**43.4)**	**16.4**	(**14.5)**	**7.7**	(**6.7)**	**192**	**21.1**	(**23.2)**	**26.6**	(**27.9)**	**28.2**	(**25.8)**	**23.7**	(**22.7)**	**209,140.1**	(**205,712.7)**	**176,751**
172	Congenital malformations: of the nervous, circulatory and respiratory systems, cleft palate and cleft lip, urinary tract, bones and muscles, other and chromosomal abnormalities not elsewhere classified	Q00–Q07; Q20–Q37; Q60–Q99	85,534	27.3	(29.8)	4.0	(4.1)	42.5	(42.7)	16.4	(14.3)	7.4	(6.6)	184	21.4	(22.9)	27.5	(28.8)	28.3	(26.0)	22.4	(21.9)	206,001.2	(205,281.3)	179,413
173	Congenital malformations of eye, ear, face and neck	Q10–Q18	19,689	23.2	(27.2)	4.7	(4.5)	44.7	(44.4)	17.4	(15.0)	8.2	(7.0)	205	22.6	(23.5)	23.9	(25.9)	28.9	(26.4)	24.1	(23.8)	207,917.6	(207,897.6)	170,832
174	Other congenital malformations of the digestive system	Q38–Q45	6,481	34.8	(32.1)	1.6	(4.0)	41.6	(42.7)	13.2	(12.7)	5.7	(6.0)	109	22.3	(25.2)	33.9	(29.5)	24.3	(24.7)	19.3	(20.1)	197,795.3	(195,288.3)	153,863
175	Congenital malformations of the sexual organs	Q50–Q56	16,192	22.9	(25.7)	1.7	(3.2)	47.8	(45.5)	16.6	(16.3)	9.3	(7.4)	208	16.7	(23.4)	23.0	(25.1)	28.6	(25.7)	31.3	(25.5)	230,793.1	(209,796.9)	169,051
	**F–Mental and behavioural disorders**	**F00–99**	**683,194**	**37.2**	**(37.1)**	**2.9**	**(4.1)**	**39.5**	**(39.7)**	**12.6**	**(11.6)**	**4.2**	**(4.2)**	**75**	**25.4**	**(28.3)**	**38.4**	**(36.6)**	**23.3**	**(22.3)**	**12.5**	**(12.4)**	**177,310.4**	**(173,513.9)**	**130,361**
176	Dementia ^c^	F00, G30, F01, F02.0, F03.9, G31.8B, G31.8E, G31.9, G31.0B	36,803	49.8	(44.7)	0.0	(1.0)	29.3	(30.2)	7.6	(10.2)	2.9	(6.9)	5	20.8	(13.5)	53.4	(39.2)	18.3	(35.4)	7.5	(11.9)	172,988.4	(194,011.8)	119,118
177	Organic, including symptomatic, mental disorders	F04–F09	26,430	48.4	(51.2)	0.4	(2.3)	32.1	(30.4)	8.4	(7.9)	3.4	(3.1)	8	18.5	(23.1)	49.6	(45.0)	23.0	(23.4)	8.7	(8.0)	177,330.1	(169,182.0)	135,271
178	Mental and behavioural disorders due to use of alcohol	F10	59,143	41.7	(42.7)	2.2	(3.3)	41.1	(39.0)	8.3	(8.7)	2.9	(2.5)	35	28.5	(32.1)	45.4	(43.4)	17.8	(17.3)	7.8	(6.8)	163,427.6	(157,365.0)	103,020
179	Mental and behavioural disorders due to psychoactive substance use	F11–F19	53,669	48.9	(48.4)	2.6	(2.5)	35.9	(36.1)	6.8	(7.3)	1.8	(1.9)	7	32.1	(31.0)	40.7	(41.6)	18.9	(19.0)	7.8	(7.9)	159,592.7	(161,082.1)	94,790
180	Schizophrenia ^c^	F20	29,422	54.1	(55.1)	1.1	(1.6)	31.8	(30.5)	6.1	(6.3)	2.6	(2.5)	3	21.5	(25.1)	53.0	(51.5)	22.9	(20.9)	2.0	(2.1)	158,208.7	(154,198.2)	52,774
181	Schizotypal and delusional disorders	F21–F29	39,694	47.8	(48.5)	1.6	(2.5)	33.4	(32.5)	8.6	(8.3)	3.8	(3.7)	9	25.6	(28.5)	48.6	(47.0)	20.3	(19.0)	4.9	(4.8)	159,138.4	(155,383.1)	72,406
182	Bipolar affective disorder ^c^	F30–F31	22,669	35.8	(37.3)	1.0	(2.6)	39.0	(38.2)	15.6	(13.4)	5.8	(5.4)	96	21.7	(28.2)	44.6	(41.6)	23.3	(20.6)	9.9	(9.0)	176,371.8	(166,726.1)	120,692
183	Depression ^c^	F32, F33, F34.1, F06.32	454,933	35.5	(35.3)	1.5	(3.1)	41.6	(42.3)	13.9	(12.2)	4.4	(4.3)	102	23.0	(28.0)	39.0	(35.8)	24.3	(22.6)	13.5	(13.2)	182,965.5	(176,585.6)	135,764
184	Mood (affective) disorders	F340, F348–F349, F38–F39	6,887	40.5	(41.3)	1.5	(3.3)	37.2	(36.9)	13.6	(11.8)	3.9	(3.7)	43	23.8	(29.6)	44.6	(41.6)	22.9	(20.6)	8.3	(7.9)	171,327.1	(163,524.5)	107,866
185	Phobic anxiety disorders	F40	14,324	40.5	(41.1)	3.0	(2.7)	38.1	(39.5)	12.3	(11.0)	4.4	(3.9)	44	35.2	(33.5)	36.9	(39.6)	19.1	(17.8)	8.5	(8.8)	156,603.7	(158,543.5)	85,095
186	Other anxiety disorders	F41	38,079	38.3	(40.1)	2.2	(2.8)	38.4	(38.2)	13.8	(11.9)	4.4	(4.1)	63	28.0	(30.7)	40.6	(40.0)	21.4	(19.3)	9.6	(9.6)	167,030.9	(163,898.3)	92,980
187	Obsessive compulsive disorder (OCD) ^c^	F42	10,062	32.7	(33.8)	7.6	(4.2)	39.0	(39.6)	13.2	(13.6)	5.7	(6.1)	134	37.0	(27.5)	34.3	(38.8)	19.5	(21.9)	8.7	(11.5)	154,597.8	(174,007.1)	143,639
188	Post-traumatic stress disorder	F431	16,055	35.7	(37.6)	1.0	(2.1)	39.2	(35.8)	11.6	(10.7)	3.7	(3.4)	99	26.6	(32.2)	45.3	(41.9)	19.5	(17.3)	8.0	(7.8)	163,647.5	(156,932.9)	74,076
189	Reactions to severe stress and adjustment disorders	F432–F439	61,701	37.6	(38.2)	5.1	(3.6)	38.8	(39.4)	11.9	(11.9)	3.4	(3.6)	69	33.0	(29.4)	34.9	(37.5)	21.1	(21.2)	10.4	(11.3)	162,759.8	(168,874.8)	118,942
190	Dissociative (conversion) disorders, somatoform disorders and other neurotic disorders	F44, F45, F48	21,420	36.9	(38.4)	1.5	(3.3)	41.0	(40.2)	13.2	(11.1)	4.6	(4.1)	82	22.5	(28.3)	40.8	(38.9)	24.1	(21.0)	12.3	(11.4)	180,684.6	(172,333.9)	154,154
191	Eating disorders	F50	7,751	26.3	(35.4)	12.2	(4.6)	41.6	(36.6)	14.0	(14.3)	4.4	(6.7)	193	51.2	(29.2)	28.4	(40.1)	14.5	(18.8)	5.4	(11.0)	130,334.0	(168,077.4)	82,927
192	Behavioural syndromes associated with physiological disturbances and physical factors	F51–F59	6,163	22.0	(26.3)	1.6	(2.2)	42.5	(41.4)	20.6	(18.3)	10.7	(9.0)	212	21.0	(24.4)	27.5	(29.3)	26.5	(23.2)	24.4	(22.6)	210,461.7	(203,097.3)	175,784
193	Emotionally unstable personality disorder	F603	21,848	49.1	(49.4)	2.5	(2.2)	33.1	(32.1)	10.3	(10.6)	2.7	(2.8)	6	29.9	(27.9)	44.7	(46.8)	20.3	(20.4)	4.6	(4.5)	155,513.9	(156,772.4)	66,340
194	Specific personality disorders	F602, F604–F609	50,415	43.4	(45.5)	1.7	(2.2)	37.0	(35.7)	11.7	(10.5)	3.7	(3.4)	24	27.6	(30.0)	44.5	(44.6)	20.8	(18.7)	6.7	(6.3)	161,422.7	(157,003.0)	80,112
195	Disorders of adult personality and behaviour	F61–F69	17,533	43.2	(45.0)	1.7	(2.4)	37.1	(35.4)	11.1	(10.2)	3.8	(3.5)	27	27.8	(30.5)	46.0	(45.6)	19.2	(17.3)	6.3	(6.0)	159,854.0	(155,763.3)	76,056
196	Mental retardation	F70–F79	13,822	80.3	(81.9)	4.0	(2.8)	4.0	(4.1)	0.6	(0.6)	0.3	(0.4)	1	16.5	(15.3)	49.7	(51.2)	32.4	(32.1)	1.1	(1.2)	164,212.1	(166,397.7)	55,655
197	Disorders of psychological development	F80–F89	9,911	59.9	(65.0)	17.2	(5.2)	14.1	(15.1)	1.9	(3.6)	1.2	(2.2)	2	47.2	(22.7)	35.9	(46.9)	14.6	(27.3)	1.5	(2.8)	133,171.3	(160,337.1)	110,011
198	Hyperkinetic disorders (ADHD) ^c^	F90	42,908	45.6	(38.7)	15.6	(5.6)	27.5	(35.1)	6.3	(12.3)	2.4	(5.6)	14	49.5	(29.9)	28.6	(34.6)	14.4	(21.4)	6.7	(13.7)	121,815.9	(176,405.0)	74,858
199	Behavioural and emotional disorders with onset usually occurring in childhood and adolescence	F91–F99	39,602	45.4	(44.2)	5.8	(4.2)	33.3	(34.7)	8.8	(9.8)	3.3	(3.6)	15	33.6	(29.2)	40.4	(42.9)	18.4	(19.5)	6.9	(7.8)	154,155.5	161,308.2)	108,201
	0 chronic conditions		1565,998	16.7	(21.3)	11.3	(5.9)	42.7	(42.9)	13.5	(15.2)	8.2	(8.3)		31.3	(24.1)	15.8	(20.2)	22.6	(24.4)	25.7	28.0)	200,442.7	(215,232)	204,220
	1 chronic condition		906,365	20.6	(23.1)	4.7	(4.8)	47.1	(45.9)	16.6	(15.7)	8.6	(7.9)		22.0	(23.7)	20.3	(22.3)	27.1	(25.6)	29.8	27.7)	223,117.6	(216,765)	201,832
	2 chronic conditions		601,767	25.3	(28.8)	2.4	(3.5)	46.5	(45.1)	16.2	(14.2)	7.3	(6.1)		19.6	(24.5)	24.9	(27.2)	27.4	(25.1)	27.7	22.8)	222,043.5	(210,641)	204,691
	3 chronic conditions		435,614	29.1	(32.2)	1.3	(3.2)	45.2	(43.6)	15.5	(13.2)	6.3	(5.3)		19.3	(25.2)	29.1	(30.1)	26.6	(24.4)	24.8	(20.0)	215,858.3	(204,289)	195,793
	4 chronic conditions		306,882	32.8	(35.8)	0.8	(2.7)	43.7	(42.5)	14.5	(11.9)	5.4	(4.4)		19.9	(26.0)	33.2	(33.1)	25.3	(23.4)	21.4	(17.2)	207,774.6	(197,469)	179,535
	5 chronic conditions		218,183	36.1	(35.8)	0.5	(2.7)	42.5	(42.5)	13.1	(11.9)	4.5	(4.4)		20.9	(26.0)	36.9	(33.1)	23.8	(23.4)	18.2	(17.2)	199,222.1	(189,897)	190,270
	6 chronic conditions		155,685	39.0	(39.7)	0.3	(2.3)	40.9	(40.3)	12.2	(11.1)	3.9	(3.6)		21.8	(26.3)	39.8	(36.0)	22.5	(22.5)	15.8	(15.0)	192,274.6	(183,651)	157,125
	7 chronic conditions		109,688	41.2	(42.6)	0.2	(2.2)	39.9	(39.0)	11.3	(10.0)	3.6	(3.2)		22.5	(27.1)	42.2	(38.1)	21.3	(21.5)	13.9	(13.1)	187,010,8	(179,111)	173,999
	8 chronic conditions		77,563	43.1	(45.1)	0.2	(1.8)	38.9	(38.0)	10.7	(9.2)	3.2	(2.8)		23.3	(27.3)	44.2	(40.6)	20.5	(21.0)	11.9	(11.0)	181,557.9	(173,288)	148,095
	9 chronic conditions		54,429	44.4	(48.2)	0.1	(1.3)	37.9	(36.3)	10.0	(8.5)	3.2	(2.7)		23.4	(26.6)	45.9	(43.6)	19.6	(19.8)	11.0	(9.8)	179,234.3	(171,470)	138,097
	10 or mor chronic conditions		123,265	47.3	(51.8)	0.1	(0.8)	36.4	(33.9)	9.2	(7.6)	2.6	(2.0)		23.9	(26.1)	48.8	(47.3)	18.7	(19.0)	8.6	(7.3)	172,596.9	(166,083)	124,612
	**Having one or more chronic conditions**		**2,989,441**	**29.0**	(**27.5)**	**2.3**	(**4.3)**	**44.6**	(**45.0)**	**14.9**	(**14.2)**	**2.8**	(**6.5)**		**21.0**	(**24.6)**	**29.1**	(**26.8)**	**25.5**	(**24.9)**	**23.9**	(**23.2)**	**211,603.1**	**(204,664.7)**	**191,016**
	**Total population**		529,918	**24.8**	(**24.8)**	**5.4**	(**5.4)**	**44.0**	(**44.0)**	**14.4**	(**14.4)**	**7.1**	(**7.1)**		**24.5**	(**24.5)**	**24.5**	(**24.5)**	**24.5**	(**24.5)**	**24.5**	(**24.5)**	**207,874.5**	**(207,050.9)**	**195,597**
			138,625																						
	Depression medicine ^c^ **	ATC: N06A	102,568	36.2	(35.5)	1.7	(3.4)	41.2	(41.9)	13.2	(11.8)	4.3	(4.3)		23.6	(28.3)	38.4	(35.2)	23.9	(22.5)	13.8	(13.6)	183,196.8	(177,001.5)	138,760
	Antipsychotic medicine ^c^ **	ATC: N05A	7,468	46.5	(46.6)	2.3	(3.4)	34.0	(33.8)	9.3	(8.8)	3.0	(3.0)		26.9	(29.8)	45.5	(43.5)	20.3	(19.5)	6.9	(6.7)	163,651.2	(159,445.8)	114,375
	Indication prescribed anxiety medicine ^c^ **	All prescrib. w.indication codes 163 (for anxiety) or 371 (for anxiety, addictive)	129,484	37.0	(37.2)	1.7	(3.0)	40.6	(40.7)	13.2	(11.6)	4.3	(4.3)		25.1	(29.4)	38.8	(36.4)	23.2	(21.4)	12.6	(12.5)	178,542.0	(173,346.2)	126,524
	Heart failure medication ^c^ **	ATC: C01AA05, C03, C07 or C09A with indication code 430 (for heart failure)	**688,006**	41.9	(35.2)	0.1	(3.6)	40.5	(42.5)	8.9	(11.8)	4.2	(3.5)		22.1	(25.3)	43.3	(37.2)	19.6	(23.1)	14.8	(13.8)	189,647.8	(181,334.0)	230,031
	Ischaemic heart medication ^c^ **	ATC: C01A, C01B, C01D, C01E		44.5	(34.8)	0.0	(3.9)	36.8	(42.9)	9.1	(10.8)	3.3	(3.7)		23.6	(25.6)	44.9	(33.8)	18.4	(24.0)	12.9	(16.2)	184,004.0	(185,726.0)	169,155
	**All five types of the medicine above**		**4,555,439**	**38.2**	(**36.9)**	**1.6**	(**3.5)**	**40.0**	(**40.7)**	**12.2**	(**11.3)**	**4.1**	(**4.1)**		**23.9**	(**28.5)**	**39.5**	(**35.6)**	**22.9**	(**22.2)**	**13.4**	(**13.2)**	**182,468.1**	(**176,129.8)**	**142,957**
	**Extra**																								
	Ischaemic Heart Diseases	I05-I06; I11-I13; I20-I28; I30-I52	315,901	38.2	(30.9)	0.3	(3.2)	40.9	(44.6)	11.4	(13.1)	4.8	(5.4)		21.4	(24.7)	38.8	(30.1)	21.2	(24.1)	18.4	(20.6)	189,230.4	(198,833.6)	195,878
	Artritis	M01-M03; M5-M9; M7-M14; M15-M20; M45	505,792	34.7	(28.1)	0.3	(3.4)	43.9	(47.4)	13.5	(13.6)	4.6	(5.4)		19.8	(23.5)	34.9	(28.3)	23.9	(25.6)	21.2	(22.2)	201,022.4	(203,946.2)	190,758
	Arthrosis	M15-M19	338,166	37.1	(29.4)	0.1	(2.5)	43.0	(48.9)	12.9	(13.0)	3.7	(4.0)		20.6	(22.8)	36.7	(29.7)	23.0	(26.2)	19.5	(21.0)	208,141.8	(202,121.6)	187,430
	Back conditions	M32-34; M41-M43; M46-49; M50-51; M53-M54	212,948	33.2	(30.4)	1.3	(4.4)	44.9	(45.7)	13.2	(12.4)	4.6	(4.6)		20.3	(24.8)	34.1	(30.8)	24.7	(24.3)	20.6	(19.6)	203,248.9	(197,057.1)	207,419
	Overweight	E66	220,928	30.8	(36.8)	0.8	(3.3)	45.9	(44.1)	16.1	(10.4)	4.0	(3.0)		16.0	(23.7)	33.4	(34.2)	31.8	(25.1)	18.5	(16.6)	206,277.9	(188,909.3)	126,742
	Endometriosis	N80	29,190	21.2	(13.2)	0.5	(1.1)	46.8	(21.7)	22.8	(16.4)	7.2	(2.7)		12.8	(11.7)	25.5	(21.5)	35.3	(13.8)	26.1	(9.1)	218,423.8	(106,273.3)	104,183

Missing categories not showed but can be identified as the reaming difference to 100%.

Age and gender standardized estimates (std) in brackets. SD = Standard Deviation.

ICD-10 International Statistical Classification of Diseases, 10^th^ Revision

^c^ = complex defined conditions; see reference for further details [55].

Conditions no. marked ‘A’, overlap with other conditions and are thus not counted twice [55].

* Overall population frequencies are adapted from Hvidberg et al. 2019 [56].

** 2-year prevalence.

**Table 5:** Catalogue of odds ratios (OR) on socioeconomic differences of 199 chronic conditions: Multiple binary logistic regression models on educational levels and income quartiles, in Denmark on 1 January 2013.

			OR and rank of education levels * Reference: higher education. ** Reference: no education											OR of Income quartiles *** Reference: 4^the^ quartile **** Reference: 1^st^ quartile							
No.	Name of condition	ICD-10 code / definition	No education or training*		Students or in training*		Shorter education*		Middle education (BSc or equal)*		Higher (MSc degree or doctorate)**		Raw Rank	1^st^ quartile ***		2^nd^ quartile ****		3^rd^ quartile ****		4^th^ quartile ****	
			OR	p.	OR	p.	OR	p.	OR	p.	OR	p.		OR	p.	OR	p.	OR	p.	OR	p.
1	Chronic viral hepatitis	B18	**3.7**	**< .0001**	3.2	0.0018	1.3	0.0069	1.2	0.1060	0.3	< .0001	13	6.5	< .0001	0.9	0.0592	0.3	< .0001	0.2	< .0001
2	Human immunodeficiency virus [HIV] disease	B20-24	1.1	0.1682	1.3	0.5857	1.0	0.8633	0.9	0.2685	0.9	0.1682	196	2.3	< .0001	1.0	0.9696	0.7	< .0001	0.4	< .0001
3	Malignant neoplasms of other and unspecified localizations	C00-C14; C30-C33; C37-C42; C45-C49; C69; C73-74; C754-C759	1.1	0.0225	1.2	0.5346	1.0	0.2356	1.0	0.9873	0.9	0.0225	135	1.1	0.0058	1.0	0.0789	1.0	0.1687	0.9	0.0058
4	Malignant neoplasms of digestive organs	C15-C17; C22-C26	1.1	0.2680	2.6	0.3830	1.1	0.2311	1.0	0.8629	0.9	0.2680	65	1.2	0.0008	0.9	0.0106	0.9	0.0108	0.8	0.0008
5	Malignant neoplasm of colon	C18	0.8	< .0001	0.1	0.1663	0.9	0.0052	0.9	0.0117	1.2	< .0001	61	0.9	< .0001	1.0	0.9341	1.0	0.0719	1.1	< .0001
6	Malignant neoplasms of rectosigmoid junction, rectum, anus and anal canal	C19-C21	1.0	0.4099	<0.001	0.9796	1.1	0.2536	1.0	0.8786	1.0	0.4099	67	0.9	0.0588	1.0	0.1806	1.0	0.6698	1.1	0.0588
7	Malignant neoplasm of bronchus and lung	C34	**1.5**	**< .0001**	0.1	0.4162	1.4	< .0001	1.1	0.2620	0.7	< .0001	31	1.4	< .0001	1.0	0.1285	0.8	< .0001	0.7	< .0001
8	Malignant melanoma of skin	C43	0.6	< .0001	0.7	0.1870	0.8	< .0001	0.9	0.0325	**1.8**	**< .0001**	206	0.6	< .0001	1.1	0.0443	1.3	< .0001	1.7	< .0001
9	Other malignant neoplasms of skin	C44	0.7	< .0001	0.5	0.4750	0.8	< .0001	0.9	< .0001	**1.4**	**< .0001**	125	0.8	< .0001	1.0	0.7653	1.1	< .0001	1.3	< .0001
10	Malignant neoplasm of breast	C50	0.7	< .0001	0.6	0.3829	0.8	< .0001	0.9	< .0001	**1.4**	**< .0001**	115	0.8	< .0001	1.0	0.6226	1.1	< .0001	1.2	< .0001
11	Malignant neoplasms of female genital organs	C51-C52; C56-C58	0.9	0.3103	1.0	0.9756	0.9	0.2250	0.9	0.1178	1.1	0.3103	77	1.1	0.0798	1.0	0.9558	0.9	0.1510	0.9	0.0798
12	Malignant neoplasm of cervix uteri, corpus uteri and part unspecified	C53-C55	1.1	0.0216	1.1	0.9067	1.1	0.1147	1.1	0.3028	0.9	0.0216	70	1.0	0.7474	1.0	0.8142	1.1	0.0772	1.0	0.7474
13	Malignant tumour of the male genitalia	C60, C62-C63	0.8	0.0004	0.6	0.2767	1.0	0.3408	0.9	0.0454	1.2	0.0004	214	0.7	< .0001	1.1	0.0717	1.2	0.0004	1.4	< .0001
14	Malignant neoplasm of prostate	C61	0.7	< .0001	<0.001	0.9716	0.8	< .0001	0.9	0.0517	**1.4**	**< .0001**	158	0.7	< .0001	1.0	0.7362	1.2	< .0001	1.4	< .0001
15	Malignant neoplasms of urinary tract	C64-C68	1.2	0.0001	1.0	0.9817	1.2	0.0003	1.1	0.3546	0.8	0.0001	62	1.3	< .0001	0.9	0.0052	0.9	0.0147	0.8	< .0001
16	Brain cancer^a^	C71, C75.1-C75.3, D33.0-D33.2, D35.2-D35.4, D43.0-D43.2, D44.3-D44.5 (brain). C70, D32, D42 (brain membrane). C72, D33.3-D33.9, D43.3-D43.9 (cranial nerve, spinal cord)	0.9	0.1278	0.7	0.3726	1.0	0.3676	1.0	0.6544	1.1	0.1278	146	1.0	0.6338	1.0	0.8947	0.9	0.0633	1.0	0.6338
17	Malignant neoplasms of ill-defined, secondary and unspecified sites, and of independent (primary) multiple sites	C76-C80, C97	1.1	0.0455	0.5	0.2009	1.0	0.4457	1.0	0.4361	0.9	0.0455	118	1.1	0.0429	1.0	0.1957	0.9	0.0145	1.0	0.0429
18	Malignant neoplasms, stated or presumed to be primary, of lymphoid, haematopoietic and related tissue	C81-C96	0.8	< .0001	0.5	0.1441	0.9	< .0001	1.0	0.3419	1.2	< .0001	129	1.0	0.0891	1.0	0.2702	1.0	0.7891	1.0	0.0891
19	In situ neoplasms	D00-D09	0.9	0.0011	0.9	0.6934	1.0	0.2075	1.0	0.6503	1.1	0.0011	186	0.8	< .0001	1.1	< .0001	1.3	< .0001	1.3	< .0001
20	Haemolytic anaemias	D55-D59	1.3	0.0009	2.5	0.0203	1.0	0.6038	0.9	0.1070	0.7	0.0009	136	1.7	< .0001	0.9	0.0128	0.7	< .0001	0.6	< .0001
21	Aplastic and other anaemias	D60-D63	1.0	0.3823	4.0	< .0001	1.0	0.7742	1.0	0.6669	1.0	0.3823	39	1.0	0.5901	1.1	0.0176	1.1	0.0239	1.0	0.5901
22	Other anaemias	D64	1.4	< .0001	2.5	0.0002	1.1	< .0001	1.1	0.0350	0.7	< .0001	18	1.2	< .0001	1.1	< .0001	1.0	0.2188	0.8	< .0001
23	Coagulation defects, purpura and other haemorrhagic conditions	D65-D69	0.9	< .0001	0.9	0.7327	0.9	< .0001	0.9	0.0263	1.1	< .0001	185	0.8	< .0001	1.1	< .0001	1.2	< .0001	1.2	< .0001
24	Other diseases of blood and blood-forming organs	D70-D77	1.1	0.0173	0.5	0.3340	1.1	0.1428	1.1	0.2374	0.9	0.0173	108	1.2	< .0001	1.0	0.2343	0.9	0.0147	0.8	< .0001
25	Certain disorders involving the immune mechanism	D80-D89	0.9	0.1524	0.5	0.1561	1.0	0.6143	1.0	0.6135	1.1	0.1524	182	0.9	0.0006	1.1	0.1528	1.1	0.0014	1.2	0.0006
26	Diseases of the thyroid^a^	E00–E04, E06, E07	1.0	0.2760	0.9	0.2191	1.0	0.3141	1.0	0.1960	1.0	0.2760	121	1.0	0.0804	1.0	0.0368	1.0	0.5508	1.0	0.0804
27	Thyrotoxicosis^a^	E05	1.4	< .0001	0.9	0.7821	1.2	< .0001	1.1	0.0242	0.7	< .0001	55	1.2	< .0001	1.1	< .0001	0.9	< .0001	0.8	< .0001
28	Diabetes type 1^a^	E10	1.0	0.4457	1.3	0.3165	1.0	0.4157	1.0	0.6466	1.0	0.4457	194	1.0	0.2003	1.2	< .0001	1.1	< .0001	1.0	0.2003
29	Diabetes type 2^a^	E11	**1.9**	**< .0001**	1.6	0.0048	1.4	< .0001	1.2	< .0001	0.5	< .0001	33	1.8	< .0001	1.0	< .0001	0.7	< .0001	0.5	< .0001
30	Diabetes others^a^	E12–E14	**1.5**	**0.0114**	2.1	0.4858	1.2	0.1651	1.1	0.6478	0.7	0.0114	111	1.7	< .0001	1.0	0.5425	0.9	0.1523	0.6	< .0001
31	Disorders of other endocrine glands	E20-E35, except E30	0.9	0.0207	1.2	0.1934	0.9	0.0231	1.0	0.4706	1.1	0.0207	170	1.0	0.9765	1.1	0.0068	1.1	0.0038	1.0	0.9765
32	Metabolic disorders	E70-E77; E79-E83; E85, E88-E89;	1.1	0.0470	1.0	0.9377	0.9	0.0412	1.0	0.5067	0.9	0.0470	127	1.1	0.0056	1.0	0.1004	1.0	0.4102	0.9	0.0056
33	Disturbances in lipoprotein circulation and other lipids^a^	E78	1.4	< .0001	0.9	0.7509	1.3	< .0001	1.2	< .0001	0.7	< .0001	58	1.1	< .0001	0.9	< .0001	0.9	< .0001	0.9	< .0001
34	Cystic fibrosis^a^	E84	**0.5**	**< .0001**	0.4	0.2917	0.4	< .0001	0.5	< .0001	1.9	< .0001	201	0.8	0.0910	1.3	0.0098	1.2	0.1939	1.3	0.0910
35	Inflammatory diseases of the central nervous system	G00-G09	0.9	0.2391	1.0	0.9707	1.0	0.7579	1.0	0.8719	1.1	0.2391	162	0.9	0.0029	1.0	0.4941	1.1	0.0333	1.1	0.0029
36	Systemic atrophies primarily affecting the central nervous system and other degenerative diseases	G10-G14, G30-G32	0.8	< .0001	0.5	0.4765	0.8	0.0002	0.8	0.0006	1.3	< .0001	40	0.8	< .0001	1.1	0.0014	1.2	< .0001	1.3	< .0001
37	Parkinson’s disease^a^	G20, G21, G22, F02.3	1.4	< .0001	0.6	0.1187	1.2	< .0001	1.1	0.0342	0.7	< .0001	12	1.2	< .0001	1.1	< .0001	1.2	< .0001	0.8	< .0001
38	Extrapyramidal and movement disorders	G23-G26	0.9	0.0103	1.5	0.2509	0.9	0.0006	1.0	0.5755	1.1	0.0103	79	1.0	0.7443	1.0	0.8960	1.0	0.1679	1.0	0.7443
39	Sclerosis	G35	1.0	0.4357	0.1	0.0015	1.1	0.0713	1.0	0.9863	1.0	0.4357	188	0.7	< .0001	1.4	< .0001	1.7	< .0001	1.4	< .0001
40	Demyelinating diseases of the central nervous system	G36-G37	0.9	0.4661	1.8	0.2089	1.1	0.1551	1.0	0.9505	1.1	0.4661	198	1.0	0.4301	1.0	0.7935	1.0	0.8183	1.1	0.4301
41	Epilepsy ^a^	G40–G41	**2.1**	**< .0001**	1.3	0.0856	1.3	< .0001	1.2	< .0001	0.5	< .0001	22	1.5	< .0001	1.2	< .0001	1.0	0.0065	0.7	< .0001
42	Migraine^a^	G43	1.0	0.0597	0.9	0.4335	1.0	0.0016	1.0	0.0833	1.0	0.0597	195	0.9	< .0001	1.1	< .0001	1.1	< .0001	1.1	< .0001
43	Other headache syndromes	G44	**1.6**	**< .0001**	1.0	0.9421	1.3	< .0001	1.2	< .0001	0.6	< .0001	173	1.2	< .0001	1.0	0.8120	0.9	< .0001	0.8	< .0001
44	Transient cerebral ischaemic attacks and related syndromes and vascular syndromes of brain in cerebrovascular diseases	G45-G46	0.9	0.0002	0.3	0.1257	0.9	0.0296	1.0	0.9809	1.1	0.0002	71	0.9	0.0002	1.0	0.7676	1.0	0.6774	1.1	0.0002
45	Sleep disorders	G47	1.0	0.9626	0.6	0.0588	1.1	< .0001	1.1	0.0003	1.0	0.9626	191	0.8	< .0001	1.0	0.1287	1.2	< .0001	1.2	< .0001
46	Disorders of trigeminal nerve and facial nerve disorders	G50-G51	1.2	< .0001	1.4	0.1782	1.2	< .0001	1.1	0.0780	0.8	< .0001	150	1.1	0.0292	1.0	0.3709	0.9	0.0009	0.9	0.0292
47	Disorders of other cranial nerves, cranial nerve disorders in diseases classified elsewhere, nerve root and plexus disorders and Nerve root and plexus compressions in diseases classified elsewhere	G52-G55	1.1	0.0096	0.2	0.0911	1.1	0.0270	1.0	0.8626	0.9	0.0096	147	1.0	0.3605	1.1	0.0158	1.0	0.3054	1.0	0.3605
48	Mononeuropathies of upper limb	G56	**2.5**	**< .0001**	2.6	< .0001	2.1	< .0001	1.4	< .0001	0.4	< .0001	105	1.4	< .0001	1.1	< .0001	1.0	0.4854	0.7	< .0001
49	Mononeuropathies of lower limb, other mononeuropathies and mononeuropathy in diseases classified elsewhere	G57-G59	1.3	< .0001	1.3	0.4118	1.2	< .0001	1.2	< .0001	0.8	< .0001	154	1.0	0.5604	1.0	0.3014	1.0	0.6893	1.0	0.5604
50	Polyneuropathies and other disorders of the peripheral nervous system	G60-G64	0.9	< .0001	1.3	0.4585	0.9	< .0001	0.9	0.0166	1.1	< .0001	91	1.0	0.4780	1.0	0.1233	1.0	0.5627	1.0	0.4780
51	Diseases of myoneural junction and muscle	G70-G73	1.1	0.0846	0.8	0.6095	0.9	0.0387	0.9	0.0846	0.9	0.0846	130	0.9	0.0051	1.2	0.0002	1.2	< .0001	1.2	0.0051
52	Cerebral palsy and other paralytic syndromes	G80-G83	**2.6**	**< .0001**	0.5	0.1122	1.1	0.1424	1.0	0.4005	0.4	< .0001	11	0.7	< .0001	1.8	< .0001	1.8	< .0001	1.4	< .0001
53	Other disorders of the nervous system	G90-G99	1.1	0.0045	1.0	0.8379	1.0	0.5210	1.0	0.1377	0.9	0.0045	126	1.0	0.3843	1.0	0.0060	1.0	0.0075	1.0	0.3843
54	Disorders of eyelid, lacrimal system and orbit	H02-H06	1.0	0.7222	0.9	0.8753	1.0	0.2481	1.0	0.5649	1.0	0.7222	131	0.9	0.0515	1.0	0.5737	1.0	0.3028	1.1	0.0515
55	Corneal scars and opacities	H17	**1.7**	**< .0001**	3.1	0.0758	1.5	< .0001	1.3	0.0489	0.6	< .0001	120	1.5	< .0001	0.9	0.0173	0.8	< .0001	0.7	< .0001
56	Other disorders of cornea	H18	0.8	< .0001	0.3	0.0916	0.8	< .0001	0.9	0.0192	1.3	< .0001	139	0.9	0.0040	1.0	0.3049	1.1	0.0518	1.1	0.0040
57	Diseases of the eye lens (cataracts)	H25-H28	1.0	0.2936	0.2	0.1531	1.0	0.4757	1.0	0.7220	1.0	0.2936	21	1.2	< .0001	1.0	< .0001	0.9	< .0001	0.9	< .0001
58	Disorders of the choroid and retina	H31-H32	1.1	0.3237	0.2	0.3050	1.1	0.3406	1.2	0.1127	0.9	0.3237	156	1.1	0.4458	0.9	0.1341	0.9	0.2503	0.9	0.4458
59	Retinal vascular occlusions	H34	0.8	0.0006	1.3	0.7922	0.8	< .0001	0.8	0.0012	1.2	0.0006	56	1.0	0.7139	1.0	0.7529	1.0	0.7786	1.0	0.7139
60	Other retinal disorders	H35	0.8	< .0001	0.8	0.5504	0.9	< .0001	0.9	0.0004	1.2	< .0001	41	0.9	< .0001	1.1	< .0001	1.1	< .0001	1.2	< .0001
61	Retinal disorders in diseases classified elsewhere	H36	0.8	< .0001	0.5	0.1491	0.9	0.0001	0.9	0.0606	1.2	< .0001	97	1.1	0.0176	0.9	0.0150	0.9	0.0125	0.9	0.0176
62	Glaucoma ^c^	H40–H42	0.9	< .0001	0.9	0.7527	0.9	< .0001	0.9	0.0043	1.2	< .0001	59	0.9	< .0001	1.0	0.0010	1.1	< .0001	1.1	< .0001
63	Disorders of the vitreous body and globe	H43-H45	**0.5**	**< .0001**	0.7	0.4859	0.6	< .0001	0.8	< .0001	1.9	< .0001	189	0.8	< .0001	1.0	0.6826	1.1	0.0271	1.3	< .0001
64	Disorders of optic nerve and visual pathways	H46-H48	1.1	0.1291	1.3	0.6208	1.1	0.0812	1.0	0.6353	0.9	0.1291	166	1.0	0.7669	1.2	< .0001	1.1	0.2515	1.0	0.7669
65	Disorders of ocular muscles, binocular movement, accommodation and refraction	H49-H52	0.6	< .0001	0.4	< .0001	0.7	< .0001	0.8	< .0001	1.7	< .0001	213	0.6	< .0001	1.1	< .0001	1.2	< .0001	1.6	< .0001
66	Visual disturbances	H53	1.0	0.4343	1.7	0.0194	0.9	0.0324	1.0	0.3886	1.0	0.4343	119	1.0	0.3063	1.1	0.0064	1.0	0.6131	1.0	0.3063
67	Blindness and partial sight	H54	1.5	< .0001	1.0	0.9515	1.0	0.8190	0.9	0.0505	0.7	< .0001	26	1.2	0.0009	1.2	< .0001	1.2	0.0002	0.8	0.0009
68	Nystagmus and other irregular eye movements and other disorders of eye and adnexa	H55, H57	0.7	< .0001	1.1	0.7333	0.7	< .0001	0.8	< .0001	1.4	< .0001	168	0.8	< .0001	1.0	0.4342	1.0	0.2302	1.2	< .0001
69	Otosclerosis	H80	1.0	0.4675	0.8	0.7381	1.0	0.7022	1.0	0.8297	1.0	0.4675	148	1.0	0.4403	1.0	0.8848	1.0	0.3664	1.0	0.4403
70	Ménière’s disease^a^	H810	1.1	0.0099	0.6	0.6305	1.2	0.0041	1.1	0.1644	0.9	0.0099	100	1.1	0.0316	1.0	0.3091	1.0	0.1543	0.9	0.0316
71	Other diseases of the inner ear	H83	**2.8**	**< .0001**	0.4	0.3408	2.7	< .0001	1.5	< .0001	0.4	< .0001	95	1.7	< .0001	1.0	0.0224	0.8	< .0001	0.6	< .0001
72	Conductive and sensorineural hearing loss	H90	1.1	< .0001	1.3	0.2494	1.0	0.8868	1.0	0.6410	0.9	< .0001	98	1.0	0.1252	1.1	< .0001	1.0	0.3104	1.0	0.1252
73	Other hearing loss and other disorders of ear, not elsewhere classified	H910, H912, H913, H918, H930, H932, H933	1.3	< .0001	1.4	0.5653	1.2	0.0037	1.2	0.0065	0.8	< .0001	93	1.1	0.0195	1.0	0.1618	1.0	0.7963	0.9	0.0195
74	Presbycusis (age-related hearing loss)	H911	0.9	0.0167	2.4	0.1091	0.9	0.0002	1.0	0.0783	1.1	0.0167	19	1.0	0.0114	1.1	< .0001	1.1	< .0001	1.0	0.0114
75	Hearing loss, unspecified	H919	0.9	0.0007	1.2	0.6006	1.0	0.0975	1.1	0.0169	1.1	0.0007	101	0.9	0.0001	1.0	0.2527	1.1	< .0001	1.1	0.0001
76	Tinnitus	H931	0.8	< .0001	0.6	0.0443	0.8	< .0001	1.0	0.3376	1.2	< .0001	159	1.0	0.1994	1.0	0.5239	1.0	0.0060	1.0	0.1994
77	Other specified disorders of ear	H938	1.1	0.0049	0.3	0.0741	1.1	0.0206	1.2	0.0001	0.9	0.0049	80	1.1	0.0008	1.0	0.2712	1.0	0.0468	0.9	0.0008
78	Aortic and mitral valve disease^a^	I05, I06, I34, I35	0.9	0.0613	0.6	0.4079	0.9	0.0019	0.9	0.0163	1.1	0.0613	23	1.0	0.6065	1.0	0.3627	1.0	0.1093	1.0	0.6065
79	Hypertensive diseases^a^	I10-I15	1.4	< .0001	1.0	0.6465	1.3	< .0001	1.1	< .0001	0.7	< .0001	81	1.2	< .0001	1.1	< .0001	1.0	< .0001	0.9	< .0001
80	Heart failure^a^	I11.0, I13.0, I13.2, I42.0, I42.6, I42.7, I42.9, I50.0, I50.1, I50.9	1.0	0.1812	1.4	0.5987	1.0	0.5388	0.9	0.0129	1.0	0.1812	32	1.1	< .0001	1.0	0.0043	1.0	0.0945	0.9	< .0001
81	Angina pectoris	I20	1.1	0.0140	0.7	0.5550	1.0	0.9941	1.0	0.8099	0.9	0.0140	52	1.1	< .0001	0.9	< .0001	0.9	< .0001	0.9	< .0001
82	Acute myocardial infarction and subsequent myocardial infarction	I21-I22	1.2	< .0001	0.1	0.0810	1.1	< .0001	1.1	0.0161	0.8	< .0001	42	1.1	< .0001	1.0	0.0337	1.0	0.3470	0.9	< .0001
83	AMI complex/other	I23-I24	1.3	0.0443	0.2	0.7299	1.2	0.1699	1.3	0.0331	0.8	0.0443	47	1.1	0.2322	1.0	0.4270	0.9	0.1358	0.9	0.2322
84	Chronic ischaemic heart disease	I25	1.0	0.2605	1.7	0.3938	1.0	0.3256	0.9	0.0223	1.0	0.2605	34	1.2	< .0001	1.0	< .0001	0.9	< .0001	0.9	< .0001
85	Pulmonary heart disease and diseases of pulmonary circulation	I26-I28	1.1	0.0987	1.1	0.9040	1.1	0.1257	1.0	0.9517	0.9	0.0987	48	1.0	0.7857	1.0	0.0554	1.0	0.7841	1.0	0.7857
86	Acute pericarditis	I30	1.1	0.0959	1.7	0.2182	1.1	0.0856	1.0	0.8514	0.9	0.0959	174	1.0	0.7512	1.0	0.7742	1.0	0.8380	1.0	0.7512
87	Other forms of heart disease	I31-I43, except I34-I35 and I42	0.9	0.0151	1.0	0.9926	0.9	0.0117	0.9	0.0723	1.1	0.0151	92	0.9	0.0048	0.9	0.0185	1.0	0.7027	1.1	0.0048
88	Atrioventricular and left bundle-branch block	I44	0.9	0.1924	1.6	0.5034	0.9	0.1158	1.0	0.6559	1.1	0.1924	46	1.0	0.7347	1.0	0.0793	1.1	0.0079	1.0	0.7347
89	Other conduction disorders	I45-46	1.0	0.4277	1.9	0.0739	1.0	0.4883	0.9	0.0238	1.0	0.4277	104	1.0	0.4341	1.0	0.1577	1.1	0.0882	1.0	0.4341
90	Paroxysmal tachycardia	I47	0.9	< .0001	1.1	0.7243	0.9	0.0105	1.0	0.5039	1.1	< .0001	138	0.9	< .0001	1.0	0.3077	1.0	0.0763	1.1	< .0001
91	Atrial fibrillation and flutter	I48	0.7	< .0001	0.7	0.3106	0.8	< .0001	0.9	< .0001	1.3	< .0001	49	0.9	< .0001	1.0	0.0203	1.1	< .0001	1.2	< .0001
92	Other cardiac arrhythmias	I49	0.8	< .0001	0.3	0.0070	0.8	< .0001	1.0	0.5504	1.3	< .0001	113	0.9	< .0001	1.0	0.7890	1.1	0.0035	1.2	< .0001
93	Complications and ill-defined descriptions of heart disease and other heart disorders in diseases classified elsewhere	I51-52	0.7	< .0001	0.5	0.3167	0.7	< .0001	0.8	0.0050	1.4	< .0001	89	0.8	< .0001	1.0	0.9764	1.1	0.2036	1.2	< .0001
94	Stroke	I60, I61,I63-I64, Z501 (rehabilitation)	1.1	0.0003	1.0	0.9320	1.1	0.0297	1.0	0.1368	0.9	0.0003	36	1.1	< .0001	1.1	< .0001	1.0	0.3570	0.9	< .0001
95	Cerebrovascular diseases	I62, I65-I68	0.8	< .0001	1.2	0.7945	0.8	< .0001	0.9	0.0018	1.2	< .0001	64	0.9	0.0503	1.0	0.1897	1.0	0.0871	1.1	0.0503
96	Sequelae of cerebrovascular disease	I69	1.0	0.6180	0.7	0.7009	1.0	0.1362	1.0	0.9076	1.0	0.6180	20	1.1	0.0003	1.1	< .0001	1.1	0.0016	0.9	0.0003
97	Atherosclerosis	I70	**1.7**	**< .0001**	0.4	0.4467	1.4	< .0001	1.1	0.0227	0.6	< .0001	10	1.6	< .0001	1.1	0.0002	0.9	< .0001	0.6	< .0001
98	Aortic aneurysm and aortic dissection	I71	0.9	0.0313	0.1	0.4481	1.0	0.6760	1.0	0.7870	1.1	0.0313	60	1.1	0.0214	1.0	0.0621	1.0	0.4990	0.9	0.0214
99	Diseases of arteries, arterioles and capillaries	I72, I74, I77-I79	0.7	< .0001	1.0	0.9770	0.8	< .0001	0.9	0.0120	1.4	< .0001	145	0.8	< .0001	1.0	0.3402	1.0	0.2763	1.2	< .0001
100	Other peripheral vascular diseases	I73	**1.5**	**< .0001**	0.5	0.3859	1.4	< .0001	1.1	0.1857	0.7	< .0001	17	1.4	< .0001	1.1	0.0003	0.9	< .0001	0.7	< .0001
101	Phlebitis, thrombosis of the portal vein and others	I80-I82	1.4	< .0001	1.4	0.1703	1.2	< .0001	1.0	0.3057	0.7	< .0001	83	1.1	< .0001	1.1	0.0002	1.0	0.3710	0.9	< .0001
102	Varicose veins of lower extremities	I83	1.3	< .0001	1.2	0.4820	1.5	< .0001	1.4	< .0001	0.7	< .0001	178	0.9	0.0023	1.1	0.0001	1.2	< .0001	1.1	0.0023
103	Haemorrhoids^a^	I84	1.0	0.0476	0.9	0.2775	1.1	< .0001	1.2	< .0001	1.0	0.0476	199	0.8	< .0001	1.1	< .0001	1.2	< .0001	1.2	< .0001
104	Oesophageal varices (chronic), varicose veins of other sites, other disorders of veins, nonspecific lymphadenitis, other noninfective disorders of lymphatic vessels and lymph nodes and other and unspecified disorders of the circulatory system	I85-I99, except I89 and I95	1.0	0.6540	0.9	0.8160	1.0	0.7672	1.0	0.7506	1.0	0.6540	132	1.1	0.0815	1.0	0.9470	1.0	0.5869	0.9	0.0815
105	Respiratory allergy^a^	J30, except J30.0	0.6	< .0001	0.6	< .0001	0.8	< .0001	0.9	< .0001	1.6	< .0001	202	0.7	< .0001	1.1	< .0001	1.2	< .0001	1.4	< .0001
105A	Chronic lower respiratory diseases^a^	J40-J43, J47	1.1	< .0001	0.9	0.0763	1.0	< .0001	1.0	< .0001	1.0	< .0001	133	1.0	0.2684	1.0	< .0001	1.0	0.6096	1.0	0.2684
106	Bronchitis, not specified as acute or chronic, simple and mucopurulent chronic bronchitis and unspecified chronic bronchitis	J40-J42	**1.8**	**< .0001**	1.0	0.9779	1.4	< .0001	1.1	0.0556	0.6	< .0001	4	2.0	< .0001	1.0	0.0966	0.8	< .0001	0.5	< .0001
107	Emphysema	J43	**1.8**	**< .0001**	2.1	0.4656	1.5	< .0001	1.2	0.0794	0.6	< .0001	16	1.5	< .0001	1.0	0.2472	0.9	0.1012	0.6	< .0001
108	Chronic obstructive lung disease (COPD)^a^	J44, J96, J13-J18	**1.9**	**< .0001**	1.2	0.0777	1.5	< .0001	1.2	< .0001	0.5	< .0001	37	1.6	< .0001	1.0	< .0001	0.9	< .0001	0.6	< .0001
109	Asthma, status asthmaticus^a^	J45-J46	1.1	< .0001	1.3	0.0003	1.0	0.0111	1.1	< .0001	0.9	< .0001	142	1.2	< .0001	1.0	0.5753	0.9	< .0001	0.9	< .0001
110	Bronchiectasis	J47	**0.5**	**< .0001**	0.2	0.2372	0.7	< .0001	0.9	0.2450	**1.8**	**< .0001**	153	0.7	< .0001	0.9	0.1948	1.2	0.0002	1.4	< .0001
111	Other diseases of the respiratory system	J60-J84; J95, J97-J99	1.3	< .0001	2.2	0.0160	1.2	< .0001	1.1	0.0581	0.8	< .0001	53	1.1	0.0342	1.0	0.8695	1.0	0.7086	0.9	0.0342
112	Ulcers^a^	K25-K27	**1.7**	**< .0001**	2.0	< .0001	1.3	< .0001	1.1	< .0001	0.6	< .0001	57	1.4	< .0001	1.0	0.0082	0.9	< .0001	0.7	< .0001
113	Inguinal hernia	K40	1.0	0.2223	0.6	0.2727	1.0	0.1355	1.0	0.3774	1.0	0.2223	183	0.9	< .0001	1.0	0.7657	1.1	< .0001	1.1	< .0001
114	Ventral hernia	K43	**1.6**	**< .0001**	1.7	0.2602	1.3	< .0001	1.2	0.0065	0.6	< .0001	74	1.3	< .0001	1.0	0.5596	0.9	0.1212	0.8	< .0001
115	Crohn’s diease	K50	1.2	< .0001	1.2	0.3412	1.2	< .0001	1.1	0.0023	0.8	< .0001	187	1.0	0.8498	1.1	< .0001	1.1	0.0427	1.0	0.8498
116	Ulcerative colitis	K51	0.9	< .0001	0.7	0.1054	1.0	0.2370	1.1	0.0562	1.1	< .0001	197	0.8	< .0001	1.1	0.0007	1.2	< .0001	1.2	< .0001
117	Other noninfective gastroenteritis and colitis	K52	1.2	< .0001	1.4	0.1429	1.2	< .0001	1.1	0.0087	0.8	< .0001	76	1.1	0.0005	1.1	0.0076	1.0	0.9215	0.9	0.0005
118	Irritable bowel syndrome (IBS)	K58	1.2	< .0001	1.2	0.1544	1.3	< .0001	1.3	< .0001	0.9	< .0001	179	1.0	0.3684	1.0	0.0152	1.0	0.5176	1.0	0.3684
119	Other functional intestinal disorders	K59	1.2	< .0001	1.1	0.5923	1.1	< .0001	1.1	0.0272	0.8	< .0001	68	1.1	0.0001	1.1	< .0001	1.0	0.1487	0.9	0.0001
120	Diseases of liver, biliary tract and pancreas	K71-K77; K86-K87	1.3	< .0001	1.5	0.1468	1.2	< .0001	1.1	0.0275	0.8	< .0001	90	1.4	< .0001	1.0	0.0551	0.8	< .0001	0.7	< .0001
121	Psoriasis^a^	L40	0.9	0.0002	0.9	0.6103	1.0	0.6998	1.0	0.7750	1.1	0.0002	172	0.8	< .0001	1.1	< .0001	1.2	< .0001	1.2	< .0001
122	Infectious arthropathies	M01-M03	0.9	0.0062	0.8	0.6078	1.0	0.8455	1.0	0.4501	1.1	0.0062	203	0.8	< .0001	1.0	0.3739	1.0	0.2390	1.2	< .0001
123	Rheumatoid arthritis^a^	M05, M06, M07.1, M07.2, M07.3, M08, M09	0.7	< .0001	0.7	0.0523	0.7	< .0001	0.8	< .0001	1.4	< .0001	152	0.9	0.0004	1.0	0.1300	1.0	0.0007	1.1	0.0004
124	Inflammatory polyarthropathies—except rheumatoid arthritis^a^	M074–M079, M10–M14, M45	1.4	< .0001	0.7	0.0971	1.3	< .0001	1.1	< .0001	0.7	< .0001	114	1.1	< .0001	1.0	0.0001	1.0	0.0958	0.9	< .0001
125	Polyarthrosis [Arthrosis]	M15	1.0	0.5235	0.1	0.3951	1.0	0.7427	1.0	0.6639	1.0	0.5235	51	1.1	0.0001	0.9	0.0018	0.9	< .0001	0.9	0.0001
126	Coxarthrosis [arthrosis of hip]	M16	1.2	< .0001	0.7	0.4097	1.1	< .0001	1.1	0.0051	0.8	< .0001	30	1.1	< .0001	1.0	< .0001	1.0	0.4104	0.9	< .0001
127	Gonarthrosis [arthrosis of knee]	M17	**1.5**	**< .0001**	1.5	0.0940	1.5	< .0001	1.3	< .0001	0.7	< .0001	87	1.1	< .0001	1.0	0.0033	1.0	< .0001	0.9	< .0001
128	Arthrosis of first carpometacarpal joint and other arthrosis	M18-M19	**1.5**	**< .0001**	1.3	0.3171	1.5	< .0001	1.2	< .0001	0.7	< .0001	106	1.2	< .0001	1.0	0.0201	0.9	< .0001	0.9	< .0001
129	Acquired deformities of fingers and toes	M20	1.1	0.0002	1.3	0.1567	1.3	< .0001	1.2	< .0001	0.9	0.0002	165	0.9	< .0001	1.1	< .0001	1.2	< .0001	1.2	< .0001
130	Other acquired deformities of limbs	M21	1.3	< .0001	1.2	0.4662	1.3	< .0001	1.3	< .0001	0.8	< .0001	143	1.1	0.0107	1.0	0.0770	1.0	0.1238	0.9	0.0107
131	Disorders of patella (knee cap)	M22	1.4	< .0001	1.7	< .0001	1.4	< .0001	1.2	< .0001	0.7	< .0001	209	1.0	0.9800	1.3	< .0001	1.2	< .0001	1.0	0.9800
132	Internal derangement of knee	M230, M231, M233, M235, M236, M238	0.8	0.0001	0.9	0.5517	0.9	0.0746	1.0	0.8526	1.2	0.0001	215	1.0	0.5061	0.9	0.1084	1.0	0.1927	1.0	0.5061
133	Derangement of meniscus due to old tear or injury	M232	0.9	< .0001	1.2	0.2846	1.0	0.2706	1.0	0.1740	1.1	< .0001	211	0.8	< .0001	1.0	0.0186	1.2	< .0001	1.3	< .0001
134	Internal derangement of knee, unspecified	M239	1.2	< .0001	1.0	0.9767	1.3	< .0001	1.2	< .0001	0.8	< .0001	207	0.8	< .0001	1.0	0.0438	1.1	< .0001	1.2	< .0001
135	Other specific joint derangements	M24, except M240-M241	0.8	0.0008	0.6	0.1445	0.9	0.0550	0.9	0.2593	1.2	0.0008	210	0.9	0.1939	1.0	0.3638	1.0	0.4749	1.1	0.1939
136	Other joint disorders, not elsewhere classified	M25	1.4	< .0001	1.7	0.0574	1.3	< .0001	1.1	0.0077	0.7	< .0001	181	1.2	0.0002	1.0	0.7628	0.9	0.0107	0.9	0.0002
137	Systemic connective tissue disorders	M30-M36, except M32,M34	1.1	< .0001	1.0	0.9420	1.0	0.1493	1.1	0.0019	0.9	< .0001	84	1.1	0.0025	1.0	0.6394	1.0	0.0764	0.9	0.0025
138	Systemic lupus erythematosus	M32	1.3	0.0110	0.5	0.3547	1.2	0.0743	1.2	0.0341	0.8	0.0110	155	1.2	0.0212	1.0	0.4508	0.9	0.2257	0.8	0.0212
139	Dermatopolymyositis	M33	0.9	0.3207	1.5	0.7120	0.8	0.1503	0.8	0.1459	1.2	0.3207	123	1.0	0.8885	0.9	0.4902	1.1	0.6129	1.0	0.8885
140	Systemic sclerosis	M34	1.3	0.0900	0.1	0.0623	1.3	0.0677	1.2	0.2396	0.8	0.0900	85	1.1	0.1929	0.9	0.2016	1.0	0.9955	0.9	0.1929
141	Kyphosis, lordosis	M40	1.1	0.0891	0.3	0.1107	0.8	0.0027	1.0	0.7479	0.9	0.0891	144	1.4	< .0001	1.0	0.7499	0.8	< .0001	0.7	< .0001
142	Scoliosis	M41	1.0	0.4993	0.8	0.3434	0.8	< .0001	0.9	0.1130	1.0	0.4993	163	1.1	0.0072	1.1	< .0001	1.0	0.8363	0.9	0.0072
143	Spinal osteochondrosis	M42	**1.7**	**< .0001**	1.2	0.6868	1.3	< .0001	1.1	0.0560	0.6	< .0001	137	1.6	< .0001	1.1	0.0561	0.9	< .0001	0.6	< .0001
144	Other deforming dorsopathies	M43	1.4	< .0001	0.4	0.0565	1.2	< .0001	1.1	0.0003	0.7	< .0001	116	1.2	< .0001	1.0	0.6688	0.9	0.0117	0.9	< .0001
145	Other inflammatory spondylopathies	M46	1.1	0.0243	1.6	0.1910	1.1	0.0429	1.0	0.6276	0.9	0.0243	160	1.0	0.3595	1.1	0.0020	1.1	0.1286	1.0	0.3595
146	Spondylosis	M47	1.6	< .0001	1.8	0.0691	1.4	< .0001	1.2	< .0001	0.6	< .0001	66	1.4	< .0001	1.0	0.0004	0.8	< .0001	0.7	< .0001
147	Other spondylopathies and spondylopathies in diseases classified elsewhere	M48, M49	0.9	< .0001	1.2	0.6956	0.9	< .0001	0.9	0.0007	1.2	< .0001	54	0.9	< .0001	0.9	< .0001	1.0	0.2121	1.1	< .0001
148	Cervical disc disorders	M50	1.1	0.0074	0.5	0.2805	1.2	< .0001	1.1	0.0652	0.9	0.0074	190	0.9	0.0006	1.0	0.2964	1.0	0.4390	1.1	0.0006
149	Other intervertebral disc disorders	M51	1.5	< .0001	1.1	0.5309	1.4	< .0001	1.2	< .0001	0.7	< .0001	167	1.1	< .0001	1.0	0.0839	0.9	0.0020	0.9	< .0001
150	Other dorsopathies, not elsewhere classified	M53	1.4	< .0001	0.9	0.8440	1.4	< .0001	1.1	0.0815	0.7	< .0001	176	0.9	0.0234	1.0	0.4055	1.0	0.3217	1.1	0.0234
151	Dorsalgia	M54	**1.5**	**< .0001**	1.7	0.0028	1.3	< .0001	1.1	0.0026	0.7	< .0001	149	1.2	< .0001	1.1	< .0001	0.9	0.0007	0.9	< .0001
152	Soft tissue disorders	M60-M63, except M60.0	**1.6**	**< .0001**	1.9	0.0036	1.4	< .0001	1.2	0.0001	0.6	< .0001	157	1.2	< .0001	1.1	< .0001	1.0	0.5573	0.9	< .0001
153	Synovitis and tenosynovitis	M65	1.2	< .0001	1.0	0.9302	1.2	< .0001	1.1	0.0056	0.8	< .0001	177	0.9	0.0524	1.0	0.2275	1.1	0.0002	1.1	0.0524
154	Disorders of synovium and tendon	M66-68	1.2	< .0001	1.6	0.0136	1.3	< .0001	1.2	< .0001	0.8	< .0001	204	0.9	0.0003	1.1	< .0001	1.2	< .0001	1.1	0.0003
155	Soft tissue disorders related to use, overuse and pressure	M70	1.2	0.0004	0.7	0.2977	1.3	< .0001	1.3	< .0001	0.8	0.0004	164	0.9	0.0051	1.0	0.3364	1.1	< .0001	1.1	0.0051
156	Fibroblastic disorders	M72	1.0	0.0594	0.6	0.1572	1.2	< .0001	1.2	< .0001	1.0	0.0594	161	0.9	< .0001	1.0	0.3175	1.1	< .0001	1.1	< .0001
157	Shoulder lesions	M75	**1.8**	**< .0001**	2.0	< .0001	1.7	< .0001	1.4	< .0001	0.6	< .0001	175	1.0	0.0062	1.1	< .0001	1.0	0.0067	1.0	0.0062
158	Enthesopathies of lower limb, excluding foot	M76	0.5	< .0001	0.5	0.0107	0.7	< .0001	1.0	0.4104	2.0	< .0001	216	0.6	< .0001	1.0	0.2339	1.2	< .0001	1.6	< .0001
159	Other enthesopathies	M77	1.2	0.0006	0.5	0.1182	1.2	0.0001	1.0	0.5625	0.8	0.0006	200	0.7	< .0001	1.2	< .0001	1.2	< .0001	1.3	< .0001
160	Rheumatism, unspecified	M790	**2.7**	**< .0001**	0.9	0.9034	1.7	< .0001	1.2	0.0214	0.4	< .0001	38	2.8	< .0001	0.9	< .0001	0.6	< .0001	0.4	< .0001
161	Myalgia	M791	**2.2**	**< .0001**	2.5	0.0098	1.7	< .0001	1.3	< .0001	0.5	< .0001	94	1.5	< .0001	1.0	0.3236	0.8	< .0001	0.7	< .0001
162	Other soft tissue disorders, not elsewhere classified	M792- M794; M798-M799	1.0	0.7396	0.6	0.1211	1.0	0.7377	1.0	0.5855	1.0	0.7396	169	1.1	0.0871	1.0	0.3447	0.9	0.0739	0.9	0.0871
163	Other soft tissue disorders, not elsewhere classified: pain in limb	M796	**1.6**	**< .0001**	2.4	< .0001	1.4	< .0001	1.3	< .0001	0.6	< .0001	151	1.1	0.0047	1.1	0.0194	1.0	0.5149	0.9	0.0047
164	Fibromyalgia	M797	**4.2**	**< .0001**	0.9	0.9065	2.8	< .0001	2.0	< .0001	0.2	< .0001	72	3.9	< .0001	1.0	0.9085	0.6	< .0001	0.3	< .0001
165	Osteoporosis^a^	M80–M81	1.0	0.0032	0.4	0.1920	1.0	0.4342	1.0	0.1900	1.1	0.0032	25	1.0	0.0014	1.0	0.0036	1.1	< .0001	1.0	0.0014
166	Osteoporosis in diseases classified elsewhere	M82	1.2	0.3723	0.4	0.7516	1.0	0.8879	0.9	0.6115	0.9	0.3723	50	1.0	0.9202	1.0	0.8278	1.1	0.5685	1.0	0.9202
167	Adult osteomalacia and other disorders of bone density and structure	M83,M85, except M833	1.0	0.3320	0.9	0.7759	1.0	0.2613	1.1	0.0010	1.0	0.3320	117	1.0	0.0116	1.0	0.8213	1.1	< .0001	1.1	0.0116
168	Disorders of continuity of bone	M84	**1.5**	**0.0066**	1.4	0.7378	1.3	0.0242	1.3	0.1084	0.7	0.0066	110	1.2	0.0182	0.9	0.1856	0.7	< .0001	0.8	0.0182
169	Other osteopathies	M86-M90	**1.5**	**< .0001**	1.5	0.1730	1.4	< .0001	1.2	< .0001	0.7	< .0001	73	1.3	< .0001	1.0	0.6557	0.9	< .0001	0.8	< .0001
170	Other disorders of the musculoskeletal system and connective tissue	M95-M99	**1.6**	**< .0001**	1.9	< .0001	1.4	< .0001	1.2	< .0001	0.6	< .0001	141	1.3	< .0001	1.0	0.0164	0.9	< .0001	0.8	< .0001
171	Chronic renal failure (CRF)^a^	N18	1.2	0.0004	0.3	0.1159	1.1	0.0722	1.0	0.5261	0.9	0.0004	29	1.2	< .0001	1.0	0.3935	0.9	0.0003	0.9	< .0001
172	Congenital malformations: of the nervous, circulatory, respiratory system; cleft palate and cleft lip, urinary tract, bones and muscles, other and chromosomal abnormalities not elsewhere classified	Q00-Q07; Q20-Q37; Q60-Q99	1.1	< .0001	1.1	0.1337	1.0	0.0005	1.0	0.8369	0.9	< .0001	184	0.9	< .0001	1.3	< .0001	1.2	< .0001	1.1	< .0001
173	Congenital malformations of eye, ear, face and neck	Q10-Q18	1.1	0.1178	0.9	0.7543	1.0	0.7253	1.0	0.3212	1.0	0.1178	205	0.8	< .0001	1.1	< .0001	1.2	< .0001	1.2	< .0001
174	Other congenital malformations of the digestive system	Q38-Q45	1.1	0.2011	0.8	0.5958	1.0	0.8432	1.0	0.7702	0.9	0.2011	109	1.1	0.0718	1.0	0.4355	1.0	0.4613	0.9	0.0718
175	Congenital malformations of the sexual organs	Q50-Q56	0.9	0.0057	0.6	0.0312	0.9	0.0125	1.0	0.4999	1.1	0.0057	208	0.8	< .0001	1.1	0.0061	1.1	< .0001	1.2	< .0001
176	Dementia^a^	F00, G30, F01, F02.0, F03.9, G31.8B, G31.8E, G31.9, G31.0B	0.9	0.0001	0.0	0.0188	0.9	0.0067	0.9	0.0134	1.1	0.0001	5	0.9	< .0001	1.3	< .0001	1.4	< .0001	1.1	< .0001
177	Organic, including symptomatic, mental disorders	F04-F09	1.3	< .0001	0.9	0.6999	0.9	0.1060	0.8	< .0001	0.8	< .0001	8	1.0	0.8686	1.2	< .0001	1.3	< .0001	1.0	0.8686
178	Mental and behavioural disorders due to use of alcohol	F10	**2.1**	**< .0001**	1.7	0.0010	1.4	< .0001	1.1	0.0250	0.5	< .0001	35	3.7	< .0001	1.0	0.5654	0.5	< .0001	0.3	< .0001
179	Mental and behavioural disorders due to psychoactive substance use	F11-F19	**4.7**	**< .0001**	8.4	< .0001	2.5	< .0001	1.5	< .0001	0.2	< .0001	7	2.7	< .0001	1.0	0.0167	0.6	< .0001	0.4	< .0001
180	Schizophrenia^a^	F20	**3.2**	**< .0001**	1.2	0.5170	1.5	< .0001	1.0	0.8596	0.3	< .0001	3	4.7	< .0001	1.8	< .0001	1.2	< .0001	0.2	< .0001
181	Schizotypal and delusional disorders	F21-F29.	1.3	< .0001	1.1	0.4555	0.9	0.0485	0.9	0.0002	0.8	< .0001	9	2.6	< .0001	1.0	0.1019	0.7	< .0001	0.4	< .0001
182	Bipolar affective disorder^a^	F30-F31	**0.5**	**< .0001**	0.6	0.0083	0.6	< .0001	0.9	0.0001	2.1	< .0001	96	1.0	0.1900	1.1	0.0009	1.1	< .0001	1.0	0.1900
183	Depression^a^	F32, F33, F34.1, F06.32	**1.7**	**< .0001**	2.6	< .0001	1.4	< .0001	1.3	< .0001	0.6	< .0001	102	1.8	< .0001	1.1	< .0001	0.8	< .0001	0.6	< .0001
184	Mood (affective) disorders	F340, F348-F349, F38-F39	1.1	0.0769	1.0	0.8906	1.0	0.8633	1.1	0.2244	0.9	0.0769	43	1.3	0.0001	1.0	0.8836	1.0	0.7776	0.8	0.0001
185	Phobic anxiety disorders	F40	**1.9**	**< .0001**	1.7	0.0005	1.2	< .0001	1.1	0.0257	0.5	< .0001	44	2.3	< .0001	0.8	< .0001	0.5	< .0001	0.4	< .0001
186	Other anxiety disorders	F41	1.4	< .0001	1.4	0.0027	1.0	0.2171	1.1	0.0009	0.7	< .0001	63	1.6	< .0001	1.0	0.1153	0.8	< .0001	0.6	< .0001
187	Obsessive compulsive disorder (OCD)^a^	F42	0.8	0.0008	0.7	0.0474	0.8	0.0002	0.9	0.1685	1.2	0.0008	134	1.2	0.0005	1.0	0.1154	0.9	0.0005	0.8	0.0005
188	Post-traumatic stress disorder	F431	**2.4**	**< .0001**	2.0	0.0028	1.4	< .0001	1.2	< .0001	0.4	< .0001	99	4.8	< .0001	0.9	0.0002	0.4	< .0001	0.2	< .0001
189	Reactions to severe stress and adjustment disorders	F432-F439	1.8	< .0001	2.2	< .0001	1.4	< .0001	1.3	< .0001	0.6	< .0001	69	1.6	< .0001	0.9	< .0001	0.8	< .0001	0.6	< .0001
190	Dissociative (conversion) disorders, somatoform disorders and other neurotic disorders	F44, F45, F48	1.4	< .0001	0.8	0.2281	1.1	0.0005	1.1	0.2081	0.7	< .0001	82	1.7	< .0001	1.0	0.8092	0.8	< .0001	0.6	< .0001
191	Eating disorders	F50	0.9	0.0425	0.9	0.5827	1.0	0.8915	1.1	0.1558	1.2	0.0425	193	1.5	< .0001	0.9	< .0001	0.7	< .0001	0.7	< .0001
192	Behavioural syndromes associated with physiological disturbances and physical factors	F51-F59	**0.5**	**< .0001**	0.4	0.0056	0.6	< .0001	0.9	0.0430	1.9	< .0001	212	0.8	0.0003	1.1	0.0740	1.1	0.2228	1.2	0.0003
193	Emotionally unstable personality disorder	F603	**3.2**	**< .0001**	2.6	< .0001	1.6	< .0001	1.3	< .0001	0.3	< .0001	6	2.4	< .0001	1.1	< .0001	0.8	< .0001	0.4	< .0001
194	Specific personality disorders	F602, F604-F609	**2.1**	**< .0001**	2.4	< .0001	1.3	< .0001	1.2	< .0001	0.5	< .0001	24	2.9	< .0001	1.0	0.0154	0.7	< .0001	0.3	< .0001
195	Disorders of adult personality and behaviour	F61-F69	1.8	< .0001	1.5	0.0187	1.2	0.0005	1.1	0.0908	0.6	< .0001	27	2.7	< .0001	1.0	0.1393	0.7	< .0001	0.4	< .0001
196	Mental retardation	F70-F79	**64.4**	**< .0001**	3.9	0.0240	1.5	0.0167	0.9	0.6150	0.0	< .0001	1	4.2	< .0001	3.7	< .0001	2.5	< .0001	0.2	< .0001
197	Disorders of psychological development	F80-F89	**6.0**	< .0001	3.3	< .0001	1.3	0.0042	0.8	0.0257	0.2	< .0001	2	3.4	< .0001	1.3	< .0001	0.7	< .0001	0.3	< .0001
198	Hyperkinetic disorders (ADHD)^a^	F90	**3.4**	**< .0001**	7.4	< .0001	1.5	< .0001	1.1	0.0827	0.3	< .0001	14	2.9	< .0001	0.8	< .0001	0.5	< .0001	0.3	< .0001
199	Behavioural and emotional disorders with onset usually occurring in childhood and adolescence	F91-F99	1.7	< .0001	2.1	< .0001	1.2	< .0001	1.0	0.3046	0.6	< .0001	15	1.7	< .0001	1.0	0.2735	0.8	< .0001	0.6	< .0001
	Men: Age 16–24 years (reference)																				
	Men: Age 25–34 years		0.0	< .0001	<0.001	0.8363	0.0	< .0001	0.0	< .0001	247.7	< .0001		0.0	< .0001	6.1	< .0001	21.2	< .0001	78.6	< .0001
	Men: Age 35–44 years		0.0	0.1682	<0.001	0.9403	0.0	< .0001	0.0	< .0001	291.9	< .0001		0.0	< .0001	10.0	< .0001	48.4	< .0001	422.7	< .0001
	Men: Age 45–54 years		0.0	0.0225	<0.001	0.9873	0.0	< .0001	0.0	< .0001	193.7	< .0001		0.0	< .0001	11.5	< .0001	52.6	< .0001	529.0	< .0001
	Men: Age 55–64 years		0.0	0.2680	<0.001	0.9395	0.0	< .0001	0.0	< .0001	204.1	< .0001		0.0	< .0001	17.9	< .0001	59.1	< .0001	570.1	< .0001
	Men: Age 65–74 years		0.0	< .0001	<0.001	0.8895	0.0	< .0001	0.0	< .0001	147.4	< .0001		0.0	< .0001	11.5	< .0001	18.7	< .0001	138.2	< .0001
	Men: Age 75+ years		0.0	0.4099	<0.001	0.9232	0.0	< .0001	0.0	< .0001	98.1	< .0001		0.0	< .0001	11.2	< .0001	14.1	< .0001	80.9	< .0001
	Women: Age 16–24 years		0.5	< .0001	0.8	0.5319	0.9	< .0001	1.2	< .0001	1.9	< .0001		7.1	< .0001	1.0	< .0001	0.4	< .0001	0.1	< .0001
	Women: Age 25–34 years		0.0	< .0001	<0.001	0.8431	0.0	< .0001	0.1	< .0001	454.7	< .0001		0.0	< .0001	8.3	< .0001	24.8	< .0001	54.5	< .0001
	Women: Age 35–44 years		0.0	< .0001	<0.001	0.9157	0.0	< .0001	0.1	< .0001	444.0	< .0001		0.0	< .0001	16.0	< .0001	85.6	< .0001	473.7	< .0001
	Women: Age 45–54 years		0.0	< .0001	<0.001	0.9522	0.0	< .0001	0.1	< .0001	189.0	< .0001		0.0	< .0001	16.6	< .0001	74.4	< .0001	440.0	< .0001
	Women: Age 55–64 years		0.0	0.3103	<0.001	0.9662	0.0	< .0001	0.1	< .0001	96.7	< .0001		0.0	< .0001	17.6	< .0001	47.0	< .0001	248.1	< .0001
	Women: Age 65–74 years		0.0	0.0216	<0.001	0.9243	0.0	0.1548	0.1	< .0001	41.4	< .0001		0.0	< .0001	5.9	< .0001	6.9	< .0001	26.9	< .0001
	Women: Age 75+ years		0.1	0.0004	<0.001	0.9589	0.0	< .0001	0.2	< .0001	13.7	< .0001		0.0	< .0001	10.4	< .0001	8.4	< .0001	25.7	< .0001
	**Model tests**																				
	AIC: Intercept		1,533,641.2		772,558.4		1,867,270.7		1,238,743.3		1,533,641.2			3,098,250.7		3,098,231.3		3,098,232.7		3,098,250.7	
	AIC: Intercept and covariates		1,210,419.8		48,445.7		1,727,374.0		1,171,627.5		1,210,419.8			1,758,718.9		2,558,737.2		2,140,850.4		1,758,718.9	
	Testing Global Null Hypothesis: BETA = 0: Wald test		166,135.2		38,977.1	< .0001	67,201.0	< .0001	54,635.1	< .0001	166135.240	< .0001		468,963.5	< .0001	391,076.6	< .0001	486,672.1	< .0001	468,963.5	< .0001
	Convergence criterion (GCONV = 1E-8) satisfied:		yes		Yes		Yes		Yes		Yes			yes		Yes		Yes		Yes	
	N		1,449,842		564,980		2,323,886		978,506		1,449,842			2,234,914		2,234,900		2,234,901		2,234,914	

ICD-10 International Statistical Classification of Diseases, 10^th^ Revision

^c^ = complex defined conditions; see reference for further details [55]. p. = p-value.

Conditions no. marked ‘A’, overlap with other conditions and are thus not counted twice [55].

### Compliance with ethical standards

Declaration and approval to conduct the study were obtained from the Danish Data Protection Agency and the Secretariat for Research Processing Records, Data and Development Support, Region Zealand (REG-142-2021). No informed consent was required. Statistics Denmark anonymized all register-data before the data were made available on their server.

The conditions were ranked according to their proportion of patients with no educational achievement. The higher ranking, the higher proportion of patients with no educational achievement within the disease. Direct standardisation of age and sex from the national average of age and sex on 1 January 2013 was included to identify differences free of primary effects [85, 86].

In summary, the prevalence rates for the disease groups and chronic conditions were stratified and presented by educational levels, sex and age (Tables 2 and 3 and S1 Table in S1 File), education levels, income quartiles (Table 4 and S2 Table in S1 File), and socioeconomic positions (S3 Table in S1 File). A ratio between disease prevalence for no and high educational levels was calculated to identify inequalities within and between conditions (Tables 2 and 3, S1, S2 and S4 Table in S1 File). Ranked conditions were used to identify clusters and groups of conditions with the lowest educational levels (Table 4 and S2 Table in S1 File). Moreover, mean and ratios were presented for chronic conditions (Table 2) and income (Table 4) with standard deviations. Standardised prevalence estimates are presented in brackets where applicable. Finally, to provide the reader with an overview of highlights, the analysis and commenting will be focussed on all the 14 overall disease groups of the 199 chronic conditions, 29 specific common conditions including overweight, regularly measured in the National Population Health Surveys every four years [87] among others; and the 50 specific chronic conditions with the highest proportions of patients with no educational achievement. However, readers can still look up other estimates of the total 199 presented chronic conditions within the tables.

Data analysis and management were carried out using SAS 9.4 at Statistics Denmark’s research servers. Means and standard deviations were calculated using SAS “proc means”, frequencies were calculated based on the “proc tabulate” function, and logistic regression was calculated using the SAS “proc logistic” function.

## Results

The following descriptions provide an overview of the 14 overall disease groups, on the 29 most common conditions as well as overweight, and the 50 conditions with the highest proportion of patients with no educational achievement.

Table 2 shows the prevalence of the 14 overall disease groups and five common medicines associated with educational achievement, sex, and age groups for patients with no educational achievement. The prevalence of having one or more chronic conditions for patients with no educational achievement was 768 per thousand compared to 601.3 for patients with higher educational achievement. This is equivalent to an overall educational ratio of 1.3, i.e., indicating that patients with no educational achievement are 1.3 times more likely to have a chronic condition than people with high educational achievement. Women with no educational achievement had a higher prevalence (272.6 per thousand) of one or more chronic conditions than males with no educational achievement (188.2 per thousand). Increasing age for patients with no educational achievement also increased the risk of having one or more chronic conditions, ranging from 582.0 (age 16–44), 819.5 (age 45–74) to 954.6 per thousand for 75+ years old. The largest overall educational differences were found among patients using antipsychotic medicine with a ratio of 4.3. Other large differences were found for disease group F (mental and behavioural disorders; ratio 2.5), disease group E (endocrine, nutritional and metabolic diseases; ratio 2.4), disease group I (diseases of the circulatory system; ratio 2.1), and disease group K (diseases of the digestive system; ratio 2.1).

Table 3 provides the prevalence for 29 common, highly prevalent diseases and overweight (see S1 and S4 Tables in S1 File for all conditions) by no and high educational achievement, sex, and age groups for people with no education (see S1 and S4 Tables in S1 File for all 199 conditions). The collective conditions account for a total disease burden of 563 per thousand having one or more chronic conditions. The highest educational differences are found among mental conditions like schizophrenia (ratio 5.9), hyperkinetic disorders (ratio 5.2), dementia (ratio 4.9), but also osteoporosis (ratio 3.9), type 2 diabetes (ratio 3.8), COPD (ratio 3.3), and heart conditions and stroke (ratios ranging from 2.3–3.1). The lowest educational differences are found in respiratory allergy (ratio 0.8), asthma (ratio 0.7), and type 1 diabetes (ratio 1.1) and rheumatoid arthritis (ratio 1.1). The sex and age trends indicate a higher disease prevalence among women and increasing prevalence with increasing age, except for type 1 diabetes, schizophrenia, and hyperkinetic disorders. Moreover, schizophrenia, hyperkinetic disorders, and type 1 diabetes also showed decreasing prevalence rates with increasing age, contrary to the general trend of most other conditions.

**Fig 1:**
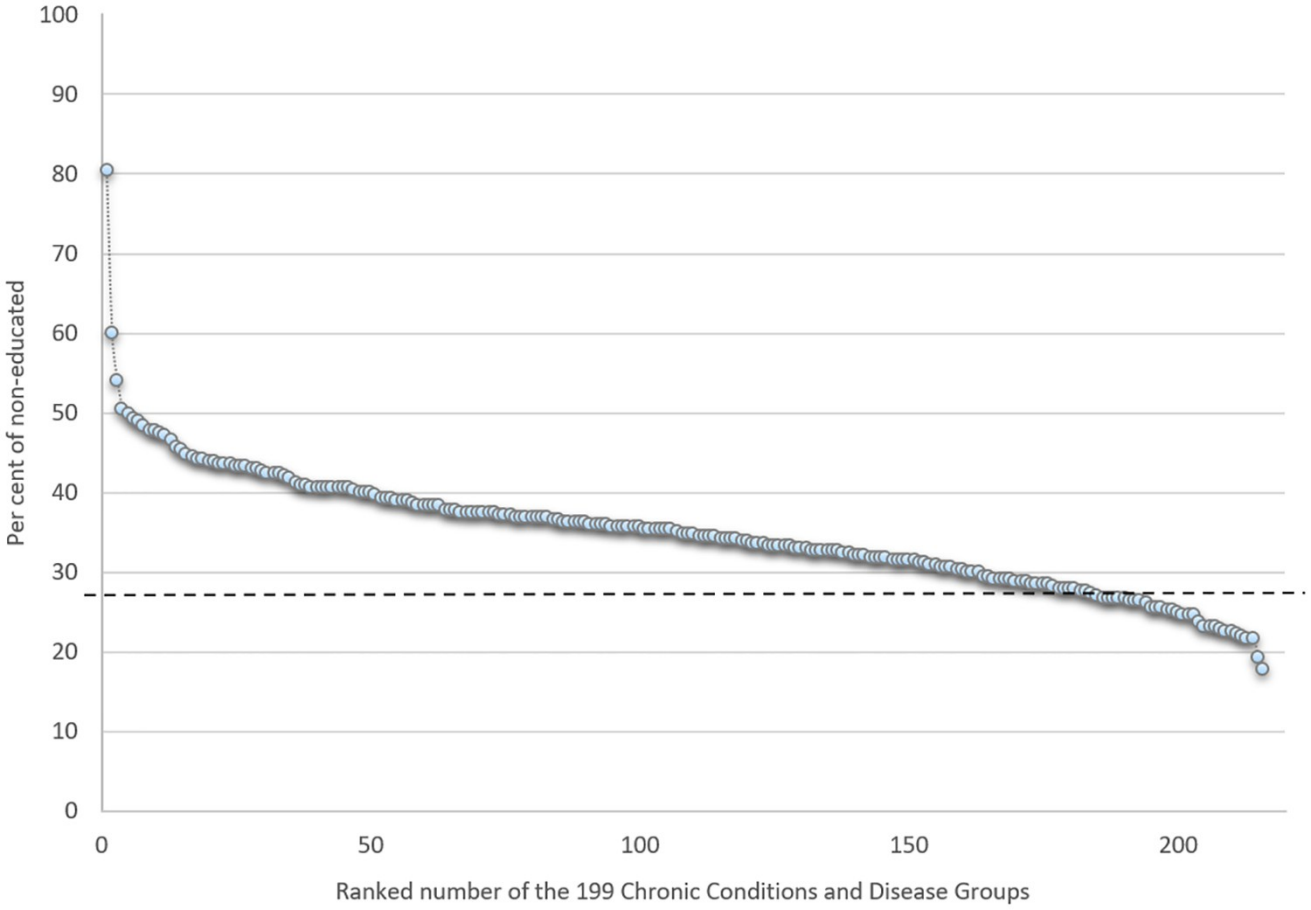
Per cent of people with no education within the 199 chronic conditions and disease groups ranked by descending proportion. The dashed line is the national average of 29.0 per cent.

Table 4 displays the prevalence (per cent within conditions) of the five educational levels, income quartiles, and the mean income for all the 199 chronic conditions. However, the following exclusively comments on the 50 conditions with the highest proportion of patients with no educational achievement ordered in ICD-10 overall disease groups (S2 Table in S1 File comprising only the 50 conditions sorted). **Mental conditions (F)** had the overall highest proportion of patients with no educational achievement with 15 conditions out of the 50 conditions. This included eight conditions in the top 10 of conditions with the highest proportion of patients with no educational achievement: mental retardation (F70-F79), disorders of psychological development (F80-F89), schizophrenia (F20), dementia (F00, G30, and other disease codes for dementia), emotionally unstable personality disorder (F603), mental and behavioural disorders due to psychoactive substance use (F11-F19), organic, incl. symptomatic, mental disorders (F04-F09), and schizotypal and delusional disorders (F21-F29). **Diseases of the respiratory system (J)** had the overall second-highest proportion of patients with no educational achievement with one condition in the top 10 and three conditions in the top 50: Bronchitis (F40-42), Emphysema (J43), and COPD (J44, J96, J13-J18). **Diseases of the circulatory system (I)** had the overall third-highest proportion of patients with no educational achievement. This included 12 conditions: atherosclerosis (I70), other peripheral vascular diseases (I73), sequelae of cerebrovascular disease (I69), aortic and mitral valve disease (I05, I06, I134, I135), heart failure (I11, I13, I13.2, I142, I142.6, I142.7, I142.9, I150, I150.1, I150.9), chronic ischaemic heart disease (I25), stroke (I60, I61, I63-64, Z501), acute myocardial infarction (I21-I22), ischaemic heart diseases (I20-I25), atrioventricular and left bundle branch block (I44), AMI complex/other (I23-I24), pulmonary heart disease and diseases of pulmonary circulation (I26-I28), and atrial fibrillation and flutter (I48). **Diseases of the nervous system (G)** had the overall fourth highest proportion of patients with no educational achievement and included four conditions: cerebral palsy and other paralytic syndromes (G80–G83), Parkinson’s disease (G20, G21, G22, F02.3), epilepsy (G40–G41), and systemic atrophies primarily affecting the central nervous system (G10–G14, G30–G32). Chronic viral hepatitis (B18) was ranked number five. **Cancers (C)** and **In situ and benign neoplasms (D)** had the overall sixth highest proportion of patients with no educational achievement. This included three conditions: other anaemias (D64), malignant neoplasms of bronchus and lung (C43), and aplastic and other anaemias (D60-D63). **Diseases of the eye and ear (H)** had the seventh highest proportion of patients with no educational achievement. This included four conditions: presbycusis (H911), disease of the eye lens (H25-H28), blindness and partial sight (H54), and other retinal disorders (H35). **Diseases of the musculoskeletal system and connective tissue (M)** had the eighth highest proportion of patients with no educational achievement and comprised four conditions: coxarthrosis (M16), rheumatism (M790), osteoporosis in disease classified elsewhere (M82), and polyarthrotis (M15). Finally, chronic renal failure (N18) and type 2 diabetes (E11) had the ninth and tenth highest proportion of patients with no educational achievement. Notably, the above-mentioned specific conditions do overlap in ranks and can therefore not be entirely categorised into ten discrete groups, although this broadly reflects the groups’ average ranks. Income inequalities across the conditions broadly follow those of education, although differences exist. Readers are encouraged to explore this further for conditions of interest. The differences in the disease prevalence for people with no educational achievement within diseases ranged from 17.8–80.3 per cent. Hereof, many chronic conditions occurred between 30 and 40 per cent (Fig 1). In total, 153 conditions of the 199 chronic conditions had more patients with no education than the national average of 29.0. An extract of Table 4 with solely the 50 chronic conditions sorted by the highest proportion of patients with no educational achievement can be found in S2 Table in S1 File. The table also includes the ratio between prevalence for high and no educational levels and per cent proportions of high and low levels of education. Finally, an overview of the socioeconomic positions can be found in S3 Table in S1 File.

Table 5 presents an off the shelf catalogue of odds ratios (OR) from nine logistic regression models between the five educational levels and the income quartiles and all the 199 chronic conditions, gender and age to control for potential confounding and residual correlations of the crude estimates. However, the following exclusively comments the regression model for no educational achievement vs high educational achievement and overall tendencies for the conditions based on their disease groups. Overall, **Mental and behavioural disorders (F)** and **Diseases of the musculoskeletal system and connective tissue (M**) have the highest level of social disparity. For the mental conditions, significant, high OR ranging from 2.1.-6.0 (excluding mental retardation) for conditions like mental and behavioural disorders due to psychoactive substance use, schizophrenia, post-traumatic stress disorder, emotionally unstable personality disorder, and hyperkinetic disorders (ADHD). For **musculoskeletal diseases**, significant high OR ranging from 1.7–4.0 were found for conditions like fibromyalgia, rheumatism unspecified, myalgia, shoulder lesions, and spinal osteochondrosis. Interesting, significant decreasing OR were found for a common condition, rheumatoid arthritis (OR = 0.7) and enthesopathies of lower limb (OR = 0.5). For **Diseases of the nervous system (G)** and **Diseases of the eye and ear (H)**, some high significant OR were found within the range of 1.7–2.8 for specific conditions like corneal scars and opacities, epilepsy, mononeuropathies of upper limb, cerebral palsy and other paralytic syndromes, and other disease of the inner ear. **Diseases of the circulatory system (I)**, **Diseases of the respiratory system (J)**, and **Disease of the digestive system (K)** displayed higher significant OR of 1.6–1.9 for chronic conditions like ventral hernia, ulcers, atherosclerosis, bronchitis, emphysema, and chronic obstructive disease (COPD). Particularly disease groups I and H relatively many had high, significant, decreasing OR ranging from 0.5–0.7, indicating significant correlation with higher educational achievement. **Disease group (B)**, and **endocrine disorders (E)** show high significant OR for patients with Chronic viral hepatitis with odds ratio at 3.7 and diabetes type 2 with odds ratio at 1.9. Finally, **Cancers (C)** was the disease group with the lowest OR and highest number of decreasing OR, i.e., cancers displayed the reversed social disparity as patients with high educational achievement have higher risk of getting e.g., malignant neoplasm of skin, other malignant neoplasms of skin, malignant neoplasms of breast, and malignant neoplasm of prostate.

## Discussion

From present findings, the overall disease prevalence is 768 per thousand for people with no educational achievement compared to 601.3 for people with higher education (ratio 1.3), which suggests that overall people with no educational achievement have a 30 per cent higher disease burden. Among patients with no educational achievement, women had higher disease prevalence than men, and the prevalence increased with age for most conditions, although variations were found across conditions. These results are comparable to those of other studies, which also find a higher disease burden for patients with no educational achievement, and a higher prevalence of women among patients with no educational achievement [24, 45]. Across disease groups, the largest educational differences were found within disease group F–Mental and behavioural disorders (ratio 2.5), E–Endocrine, nutritional and metabolic diseases (ratio 2.4), I–Diseases of the circulate system (ratio 2.1), and K–Diseases of the digestive system (ratio 2.1). Among specific chronic conditions, schizophrenia had the largest educational differences (ratio 5.9) followed by hyperkinetic disorders (ratio 5.2), dementia (ratio 4.9), osteoporosis (ratio 3.9), type 2 diabetes (ratio 3.8), COPD (ratio 3.3), heart conditions and stroke (ratios ranging from 2.3–3.1). The logistic regression estimates enable readers and health professionals to identify any confounding and residual correlations of the crude estimates for social disparities for their conditions of interests—adjusted not only of gender and age, but also an unprecedented total of all 199 chronic conditions. Findings on socioeconomic differences showed that showed that many of the crude estimate tendencies where also confirmed in the adjusted regression analysis, and that particular people with no educational achievement had the highest OR within disease group F–Mental and behavioural disorders and M—Diseases of the musculoskeletal system and connective tissue. Earlier studies have also shown that schizophrenia is often associated with low educational achievement and high unemployment [88, 89]. Low parental socioeconomic and a family history of mental disorders are in the literature two well establish risk factors of schizophrenia [90, 91]. However, a large Danish study showed that even though parental socioeconomic and a family history of mental disorders contribute to the development of schizophrenia, they made a very limited difference on a person with schizophrenias ability to be employed and get an education [88].

Furthermore, present findings showed an increasing prevalence of people with no educational achievement for every number of chronic diseases they have more than one. People living with multimorbidity have to follow a trajectory for each of the single diseases they suffer from that is a potential driver for a high treatment burden [92]. Additionally, socioeconomic factors such as low educational achievement is associated with high treatment burden [25, 92, 93]. Treatment burden is all the aspects of treatment that a patient experience regarding manage of their disease(s) and the impact this has on the patient’s wellbeing [92, 94]. The increasing number of people living with multimorbidity is an advancing public health problem [24, 94]. This underlines the importance of minimizing the treatment burden for patients with multimorbidity [25]. Especially, because a high treatment burden may lead to involuntary non-adherence, adverse health outcomes and increasing health care cost [92, 93].

### Strengths

One major strength of the current study is the size and high quality of the data. A full nationwide register-based recorded dataset for chronic conditions and medical treatments including more than 4.5 million people. The high number of recorded chronic conditions is also a major strength. Moreover, the study is unique in terms of the big data linkage of seven different high-quality registers, combining many chronic conditions with three different socioeconomic measurements. This approach using uniform methodology within a single study has provided comparable socioeconomic data of 199 chronic conditions, and thus the potential to identify treatment potentials, optimising treatments by differentiating and targeting patients accordingly to socioeconomic differences.

### Limitations

At least three main limitations exist. First, there are *register-based issues* of report identification and data misclassification. One fundamental limitation of register studies is not identifying conditions not treated in the healthcare system, self-treatments, as it is not reported within registers, leading to underestimates (see detailed evaluation in previous studies [55, 56, 95]. On the other hand, being treated for a chronic condition is not necessarily the same as being actually or severely ill. There may be instances of defensive medicine, patients being treated on suspicion of a chronic condition, or even over-diagnosis, leading to the identification and overestimates from registers [56]. Moreover, data misclassification is also a source of bias due to different coding practices between hospitals [61], clinical disagreements, dissimilar clinical and administrative practices, and interpretation of the ICD-10 criteria [96]. These issues would naturally affect our estimates of socioeconomic disparities. However, earlier studies have not found evidence of systematic misclassification; reported diagnoses of psychiatric and somatic conditions have been validated with good results [95, 97–102].

Second, high ratios, i.e., large differences in disease prevalence by thousandth between high and low educational achievements for a disease, do not necessarily, although often, comprise conditions that have the overall lowest proportions of people with low educational achievement, as seen in Table 3, S1, S4 Tables in S1 File. As conditions with the lowest educational levels are of interest to healthcare professionals, this poses a challenge. On the other hand, conditions with the lowest proportions of people with no educational achievement might not have a potential for improvement, as there might not be any real differences between high and no educational achievement prevalence (e.g., low ratio). Thus, the ratio was used to measure real-world treatment potential. However, we urge readers to use both the ratio and proportions of educational achievements when identifying and comparing conditions of interest.

Third, practical difficulties in presenting an overview of the results due to the number of socioeconomic measurements and diseases. While the catalogue is easily accessible for a few or single conditions for use in specific treatments, it is difficult to maintain an overview of differences across all the conditions. This might pose an issue for the practical use by decision-makers in prioritising and comparing a larger number of conditions.

### Future studies

There is no gold standard for socioeconomic measurements. In this study, we mainly relayed in educational achievements, and future studies should aim to combine educational achievement, income, and job status to get a more accurate socioeconomic measure. This is especially challenging when handling a large chunk of data like this study. Therefore, future studies also need to consider how to handle and compare large chunks of data and conditions.

## Conclusions

The present study provides a catalogue of diseases prevalence associated with socioeconomic differences for 199 different clinical-reported chronic conditions and conditions by sex, age, education, income, and socioeconomic position, based on a total nationwide population. To the best of the authors’ knowledge, the study provides the most comprehensive, comparable descriptive register study and catalogue of chronic conditions’ socioeconomic prevalence, characteristics, and differences.

## Supporting information

S1 File (DOCX)
