# Supplementary material for: Catalogue of socioeconomic disparities and characteristics of 199+ chronic conditions—A nationwide register-based population study
Source: PLoS One. 2022 Dec 30;17(12):e0278380. doi: 10.1371/journal.pone.0278380 (PMC9803180; doi:10.1371/journal.pone.0278380)
Supplement: S1 File — (DOCX) [file pone.0278380.s001.docx]

***S1*** ***Table.*** Catalogue of disease prevalence: number of patients, prevalence rates per thousand, educational, gender and age estimates of 199+ chronic conditions and disease groups in Denmark, on 1 January 2013.

|  |  |  | **N and Prevalence** | | | | | | | | | | | | | | | | | | | |
| --- | --- | --- | --- | --- | --- | --- | --- | --- | --- | --- | --- | --- | --- | --- | --- | --- | --- | --- | --- | --- | --- | --- |
| **No.** | **Name of condition** | **ICD-10 code / definition** | **Total population*** | | | **Disease prevalence by education** | | | | | | | **Disease prevalence by gender and age**  **- within no education or training** | | | | | | | | | |
|  |  |  |  |  |  | No education  or training | | | Higher (MSc degree or doctorate) | | |  | Female | | Male | | Age 16-44 | | Age 45-74 | | Age 75+ | |
|  |  |  | N | Per thousand | | N | Per thousand | | N | Per Thousand | | *Ratio* | Per thousand | | | | Per thousand | | | | | |
|  | **B – Viral hepatitis and human immunodeficiency virus [HIV] disease** | **B18, B20–B24** | **8,500** | **1.9** | **(1.9)** | **3,091** | **2.7** | **(3.1)** | **488** | **1.5** | **(0.8)** | ***1.8*** | **1.9** |  | **3.6** |  | **3.7** |  | **3.0** |  | **0.2** |  |
| 1 | Chronic viral hepatitis | B18 | 4,584 | 1.0 | (1.0) | 2,133 | 1.9 | (2.1) | 160 | 0.5 | (0.1) | *3.8* | 1.4 |  | 2.5 |  | 2.7 |  | 2.1 |  | 0.1 |  |
| 2 | Human immunodeficiency virus [HIV] disease | B20–24 | 4,229 | 0.9 | (0.9) | 1,076 | 1.0 | (1.1) | 346 | 1.1 | (0.8) | *0.9* | 0.6 |  | 1.3 |  | 1.2 |  | 1.1 |  | 0.1 |  |
|  | **C – Malignant neoplasms** | **C00–C99; D32–D33; D35.2–D35.4; D42–D44** | **229,331** | **50.3** | **(50.4)** | **76,501** | **67.8** | **(48.5)** | **13,755** | **42.8** | **(52.8)** | ***1.6*** | **79.2** |  | **55.2** |  | **10.0** |  | **76.5** |  | **145.1** |  |
| 3 | Malignant neoplasms of other and unspecified localizations | C00–C14; C30–C33; C37–C42; C45–C49; C69; C73–74; C754–C759 | 20,557 | 4.5 | (4.6) | 6,702 | 5.9 | (4.9) | 1,209 | 3.8 | (4.1) | *1.6* | 5.6 |  | 6.3 |  | 1.2 |  | 7.6 |  | 9.6 |  |
| 4 | Malignant neoplasms of digestive organs | C15–C17; C22–C26 | 4,839 | 1.1 | (1.1) | 1,825 | 1.6 | (1.2) | 213 | 0.7 | (0.8) | *2.4* | 1.4 |  | 1.8 |  | 0.2 |  | 1.9 |  | 3.3 |  |
| 5 | Malignant neoplasm of colon | C18 | 18,826 | 4.1 | (4.1) | 7,233 | 6.4 | (4.0) | 936 | 2.9 | (4.2) | *2.2* | 7.5 |  | 5.2 |  | 0.2 |  | 5.7 |  | 19.2 |  |
| 6 | Malignant neoplasms of rectosigmoid junction, rectum, anus and anal canal | C19–C21 | 10,680 | 2.3 | (2.3) | 4,011 | 3.6 | (2.5) | 531 | 1.7 | (2.2) | *2.2* | 3.5 |  | 3.7 |  | 0.1 |  | 3.7 |  | 9.3 |  |
| 7 | Malignant neoplasm of bronchus and lung | C34 | 14,762 | 3.2 | (3.3) | 6,254 | 5.5 | (4.2) | 482 | 1.5 | (2.1) | *3.7* | 6.1 |  | 4.9 |  | 0.3 |  | 7.0 |  | 10.7 |  |
| 8 | Malignant melanoma of skin | C43 | 19,636 | 4.3 | (4.3) | 4,515 | 4.0 | (3.1) | 1,778 | 5.5 | (5.8) | *0.7* | 4.7 |  | 3.2 |  | 1.2 |  | 4.5 |  | 7.4 |  |
| 9 | Other malignant neoplasms of skin | C44 | 15,597 | 3.4 | (3.4) | 5,209 | 4.6 | (2.8) | 1,025 | 3.2 | (4.1) | *1.4* | 4.8 |  | 4.4 |  | 0.2 |  | 3.9 |  | 14.4 |  |
| 10 | Malignant neoplasm of breast | C50 | 50,687 | 11.1 | (11.1) | 17,376 | 15.4 | (9.4) | 2,426 | 7.6 | (12.4) | *2.0* | 29.1 |  | 0.2 |  | 0.6 |  | 19.7 |  | 29.5 |  |
| 11 | Malignant neoplasms of female genital organs | C51–C52; C56–C58 | 7,245 | 1.6 | (1.6) | 2,671 | 2.4 | (1.7) | 352 | 1.1 | (1.6) | *2.2* | 4.5 |  | 0.0 |  | 0.3 |  | 2.9 |  | 4.4 |  |
| 12 | Malignant neoplasm of cervix uteri, corpus uteri and part unspecified | C53–C55 | 11,608 | 2.5 | (2.5) | 4,350 | 3.9 | (2.7) | 494 | 1.5 | (2.4) | *2.5* | 7.3 |  | 0.0 |  | 0.7 |  | 4.4 |  | 7.9 |  |
| 13 | Malignant tumour of male genitalia | C60, C62–C63 | 5,194 | 1.1 | (1.1) | 1,116 | 1.0 | (1.1) | 535 | 1.7 | (1.3) | *0.6* | 0.0 |  | 2.1 |  | 1.3 |  | 1.0 |  | 0.5 |  |
| 14 | Malignant neoplasm of prostate | C61 | 26,697 | 5.9 | (5.9) | 8,146 | 7.2 | (5.1) | 2,206 | 6.9 | (7.1) | *1.1* | 0.0 |  | 15.2 |  | 0.0 |  | 7.1 |  | 20.2 |  |
| 15 | Malignant neoplasms of urinary tract | C64–C68 | 10,319 | 2.3 | (2.3) | 3,962 | 3.5 | (2.6) | 476 | 1.5 | (1.8) | *2.4* | 2.4 |  | 4.7 |  | 0.2 |  | 3.6 |  | 9.0 |  |
| 16 | Brain cancer ^c^ | C71, C75.1–C75.3, D33.0–D33.2, D35.2–D35.4, D43.0–D43.2, D44.3–D44.5 (brain). C70, D32, D42 (brain membrane). C72, D33.3–D33.9, D43.3–D43.9 (cranial nerve, spinal cord) | 15,310 | 3.4 | (3.4) | 4,860 | 4.3 | (3.7) | 957 | 3.0 | (3.1) | *1.4* | 4.1 |  | 4.5 |  | 2.0 |  | 5.3 |  | 5.7 |  |
| 17 | Malignant neoplasms of ill–defined, secondary and unspecified sites, and of independent (primary) multiple sites | C76–C80, C97 | 25,619 | 5.6 | (5.6) | 8,737 | 7.7 | (5.9) | 1,429 | 4.4 | (5.4) | *1.7* | 9.1 |  | 6.3 |  | 1.2 |  | 9.7 |  | 13.8 |  |
| 18 | Malignant neoplasms, stated or presumed to be primary, of lymphoid, haematopoietic and related tissue | C81–C96 | 19,712 | 4.3 | (4.3) | 6,521 | 5.8 | (4.4) | 1,276 | 4.0 | (4.6) | *1.5* | 5.8 |  | 5.8 |  | 1.7 |  | 6.1 |  | 11.9 |  |
|  | **D – In situ and benign neoplasms, and neoplasms of uncertain or unknown behaviour and diseases of the blood and blood–forming organs and certain disorders involving the immune mechanism** | **D00–D09; D55–D59; D60–D67; D80–D89** | **116,560** | **25.6** | **(25.7)** | **40,941** | **36.3** | **(29.6)** | **6,187** | **19.3** | **(21.5)** | ***1.9*** | **45.5** |  | **26.0** |  | **16.4** |  | **33.0** |  | **80.3** |  |
| 19 | In situ neoplasms | D00–D09 | 19,810 | 4.3 | (4.4) | 5,309 | 4.7 | (4.0) | 1,377 | 4.3 | (4.4) | *1.1* | 7.1 |  | 2.0 |  | 2.2 |  | 5.6 |  | 6.8 |  |
| 20 | Haemolytic anaemias | D55–D59 | 3,055 | 0.7 | (0.7) | 995 | 0.9 | (0.9) | 195 | 0.6 | (0.6) | *1.5* | 1.2 |  | 0.6 |  | 1.0 |  | 0.7 |  | 1.2 |  |
| 21 | Aplastic and other anaemias | D60–D63 | 14,918 | 3.3 | (3.3) | 6,047 | 5.4 | (3.9) | 614 | 1.9 | (2.5) | *2.8* | 6.5 |  | 4.1 |  | 1.3 |  | 4.4 |  | 15.0 |  |
| 22 | Other anaemias | D64 | 46,613 | 10.2 | (10.3) | 20,597 | 18.3 | (13.1) | 1,478 | 4.6 | (7.0) | *4.0* | 23.0 |  | 13.0 |  | 4.2 |  | 13.9 |  | 55.0 |  |
| 23 | Coagulation defects, purpura and other haemorrhagic conditions | D65–D69 | 25,376 | 5.6 | (5.6) | 6,842 | 6.1 | (6.1) | 1,912 | 6.0 | (5.5) | *1.0* | 7.1 |  | 4.9 |  | 5.7 |  | 6.1 |  | 6.6 |  |
| 24 | Other diseases of blood and blood–forming organs | D70–D77 | 8,896 | 2.0 | (2.0) | 3,095 | 2.7 | (2.4) | 457 | 1.4 | (1.5) | *1.9* | 3.1 |  | 2.3 |  | 1.5 |  | 3.2 |  | 3.6 |  |
| 25 | Certain disorders involving the immune mechanism | D80–D89 | 7,660 | 1.7 | (1.7) | 2,123 | 1.9 | (1.9) | 560 | 1.7 | (1.5) | *1.1* | 1.9 |  | 1.9 |  | 1.7 |  | 2.1 |  | 1.4 |  |
|  | **E – Endocrine, nutritional and metabolic diseases** | **E00–E14; E20–E29; E31–35; E70–E78; E84–E85; E88–E89** | **877,433** | **192.6** | **(192.7)** | **323,816** | **286.9** | **(225.8)** | **38,834** | **120.9** | **(146.8)** | ***2.4*** | **332.0** |  | **236.9** |  | **56.4** |  | **348.8** |  | **519.9** |  |
| 26 | Diseases of the thyroid ^c^ | E00–E04, E06, E07 | 131,908 | 29.0 | (29.0) | 44,216 | 39.2 | (30.7) | 7,002 | 21.8 | (27.1) | *1.8* | 64.1 |  | 11.5 |  | 11.8 |  | 45.3 |  | 70.2 |  |
| 27 | Thyrotoxicosis ^c^ | E05 | 41,374 | 9.1 | (9.0) | 16,176 | 14.3 | (11.4) | 1,879 | 5.8 | (7.4) | *2.5* | 23.2 |  | 4.5 |  | 3.9 |  | 14.4 |  | 32.5 |  |
| 28 | Diabetes type 1 ^c^ | E10 | 23,062 | 5.1 | (5.1) | 6,012 | 5.3 | (5.5) | 1,541 | 4.8 | (4.4) | *1.1* | 4.5 |  | 6.2 |  | 6.1 |  | 5.5 |  | 3.4 |  |
| 29 | Diabetes type 2 ^c^ | E11 | 242,177 | 53.2 | (53.3) | 102,366 | 90.7 | (73.6) | 7,735 | 24.1 | (29.1) | *3.8* | 93.9 |  | 87.2 |  | 14.1 |  | 112.7 |  | 164.1 |  |
| 30 | Diabetes others ^c^ | E12–E14 | 1,117 | 0.2 | (0.2) | 386 | 0.3 | (0.3) | 52 | 0.2 | (0.2) | *2.1* | 0.4 |  | 0.3 |  | 0.2 |  | 0.4 |  | 0.5 |  |
| 31 | Disorders of other endocrine glands | E20–E35, except E30 | 28,650 | 6.3 | (6.4) | 8,276 | 7.3 | (7.3) | 1,815 | 5.6 | (5.5) | *1.3* | 9.8 |  | 4.6 |  | 7.7 |  | 6.5 |  | 9.2 |  |
| 32 | Metabolic disorders | E70–E77; E79–E83; E85, E88–E89; | 23,690 | 5.2 | (5.2) | 7,872 | 7.0 | (6.4) | 1,391 | 4.3 | (4.3) | *1.6* | 8.7 |  | 5.0 |  | 5.4 |  | 7.4 |  | 8.6 |  |
| 33 | Disturbances in lipoprotein circulation and other lipids ^c^ | E78 | 652,242 | 143.2 | (143.1) | 252,468 | 223.7 | (169.4) | 25,487 | 79.3 | (103.7) | *2.8* | 247.2 |  | 197.6 |  | 19.4 |  | 281.6 |  | 421.8 |  |
| 34 | Cystic fibrosis ^c^ | E84 | 947 | 0.2 | (0.2) | 234 | 0.2 | (0.2) | 140 | 0.4 | 0.4) | *0.5* | 0.2 |  | 0.2 |  | 0.4 |  | 0.1 |  | 0.1 |  |
|  | **G – Diseases of the nervous system** | **G00–G14; G20–G32; G35–G37; G40–47; G50–64; G70–73; G80–G83; G90–G99** | **561,054** | **123.2** | **(123.5)** | **186,070** | **164.9** | **(152.2)** | **28,699** | **89.3** | **(87.2)** | ***1.8*** | **192.7** |  | **134.0** |  | **108.2** |  | **192.5** |  | **187.4** |  |
| 35 | Inflammatory diseases of the central nervous system | G00–G09 | 7,642 | 1.7 | (1.7) | 2,292 | 2.0 | (1.9) | 516 | 1.6 | (1.5) | *1.3* | 2.1 |  | 2.0 |  | 1.8 |  | 2.2 |  | 2.0 |  |
| 36 | Systemic atrophies primarily affecting the central nervous system and other degenerative diseases | G10–G14, G30–G32 | 10,401 | 2.3 | (2.3) | 4,214 | 3.7 | (2.5) | 538 | 1.7 | (2.3) | *2.2* | 4.4 |  | 3.0 |  | 0.8 |  | 2.9 |  | 11.2 |  |
| 37 | Parkinson’s disease ^c^ | G20, G21, G22, F02.3 | 57,583 | 12.6 | (12.6) | 27,135 | 24.0 | (20.5) | 1,899 | 5.9 | (6.9) | *4.1* | 27.5 |  | 20.2 |  | 12.3 |  | 25.8 |  | 39.6 |  |
| 38 | Extrapyramidal and movement disorders | G23–G26 | 10,837 | 2.4 | (2.4) | 3,997 | 3.5 | (2.9) | 563 | 1.8 | (2.0) | *2.0* | 4.3 |  | 2.7 |  | 1.9 |  | 3.6 |  | 6.3 |  |
| 39 | Sclerosis | G35 | 13,284 | 2.9 | (2.9) | 3,535 | 3.1 | (3.2) | 925 | 2.9 | (2.5) | *1.1* | 4.2 |  | 2.0 |  | 2.1 |  | 4.4 |  | 1.4 |  |
| 40 | Demyelinating diseases of the central nervous system | G36–G37 | 4,571 | 1.0 | (1.0) | 1,155 | 1.0 | (1.1) | 331 | 1.0 | (0.8) | *1.0* | 1.3 |  | 0.8 |  | 0.9 |  | 1.3 |  | 0.4 |  |
| 41 | Epilepsy ^c^ | G40–G41 | 61,695 | 13.5 | (13.6) | 26,815 | 23.8 | (23.1) | 2,194 | 6.8 | (6.1) | *3.5* | 24.2 |  | 23.3 |  | 24.1 |  | 24.3 |  | 21.8 |  |
| 42 | Migraine ^c^ | G43 | 149,866 | 32.9 | (33.0) | 38,243 | 33.9 | (34.9) | 10,093 | 31.4 | (29.6) | *1.1* | 52.5 |  | 13.2 |  | 27.4 |  | 44.5 |  | 15.6 |  |
| 43 | Other headache syndromes | G44 | 16,469 | 3.6 | (3.6) | 4,690 | 4.2 | (4.6) | 828 | 2.6 | (2.0) | *1.6* | 5.0 |  | 3.2 |  | 5.7 |  | 4.0 |  | 1.9 |  |
| 44 | Transient cerebral ischaemic attacks and related syndromes and vascular syndromes of brain in cerebrovascular diseases | G45–G46 | 43,977 | 9.7 | (9.7) | 16,484 | 14.6 | (10.4) | 2,086 | 6.5 | (8.3) | *2.2* | 15.1 |  | 14.0 |  | 1.6 |  | 15.2 |  | 35.7 |  |
| 45 | Sleep disorders | G47 | 36,806 | 8.1 | (8.1) | 9,770 | 8.7 | (8.5) | 2,229 | 6.9 | (5.7) | *1.2* | 4.9 |  | 12.9 |  | 6.1 |  | 12.2 |  | 3.4 |  |
| 46 | Disorders of trigeminal nerve and facial nerve disorders | G50–G51 | 21,488 | 4.7 | (4.7) | 6,754 | 6.0 | (5.4) | 1,207 | 3.8 | (3.6) | *1.6* | 6.9 |  | 5.0 |  | 3.6 |  | 6.9 |  | 7.4 |  |
| 47 | Disorders of other cranial nerves, cranial nerve disorders in diseases classified elsewhere, nerve root and plexus disorders and nerve root and plexus compressions in diseases classified elsewhere | G52–G55 | 12,429 | 2.7 | (2.7) | 3,930 | 3.5 | (3.2) | 635 | 2.0 | (1.8) | *1.8* | 3.6 |  | 3.3 |  | 1.8 |  | 4.6 |  | 3.3 |  |
| 48 | Mononeuropathies of upper limb | G56 | 122,395 | 26.9 | (26.9) | 43,178 | 38.3 | (35.4) | 3,900 | 12.1 | (11.1) | *3.2* | 48.6 |  | 26.8 |  | 18.8 |  | 50.6 |  | 38.1 |  |
| 49 | Mononeuropathies of lower limb, other mononeuropathies and mononeuropathy in diseases classified elsewhere | G57–G59 | 18,627 | 4.1 | (4.1) | 5,756 | 5.1 | (4.6) | 902 | 2.8 | (2.7) | *1.8* | 5.9 |  | 4.2 |  | 2.4 |  | 6.9 |  | 4.8 |  |
| 50 | Polyneuropathies and other disorders of the peripheral nervous system | G60–G64 | 30,289 | 6.6 | (6.7) | 10,937 | 9.7 | (7.9) | 1,529 | 4.8 | (5.2) | *2.0* | 9.3 |  | 10.2 |  | 2.8 |  | 11.5 |  | 16.7 |  |
| 51 | Diseases of myoneural junction and muscle | G70–G73 | 5,758 | 1.3 | (1.3) | 1,898 | 1.7 | (1.6) | 386 | 1.2 | (1.2) | *1.4* | 1.7 |  | 1.6 |  | 1.6 |  | 1.8 |  | 1.4 |  |
| 52 | Cerebral palsy and other paralytic syndromes | G80–G83 | 14,410 | 3.2 | (3.2) | 6,846 | 6.1 | (6.2) | 540 | 1.7 | (1.4) | *3.6* | 5.5 |  | 6.7 |  | 8.8 |  | 5.3 |  | 3.2 |  |
| 53 | Other disorders of the nervous system | G90–G99 | 44,394 | 9.7 | (9.8) | 14,788 | 13.1 | (12.0) | 2,447 | 7.6 | (7.6) | *1.7* | 13.8 |  | 12.4 |  | 10.1 |  | 14.2 |  | 15.3 |  |
|  | **H – Diseases of the eye and adnexa and diseases of the ear and mastoid process** | **H02–H06; H17–H18; H25–H28; H31–H32; H34–H36; H40–55; H57; H80-H810; H93, H90–H93** | **448,176** | **98.4** | **(98.6)** | **162,019** | **143.6** | **(101.4)** | **24,682** | **76.8** | **(97.1)** | ***1.9*** | **163.1** |  | **121.8** |  | **33.4** |  | **128.5** |  | **379.4** |  |
| 54 | Disorders of eyelid, lacrimal system and orbit | H02–H06 | 13,191 | 2.9 | (2.9) | 4,338 | 3.8 | (3.0) | 736 | 2.3 | (2.7) | *1.7* | 4.8 |  | 2.7 |  | 1.0 |  | 4.3 |  | 7.6 |  |
| 55 | Corneal scars and opacities | H17 | 2,173 | 0.5 | (0.5) | 734 | 0.7 | (0.5) | 100 | 0.3 | (0.3) | *2.1* | 0.6 |  | 0.7 |  | 0.3 |  | 0.7 |  | 1.2 |  |
| 56 | Other disorders of cornea | H18 | 9,473 | 2.1 | (2.1) | 3,073 | 2.7 | (1.9) | 657 | 2.0 | (2.4) | *1.3* | 3.5 |  | 1.9 |  | 1.2 |  | 2.2 |  | 7.0 |  |
| 57 | Diseases of the eye lens (cataracts) | H25–H28 | 68,009 | 14.9 | (15.1) | 29,764 | 26.4 | (16.3) | 2,743 | 8.5 | (14.3) | *3.1* | 34.1 |  | 17.8 |  | 0.8 |  | 20.8 |  | 87.0 |  |
| 58 | Disorders of the choroid and retina | H31–H32 | 1,900 | 0.4 | (0.4) | 582 | 0.5 | (0.4) | 115 | 0.4 | (0.4) | *1.4* | 0.6 |  | 0.5 |  | 0.3 |  | 0.5 |  | 1.0 |  |
| 59 | Retinal vascular occlusions | H34 | 10,358 | 2.3 | (2.3) | 4,051 | 3.6 | (2.3) | 570 | 1.8 | (2.4) | *2.0* | 3.9 |  | 3.3 |  | 0.2 |  | 3.0 |  | 11.2 |  |
| 60 | Other retinal disorders | H35 | 68,485 | 15.0 | (15.1) | 27,739 | 24.6 | (14.3) | 3,189 | 9.9 | (15.6) | *2.5* | 32.4 |  | 15.9 |  | 2.1 |  | 15.2 |  | 90.3 |  |
| 61 | Retinal disorders in diseases classified elsewhere | H36 | 19,279 | 4.2 | (4.3) | 6,889 | 6.1 | (5.4) | 954 | 3.0 | (2.9) | *2.1* | 5.6 |  | 6.6 |  | 2.3 |  | 8.0 |  | 7.7 |  |
| 62 | Glaucoma ^c^ | H40–H42 | 67,310 | 14.8 | (14.9) | 25,887 | 22.9 | (14.0) | 3,384 | 10.5 | (15.5) | *2.2* | 28.5 |  | 16.8 |  | 1.7 |  | 19.1 |  | 71.2 |  |
| 63 | Disorders of the vitreous body and globe | H43–H45 | 7,572 | 1.7 | (1.7) | 2,016 | 1.8 | (1.4) | 741 | 2.3 | (2.5) | *0.8* | 2.0 |  | 1.6 |  | 0.6 |  | 2.2 |  | 2.8 |  |
| 64 | Disorders of optic nerve and visual pathways | H46–H48 | 6,184 | 1.4 | (1.4) | 1,808 | 1.6 | (1.6) | 407 | 1.3 | (1.1) | *1.3* | 1.8 |  | 1.4 |  | 1.7 |  | 1.7 |  | 1.2 |  |
| 65 | Disorders of ocular muscles, binocular movement, accommodation and refraction | H49–H52 | 18,247 | 4.0 | (4.0) | 3,939 | 3.5 | (3.8) | 2,026 | 6.3 | (5.9) | *0.6* | 3.6 |  | 3.4 |  | 4.4 |  | 3.3 |  | 2.5 |  |
| 66 | Visual disturbances | H53 | 22,232 | 4.9 | (4.9) | 7,555 | 6.7 | (5.7) | 1,384 | 4.3 | (4.6) | *1.6* | 7.6 |  | 5.7 |  | 4.0 |  | 7.0 |  | 10.6 |  |
| 67 | Blindness and partial sight | H54 | 6,614 | 1.5 | (1.5) | 2,855 | 2.5 | (2.0) | 277 | 0.9 | (1.1) | *2.9* | 2.9 |  | 2.1 |  | 1.6 |  | 2.1 |  | 5.2 |  |
| 68 | Nystagmus and other irregular eye movements and other disorders of eye and adnexa | H55, H57 | 11,133 | 2.4 | (2.5) | 3,232 | 2.9 | (2.6) | 919 | 2.9 | (3.0) | *1.0* | 3.4 |  | 2.2 |  | 2.4 |  | 3.1 |  | 3.1 |  |
| 69 | Otosclerosis | H80 | 10,360 | 2.3 | (2.3) | 3,260 | 2.9 | (2.2) | 643 | 2.0 | (2.3) | *1.4* | 3.8 |  | 1.9 |  | 0.8 |  | 3.4 |  | 5.3 |  |
| 70 | Ménière’s disease ^c^ | H810 | 10,003 | 2.2 | (2.2) | 3,562 | 3.2 | (2.3) | 488 | 1.5 | (1.9) | *2.1* | 3.8 |  | 2.4 |  | 0.4 |  | 3.4 |  | 7.2 |  |
| 71 | Other diseases of the inner ear | H83 | 29,865 | 6.6 | (6.3) | 10,685 | 9.5 | (7.7) | 879 | 2.7 | (2.5) | *3.5* | 1.8 |  | 18.0 |  | 0.8 |  | 10.4 |  | 22.2 |  |
| 72 | Conductive and sensorineural hearing loss | H90 | 43,238 | 9.5 | (9.6) | 15,433 | 13.7 | (10.7) | 2,312 | 7.2 | (8.7) | *1.9* | 14.8 |  | 12.4 |  | 6.3 |  | 13.2 |  | 28.1 |  |
| 73 | Other hearing loss and other disorders of ear, not elsewhere classified | H910, H912, H913, H918, H930, H932, H933 | 8,306 | 1.8 | (1.8) | 2,992 | 2.7 | (2.2) | 405 | 1.3 | (1.4) | *2.1* | 2.7 |  | 2.6 |  | 1.3 |  | 2.6 |  | 5.2 |  |
| 74 | Presbycusis (age–related hearing loss) | H911 | 80,659 | 17.7 | (17.6) | 35,451 | 31.4 | (17.1) | 2,892 | 9.0 | (16.5) | *3.5* | 35.2 |  | 27.2 |  | 0.6 |  | 12.2 |  | 139.1 |  |
| 75 | Hearing loss, unspecified | H919 | 87,806 | 19.3 | (19.3) | 31,207 | 27.7 | (19.5) | 4,505 | 14.0 | (17.8) | *2.0* | 27.4 |  | 27.9 |  | 4.7 |  | 28.1 |  | 66.9 |  |
| 76 | Tinnitus | H931 | 40,124 | 8.8 | (8.7) | 12,187 | 10.8 | (8.9) | 2,657 | 8.3 | (8.6) | *1.3* | 9.7 |  | 12.0 |  | 2.5 |  | 13.8 |  | 17.0 |  |
| 77 | Other specified disorders of ear | H938 | 20,537 | 4.5 | (4.4) | 7,573 | 6.7 | (4.8) | 949 | 3.0 | (3.9) | *2.3* | 7.4 |  | 5.9 |  | 1.1 |  | 6.9 |  | 16.0 |  |
|  | **I – Diseases of the circulatory system** | **I05–I06; I10–28; I30–33; I36–141; I44–I52; I60–I88; I90–I94; I96–I99** | **1,254,427** | **275.4** | **(275.5)** | **444,581** | **393.9** | **(308.9)** | **60,629** | **188.7** | **(221.7)** | ***2.1*** | **453.8** |  | **327.4** |  | **87.3** |  | **453.2** |  | **768.1** |  |
| 78 | Aortic and mitral valve disease ^c^ | I05, I06, I34, I35 | 30,123 | 6.6 | (6.6) | 13,077 | 11.6 | (7.7) | 1,314 | 4.1 | (6.0) | *2.8* | 12.9 |  | 10.1 |  | 1.0 |  | 8.1 |  | 40.0 |  |
| 79 | Hypertensive diseases ^c^ | I10–I15 | 1,060,046 | 232.7 | (232.7) | 391,287 | 346.7 | (263.7) | 47,086 | 146.6 | (182.6) | *2.4* | 408.6 |  | 278.0 |  | 51.5 |  | 401.4 |  | 713.8 |  |
| 80 | Heart failure ^c^ | I11.0, I13.0, I13.2, I42.0, I42.6, I42.7, I42.9, I50.0, I50.1, I50.9 | 37,540 | 8.2 | (8.3) | 15,906 | 14.1 | (10.2) | 1,482 | 4.6 | (6.1) | *3.1* | 11.9 |  | 16.5 |  | 1.3 |  | 13.1 |  | 39.3 |  |
| 80A | Ischaemic heart diseases | I20–I25 | 139,173 | 30.6 | (30.7) | 56,244 | 49.8 | (37.7) | 5,320 | 16.6 | (20.9) | *3.0* | 45.5 |  | 54.7 |  | 4.7 |  | 54.8 |  | 115.3 |  |
| 81 | Angina pectoris | I20 | 78,476 | 17.2 | (17.3) | 30,898 | 27.4 | (21.4) | 3,136 | 9.8 | (11.8) | *2.8* | 25.6 |  | 29.4 |  | 3.0 |  | 33.4 |  | 53.6 |  |
| 82 | Acute myocardial infarction and subsequent myocardial infarction | I21–I22 | 36,654 | 8.0 | (8.1) | 14,841 | 13.2 | (10.2) | 1,310 | 4.1 | (4.9) | *3.2* | 10.5 |  | 16.1 |  | 1.4 |  | 14.5 |  | 30.2 |  |
| 83 | AMI complex/other | I23–I24 | 2,969 | 0.7 | (0.7) | 1,194 | 1.1 | (0.8) | 97 | 0.3 | (0.4) | *3.5* | 0.9 |  | 1.2 |  | 0.2 |  | 1.1 |  | 2.4 |  |
| 84 | Chronic ischaemic heart disease | I25 | 84,592 | 18.6 | (18.6) | 35,570 | 31.5 | (23.3) | 3,073 | 9.6 | (12.5) | *3.3* | 26.7 |  | 36.9 |  | 1.6 |  | 32.2 |  | 82.1 |  |
| 85 | Pulmonary heart disease and diseases of pulmonary circulation | I26–I28 | 15,352 | 3.4 | (3.4) | 6,122 | 5.4 | (4.1) | 641 | 2.0 | (2.6) | *2.7* | 6.4 |  | 4.4 |  | 1.5 |  | 5.0 |  | 13.6 |  |
| 86 | Acute pericarditis | I30 | 5,563 | 1.2 | (1.2) | 1,588 | 1.4 | (1.4) | 375 | 1.2 | (1.0) | *1.2* | 0.9 |  | 2.0 |  | 1.4 |  | 1.4 |  | 1.3 |  |
| 87 | Other forms of heart disease | I31–I43, except I34–I35 and I42 | 8,119 | 1.8 | (1.8) | 2,931 | 2.6 | (2.1) | 473 | 1.5 | (1.6) | *1.8* | 2.4 |  | 2.9 |  | 1.2 |  | 2.5 |  | 5.2 |  |
| 88 | Atrioventricular and left bundle branch block | I44 | 14,604 | 3.2 | (3.2) | 5,903 | 5.2 | (3.4) | 677 | 2.1 | (2.9) | *2.5* | 4.9 |  | 5.6 |  | 0.6 |  | 3.4 |  | 18.6 |  |
| 89 | Other conduction disorders | I45–46 | 11,823 | 2.6 | (2.6) | 4,182 | 3.7 | (2.9) | 662 | 2.1 | (2.3) | *1.8* | 3.3 |  | 4.2 |  | 1.4 |  | 3.3 |  | 9.0 |  |
| 90 | Paroxysmal tachycardia | I47 | 39,510 | 8.7 | (8.7) | 12,836 | 11.4 | (8.9) | 2,399 | 7.5 | (8.5) | *1.5* | 12.4 |  | 10.3 |  | 4.2 |  | 11.8 |  | 22.8 |  |
| 91 | Atrial fibrillation and flutter | I48 | 112,342 | 24.7 | (24.7) | 44,765 | 39.7 | (25.8) | 5,768 | 18.0 | (24.5) | *2.2* | 40.0 |  | 39.2 |  | 2.1 |  | 31.1 |  | 129.5 |  |
| 92 | Other cardiac arrhythmias | I49 | 34,418 | 7.6 | (7.6) | 11,871 | 10.5 | (7.6) | 2,086 | 6.5 | (7.7) | *1.6* | 11.6 |  | 9.3 |  | 2.4 |  | 9.4 |  | 28.0 |  |
| 93 | Complications and ill–defined descriptions of heart disease and other heart disorders in diseases classified elsewhere | I51–52 | 7,337 | 1.6 | (1.6) | 2,659 | 2.4 | (1.8) | 453 | 1.4 | (1.6) | *1.7* | 2.5 |  | 2.2 |  | 0.8 |  | 2.3 |  | 5.4 |  |
| 94 | Stroke | I60, I61, I63–I64, Z501 (rehabilitation) | 72,606 | 15.9 | (16.0) | 29,883 | 26.5 | (19.2) | 2,783 | 8.7 | (11.7) | *3.1* | 26.9 |  | 26.0 |  | 2.7 |  | 26.6 |  | 68.0 |  |
| 95 | Cerebrovascular diseases | I62, I65–I68 | 17,308 | 3.8 | (3.8) | 6,554 | 5.8 | (4.4) | 901 | 2.8 | (3.4) | *2.1* | 6.2 |  | 5.4 |  | 1.1 |  | 6.3 |  | 12.8 |  |
| 96 | Sequelae of cerebrovascular disease | I69 | 50,952 | 11.2 | (11.2) | 22,350 | 19.8 | (14.0) | 1,660 | 5.2 | (7.8) | *3.8* | 20.9 |  | 18.6 |  | 1.6 |  | 18.2 |  | 56.4 |  |
| 97 | Atherosclerosis | I70 | 32,064 | 7.0 | (7.0) | 15,289 | 13.5 | (9.9) | 705 | 2.2 | (3.8) | *6.2* | 14.2 |  | 12.8 |  | 0.7 |  | 13.2 |  | 37.0 |  |
| 98 | Aortic aneurysm and aortic dissection | I71 | 10,296 | 2.3 | (2.3) | 3,965 | 3.5 | (2.5) | 462 | 1.4 | (1.8) | *2.4* | 2.4 |  | 4.7 |  | 0.2 |  | 3.2 |  | 10.3 |  |
| 99 | Diseases of arteries, arterioles and capillaries | I72, I74, I77–I79 | 11,830 | 2.6 | (2.6) | 3,764 | 3.3 | (2.8) | 752 | 2.3 | (2.5) | *1.4* | 3.5 |  | 3.2 |  | 1.1 |  | 3.8 |  | 5.8 |  |
| 100 | Other peripheral vascular diseases | I73 | 28,508 | 6.3 | (6.3) | 12,644 | 11.2 | (8.5) | 746 | 2.3 | (3.4) | *4.8* | 11.5 |  | 10.9 |  | 0.9 |  | 12.5 |  | 25.8 |  |
| 101 | Phlebitis, thrombosis of the portal vein and others | I80–I82 | 37,388 | 8.2 | (8.3) | 13,747 | 12.2 | (10.1) | 1,709 | 5.3 | (5.8) | *2.3* | 13.9 |  | 10.2 |  | 5.3 |  | 12.8 |  | 22.6 |  |
| 102 | Varicose veins of lower extremities | I83 | 23,530 | 5.2 | (5.2) | 6,589 | 5.8 | (5.4) | 1,169 | 3.6 | (3.4) | *1.6* | 7.8 |  | 3.6 |  | 2.6 |  | 7.5 |  | 7.0 |  |
| 103 | Haemorrhoids ^c^ | I84 | 74,285 | 16.3 | (16.3) | 18,741 | 16.6 | (16.5) | 5,238 | 16.3 | (14.5) | *1.0* | 19.2 |  | 13.7 |  | 14.3 |  | 17.0 |  | 19.5 |  |
| 104 | Oesophageal varices (chronic), varicose veins of other sites, other disorders of veins, non–specific lymphadenitis, other non–infective disorders of lymphatic vessels and lymph nodes and other and unspecified disorders of the circulatory system | I85–I99, except I89 and I95 | 15,194 | 3.3 | (3.3) | 4,972 | 4.4 | (3.9) | 840 | 2.6 | (2.8) | *1.7* | 4.4 |  | 4.5 |  | 2.8 |  | 4.7 |  | 6.4 |  |
|  | **J – Diseases of the respiratory system** | **J30.1; J40–J47; J60–J84; J95, J97–J99** | **1,210,598** | **265.7** | **(266.3)** | **336,462** | **298.1** | **(278.0)** | **88,121** | **274.3** | **(275.5)** | ***1.1*** | **348.9** |  | **241.8** |  | **218.5** |  | **318.2** |  | **382.2** |  |
| 105 | Respiratory allergy ^c^ | J30, except J30.0 | 841,685 | 184.8 | (185.2) | 207,831 | 184.2 | (172.6) | 70,172 | 218.4 | (218.2) | *0.8* | 221.9 |  | 142.2 |  | 142.2 |  | 195.3 |  | 226.9 |  |
| 105A | Chronic lower respiratory diseases ^c^ | J40–J43, J47 | 418,120 | 91.8 | (92.0) | 136,583 | 121.0 | (110.3) | 27,404 | 85.3 | (83.4) | *1.4* | 147.2 |  | 92.0 |  | 68.5 |  | 137.0 |  | 169.0 |  |
| 106 | Bronchitis, not specified as acute or chronic, simple and mucopurulent chronic bronchitis  and unspecified chronic bronchitis | J40–J42 | 12,790 | 2.8 | (2.8) | 6,448 | 5.7 | (4.5) | 277 | 0.9 | (1.4) | *6.6* | 6.7 |  | 4.6 |  | 0.9 |  | 6.1 |  | 13.1 |  |
| 107 | Emphysema | J43 | 5,557 | 1.2 | (1.2) | 2,489 | 2.2 | (1.8) | 153 | 0.5 | (0.6) | *4.6* | 2.3 |  | 2.1 |  | 0.4 |  | 2.7 |  | 3.9 |  |
| 108 | Chronic obstructive lung disease (COPD) ^c^ | J44, J96, J13–J18 | 216,184 | 47.5 | (47.6) | 88,085 | 78.0 | (64.7) | 7,600 | 23.7 | (30.1) | *3.3* | 89.3 |  | 65.5 |  | 23.9 |  | 88.6 |  | 143.9 |  |
| 109 | Asthma, status asthmaticus ^c^ | J45–J46 | 361,129 | 79.3 | (79.4) | 115,623 | 102.4 | (96.5) | 21,606 | 67.3 | (69.4) | *1.5* | 120.8 |  | 82.1 |  | 77.6 |  | 107.5 |  | 132.2 |  |
| 110 | Bronchiectasis | J47 | 4,362 | 1.0 | (1.0) | 1,355 | 1.2 | (0.9) | 273 | 0.8 | (1.0) | *1.4* | 1.5 |  | 0.8 |  | 0.3 |  | 1.4 |  | 2.2 |  |
| 111 | Other diseases of the respiratory system | J60–J84; J95, J97–J99 | 21,993 | 4.8 | (4.9) | 8,626 | 7.6 | (6.2) | 815 | 2.5 | (3.0) | *3.0* | 7.7 |  | 7.5 |  | 2.8 |  | 8.3 |  | 14.4 |  |
|  | **K – Diseases of the digestive system** | **K25–K27; K40, K43, K50–52; K58–K59; K71–K77; K86–K87** | **329,337** | **72.3** | **(72.6)** | **113,926** | **100.9** | **(88.5)** | **15,438** | **48.1** | **(50.7)** | ***2.1*** | **114.1** |  | **86.3** |  | **57.8** |  | **107.2** |  | **159.4** |  |
| 112 | Ulcers ^c^ | K25–K27 | 157,379 | 34.5 | (34.8) | 61,390 | 54.4 | (45.9) | 5,758 | 17.9 | (20.8) | *3.0* | 63.2 |  | 44.6 |  | 26.4 |  | 57.4 |  | 95.3 |  |
| 113 | Inguinal hernia | K40 | 25,032 | 5.5 | (5.5) | 6,910 | 6.1 | (5.4) | 1,878 | 5.8 | (5.4) | *1.0* | 1.6 |  | 11.1 |  | 2.5 |  | 7.2 |  | 9.3 |  |
| 114 | Ventral hernia | K43 | 7,941 | 1.7 | (1.7) | 2,962 | 2.6 | (2.3) | 312 | 1.0 | (1.0) | *2.7* | 3.0 |  | 2.2 |  | 1.2 |  | 3.3 |  | 3.3 |  |
| 115 | Crohn’s disease | K50 | 18,913 | 4.2 | (4.2) | 5,052 | 4.5 | (4.7) | 1,205 | 3.8 | (3.3) | *1.2* | 5.2 |  | 3.6 |  | 5.0 |  | 4.6 |  | 3.4 |  |
| 116 | Ulcerative colitis | K51 | 29,538 | 6.5 | (6.5) | 7,511 | 6.7 | (6.4) | 2,135 | 6.6 | (6.2) | *1.0* | 7.3 |  | 6.0 |  | 5.0 |  | 7.2 |  | 8.2 |  |
| 117 | Other non–infective gastroenteritis and colitis | K52 | 20,844 | 4.6 | (4.6) | 7,733 | 6.9 | (5.6) | 810 | 2.5 | (3.2) | *2.7* | 9.1 |  | 4.3 |  | 3.6 |  | 6.5 |  | 13.5 |  |
| 118 | Irritable bowel syndrome (IBS) | K58 | 37,593 | 8.3 | (8.3) | 10,531 | 9.3 | (9.3) | 2,040 | 6.4 | (6.0) | *1.5* | 12.8 |  | 5.5 |  | 8.4 |  | 10.1 |  | 8.7 |  |
| 119 | Other functional intestinal disorders | K59 | 51,933 | 11.4 | (11.5) | 19,528 | 17.3 | (14.5) | 2,129 | 6.6 | (8.0) | *2.6* | 21.7 |  | 12.4 |  | 10.0 |  | 15.8 |  | 34.3 |  |
| 120 | Diseases of liver, biliary tract and pancreas | K71–K77; K86–K87 | 26,956 | 5.9 | (6.0) | 9,766 | 8.7 | (7.8) | 1,097 | 3.4 | (3.4) | *2.5* | 9.0 |  | 8.3 |  | 4.3 |  | 11.5 |  | 8.4 |  |
|  | **L – Diseases of the skin and subcutaneous tissue** | **L40** | 65,469 | 14.4 | (14.5) | 18,801 | 16.7 | (14.7) | 4,593 | 14.3 | (14.4) | *1.2* | 18.4 |  | 14.7 |  | 8.8 |  | 20.4 |  | 20.1 |  |
| 121 | Psoriasis ^c^ | L40 | 65,469 | 14.4 | (14.5) | 18,801 | 16.7 | (14.7) | 4,593 | 14.3 | (14.4) | *1.2* | 18.4 |  | 14.7 |  | 8.8 |  | 20.4 |  | 20.1 |  |
|  | **M – Diseases of the musculoskeletal system and connective tissue** | **M01–M25; M30–M36; M40–M54; M60.1–M99** | **1,032,808** | **226.7** | **(227.1)** | **332,557** | **294.7** | **(246.7)** | **51,276** | **159.6** | **(179.6)** | ***1.8*** | **353.4** |  | **229.4** |  | **137.4** |  | **328.9** |  | **476.0** |  |
| 122 | Infectious arthropathies | M01–M03 | 9,402 | 2.1 | (2.1) | 2,317 | 2.1 | (2.1) | 720 | 2.2 | (2.0) | *0.9* | 2.0 |  | 2.2 |  | 1.9 |  | 2.2 |  | 2.1 |  |
| 122A | Inflammatory polyarthropathies and ankylosing spondylitis ^c^ | M05–M14, M45 | 165,944 | 36.4 | (36.5) | 55,657 | 49.3 | (40.8) | 9,676 | 30.1 | (31.9) | *1.6* | 50.8 |  | 47.6 |  | 13.9 |  | 59.2 |  | 84.2 |  |
| 123 | Rheumatoid arthritis ^c^ | M05, M06, M07.1, M07.2, M07.3, M08, M09 | 77,345 | 17.0 | (17.0) | 24,192 | 21.4 | (18.0) | 5,714 | 17.8 | (19.1) | *1.2* | 29.2 |  | 12.8 |  | 7.6 |  | 26.5 |  | 31.8 |  |
| 124 | Inflammatory polyarthropathies – except rheumatoid arthritis ^c^ | M074–M079, M10–M14, M45 | 115,945 | 25.5 | (25.5) | 39,923 | 35.4 | (29.4) | 5,659 | 17.6 | (18.4) | *2.0* | 32.3 |  | 38.7 |  | 9.3 |  | 42.1 |  | 62.4 |  |
| 125 | Polyarthrosis [arthrosis] | M15 | 16,935 | 3.7 | (3.7) | 6,680 | 5.9 | (3.9) | 545 | 1.7 | (3.1) | *3.5* | 9.5 |  | 2.0 |  | 0.3 |  | 7.2 |  | 12.3 |  |
| 126 | Coxarthrosis [arthrosis of hip] | M16 | 104,115 | 22.9 | (22.7) | 44,227 | 39.2 | (26.4) | 3,824 | 11.9 | (18.8) | *3.3* | 46.2 |  | 31.4 |  | 2.8 |  | 34.9 |  | 115.2 |  |
| 127 | Gonarthrosis [arthrosis of knee] | M17 | 178,811 | 39.3 | (39.4) | 64,860 | 57.5 | (42.4) | 6,420 | 20.0 | (27.3) | *2.9* | 71.1 |  | 42.3 |  | 6.4 |  | 69.2 |  | 114.5 |  |
| 128 | Arthrosis of first carpometacarpal joint and other arthrosis | M18–M19 | 91,101 | 20.0 | (20.1) | 32,138 | 28.5 | (22.3) | 3,149 | 9.8 | (12.6) | *2.9* | 35.1 |  | 21.1 |  | 5.7 |  | 37.9 |  | 42.2 |  |
| 129 | Acquired deformities of fingers and toes | M20 | 55,730 | 12.2 | (12.3) | 16,302 | 14.4 | (11.8) | 2,630 | 8.2 | (10.0) | *1.8* | 22.4 |  | 5.7 |  | 6.0 |  | 18.9 |  | 16.8 |  |
| 130 | Other acquired deformities of limbs | M21 | 20,584 | 4.5 | (4.5) | 6,564 | 5.8 | (5.1) | 857 | 2.7 | (3.1) | *2.2* | 7.7 |  | 3.8 |  | 3.6 |  | 6.7 |  | 7.3 |  |
| 131 | Disorders of patella (knee cap) | M22 | 38,999 | 8.6 | (8.6) | 8,826 | 7.8 | (10.0) | 2,245 | 7.0 | (6.3) | *1.1* | 9.2 |  | 6.3 |  | 17.1 |  | 4.6 |  | 0.6 |  |
| 132 | Internal derangement of knee | M230, M231, M233, M235, M236, M238 | 9,192 | 2.0 | (2.0) | 1,772 | 1.6 | (1.9) | 680 | 2.1 | (2.0) | *0.7* | 1.3 |  | 1.9 |  | 2.8 |  | 1.2 |  | 0.4 |  |
| 133 | Derangement of meniscus due to old tear or injury | M232 | 36,374 | 8.0 | (8.0) | 8,064 | 7.1 | (7.6) | 2,467 | 7.7 | (7.1) | *0.9* | 6.9 |  | 7.4 |  | 7.3 |  | 8.8 |  | 2.3 |  |
| 134 | Internal derangement of knee, unspecified | M239 | 28,206 | 6.2 | (6.2) | 6,488 | 5.7 | (6.4) | 1,551 | 4.8 | (4.4) | *1.2* | 5.7 |  | 5.8 |  | 7.8 |  | 5.7 |  | 2.1 |  |
| 135 | Other specific joint derangements | M24, except M240–M241 | 5,923 | 1.3 | (1.3) | 1,325 | 1.2 | (1.3) | 401 | 1.2 | (1.3) | *0.9* | 1.0 |  | 1.4 |  | 2.1 |  | 0.8 |  | 0.5 |  |
| 136 | Other joint disorders, not elsewhere classified | M25 | 12,043 | 2.6 | (2.7) | 3,345 | 3.0 | (3.1) | 591 | 1.8 | (1.7) | *1.6* | 3.8 |  | 2.1 |  | 3.1 |  | 3.3 |  | 1.8 |  |
| 137 | Systemic connective tissue disorders | M30–M36, except M32, M34 | 42,631 | 9.4 | (9.4) | 15,606 | 13.8 | (10.5) | 1,965 | 6.1 | (7.8) | *2.3* | 20.4 |  | 6.5 |  | 5.3 |  | 12.4 |  | 32.8 |  |
| 138 | Systemic lupus erythematosus | M32 | 3,376 | 0.7 | (0.7) | 1,040 | 0.9 | (0.9) | 181 | 0.6 | (0.6) | *1.6* | 1.5 |  | 0.3 |  | 0.6 |  | 1.2 |  | 0.7 |  |
| 139 | Dermatopolymyositis | M33 | 1,137 | 0.2 | (0.2) | 381 | 0.3 | (0.3) | 72 | 0.2 | (0.2) | *1.5* | 0.4 |  | 0.2 |  | 0.1 |  | 0.4 |  | 0.4 |  |
| 140 | Systemic sclerosis | M34 | 1,675 | 0.4 | (0.4) | 611 | 0.5 | (0.5) | 65 | 0.2 | (0.3) | *2.7* | 0.8 |  | 0.2 |  | 0.2 |  | 0.8 |  | 0.6 |  |
| 141 | Kyphosis, lordosis | M40 | 4,160 | 0.9 | (0.9) | 1,327 | 1.2 | (1.2) | 291 | 0.9 | (0.8) | *1.3* | 1.2 |  | 1.1 |  | 1.1 |  | 1.4 |  | 0.7 |  |
| 142 | Scoliosis | M41 | 17,686 | 3.9 | (3.9) | 5,287 | 4.7 | (4.8) | 1,059 | 3.3 | (3.8) | *1.4* | 5.9 |  | 3.4 |  | 6.9 |  | 3.4 |  | 4.4 |  |
| 143 | Spinal osteochondrosis | M42 | 8,034 | 1.8 | (1.8) | 2,616 | 2.3 | (2.4) | 423 | 1.3 | (1.0) | *1.8* | 1.6 |  | 3.1 |  | 2.3 |  | 2.8 |  | 1.0 |  |
| 144 | Other deforming dorsopathies | M43 | 23,756 | 5.2 | (5.3) | 8,159 | 7.2 | (6.1) | 1,031 | 3.2 | (3.5) | *2.3* | 8.6 |  | 5.7 |  | 3.0 |  | 9.0 |  | 9.6 |  |
| 145 | Other inflammatory spondylopathies | M46 | 7,086 | 1.6 | (1.6) | 2,144 | 1.9 | (1.8) | 409 | 1.3 | (1.1) | *1.5* | 2.0 |  | 1.8 |  | 1.3 |  | 2.3 |  | 1.9 |  |
| 146 | Spondylosis | M47 | 61,999 | 13.6 | (13.6) | 23,358 | 20.7 | (16.9) | 2,108 | 6.6 | (7.9) | *3.2* | 23.6 |  | 17.5 |  | 3.8 |  | 27.5 |  | 31.4 |  |
| 147 | Other spondylopathies and spondylopathies in diseases classified elsewhere | M48, M49 | 50,805 | 11.2 | (11.2) | 19,916 | 17.6 | (12.3) | 1,990 | 6.2 | (8.9) | *2.8* | 21.4 |  | 13.5 |  | 1.9 |  | 19.1 |  | 41.2 |  |
| 148 | Cervical disc disorders | M50 | 11,476 | 2.5 | (2.5) | 3,049 | 2.7 | (2.8) | 607 | 1.9 | (1.4) | *1.4* | 2.9 |  | 2.5 |  | 1.9 |  | 3.8 |  | 1.0 |  |
| 149 | Other intervertebral disc disorders | M51 | 40,161 | 8.8 | (8.9) | 11,706 | 10.4 | (10.4) | 1,921 | 6.0 | (4.6) | *1.7* | 10.7 |  | 10.0 |  | 8.7 |  | 12.5 |  | 7.2 |  |
| 150 | Other dorsopathies, not elsewhere classified | M53 | 7,246 | 1.6 | (1.6) | 2,057 | 1.8 | (1.9) | 334 | 1.0 | (0.8) | *1.8* | 2.1 |  | 1.5 |  | 1.8 |  | 2.1 |  | 1.3 |  |
| 151 | Dorsalgia | M54 | 40,780 | 9.0 | (9.0) | 12,850 | 11.4 | (11.2) | 1,853 | 5.8 | (4.9) | *2.0* | 12.9 |  | 9.7 |  | 10.9 |  | 11.5 |  | 11.9 |  |
| 152 | Soft tissue disorders | M60–M63, except M60.0 | 13,422 | 2.9 | (3.0) | 4,102 | 3.6 | (3.9) | 602 | 1.9 | (1.6) | *1.9* | 4.9 |  | 2.2 |  | 4.9 |  | 3.3 |  | 2.5 |  |
| 153 | Synovitis and tenosynovitis | M65 | 19,104 | 4.2 | (4.2) | 5,362 | 4.8 | (4.6) | 969 | 3.0 | (3.1) | *1.6* | 6.2 |  | 3.1 |  | 3.5 |  | 5.9 |  | 3.8 |  |
| 154 | Disorders of synovium and tendon | M66–68 | 19,669 | 4.3 | (4.3) | 4,662 | 4.1 | (4.5) | 1,059 | 3.3 | (3.1) | *1.3* | 4.8 |  | 3.4 |  | 5.4 |  | 4.1 |  | 1.9 |  |
| 155 | Soft tissue disorders related to use, overuse and pressure | M70 | 11,090 | 2.4 | (2.4) | 3,275 | 2.9 | (2.7) | 511 | 1.6 | (1.6) | *1.8* | 3.5 |  | 2.3 |  | 1.6 |  | 3.4 |  | 3.7 |  |
| 156 | Fibroblastic disorders | M72 | 43,600 | 9.6 | (9.6) | 13,106 | 11.6 | (9.4) | 2,501 | 7.8 | (8.1) | *1.5* | 8.9 |  | 14.6 |  | 2.3 |  | 14.7 |  | 19.4 |  |
| 157 | Shoulder lesions | M75 | 58,112 | 12.8 | (12.7) | 16,538 | 14.7 | (14.6) | 2,428 | 7.6 | (6.3) | *1.9* | 14.8 |  | 14.5 |  | 9.2 |  | 20.3 |  | 8.6 |  |
| 158 | Enthesopathies of lower limb, excluding foot | M76 | 11,223 | 2.5 | (2.5) | 2,003 | 1.8 | (2.0) | 1,063 | 3.3 | (3.2) | *0.5* | 1.9 |  | 1.6 |  | 1.9 |  | 2.0 |  | 0.8 |  |
| 159 | Other enthesopathies | M77 | 10,500 | 2.3 | (2.3) | 2,600 | 2.3 | (2.5) | 644 | 2.0 | (1.7) | *1.1* | 2.8 |  | 1.7 |  | 2.1 |  | 2.9 |  | 0.9 |  |
| 160 | Rheumatism, unspecified | M790 | 6,852 | 1.5 | (1.5) | 2,786 | 2.5 | (2.4) | 188 | 0.6 | (0.6) | *4.2* | 4.2 |  | 0.6 |  | 1.4 |  | 3.6 |  | 1.3 |  |
| 161 | Myalgia | M791 | 10,168 | 2.2 | (2.2) | 3,661 | 3.2 | (3.1) | 352 | 1.1 | (0.9) | *3.0* | 4.1 |  | 2.3 |  | 2.3 |  | 3.9 |  | 3.1 |  |
| 162 | Other soft tissue disorders, not elsewhere classified | M792– M794; M798–M799 | 7,939 | 1.7 | (1.7) | 2,299 | 2.0 | (2.1) | 514 | 1.6 | (1.4) | *1.3* | 2.6 |  | 1.5 |  | 1.6 |  | 2.4 |  | 1.7 |  |
| 163 | Other soft tissue disorders, not elsewhere classified: pain in limb | M796 | 22,201 | 4.9 | (4.9) | 6,978 | 6.2 | (5.9) | 904 | 2.8 | (2.8) | *2.2* | 7.4 |  | 4.8 |  | 5.2 |  | 6.5 |  | 7.0 |  |
| 164 | Fibromyalgia | M797 | 3,399 | 0.7 | (0.7) | 1,271 | 1.1 | (1.2) | 65 | 0.2 | (0.1) | *5.6* | 2.1 |  | 0.1 |  | 1.0 |  | 1.5 |  | 0.3 |  |
| 165 | Osteoporosis ^c^ | M80–M81 | 158,813 | 34.9 | (34.8) | 68,692 | 60.9 | (36.0) | 5,055 | 15.7 | (33.2) | *3.9* | 101.6 |  | 15.6 |  | 1.1 |  | 56.9 |  | 177.0 |  |
| 166 | Osteoporosis in diseases classified elsewhere | M82 | 1,007 | 0.2 | (0.2) | 401 | 0.4 | (0.3) | 42 | 0.1 | (0.2) | *2.7* | 0.5 |  | 0.2 |  | 0.1 |  | 0.4 |  | 0.7 |  |
| 167 | Adult osteomalacia and other disorders of bone density and structure | M83, M85, except M833 | 43,271 | 9.5 | (9.5) | 14,803 | 13.1 | (9.2) | 1,932 | 6.0 | (8.8) | *2.2* | 20.9 |  | 4.5 |  | 2.4 |  | 16.2 |  | 23.4 |  |
| 168 | Disorders of continuity of bone | M84 | 1,865 | 0.4 | (0.4) | 648 | 0.6 | (0.5) | 70 | 0.2 | (0.3) | *2.6* | 0.6 |  | 0.6 |  | 0.6 |  | 0.6 |  | 0.5 |  |
| 169 | Other osteopathies | M86–M90 | 24,251 | 5.3 | (5.3) | 9,059 | 8.0 | (6.6) | 894 | 2.8 | (3.4) | *2.9* | 10.1 |  | 5.7 |  | 3.2 |  | 9.1 |  | 13.4 |  |
| 170 | Other disorders of the musculoskeletal system and connective tissue | M95–M99 | 30,038 | 6.6 | (6.6) | 9,686 | 8.6 | (8.4) | 1,401 | 4.4 | (3.9) | *2.0* | 8.5 |  | 8.7 |  | 7.8 |  | 9.1 |  | 8.3 |  |
|  | **N – Diseases of the genitourinary system** | **N18** | **20,162** | **4.4** | **(4.5)** | **8,621** | **7.6** | **(5.9)** | **713** | **2.2** | **(2.8)** | ***3.4*** | **7.0** |  | **8.4** |  | **1.7** |  | **6.8** |  | **20.5** |  |
| 171 | Chronic renal failure (CRF) ^c^ | N18 | 20,162 | 4.4 | (4.5) | 8,621 | 7.6 | (5.9) | 713 | 2.2 | (2.8) | *3.4* | 7.0 |  | 8.4 |  | 1.7 |  | 6.8 |  | 20.5 |  |
|  | **Q – Congenital malformations, deformations, and chromosomal abnormalities** | **Q00–Q56; Q60–Q99** | **124,898** | **27.4** | **(27.5)** | **33,012** | **29.3** | **(32.6)** | **9,581** | **29.8** | **(26.4)** | ***1.0*** | **31.4** |  | **26.8** |  | **43.7** |  | **24.9** |  | **16.0** |  |
| 172 | Congenital malformations: of the nervous, circulatory and respiratory systems, cleft palate and cleft lip, urinary tract, bones and muscles, other and chromosomal abnormalities not elsewhere classified | Q00–Q07; Q20–Q37; Q60–Q99 | 85,534 | 18.8 | (18.9) | 23,361 | 20.7 | (23.3) | 6,333 | 19.7 | (17.6) | *1.1* | 23.6 |  | 17.5 |  | 32.5 |  | 17.2 |  | 9.7 |  |
| 173 | Congenital malformations of eye, ear, face and neck | Q10–Q18 | 19,689 | 4.3 | (4.3) | 4,569 | 4.0 | (4.8) | 1,621 | 5.0 | (4.4) | *0.8* | 4.4 |  | 3.7 |  | 6.9 |  | 3.0 |  | 1.9 |  |
| 174 | Other congenital malformations of the digestive system | Q38–Q45 | 6,481 | 1.4 | (1.4) | 2,257 | 2.0 | (1.8) | 367 | 1.1 | (1.2) | *1.8* | 2.4 |  | 1.6 |  | 1.4 |  | 2.0 |  | 3.0 |  |
| 175 | Congenital malformations of the sexual organs | Q50–Q56 | 16,192 | 3.6 | (3.6) | 3,704 | 3.3 | (3.7) | 1,509 | 4.7 | (3.9) | *0.7* | 1.9 |  | 4.8 |  | 4.6 |  | 3.1 |  | 1.6 |  |
|  | **F – Mental and behavioural disorders** | **F00–99** | **683,194** | **150.0** | **(150.7)** | **254,454** | **225.5** | **(222.3)** | **28,936** | **90.1** | **(85.4)** | ***2.5*** | **251.9** |  | **196.1** |  | **273.4** |  | **191.2** |  | **236.7** |  |
| 176 | Dementia ^c^ | F00, G30, F01, F02.0, F03.9, G31.8B, G31.8E, G31.9, G31.0B | 36,803 | 8.1 | (8.1) | 18,319 | 16.2 | (8.8) | 1,066 | 3.3 | (7.5) | *4.9* | 22.0 |  | 9.8 |  | 0.1 |  | 5.5 |  | 74.5 |  |
| 177 | Organic, including symptomatic, mental disorders | F04–F09 | 26,430 | 5.8 | (5.9) | 12,791 | 11.3 | (9.4) | 890 | 2.8 | (3.3) | *4.1* | 11.7 |  | 10.9 |  | 8.2 |  | 9.2 |  | 22.8 |  |
| 178 | Mental and behavioural disorders due to use of alcohol | F10 | 59,143 | 13.0 | (13.2) | 24,641 | 21.8 | (22.5) | 1,701 | 5.3 | (3.9) | *4.1* | 13.8 |  | 30.7 |  | 25.3 |  | 25.2 |  | 6.2 |  |
| 179 | Mental and behavioural disorders due to psychoactive substance use | F11–F19 | 53,669 | 11.8 | (11.9) | 26,234 | 23.2 | (25.0) | 956 | 3.0 | (1.6) | *7.8* | 19.7 |  | 27.2 |  | 41.6 |  | 17.4 |  | 7.4 |  |
| 180 | Schizophrenia ^c^ | F20 | 29,422 | 6.5 | (6.5) | 15,920 | 14.1 | (15.3) | 776 | 2.4 | (0.8) | *5.8* | 11.1 |  | 17.5 |  | 24.2 |  | 11.8 |  | 2.8 |  |
| 181 | Schizotypal and delusional disorders | F21–F29 | 39,694 | 8.7 | (8.8) | 18,976 | 16.8 | (17.8) | 1,513 | 4.7 | (3.2) | *3.6* | 15.7 |  | 18.0 |  | 27.4 |  | 13.6 |  | 7.1 |  |
| 182 | Bipolar affective disorder ^c^ | F30–F31 | 22,669 | 5.0 | (5.0) | 8,109 | 7.2 | (7.0) | 1,326 | 4.1 | (3.9) | *1.7* | 8.6 |  | 5.6 |  | 7.8 |  | 7.3 |  | 5.8 |  |
| 183 | Depression ^c^ | F32, F33, F34.1, F06.32 | 454,933 | 99.9 | (100.2) | 161,524 | 143.1 | (136.8) | 19,825 | 61.7 | (59.3) | *2.3* | 180.4 |  | 101.7 |  | 142.5 |  | 130.6 |  | 179.1 |  |
| 184 | Mood (affective) disorders | F340, F348–F349, F38–F39 | 6,887 | 1.5 | (1.5) | 2,790 | 2.5 | (2.5) | 271 | 0.8 | (0.7) | *2.9* | 3.1 |  | 1.8 |  | 3.2 |  | 2.2 |  | 2.0 |  |
| 185 | Phobic anxiety disorders | F40 | 14,324 | 3.1 | (3.2) | 5,804 | 5.1 | (6.1) | 630 | 2.0 | (1.1) | *2.6* | 6.2 |  | 4.0 |  | 11.8 |  | 2.7 |  | 0.2 |  |
| 186 | Other anxiety disorders | F41 | 38,079 | 8.4 | (8.4) | 14,581 | 12.9 | (14.2) | 1,686 | 5.2 | (3.8) | *2.5* | 16.2 |  | 9.3 |  | 22.0 |  | 9.8 |  | 5.8 |  |
| 187 | Obsessive compulsive disorder (OCD) ^c^ | F42 | 10,062 | 2.2 | (2.2) | 3,294 | 2.9 | (3.5) | 578 | 1.8 | (1.6) | *1.6* | 3.4 |  | 2.4 |  | 7.2 |  | 1.1 |  | 0.4 |  |
| 188 | Post–traumatic stress disorder | F431 | 16,055 | 3.5 | (3.6) | 5,724 | 5.1 | (5.9) | 592 | 1.8 | (0.7) | *2.8* | 5.1 |  | 5.1 |  | 9.5 |  | 4.0 |  | 0.3 |  |
| 189 | Reactions to severe stress and adjustment disorders | F432–F439 | 61,701 | 13.5 | (13.7) | 23,214 | 20.6 | (23.5) | 2,086 | 6.5 | (5.2) | *3.2* | 22.7 |  | 18.2 |  | 43.0 |  | 12.2 |  | 4.4 |  |
| 190 | Dissociative (conversion) disorders, somatoform disorders and other neurotic disorders | F44, F45, F48 | 21,420 | 4.7 | (4.7) | 7,912 | 7.0 | (7.5) | 978 | 3.0 | (2.2) | *2.3* | 9.2 |  | 4.6 |  | 9.2 |  | 7.0 |  | 3.2 |  |
| 191 | Eating disorders | F50 | 7,751 | 1.7 | (1.7) | 2,039 | 1.8 | (2.5) | 339 | 1.1 | (1.3) | *1.7* | 3.2 |  | 0.3 |  | 5.2 |  | 0.3 |  | 0.1 |  |
| 192 | Behavioural syndromes associated with physiological disturbances and physical factors | F51–F59 | 6,163 | 1.4 | (1.3) | 1,354 | 1.2 | (1.5) | 660 | 2.1 | (1.7) | *0.6* | 1.1 |  | 1.3 |  | 2.3 |  | 0.8 |  | 0.3 |  |
| 193 | Emotionally unstable personality disorder | F603 | 21,848 | 4.8 | (4.9) | 10,738 | 9.5 | (11.3) | 587 | 1.8 | (0.3) | *5.2* | 13.9 |  | 4.6 |  | 22.9 |  | 4.4 |  | 0.2 |  |
| 194 | Specific personality disorders | F602, F604–F609 | 50,415 | 11.1 | (11.2) | 21,873 | 19.4 | (22.0) | 1,876 | 5.8 | (2.6) | *3.3* | 21.2 |  | 17.3 |  | 37.8 |  | 13.6 |  | 3.0 |  |
| 195 | Disorders of adult personality and behaviour | F61–F69 | 17,533 | 3.8 | (3.9) | 7,571 | 6.7 | (7.7) | 665 | 2.1 | (0.8) | *3.2* | 6.9 |  | 6.5 |  | 13.3 |  | 4.7 |  | 0.8 |  |
| 196 | Mental retardation | F70–F79 | 13,822 | 3.0 | (3.1) | 11,095 | 9.8 | (10.6) | 39 | 0.1 | N/A | *81.0* | 8.8 |  | 10.9 |  | 18.0 |  | 7.5 |  | 2.2 |  |
| 197 | Disorders of psychological development | F80–F89 | 9,911 | 2.2 | (2.2) | 5,937 | 5.3 | (5.9) | 119 | 0.4 | (0.7) | *14.2* | 3.4 |  | 7.3 |  | 14.0 |  | 1.5 |  | 0.4 |  |
| 198 | Hyperkinetic disorders (ADHD) ^c^ | F90 | 42,908 | 9.4 | (9.5) | 19,548 | 17.3 | (19.7) | 1,048 | 3.3 | (3.6) | *5.3* | 12.0 |  | 23.3 |  | 47.7 |  | 4.1 |  | 0.9 |  |
| 199 | Behavioural and emotional disorders with onset usually occurring in childhood and adolescence | F91–F99 | 39,602 | 8.7 | (8.8) | 17,990 | 15.9 | (17.6) | 1,295 | 4.0 | (3.1) | *4.0* | 15.1 |  | 16.9 |  | 32.9 |  | 9.6 |  | 3.9 |  |
|  |  |  |  |  |  |  |  |  |  |  |  |  |  |  |  |  |  |  |  |  |  |  |
|  | **Having one or more chronic conditions** |  | **2,989,441** | **656.2** | **(657.2)** | **867,345** | **768.5** | **(713.2)** | **193,164** | **601.3** | **(604.2)** | ***1.3*** | **833.8** |  | **696.1** |  | **582.0** |  | **819.5** |  | **954.6** |  |
|  |  |  |  |  |  |  |  |  |  |  |  |  |  |  |  |  |  |  |  |  |  |  |
|  | **Total population** |  | **4,555,439** | **1000** | **N/A** | **1,128,588** | **1,000** | **(1000)** | **321,254** | **1,000** | **(1000)** | ***1.0*** | **1,000** |  | **1,000** |  | **1,000** |  | **1,000** |  | **1,000** |  |
|  |  |  |  |  |  |  |  |  |  |  |  |  |  |  |  |  |  |  |  |  |  |  |
|  | Depression medicine ^c^ ** | ATC: N06A. | 529,918 | 116.3 | (116.7) | 191,590 | 70.9 | (160.4) | 22,768 | 169.8 | (70.0) | 2.4 | 212.1 |  | 122.8 |  | 161.0 |  | 158.4 |  | 216.9 |  |
|  | Antipsychotic medicine ^c^ ** | ATC: N05A. | 138,625 | 30.4 | (30.6) | 64,514 | 13.2 | (56.7) | 4,225 | 57.2 | (11.8) | 4.3 | 59.3 |  | 54.8 |  | 74.6 |  | 48.9 |  | 49.6 |  |
|  | Indication prescribed anxiety medicine ^c^ ** | All prescrib. w.indication codes 163 (for anxiety) or 371 (for anxiety, addictive) | 102,568 | 22.5 | (22.6) | 37,917 | 13.7 | (33.3) | 4,400 | 33.6 | (12.7) | 2.5 | 42.5 |  | 23.7 |  | 39.5 |  | 30.5 |  | 31.8 |  |
|  | Heart failure medication ^c^ ** | ATC: C01AA05, C03, C07 or C09A with indication code 430 (for heart failure) | 7,468 | 1.6 | (1.7) | 3,127 | 1.0 | (2.0) | 311 | 2.8 | (1.2) | 2.9 | 2.3 |  | 3.3 |  | 0.3 |  | 2.7 |  | 7.5 |  |
|  | Ischaemic heart medication ^c^ ** | ATC: C01A, C01B, C01D, C01E. | 129,484 | 28.4 | (28.5) | 57,571 | 13.5 | (35.2) | 4,324 | 51.0 | (20.9) | 3.8 | 54.6 |  | 47.0 |  | 2.4 |  | 45.3 |  | 152.3 |  |
|  | **All of the five types of medicine above** |  | **688,006** | **151.0** | **(151.6)** | **262,536** | **88.1** | **(92.4)** | **28,310** | **232.6** | **(211.0)** | **2.6** | **272.6** |  | **188.2** |  | **194.2** |  | **215.1** |  | **349.1** |  |
|  |  |  |  |  |  |  |  |  |  |  |  |  |  |  |  |  |  |  |  |  |  |  |
|  | **Extra** |  |  |  |  |  |  |  |  |  |  |  |  |  |  |  |  |  |  |  |  |  |
|  | Ischaemic Heart Diseases | I05-I06; I11-I13; I20-I28; I30-I52 | 315,901 | 69.3 | (69.3) | 120,791 | 107.0 | (78.8) | 15,151 | 47.2 | (58.6) | *2.3* | 106.9 |  | 107.2 |  | 18.2 |  | 104.7 |  | 269.9 |  |
|  | Artritis | M01-M03; M5-M9; M7-M14; M15-M20; M45 | 505,792 | 111.0 | (111.0) | 175,514 | 155.5 | (120.9) | 23,321 | 72.6 | (87.3) | *2.1* | 183.1 |  | 124.9 |  | 34.0 |  | 183.2 |  | 292.1 |  |
|  | Arthrosis | M15-M19 | 338,166 | 74.2 | (74.2) | 125,325 | 111.0 | (81.8) | 12,528 | 39.0 | (53.3) | *2.8* | 134.6 |  | 84.8 |  | 14.6 |  | 128.7 |  | 231.3 |  |
|  | Back conditions | M32-34; M41-M43; M46-49; M50-51; M53-M54 | 212,948 | 46.7 | (46.7) | 70,622 | 62.6 | (55.8) | 9,814 | 30.5 | (31.1) | *2.0* | 70.6 |  | 53.6 |  | 36.2 |  | 72.0 |  | 82.8 |  |
|  | Overweight | E66 | 220,928 | 48.5 | (48.5) | 68,152 | 60.4 | (67.3) | 8,875 | 27.6 | (14.7) | *2.2* | 88.5 |  | 29.2 |  | 72.1 |  | 62.8 |  | 33.0 |  |
|  | Endometriosis | N80 | 29,290 | 6.4 | (6.4) | 6,205 | 5.5 | (6.7) | 2,100 | 6.5 | (5.1) | *0.8* | 10.5 |  | 0.0 |  | 5.8 |  | 7.0 |  | 0.9 |  |
|  |  |  |  |  |  |  |  |  |  |  |  |  |  |  |  |  |  |  |  |  |  |  |

Age and gender standardised estimates are in brackets. Gender and age disease prevalence are/cannot be standardised.

ICD-10 International Statistical Classification of Diseases, 10^th^ Revision.

^c^ = complex defined conditions, see reference for further details [55].

* Total population frequencies and prevalence adapted from Hvidberg et al. 2019 [56].

** 2-year prevalence.

***S2 Table.*** Overview of the 50 conditions with the highest proportions of patients with no educational levels. Sorted ascending by the highest proportions of no education within the disease. N and prevalence.

|  |  | **N, Prevalence and Ratio** | | | | | | | | | | | | | |
| --- | --- | --- | --- | --- | --- | --- | --- | --- | --- | --- | --- | --- | --- | --- | --- |
| **Name of condition** | **ICD-10 code / definition** | **Total Population*** | | | **Disease prevalence by high and no education** | | | | | | | **Prevalence of education *within* disease** | | | |
|  |  |  |  |  | No Education  or Training | | | Higher (MSc degree or doctorate) | | |  | No Education  or Training | | Higher (MSc degree or doctorate) | |
|  |  | N | Per thousand | | **N** | Per thousand | | N | Per thousand | | *Ratio* | Per cent | | | |
| Mental retardation | F70–F79 | 13,822 | 3.0 | (3.1) | 11,095 | 9.8 | (10.6) | 39 | 0.1 | N/A | *81.0* | 80.3 | (81.9) | 0.3 | (0.4) |
| Disorders of psychological development | F80–F89 | 9,911 | 2.2 | (2.2) | 5,937 | 5.3 | (5.9) | 119 | 0.4 | (0.7) | *14.2* | 59.9 | (65.0) | 1.2 | (2.2) |
| Schizophrenia c | F20 | 29,422 | 6.5 | (6.5) | 15,920 | 14.1 | (15.3) | 776 | 2.4 | (0.8) | *5.8* | 54.1 | (55.1 | 2.6 | (2.5) |
| Bronchitis, not specified as acute | J40–J42 | 12,790 | 2.8 | (2.8) | 6,448 | 5.7 | (4.5) | 277 | 0.9 | (1.4) | *6.6* | 50.4 | (43.9) | 2.2 | (2.9) |
| Dementia ^c^ | F00, G30, F01, F02.0, F03.9, G31.8B, G31.8E, G31.9, G31.0B | 36803 | 8.1 | (8.1) | 18,319 | 16.2 | (8.8) | 1,066 | 3.3 | (7.5) | *4.9* | 49.8 | (44.7) | 2.9 | (6.9) |
| Emotionally unstable personality disorder | F603 | 21,848 | 4.8 | (4.9) | 10,738 | 9.5 | (11.3) | 587 | 1.8 | (0.3) | *5.2* | 49.1 | (49.4) | 2.7 | (2.8) |
| Mental and behavioural disorders due to psychoactive substance use | F11–F19 | 53,669 | 11.8 | (11.9) | 26,234 | 23.2 | (25.0) | 956 | 3.0 | (1.6) | *7.8* | 48.9 | (48.4) | 1.8 | (1.9) |
| Organic, incl. symptomatic, mental disorders | F04–F09 | 26,430 | 5.8 | (5.9) | 12,791 | 11.3 | (9.4) | 890 | 2.8 | (3.3) | *4.1* | 48.4 | (51.2) | 3.4 | (3.1) |
| Schizotypal and delusional disorders | F21–F29 | 39,694 | 8.7 | (8.8) | 18,976 | 16.8 | (17.8) | 1,513 | 4.7 | (3.2) | *3.6* | 47.8 | (48.5) | 3.8 | (3.7) |
| Atherosclerosis | I70 | 32,064 | 7.0 | (7.0) | 15,289 | 13.5 | (9.9) | 705 | 2.2 | (3.8) | *6.2* | 47.7 | (41.0) | 2.2 | (3.0) |
| Cerebral palsy and other paralytic syndromes | G80–G83 | 14,410 | 3.2 | (3.2) | 6,846 | 6.1 | (6.2) | 540 | 1.7 | (1.4) | *3.6* | 47.5 | (48.2) | 3.7 | (3.6) |
| Parkinson’s disease ^c^ | G20, G21, G22, F02.3 | 57,583 | 12.6 | (12.6) | 27,135 | 24.0 | (20.5) | 1,899 | 5.9 | (6.9) | *4.1* | 47.1 | (51.2) | 3.3 | (2.9) |
| Chronic viral hepatitis | B18 | 4,584 | 1.0 | (1.0) | 2,133 | 1.9 | (2.1) | 160 | 0.5 | (0.1) | *3.8* | 46.5 | (43.6) | 3.5 | (3.1) |
| Hyperkinetic disorders (ADHD) ^c^ | F90 | 42,908 | 9.4 | (9.5) | 19,548 | 17.3 | (19.7) | 1,048 | 3.3 | (3.6) | *5.3* | 45.6 | (38.7) | 2.4 | (5.6) |
| Behavioural and emotional disorders with onset usually occurring in childhood and adolescence | F91–F99 | 39,602 | 8.7 | (8.8) | 17,990 | 15.9 | (17.6) | 1,295 | 4.0 | (3.1) | *4.0* | 45.4 | (44.2) | 3.3 | (3.6) |
| Emphysema | J43 | 5,557 | 1.2 | (1.2) | 2,489 | 2.2 | (1.8) | 153 | 0.5 | (0.6) | *4.6* | 44.8 | (40.4) | 2.8 | (3.1) |
| Other peripheral vascular diseases | I73 | 28,508 | 6.3 | (6.3) | 12,644 | 11.2 | (8.5) | 746 | 2.3 | (3.4) | *4.8* | 44.4 | (33.8) | 2.6 | (3.9) |
| Other anaemias | D64 | 46,613 | 10.2 | (10.3) | 20,597 | 18.3 | (13.1) | 1,478 | 4.6 | (7.0) | *4.0* | 44.2 | (37.6) | 3.2 | (3.9) |
| Presbycusis (age–related hearing loss) | H911 | 80,659 | 17.7 | (17.6) | 35,451 | 31.4 | (17.1) | 2,892 | 9.0 | (16.5 | *3.5* | 44.0 | (31.9) | 3.6 | (5.3) |
| Sequelae of cerebrovascular disease | I69 | 50,952 | 11.2 | (11.2) | 22,350 | 19.8 | (14.0) | 1,660 | 5.2 | (7.8) | *3.8* | 43.9 | (39.9) | 3.3 | (3.1) |
| Diseases of the eye lens (cataracts) | H25–H28 | 68,009 | 14.9 | (15.1) | 29,764 | 26.4 | (16.3) | 2,743 | 8.5 | (14.3) | *3.1* | 43.8 | (32.3) | 4 | (5.2) |
| Epilepsy ^c^ | G40–G41 | 61,695 | 13.5 | (13.6) | 26,815 | 23.8 | (23.1) | 2,194 | 6.8 | (6.1) | *3.5* | 43.5 | (43.3) | 3.6 | (3.4) |
| Aortic and mitral valve disease c | I05, I06, I34, I35 | 30,123 | 6.6 | (6.6) | 13,077 | 11.6 | (7.7) | 1,314 | 4.1 | (6.0) | *2.8* | 43.4 | (31.3) | 4.4 | (6.1) |
| Specific personality disorders | F602, F604–F609 | 50,415 | 11.1 | (11.2) | 21,873 | 19.4 | (22.0) | 1,876 | 5.8 | (2.6) | *3.3* | 43.4 | (45.5) | 3.7 | (3.4) |
| Osteoporosis ^c^ | M80–M81 | 158,813 | 34.9 | (34.8) | 68,692 | 60.9 | (36.0) | 5,055 | 15.7 | (33.2) | *3.9* | 43.3 | (33.1) | 3.2 | (5.9) |
| Blindness and partial sight | H54 | 6,614 | 1.5 | (1.5) | 2,855 | 2.5 | (2.0) | 277 | 0.9 | (1.1) | *2.9* | 43.2 | (43.3) | 4.2 | (4.6) |
| Disorders of adult personality and behaviour | F61–F69 | 17,533 | 3.8 | (3.9) | 7,571 | 6.7 | (7.7) | 665 | 2.1 | (0.8) | *3.2* | 43.2 | (45.0) | 3.8 | (3.5) |
| Chronic renal failure (CRF) ^c^ | N18 | 20,162 | 4.4 | (4.5) | 8,621 | 7.6 | (5.9) | 713 | 2.2 | (2.8) | *3.4* | 42.8 | (36.2) | 3.5 | (4.0) |
| Coxarthrosis [arthrosis of hip] | M16 | 104,115 | 22.9 | (22.7 | 44,227 | 39.2 | (26.4) | 3,824 | 11.9 | (18.8) | *3.3* | 42.5 | (30.6) | 3.7 | (4.7) |
| Malignant neoplasm of bronchus and lung | C34 | 14,762 | 3.2 | (3.3) | 6,254 | 5.5 | (4.2) | 482 | 1.5 | (2.1) | *3.7* | 42.4 | (35.6) | 3.3 | (3.8) |
| Heart failure ^c^ | I11.0, I13.0, I13.2, I42.0, I42.6, I42.7, I42.9, I50.0, I50.1, I50.9 | 37,540 | 8.2 | (8.3) | 15,906 | 14.1 | (10.2) | 1,482 | 4.6 | (6.1) | *3.1* | 42.4 | (37.3) | 3.9 | (4.1) |
| Diabetes type 2 ^c^ | E11 | 242,177 | 53.2 | (53.3) | 102,366 | 90.7 | (73.6) | 7,735 | 24.1 | (29.1) | *3.8* | 42.3 | (38.9) | 3.2 | (3.4) |
| Chronic ischaemic heart disease | I25 | 84,592 | 18.6 | (18.6) | 35,570 | 31.5 | (23.3) | 3,073 | 9.6 | (12.5) | *3.3* | 42.0 | (37.5) | 3.6 | (3.6) |
| Mental and behavioural disorders due to use of alcohol | F10 | 59,143 | 13 | (13.2) | 24,641 | 21.8 | (22.5) | 1,701 | 5.3 | (3.9) | *4.1* | 41.7 | (42.7) | 2.9 | (2.5) |
| Stroke | I60, I61,I63–I64, Z501 (rehabilitation) | 72,606 | 15.9 | (16.0) | 29,883 | 26.5 | (19.2) | 2,783 | 8.7 | (11.7) | *3.1* | 41.2 | (35.6) | 3.8 | (3.8) |
| Chronic obstructive lung disease (COPD) ^c^ | J44, J96, J13–J18 | 216,184 | 47.5 | (47.6) | 88,085 | 78.0 | (64.7) | 7,600 | 23.7 | (30.1) | *3.3* | 40.7 | (32.8) | 3.5 | (4.6) |
| Rheumatism, unspecified | M790 | 6,852 | 1.5 | (1.5) | 2,786 | 2.5 | (2.4) | 188 | 0.6 | (0.6) | *4.2* | 40.7 | (37.0) | 2.7 | (3.4) |
| Aplastic and other anaemias | D60–D63 | 14,918 | 3.3 | (3.3) | 6,047 | 5.4 | (3.9) | 614 | 1.9 | (2.5) | *2.8* | 40.5 | (33.2) | 4.1 | (4.7) |
| Systemic atrophies primarily affecting the central nervous system and other degenerative diseases | G10–G14, G30–G32 | 10,401 | 2.3 | (2.3) | 4,214 | 3.7 | (2.5) | 538 | 1.7 | (2.3) | *2.2* | 40.5 | (36.3) | 5.2 | (6.2) |
| Other retinal disorders | H35 | 68,485 | 15 | (15.1) | 27,739 | 24.6 | (14.3) | 3,189 | 9.9 | (15.6) | *2.5* | 40.5 | (28.7) | 4.7 | (7.1) |
| Acute myocardial infarction and | I21–I22 | 36,654 | 8 | (8.1) | 14,841 | 13.2 | (10.2) | 1,310 | 4.1 | (4.9) | *3.2* | 40.5 | (39.1) | 3.6 | (3.1) |
| Mood (affective) disorders | F340, F348–F349, F38–F39 | 6,887 | 1.5 | (1.5) | 2,790 | 2.5 | (2.5) | 271 | 0.8 | (0.7) | *2.9* | 40.5 | (41.3) | 3.9 | (3.7) |
| Phobic anxiety disorders | F40 | 14,324 | 3.1 | (3.2) | 5,804 | 5.1 | (6.1) | 630 | 2.0 | (1.1) | *2.6* | 40.5 | (41.1) | 4.4 | (3.9) |
| Ischaemic heart diseases | I20–I25 | 139,173 | 30.6 | (30.7) | 56,244 | 49.8 | (37.7) | 5,320 | 16.6 | (20.9) | *3.0* | 40.4 | (36.8) | 3.8 | (3.8) |
| Atrioventricular and left bundle branch block | I44 | 14,604 | 3.2 | (3.2) | 5,903 | 5.2 | (3.4) | 677 | 2.1 | (2.9) | *2.5* | 40.4 | (31.2) | 4.6 | (5.5) |
| AMI complex/other | I23–I24 | 2,969 | 0.7 | (0.7) | 1,194 | 1.1 | (0.8) | 97 | 0.3 | (0.4) | *3.5* | 40.2 | (35.4) | 3.3 | (3.0) |
| Pulmonary heart disease and diseases of pulmonary circulation | I26–I28 | 15,352 | 3.4 | (3.4) | 6,122 | 5.4 | (4.1) | 641 | 2.0 | (2.6) | *2.7* | 39.9 | (33.2) | 4.2 | (5.0) |
| Atrial fibrillation and flutter | I48 | 112,342 | 24.7 | (24.7) | 44,765 | 39.7 | (25.8) | 5,768 | 18.0 | (24.5) | *2.2* | 39.8 | (29.8) | 5.1 | (6.2) |
| Osteoporosis in diseases classified elsewhere | M82 | 1,007 | 0.2 | (0.2) | 401 | 0.4 | (0.3) | 42 | 0.1 | (0.2) | *2.7* | 39.8 | (36.3) | 4.2 | (4.8) |
| Polyarthrosis [arthrosis] | M15 | 16,935 | 3.7 | (3.7) | 6,680 | 5.9 | (3.9) | 545 | 1.7 | (3.1) | *3.5* | 39.5 | (29.0) | 3.2 | (4.1) |
|  |  |  |  |  |  |  |  |  |  |  |  |  |  |  |  |
| **Total** |  | **4,555,439** | **-** | **-** | **1,128,588** | **1,000** | **N/A** | **321,254** | **1,000** | **N/A** | ***1.0*** | **24.8** | (**24.8)** | **7.1** | (**7.1)** |

Age and gender standardised estimates in brackets.

ICD-10 International Statistical Classification of Diseases, 10^th^ Revision.

^c^ = complex defined conditions, see reference for further details [55].

* Total population frequencies and prevalence adapted from Hvidberg et al. 2019 [56].

***S3 Table.*** Catalogue of socioeconomic positions for 199+ chronic conditions: number of patients treated, the prevalence rate in per hundred (per cent within conditions), in Denmark on 1 January 2013.

|  |  |  |  | **Prevalence of socioeconomic positions within diseases** | | | | | | | | | | | | | | | | | | | |
| --- | --- | --- | --- | --- | --- | --- | --- | --- | --- | --- | --- | --- | --- | --- | --- | --- | --- | --- | --- | --- | --- | --- | --- |
| **No.** | **Name of condition** | **ICD-10 code / definition** |  | **Retired, age** | | **Retirement, early volunteer** | | **Early retirement, health** | | **Seekleave, leave mv.** | | **Un-employed, social benefits** | | **Un-employed min. 6 months** | | **In training, education** | | **Employed** | | **Others not in workforce** | | **Missing** | |
|  |  |  | N | % | Std | % | Std | % | Std | % | Std | % | Std | % | Std | % | Std | % | Std | % | Std | % | Std |
|  | **B – Viral hepatitis and human immunodeficiency virus [HIV] disease** | **B18, B20–B24** | **8,500** | **6.0** | **(19.0)** | **1.0** | (**1.0)** | **28.3** | (**19.7)** | **2.2** | (**1.7)** | **11.4** | (**10.9)** | **2.9** | (**2.1)** | **1.9** | (**6.0)** | **40.7** | (**33.4)** | **4.9** | (**5.4)** | **0.7** | (**0.8)** |
| 1 | Chronic viral hepatitis | B18 | 4,584 | 4.7 | (18.7) | 0.9 | (1.1) | 36.7 | (24.9) | 3.2 | (2.3) | 15.6 | (14.3) | 2.5 | (1.9) | 2.1 | (6.1) | 29.1 | (25.2) | 4.7 | (4.9) | 0.5 | (0.7) |
| 2 | Human immunodeficiency virus [HIV] disease | B20–24 | 4,229 | 7.2 | (19.8) | 1.0 | (0.8) | 20.3 | (14.7) | 1.2 | (1.0) | 7.0 | (6.7) | 3.3 | (2.4) | 1.6 | (5.7) | 52.4 | (41.7) | 5.1 | (6.4) | 0.8 | (0.7) |
|  | **C – Malignant neoplasms** | **C00–C99; D32–D33; D35.2–D35.4; D42–D44** | **229,331** | **53.7** | **(19.9)** | **4.2** | (**2.8)** | **7.0** | (**8.4)** | **0.9** | (**1.8)** | **1.1** | (**3.0)** | **0.7** | (**1.3)** | **0.6** | (**6.9)** | **30.5** | (**53.1)** | **1.2** | (**2.5)** | **0.1** | (**0.3)** |
| 3 | Malignant neoplasms of other and unspecified localizations | C00–C14; C30–C33; C37–C42; C45–C49; C69; C73–74; C754–C759 | 20,557 | 43.1 | (19.9) | 4.2 | (2.5) | 10.6 | (10.1) | 1.3 | (1.9) | 1.9 | (3.5) | 1.1 | (1.6) | 1.1 | (7.7) | 35.0 | (49.6) | 1.7 | (2.8) | 0.1 | (0.3) |
| 4 | Malignant neoplasms of digestive organs | C15–C17; C22–C26 | 4,839 | 59.0 | (20.3) | 4.4 | (2.6) | 10.8 | (15.0) | 0.9 | (1.2) | 1.2 | (3.5) | 0.6 | (1.2) | 0.3 | (7.9) | 21.7 | (45.6) | 1.0 | (2.5) | 0.1 | (0.2) |
| 5 | Malignant neoplasm of colon | C18 | 18,826 | 70.9 | (20.1) | 3.5 | (2.9) | 4.6 | (7.8) | 0.6 | (2.3) | 0.6 | (3.6) | 0.5 | (1.4) | 0.1 | (7.1) | 18.3 | (51.8) | 0.9 | (2.9) | 0.0 | (0.0) |
| 6 | Malignant neoplasms of rectosigmoid junction, rectum, anus and anal canal | C19–C21 | 10,680 | 65.3 | (19.9) | 3.9 | (2.7) | 5.6 | (8.3) | 0.7 | (3.9) | 0.6 | (5.3) | 0.5 | (0.9) | 0.0 | (4.3) | 22.6 | (52.6) | 0.8 | (2.0) | 0.1 | (0.2) |
| 7 | Malignant neoplasm of bronchus and lung | C34 | 14,762 | 61.1 | (20.4) | 4.4 | (2.7) | 11.7 | (14.7) | 0.9 | (2.2) | 1.2 | (6.7) | 0.4 | (1.4) | 0.1 | (6.0) | 19.2 | (43.7) | 1.0 | (2.2) | 0.1 | (0.1) |
| 8 | Malignant melanoma of skin | C43 | 19,636 | 38.4 | (19.5) | 3.9 | (2.8) | 3.7 | (3.4) | 0.9 | (1.1) | 0.8 | (1.6) | 1.1 | (1.4) | 1.0 | (5.6) | 48.9 | (62.7) | 1.2 | (1.7) | 0.1 | (0.2) |
| 9 | Other malignant neoplasms of skin | C44 | 15,597 | 68.2 | (19.9) | 3.2 | (2.8) | 3.9 | (7.2) | 0.3 | (1.1) | 0.3 | (1.8) | 0.5 | (1.0) | 0.1 | (7.7) | 22.7 | (56.5) | 0.8 | (2.0) | 0.0 | (0.0) |
| 10 | Malignant neoplasm of breast | C50 | 50,687 | 52.6 | (25.5) | 5.7 | (3.2) | 7.5 | (8.0) | 0.9 | (2.0) | 0.8 | (2.6) | 0.7 | (0.7) | 0.1 | (1.6) | 30.2 | (49.6) | 1.5 | (1.3) | 0.1 | (0.1) |
| 11 | Malignant neoplasms of female genital organs | C51–C52; C56–C58 | 7,245 | 45.1 | (30.5) | 4.9 | (5.1) | 9.9 | (7.5) | 1.2 | (0.8) | 1.4 | (1.3) | 0.9 | (7.8) | 0.6 | (5.1) | 34.1 | (21.3) | 1.8 | (1.0) | 0.1 | (0.1) |
| 12 | Malignant neoplasm of cervix uteri, corpus uteri and part unspecified | C53–C55 | 11,608 | 49.0 | (9.1) | 5.0 | (1.6) | 8.0 | (4.0) | 0.9 | (0.8) | 1.5 | (1.9) | 0.8 | (0.6) | 0.2 | (0.8) | 32.9 | (28.7) | 1.5 | (1.7) | 0.1 | (0.1) |
| 13 | Malignant tumour of male genitalia | C60, C62–C63 | 5,194 | 12.0 | (25.6) | 1.6 | (1.0) | 6.1 | (9.8) | 1.0 | (0.5) | 2.4 | (1.1) | 2.0 | (0.9) | 1.9 | (3.0) | 70.8 | (36.9) | 1.9 | (1.3) | 0.3 | (0.1) |
| 14 | Malignant neoplasm of prostate | C61 | 26,697 | 75.5 | (26.0) | 3.5 | (5.4) | 2.0 | (9.4) | 0.2 | (0.3) | 0.2 | (3.9) | 0.3 | (0.4) | 0.0 | (0.0) | 17.8 | (34.4) | 0.4 | (0.4) | 0.1 | (0.1) |
| 15 | Malignant neoplasms of urinary tract | C64–C68 | 10,319 | 67.7 | (20.2) | 3.6 | (2.8) | 6.1 | (11.5) | 0.6 | (2.4) | 0.8 | (3.1) | (0.5 | (1.2) | 0.3 | (7.5) | 19.6 | (49.3) | 0.8 | (2.0) | 0.1 | (0.0) |
| 16 | Brain cancer ^c^ | C71, C75.1–C75.3, D33.0–D33.2, D35.2–D35.4, D43.0–D43.2, D44.3–D44.5 (brain). C70, D32, D42 (brain membrane). C72, D33.3–D33.9, D43.3–D43.9 (cranial nerve, spinal cord) | 15,310 | 38.8 | (20.1) | 3.5 | (2.3) | 14.1 | (15.7) | 1.6 | (2.2) | 2.2 | (4.0) | 0.9 | (1.1) | 1.7 | (7.1) | 35.3 | (44.3) | 1.9 | (3.0) | 0.2 | (0.3) |
| 17 | Malignant neoplasms of ill-defined, secondary and unspecified sites, and of independent (primary) multiple sites | C76–C80, C97 | 25,619 | 48.8 | (20.0) | 4.5 | (2.6) | 10.2 | (10.4) | 1.4 | (2.5) | 1.2 | (3.2) | 0.6 | (1.0) | 0.6 | (6.7) | 31.3 | (50.8) | 1.3 | (2.6) | 0.1 | (0.2) |
| 18 | Malignant neoplasms, stated or presumed to be primary, of lymphoid, haematopoietic and related tissue | C81–C96 | 19,712 | 49.7 | (20.0) | 3.6 | (2.7) | 8.0 | (9.7) | 1.1 | (2.1) | 1.5 | (3.3) | 0.7 | (1.2) | 1.9 | (7.4) | 32.0 | (50.7) | 1.3 | (2.5) | 0.2 | (0.4) |
|  | **D – In situ and benign neoplasms, and neoplasms of uncertain or unknown behaviour and diseases of the blood and blood-forming organs and certain disorders involving the immune mechanism** | **D00–D09; D55–D59; D60–D67; D80–D89** | **116,560** | **44.1** | (**20.3)** | **2.3** | (**2.1)** | **10.4** | (**12.6)** | **1.4** | (**2.0)** | **3.0** | (**4.6)** | **1.1** | (**1.5)** | **1.8** | (**6.7)** | **33.9** | (**47.0)** | **1.8** | (**3.0)** | **0.1** | (**0.2)** |
| 19 | In situ neoplasms | D00–D09 | 19,810 | 34.8 | (19.8) | 3.7 | (2.6) | 6.7 | (6.7) | 1.3 | (1.7) | 2.0 | (2.5) | 1.3 | (1.4) | 0.7 | (4.5) | 47.8 | (58.8) | 1.7 | (1.7) | 0.1 | (0.3) |
| 20 | Haemolytic anaemias | D55–D59 | 3,055 | 27.5 | (20.0) | 1.4 | (1.6) | 12.7 | (14.9) | 2.4 | (2.1) | 7.1 | (6.2) | 1.7 | (1.7) | 5.9 | (8.2) | 36.0 | (39.7) | 4.7 | (4.9) | 0.5 | (0.6) |
| 21 | Aplastic and other anaemias | D60–D63 | 14,918 | 60.1 | (20.5) | 2.0 | (2.0) | 10.8 | (18.7) | 1.1 | (2.2) | 2.6 | (6.3) | 0.8 | (1.5) | 0.9 | (7.6) | 20.4 | (37.7) | 1.4 | (3.4) | 0.1 | (0.2) |
| 22 | Other anaemias | D64 | 46,613 | 64.4 | (20.7) | 1.8 | (2.0) | 10.9 | (20.6) | 0.9 | (2.4) | 2.7 | (7.2) | 0.7 | (1.6) | 0.7 | (5.7) | 16.5 | (35.8) | 1.4 | (3.7) | 0.1 | (0.3) |
| 23 | Coagulation defects, purpura and other haemorrhagic conditions | D65–D69 | 25,376 | 22.4 | (19.9) | 2.0 | (2.0) | 11.3 | (11.3) | 2.0 | (1.8) | 3.6 | (3.6) | 1.5 | (1.5) | 4.2 | (6.9) | 50.4 | (50.2) | 2.3 | (2.6) | 0.2 | (0.2) |
| 24 | Other diseases of blood and blood-forming organs | D70–D77 | 8,896 | 35.7 | (20.1) | 2.7 | (1.9) | 14.7 | (14.4) | 2.0 | (2.7) | 3.5 | (5.2) | 1.4 | (1.8) | 1.8 | (6.9) | 35.7 | (43.7) | 2.3 | (3.1) | 0.1 | (0.1) |
| 25 | Certain disorders involving the immune mechanism | D80–D89 | 7,660 | 19.0 | (19.6) | 2.7 | (2.2) | 12.4 | (11.0) | 1.7 | (1.7) | 3.3 | (3.6) | 1.4 | (1.2) | 2.8 | (6.9) | 54.3 | (50.6) | 2.3 | (3.0) | 0.2 | (0.3) |
|  | **E – Endocrine, nutritional and metabolic diseases** | **E00–E14; E20–E29; E31–35; E70–E78; E84–E85; E88–E89** | **877,433** | **48.5** | (**20.0)** | **4.5** | (**2.8)** | **9.0** | (**10.5)** | **0.9** | (**1.8)** | **1.6** | (**4.0)** | **1.1** | (**1.8)** | **0.8** | (**7.2)** | **32.0** | (**48.7)** | **1.5** | (**2.9)** | **0.1** | (**0.3)** |
| 26 | Diseases of the thyroid ^c^ | E00–E04, E06, E07 | 131,908 | 39.5 | (19.9) | 3.9 | (2.5) | 9.3 | (10.3) | 1.2 | (1.7) | 2.0 | (3.4) | 1.3 | (1.8) | 1.3 | (7.2) | 39.4 | (50.0) | 1.9 | (2.9) | 0.2 | (0.4) |
| 27 | Thyrotoxicosis ^c^ | E05 | 41,374 | 44.4 | (20.0) | 3.5 | (2.6) | 7.6 | (8.3) | 1.3 | (2.0) | 2.1 | (3.4) | 1.3 | (1.9) | 1.2 | (7.2) | 36.9 | (51.1) | 1.8 | (3.3) | 0.1 | (0.3) |
| 28 | Diabetes type 1 ^c^ | E10 | 23,062 | 16.2 | (20.0) | 2.2 | (2.1) | 11.5 | (10.6) | 1.6 | (1.6) | 3.2 | (3.2) | 2.0 | (1.7) | 6.5 | (7.8) | 53.8 | (50.0) | 2.8 | (2.8) | 0.3 | (0.3) |
| 29 | Diabetes type 2 ^c^ | E11 | 242,177 | 52.7 | (20.2) | 4.2 | (2.6) | 11.7 | (15.4) | 1.0 | (2.4) | 2.1 | (7.1) | 1.3 | (2.3) | 0.1 | (4.2) | 25.4 | (42.5) | 1.4 | (3.1) | 0.1 | (0.2) |
| 30 | Diabetes others ^c^ | E12–E14 | 1,117 | 36.5 | (20.6) | 3.1 | (2.5) | 11.9 | (12.9) | 2.5 | (3.4) | 4.9 | (6.6) | 2.6 | (3.2) | 4.5 | (9.3) | 31.4 | (37.7) | 2.7 | (3.3) | 0.0 | (0.4) |
| 31 | Disorders of other endocrine glands | E20–E35, except E30 | 28,650 | 25.0 | (20.0) | 2.2 | (2.4) | 11.3 | (13.5) | 2.2 | (1.9) | 4.2 | (4.2) | 1.7 | (1.7) | 6.8 | (8.0) | 43.4 | (45.0) | 2.9 | (3.0) | 0.2 | (0.2) |
| 32 | Metabolic disorders | E70–E77; E79–E83; E85, E88–E89; | 23,690 | 29.6 | (20.1) | 2.5 | (2.0) | 14.8 | (15.2) | 1.7 | (1.9) | 3.8 | (4.7) | 1.6 | (1.8) | 3.0 | (6.5) | 40.3 | (44.6) | 2.5 | (2.9) | 0.2 | (0.3) |
| 33 | Disturbances in lipoprotein circulation and other lipids ^c^ | E78 | 652,242 | 54.6 | (20.0) | 5.1 | (2.9) | 8.7 | (13.1) | 0.7 | (2.0) | 1.1 | (4.9) | 0.9 | (1.8) | 0.1 | (5.4) | 27.5 | (47.2) | 1.2 | (2.6) | 0.1 | (0.2) |
| 34 | Cystic fibrosis ^c^ | E84 | 947 | 10.4 | (20.6) | 1.1 | (1.6) | 15.6 | (18.2) | 2.2 | (1.8) | 4.0 | (3.6) | 1.6 | (1.2) | 10.7 | (8.4) | 51.9 | (42.2) | 2.6 | (2.1) | 0.0 | (0.2) |
|  | **G – Diseases of the nervous system** | **G00–G14; G20–G32; G35–G37; G40–47; G50–64; G70–73; G80–G83; G90–G99** | **561,054** | **29.0** | (**20.0)** | **3.0** | (**2.1)** | **15.3** | (**15.1)** | **1.6** | (**1.9)** | **3.3** | (**4.7)** | **1.4** | (**1.5)** | **1.8** | (**6.1)** | **42.4** | (**45.6)** | **2.0** | (**2.8)** | **0.1** | (**0.2)** |
| 35 | Inflammatory diseases of the central nervous system | G00–G09 | 7,642 | 26.8 | (20.0) | 2.1 | (1.8) | 17.2 | (16.7) | 1.2 | (1.2) | 3.1 | (3.8) | 1.1 | (1.2) | 2.0 | (5.3) | 44.1 | (46.8) | 2.2 | (2.9) | 0.2 | (0.3) |
| 36 | Systemic atrophies primarily affecting the central nervous system and other degenerative diseases | G10–G14, G30–G32 | 10,401 | 67.7 | (20.8) | 1.5 | (1.4) | 18.3 | (39.2) | 0.5 | (1.2) | 1.1 | (3.9) | 0.3 | (0.7) | 0.6 | (6.0) | 9.3 | (24.8) | 0.6 | (1.7) | 0.0 | (0.2) |
| 37 | Parkinson’s disease ^c^ | G20, G21, G22, F02.3 | 57,583 | 47.7 | (20.6) | 1.9 | (1.4) | 31.2 | (44.4) | 0.7 | (1.3) | 1.9 | (7.5) | 0.5 | (0.7) | 0.3 | (2.4) | 14.6 | (18.8) | 1.1 | (2.7) | 0.1 | (0.1) |
| 38 | Extrapyramidal and movement disorders | G23–G26 | 10,837 | 43.6 | (20.4) | 2.3 | (1.7) | 18.4 | (22.7) | 1.3 | (2.1) | 2.9 | (5.4) | 1.1 | (1.5) | 2.0 | (6.6) | 26.5 | (36.5) | 1.8 | (3.0) | 0.1 | (0.1) |
| 39 | Sclerosis | G35 | 13,284 | 17.7 | (20.5) | 1.3 | (0.9) | 36.4 | (26.9) | 1.3 | (1.9) | 1.8 | (2.9) | 0.7 | (1.0) | 1.1 | (5.9) | 37.4 | (37.1) | 2.2 | (2.9) | 0.1 | (0.1) |
| 40 | Demyelinating diseases of the central nervous system | G36–G37 | 4,571 | 12.8 | (20.3) | 1.5 | (1.2) | 25.5 | (19.8) | 1.8 | (1.8) | 2.7 | (3.2) | 1.0 | (0.9) | 1.9 | (5.8) | 49.9 | (43.7) | 2.6 | (3.0) | 0.3 | (0.3) |
| 41 | Epilepsy ^c^ | G40–G41 | 61,695 | 28.4 | (20.6) | 1.6 | (1.3) | 33.2 | (34.1) | 1.4 | (1.6) | 4.5 | (5.7) | 1.1 | (1.2) | 3.3 | (6.2) | 23.9 | (26.2) | 2.4 | (3.0) | 0.1 | (0.2) |
| 42 | Migraine ^c^ | G43 | 149,866 | 14.4 | 19.3) | 3.2 | (2.1) | 11.6 | (8.8) | 1.9 | (1.8) | 3.7 | (4.0) | 1.7 | (1.5) | 2.5 | (6.5) | 58.4 | (53.2) | 2.4 | (2.5) | 0.2 | (0.2) |
| 43 | Other headache syndromes | G44 | 16,469 | 11.1 | (19.4) | 1.6 | (1.8) | 13.3 | (11.6) | 3.3 | (2.6) | 8.2 | (6.7) | 1.8 | (1.5) | 5.3 | (6.7) | 51.8 | (46.2) | 3.5 | (3.3) | 0.1 | (0.2) |
| 44 | Transient cerebral ischaemic attacks and related syndromes and vascular syndromes of brain in cerebrovascular diseases | G45–G46 | 43,977 | 60.3 | (20.1) | 3.6 | (2.5) | 7.6 | (10.8) | 0.7 | (2.0) | 1.2 | (5.2) | 0.7 | (1.6) | 0.1 | (4.9) | 24.8 | (50.4) | 1.0 | (2.3) | 0.1 | (0.2) |
| 45 | Sleep disorders | G47 | 36,806 | 21.2 | (19.5) | 3.8 | (2.3) | 11.5 | (11.0) | 1.9 | (2.2) | 3.0 | (5.0) | 2.0 | (1.8) | 1.5 | (6.6) | 53.0 | (48.6) | 2.1 | (3.0) | 0.1 | (0.1) |
| 46 | Disorders of trigeminal nerve and facial nerve disorders | G50–G51 | 21,488 | 29.8 | (19.9) | 2.9 | (2.2) | 11.6 | (10.3) | 1.4 | (1.7) | 3.1 | (4.2) | 1.6 | (1.8) | 1.5 | (5.8) | 45.8 | (50.9) | 2.1 | (2.8) | 0.2 | (0.4) |
| 47 | Disorders of other cranial nerves, cranial nerve disorders in diseases classified elsewhere, nerve root and plexus disorders and nerve root and plexus compressions in diseases classified elsewhere | G52–G55 | 12,429 | 28.7 | (20.0) | 3.1 | (2.0) | 16.1 | (13.1) | 2.3 | (3.1) | 3.5 | (5.5) | 1.4 | (1.5) | 0.7 | (5.0) | 41.7 | (46.8) | 2.3 | (2.8) | 0.1 | (0.1) |
| 48 | Mononeuropathies of upper limb | G56 | 122,395 | 27.4 | (19.7) | 4.2 | (2.5) | 11.9 | (9.0) | 2.1 | (2.8) | 3.5 | (5.0) | 1.8 | (1.9) | 0.6 | (4.4) | 46.2 | (51.8) | 2.1 | (2.7) | 0.1 | (0.2) |
| 49 | Mononeuropathies of lower limb, other mononeuropathies and mononeuropathy in diseases classified elsewhere | G57–G59 | 18,627 | 28.6 | (19.7) | 3.7 | (2.3) | 11.8 | (9.5) | 1.7 | (2.0) | 3.0 | (4.5) | 1.5 | (1.5) | 0.9 | (5.9) | 46.8 | (51.8) | 1.9 | (2.6) | 0.1 | (0.2) |
| 50 | Polyneuropathies and other disorders of the peripheral nervous system | G60–G64 | 30,289 | 47.1 | (20.2) | 2.9 | (1.9) | 16.5 | (18.3) | 1.5 | (2.6) | 2.6 | (5.7) | 1.0 | (1.6) | 0.7 | (6.2) | 26.0 | (40.1) | 1.6 | (3.1) | 0.1 | (0.2) |
| 51 | Diseases of myoneural junction and muscle | G70–G73 | 5,758 | 26.6 | (20.0) | 2.0 | (1.6) | 24.7 | (25.1) | 1.6 | (1.8) | 2.9 | (3.7) | 1.0 | (1.1) | 3.1 | (6.6) | 35.7 | (37.2) | 2.3 | (2.8) | 0.2 | (0.2) |
| 52 | Cerebral palsy and other paralytic syndromes | G80–G83 | 14,410 | 21.5 | (20.6) | 0.8 | (0.7) | 46.1 | (45.8) | 1.1 | (1.1) | 3.4 | (3.7) | 0.5 | (0.5) | 4.2 | (5.3) | 20.4 | (20.1) | 1.9 | (2.0) | 0.1 | (0.1) |
| 53 | Other disorders of the nervous system | G90–G99 | 44,394 | 33.1 | (20.2) | 2.3 | (1.8) | 18.7 | (19.7) | 1.8 | (2.2) | 3.9 | (5.5) | 1.2 | (1.4) | 2.3 | (5.9) | 34.2 | (39.9) | 2.2 | (3.2) | 0.1 | (0.2) |
|  | **H – Diseases of the eye and adnexa and diseases of the ear and mastoid process** | **H02–H06; H17–H18; H25–H28; H31–H32; H34–H36; H40–55; H57; H80,H810; H93, H90–H93** | **448,176** | **57.6** | (**20.1)** | **3.1** | (**2.6)** | **7.2** | (**11.2)** | **0.7** | (**1.6)** | **1.4** | (**3.7)** | **0.9** | (**1.7)** | **1.1** | (**6.9)** | **26.9** | (**49.3)** | **1.2** | (**2.8)** | **0.1** | (**0.2)** |
| 54 | Disorders of eyelid, lacrimal system and orbit | H02–H06 | 13,191 | 43.7 | (19.9) | 3.2 | (2.2) | 7.9 | (8.0) | 1.1 | (1.8) | 1.5 | (3.2) | 1.2 | (1.7) | 1.0 | (7.5) | 38.8 | (52.5) | 1.6 | (3.0) | 0.1 | (0.3) |
| 55 | Corneal scars and opacities | H17 | 2,173 | 40.0 | (19.6) | 2.5 | (2.2) | 9.9 | (12.1) | 1.1 | (1.9) | 2.9 | (4.4) | 1.5 | (1.7) | 1.4 | (4.9) | 38.7 | (49.5) | 2.1 | (3.6) | 0.0 | (0.2) |
| 56 | Other disorders of cornea | H18 | 9,473 | 48.8 | (19.9) | 2.6 | (2.4) | 6.9 | (9.9) | 0.9 | (1.4) | 2.0 | (4.1) | 1.1 | (1.7) | 1.4 | (6.1) | 34.8 | (51.7) | 1.3 | (2.4) | 0.2 | (0.4) |
| 57 | Diseases of the eye lens (cataracts) | H25–H28 | 68,009 | 79.4 | (20.4) | 2.6 | (2.8) | 4.7 | (16.9) | 0.3 | (2.0) | 0.7 | (5.1) | 0.4 | (1.9) | 0.1 | (7.2) | 11.3 | (40.9) | 0.6 | (2.8) | 0.0 | (0.1) |
| 58 | Disorders of the choroid and retina | H31–H32 | 1,900 | 41.6 | (20.2) | 2.3 | (2.0) | 8.4 | (9.9) | 1.0 | (1.5) | 2.3 | (3.9) | 1.3 | (1.6) | 1.4 | (5.0) | 40.2 | (53.4) | 1.5 | (2.3) | 0.0 | (0.2) |
| 59 | Retinal vascular occlusions | H34 | 10,358 | 69.5 | (20.2) | 3.0 | (2.7) | 5.3 | (9.2) | 0.4 | (1.4) | 0.7 | (4.1) | 0.6 | (1.6) | 0.2 | (6.4) | 19.3 | (50.4) | 0.9 | (3.7) | 0.0 | (0.2) |
| 60 | Other retinal disorders | H35 | 68,485 | 74.9 | (20.2) | 2.0 | (2.4) | 4.6 | (12.7) | 0.3 | (1.3) | 0.7 | (3.6) | 0.5 | (1.5) | 0.3 | (6.9) | 16.0 | (48.3) | 0.6 | (2.8) | 0.1 | (0.3) |
| 61 | Retinal disorders in diseases classified elsewhere | H36 | 19,279 | 38.5 | (20.2) | 3.6 | (2.2) | 15.9 | (15.5) | 1.3 | (1.9) | 2.6 | (4.4) | 1.4 | (1.7) | 1.1 | (6.0) | 33.7 | (44.9) | 1.8 | (3.0) | 0.1 | (0.2) |
| 62 | Glaucoma ^c^ | H40–H42 | 67,310 | 70.9 | (20.1) | 3.2 | (2.9) | 5.4 | (12.5) | 0.4 | (1.8) | 0.6 | (3.4) | 0.5 | (1.6) | 0.3 | (7.8) | 17.8 | (47.0) | 0.8 | (2.7) | 0.1 | (0.2) |
| 63 | Disorders of the vitreous body and globe | H43–H45 | 7,572 | 39.0 | (19.4) | 4.1 | (2.4) | 8.1 | (8.1) | 1.0 | (1.6) | 1.2 | (2.7) | 1.0 | (1.3) | 1.6 | (7.2) | 41.9 | (54.2) | 1.9 | (2.8) | 0.2 | (0.2) |
| 64 | Disorders of optic nerve and visual pathways | H46–H48 | 6,184 | 19.8 | (19.9) | 2.2 | (1.9) | 20.3 | (19.6) | 1.9 | (1.7) | (3.4 | (3.8) | 1.5 | (1.5) | 2.9 | (5.4) | 44.8 | (42.9) | 2.8 | (3.1) | 0.1 | (0.1) |
| 65 | Disorders of ocular muscles, binocular movement, accommodation and refraction | H49–H52 | 18,247 | 14.1 | (19.7) | 1.4 | (1.7) | 8.9 | (8.6) | 1.6 | (1.3) | 2.7 | (2.5) | 1.9 | (1.6) | 5.2 | (7.1) | 61.8 | (55.0) | 2.3 | (2.4) | 0.2 | (0.2) |
| 66 | Visual disturbances | H53 | 22,232 | 37.9 | (20.1) | 2.5 | (1.9) | 14.5 | (15.9) | 1.2 | (1.7) | 2.6 | (4.2) | 1.2 | (1.5) | 2.0 | (6.3) | 36.1 | (45.2) | 1.8 | (2.9) | 0.2 | (0.3) |
| 67 | Blindness and partial sight | H54 | 6,614 | 49.6 | (20.5) | 1.6 | (1.5) | 21.0 | (31.1) | 0.9 | (1.5) | 2.8 | (5.1) | 1.0 | (1.7) | 1.6 | (5.2) | 19.8 | (30.3) | 1.5 | (2.9) | 0.1 | (0.2) |
| 68 | Nystagmus and other irregular eye movements and other disorders of eye and adnexa | H55, H57 | 11,133 | 29.3 | (19.7) | 2.6 | (2.0) | 12.9 | (13.1) | 1.6 | (1.8) | 3.3 | (4.1) | 1.3 | (1.5) | 3.6 | (7.5) | 43.1 | (47.6) | 2.1 | (2.4) | 0.2 | (0.2) |
| 69 | Otosclerosis | H80 | 10,360 | 42.3 | (19.9) | 4.1 | (2.7) | 6.9 | (6.7) | 0.8 | (1.1) | 1.7 | (3.5) | 1.1 | (1.7) | 1.1 | (8.1) | 40.3 | (53.8) | 1.6 | (2.3) | 0.1 | (0.3) |
| 70 | Ménière’s disease ^c^ | H810 | 10,003 | 52.4 | (20.2) | 3.8 | (2.5) | 9.0 | (9.5) | 0.7 | (1.5) | 0.9 | (2.1) | 0.7 | (1.6) | 0.2 | (5.6) | 30.9 | (52.4) | 1.3 | (4.4) | 0.1 | (0.3) |
| 71 | Other diseases of the inner ear | H83 | 29,865 | 61.9 | (20.0) | 5.0 | (3.3) | 5.4 | (10.7) | 0.6 | (1.7) | 0.7 | (3.8) | 0.7 | (1.5) | 0.2 | (6.2) | 24.7 | (49.6) | 0.7 | (2.8) | 0.1 | (0.3) |
| 72 | Conductive and sensorineural hearing loss | H90 | 43,238 | 49.1 | (20.1) | 3.2 | (2.5) | 8.6 | (12.1) | 1.0 | (1.8) | 2.1 | (4.3) | 1.1 | (1.8) | 2.5 | (7.1) | 30.8 | (47.1) | 1.5 | (2.9) | 0.1 | (0.2) |
| 73 | Other hearing loss and other disorders of ear, not elsewhere classified | H910, H912, H913, H918, H930, H932, H933 | 8,306 | 45.2 | (20.1) | 3.1 | (2.3) | 11.7 | (15.7) | 0.9 | (1.5) | 2.6 | (5.7) | 1.3 | (1.8) | 1.7 | (6.4) | 31.7 | (43.0) | 1.8 | (3.5) | 0.1 | (0.1) |
| 74 | Presbycusis (age-related hearing loss) | H911 | 80,659 | 90.7 | (20.6) | 1.1 | (2.9) | 1.5 | (12.3) | 0.1 | (1.4) | 0.3 | (3.9) | 0.1 | (1.9) | 0.1 | (4.9) | 5.9 | (48.0) | 0.2 | (3.9) | 0.0 | (0.1) |
| 75 | Hearing loss, unspecified | H919 | 87,806 | 60.8 | (20.1) | 3.8 | (2.8) | 7.1 | (12.6) | 0.6 | (1.5) | 1.2 | (4.4) | 0.8 | (1.8) | 0.7 | (7.0) | 23.9 | (46.8) | 1.0 | (2.9) | 0.1 | (0.2) |
| 76 | Tinnitus | H931 | 40,124 | 40.3 | (19.9) | 4.6 | (2.7) | 10.2 | (9.7) | 1.0 | (1.5) | 1.9 | (3.8) | 1.3 | (1.9) | 0.7 | (7.5) | 38.5 | (50.3) | 1.5 | (2.5) | 0.1 | (0.2) |
| 77 | Other specified disorders of ear | H938 | 20,537 | 59.1 | (20.1) | 4.3 | (3.1) | 6.7 | (10.4) | 0.5 | (1.3) | 1.0 | (4.2) | 0.7 | (1.5) | 0.8 | (6.5) | 25.9 | (50.0) | 0.9 | (2.6) | 0.1 | (0.3) |
|  | **I – Diseases of the circulatory system** | **I05–I06; I10–28; I30–33;I36–141; I44–I52; I60–I88; I90–I94; I96–I99** | **1,254,427** | **46.7** | (**19.9)** | **4.1** | (**2.6)** | **8.0** | (**8.7)** | **1.0** | (**2.0)** | **1.7** | (**4.0)** | **1.2** | (**1.7)** | **0.6** | (**5.8)** | **35.0** | (**52.3)** | **1.4** | (**2.7)** | **0.1** | (**0.3)** |
| 78 | Aortic and mitral valve disease ^c^ | I05, I06, I34, I35 | 30,123 | 72.9 | (20.2) | 2.4 | (2.6) | 5.2 | (12.6) | 0.5 | (2.2) | 0.9 | (4.6) | 0.5 | (1.6) | 0.4 | (6.2) | 16.3 | (46.1) | 0.8 | (3.5) | 0.1 | (0.4) |
| 79 | Hypertensive diseases ^c^ | I10–I15 | 1,060,046 | 50.6 | (19.9) | 4.3 | (2.7) | 8.0 | (9.3) | 0.9 | (2.1) | 1.5 | (4.2) | 1.1 | (1.9) | 0.3 | (5.6) | 31.9 | (51.5) | 1.3 | (2.6) | 0.1 | (0.2) |
| 80 | Heart failure ^c^ | I11.0, I13.0, I13.2, I42.0, I42.6, I42.7, I42.9, I50.0, I50.1, I50.9 | 37,540 | 68.2 | (20.5) | 2.5 | (2.1) | 10.3 | (21.9) | 0.7 | (2.2) | 1.2 | (5.0) | 0.5 | (1.4) | 0.1 | (4.8) | 15.4 | (38.3) | 1.0 | (3.6) | 0.1 | (0.4) |
| 80A | Ischaemic heart diseases | I20–I25 | 139,173 | 59.2 | (20.2) | 3.6 | (2.4) | 9.5 | (13.6) | 0.8 | (2.6) | 1.5 | (6.5) | 0.8 | (1.6) | 0.1 | (4.3) | 23.2 | (44.6) | 1.2 | (4.0) | 0.1 | (0.2) |
| 81 | Angina pectoris | I20 | 78,476 | 54.1 | (20.1) | 4.0 | (2.4) | 10.9 | (13.5) | 1.0 | (2.9) | 1.8 | (6.6) | 0.9 | (1.6) | 0.1 | (3.7) | 25.8 | (44.5) | 1.3 | (4.4) | 0.1 | (0.2) |
| 82 | Acute myocardial infarction and subsequent myocardial infarction | I21–I22 | 36,654 | 57.1 | (20.2) | 3.8 | (2.6) | 8.7 | (12.2) | 1.0 | (2.3) | 1.6 | (6.8) | 1.0 | (2.0) | 0.1 | (3.7) | 25.5 | (46.8) | 1.1 | (3.3) | 0.1 | (0.1) |
| 83 | AMI complex/other | I23–I24 | 2,969 | 54.9 | (20.6) | 3.1 | (2.2) | 11.2 | (14.5) | 1.0 | (1.8) | 2.8 | (6.3) | 0.9 | (1.2) | 0.2 | (3.7) | 24.2 | (46.7) | 1.6 | (2.8) | 0.0 | (0.2) |
| 84 | Chronic ischaemic heart disease | I25 | 84,592 | 65.9 | (20.4) | 3.3 | (2.4) | 8.9 | (16.8) | 0.7 | (2.9) | 1.2 | (7.2) | 0.6 | (1.3) | 0.0 | (6.4) | 18.3 | (38.5) | 0.9 | (3.9) | 0.1 | (0.1) |
| 85 | Pulmonary heart disease and diseases of pulmonary circulation | I26–I28 | 15,352 | 56.6 | (20.4) | 2.9 | (2.5) | 9.6 | (14.4) | 1.3 | (2.6) | 2.2 | (5.5) | 0.8 | (1.4) | 1.2 | (6.3) | 24.1 | (43.8) | 1.2 | (2.8) | 0.1 | (0.2) |
| 86 | Acute pericarditis | I30 | 5,563 | 22.5 | (19.6) | 2.7 | (2.3) | 8.6 | (8.9) | 1.5 | (2.0) | 3.9 | (4.6) | 2.0 | (1.6) | 2.8 | (5.8) | 53.1 | (51.8) | 2.4 | (3.1) | 0.4 | (0.3) |
| 87 | Other forms of heart disease | I31–I43, except I34–I35 and I42 | 8,119 | 47.1 | (20.2) | 2.7 | (2.2) | 12.2 | (16.4) | 1.1 | (2.1) | 2.7 | (5.3) | 1.0 | (1.4) | 1.4 | (4.8) | 30.2 | (44.6) | 1.6 | (2.8) | 0.1 | (0.3) |
| 88 | Atrioventricular and left bundle branch block | I44 | 14,604 | 0.3 | (20.4) | 8.7 | (2.8) | 20.0 | (13.9) | 1.8 | (1.8) | 3.0 | (4.1) | 1.9 | (1.5) | 2.0 | (6.2) | 59.8 | (46.5) | 2.4 | (2.5) | 0.2 | (0.3) |
| 89 | Other conduction disorders | I45–46 | 11,823 | 51.5 | (20.3) | 2.5 | (2.3) | 9.0 | (12.2) | 1.1 | (1.9) | 2.2 | (4.7) | 0.9 | (1.6) | 1.7 | (6.6) | 29.5 | (47.3) | 1.4 | (2.8) | 0.1 | (0.4) |
| 90 | Paroxysmal tachycardia | I47 | 39,510 | 46.0 | (19.9) | 3.6 | (2.6) | 7.9 | (9.0) | 1.0 | (1.7) | 2.0 | (4.3) | 1.1 | (1.6) | 1.6 | (6.4) | 35.1 | (51.4) | 1.6 | (2.8) | 0.1 | (0.2) |
| 91 | Atrial fibrillation and flutter | I48 | 112,342 | 72.5 | (20.2) | 2.8 | (2.7) | 4.5 | (9.8) | 0.4 | (1.9) | 0.6 | (4.3) | 0.5 | (1.6) | 0.1 | (5.0) | 17.7 | (51.2) | 0.7 | (3.0) | 0.1 | (0.3) |
| 92 | Other cardiac arrhythmias | I49 | 34,418 | 54.1 | (20.0) | 2.9 | (2.4) | 6.4 | (8.3) | 0.9 | (1.9) | 1.4 | (3.9) | 0.8 | (1.5) | 0.8 | (6.0) | 31.3 | (53.1) | 1.2 | (2.6) | 0.1 | (0.3) |
| 93 | Complications and ill-defined descriptions of heart disease and other heart disorders in diseases classified elsewhere | I51–52 | 7,337 | 52.5 | (20.1) | 2.6 | (2.1) | 10.8 | (14.9) | 1.2 | (2.4) | 2.2 | (5.2) | 0.8 | (1.5) | 1.3 | (5.6) | 26.7 | (44.4) | 1.7 | (3.6) | 0.1 | (0.2) |
| 94 | Stroke | I60, I61,I63–I64, Z501 (rehabilitation) | 72,606 | 63.5 | (20.5) | 3.0 | (2.2) | 12.1 | (19.0) | 0.8 | (2.4) | 1.2 | (6.1) | 0.5 | (1.0) | 0.2 | (5.1) | 17.6 | (40.5) | 1.0 | (3.2) | 0.1 | (0.1) |
| 95 | Cerebrovascular diseases | I62, I65–I68 | 17,308 | 55.7 | (20.3) | 3.1 | (2.2) | 11.7 | (14.7) | 1.0 | (2.4) | 1.7 | (5.1) | 0.6 | (1.1) | 0.5 | (5.8) | 24.2 | (44.9) | 1.3 | (3.3) | 0.1 | (0.2) |
| 96 | Sequelae of cerebrovascular disease | I69 | 50,952 | 69.7 | (20.8) | 2.1 | (1.8) | 14.4 | (30.1) | 0.6 | (2.3) | 1.0 | (6.5) | 0.3 | (0.9) | 0.1 | (4.4) | 10.9 | (30.0) | 0.8 | (3.2) | 0.1 | (0.2) |
| 97 | Atherosclerosis | I70 | 32,064 | 72.0 | (20.7) | 2.9 | (2.4) | 10.3 | (21.7) | 0.5 | (1.6) | 1.1 | (7.7) | 0.5 | (1.1) | 0.1 | (4.8) | 11.7 | (36.9) | 0.8 | (2.9) | 0.0 | (0.2) |
| 98 | Aortic aneurysm and aortic dissection | I71 | 10,296 | 75.3 | (20.6) | 2.6 | (2.5) | 4.4 | (10.7) | 0.5 | (2.5) | 0.4 | (3.1) | 0.4 | (1.8) | 0.2 | (7.6) | 15.5 | (47.4) | 0.7 | (3.8) | 0.0 | (0.0) |
| 99 | Diseases of arteries, arterioles and capillaries | I72, I74, I77–I79 | 11,830 | 41.2 | (19.9) | 3.1 | (2.2) | 10.1 | (10.4) | 1.2 | (1.7) | 2.2 | (3.9) | 1.2 | (1.5) | 1.3 | (7.1) | 37.7 | (50.3) | 1.8 | (2.7) | 0.1 | (0.2) |
| 100 | Other peripheral vascular diseases | I73 | 28,508 | 65.0 | (20.7) | 3.8 | (2.8) | 9.2 | (13.1) | 1.0 | (2.9) | 1.6 | (4.7) | 0.8 | (1.6) | 0.5 | (7.5) | 17.1 | (43.5) | 1.0 | (3.0) | 0.1 | (0.1) |
| 101 | Phlebitis, thrombosis of the portal vein and others | I80–I82 | 37,388 | 43.1 | (20.0) | 3.0 | (2.4) | 10.8 | (12.2) | 1.5 | (2.3) | 2.9 | (5.3) | 1.3 | (1.7) | 1.2 | (5.7) | 34.5 | (47.4) | 1.6 | (2.9) | 0.1 | (0.2) |
| 102 | Varicose veins of lower extremities | I83 | 23,530 | 27.2 | (19.6) | 3.9 | (2.8) | 6.5 | (5.6) | 1.3 | (1.6) | 1.7 | (2.3) | 1.5 | (1.5) | 0.9 | (4.7) | 55.4 | (59.7) | 1.6 | (2.0) | 0.1 | (0.2) |
| 103 | Haemorrhoids ^c^ | I84 | 74,285 | 21.7 | (19.8) | 2.5 | (2.4) | 7.5 | (7.1) | 2.3 | (2.1) | 3.1 | (3.5) | 1.7 | (1.6) | 2.5 | (5.5) | 56.5 | (55.3) | 2.1 | (2.5) | 0.2 | (0.3) |
| 104 | Oesophageal varices (chronic), varicose veins of other sites, other disorders of veins, non-specific lymphadenitis, other non-infective disorders of lymphatic vessels and lymph nodes and other and unspecified disorders of the circulatory system | I85–I99, except I89 and I95 | 15,194 | 33.0 | (20.3) | 2.7 | (2.1) | 12.3 | (12.0) | 1.7 | (2.1) | 3.3 | (4.4) | 1.4 | (1.6) | 3.6 | (6.6) | 39.4 | (47.4) | 2.5 | (3.2) | 0.2 | (0.2) |
|  | **J – Diseases of the respiratory system** | **J30.1; J40–J47; J60–J84; J95, J97–J99** | **1,210,598** | **26.3** | (**19.8)** | **2.7** | (**2.3)** | **7.9** | (**7.4)** | **1.4** | (**1.4)** | **2.7** | (**3.0)** | **1.6** | (**1.7)** | **4.2** | (**7.4)** | **51.0** | (**54.3)** | **2.0** | (**2.4)** | **0.2** | (**0.3)** |
| 105 | Respiratory allergy ^c^ | J30, except J30.0 | 841,685 | 24.2 | (19.6) | 2.6 | (2.3) | 7.2 | (6.7) | 1.4 | (1.4) | 2.3 | (2.5) | 1.6 | (1.6) | 4.2 | (7.3) | 54.2 | (56.1) | 2.0 | (2.2) | 0.2 | (0.3) |
| 105A | Chronic lower respiratory diseases ^c^ | J40–J43, J47 | 418,120 | 30.5 | (20.0) | 2.7 | (2.1) | 10.2 | (9.1) | 1.4 | (1.6) | 2.9 | (3.6) | 1.6 | (1.7) | 1.9 | (6.9) | 46.9 | (52.3) | 1.8 | (2.4) | 0.2 | (0.3) |
| 106 | Bronchitis, not specified as acute or chronic, simple and mucopurulent chronic bronchitis and unspecified chronic bronchitis | J40–J42 | 12,790 | 61.5 | (20.6) | 2.6 | (1.8) | 15.3 | (22.4) | 0.8 | (2.1) | 2.5 | (8.5) | 0.9 | (2.2) | 0.3 | (5.5) | 14.9 | (34.0) | 1.1 | (2.8) | 0.1 | (0.1) |
| 107 | Emphysema | J43 | 5,557 | 53.8 | (20.6) | 3.3 | (2.1) | 17.7 | (20.7) | 1.0 | (1.5) | 2.2 | (8.4) | 0.9 | (1.9) | 0.4 | (6.1) | 19.6 | (36.4) | 1.2 | (2.2) | 0.0 | (0.0) |
| 108 | Chronic obstructive lung disease (COPD) ^c^ | J44, J96, J13–J18 | 216,184 | 47.2 | (20.4) | 3.4 | (2.4) | 11.4 | (12.2) | 1.1 | (1.9) | 2.4 | (4.4) | 1.2 | (1.8) | 2.1 | (6.7) | 29.7 | (47.5) | 1.5 | (2.5) | 0.1 | (0.2) |
| 109 | Asthma, status asthmaticus ^c^ | J45–J46 | 361,129 | 27.3 | (20.0) | 2.4 | (2.1) | 9.9 | (9.5) | 1.4 | (1.6) | 3.3 | (3.7) | 1.6 | (1.7) | 5.5 | (7.7) | 46.3 | (50.8) | 2.2 | (2.5) | 0.2 | (0.3) |
| 110 | Bronchiectasis | J47 | 4,362 | 46.3 | (19.9) | 3.9 | (2.3) | 11.3 | (13.2) | 1.1 | (1.9) | 1.9 | (4.3) | 0.9 | (1.2) | 1.0 | (7.9) | 31.9 | (46.5) | 1.6 | (2.6) | 0.0 | (0.3) |
| 111 | Other diseases of the respiratory system | J60–J84; J95, J97–J99 | 21,993 | 50.2 | (20.3) | 3.2 | (2.3) | 14.9 | (19.6) | 1.1 | (2.0) | 2.5 | (5.7) | 0.9 | (1.5) | 1.1 | (6.0) | 24.5 | (39.6) | 1.3 | (2.7) | 0.1 | (0.4) |
|  | **K – Diseases of the digestive system** | **K25–K27; K40, K43, K50–52; K58–K59; K71–K77; K86–K87** | **329,337** | **36.2** | (**20.1)** | **2.8** | (**2.2)** | **11.6** | (**11.8)** | **1.7** | (**2.2)** | **3.6** | (**5.0)** | **1.4** | (**1.7)** | **2.4** | (**6.3)** | **38.0** | (**47.4)** | **2.1** | (**3.0)** | **0.2** | (**0.3)** |
| 112 | Ulcers ^c^ | K25–K27 | 157,379 | 42.4 | (20.2) | 2.8 | (2.1) | 13.0 | (13.9) | 1.7 | (2.5) | 4.0 | (6.7) | 1.3 | (1.9) | 1.7 | (6.0) | 30.8 | (42.8) | 2.0 | (3.5) | 0.2 | (0.4) |
| 113 | Inguinal hernia | K40 | 25,032 | 38.8 | (19.7) | 3.3 | (2.5) | 4.6 | (5.6) | 0.9 | (1.7) | 1.4 | (2.9) | 1.2 | (1.5) | 1.2 | (6.6) | 47.1 | (57.2) | 1.3 | (2.1) | 0.1 | (0.2) |
| 114 | Ventral hernia | K43 | 7,941 | 37.6 | (20.0) | 3.3 | (2.2) | 14.0 | (12.8) | 2.0 | (2.5) | 3.8 | (7.0) | 1.4 | (2.0) | 0.8 | (5.6) | 35.0 | (45.2) | 1.8 | (2.4) | 0.1 | (0.3) |
| 115 | Crohn’s diease | K50 | 18,913 | 16.5 | (19.9) | 1.9 | (2.0) | 11.8 | (10.8) | 2.0 | (1.8) | 4.1 | (3.7) | 1.7 | (1.5) | 4.9 | (7.0) | 54.4 | (50.4) | 2.5 | (2.7) | 0.2 | (0.2) |
| 116 | Ulcerative colitis | K51 | 29,538 | 23.2 | (19.9) | 2.5 | (2.3) | 7.9 | (7.2) | 1.8 | (1.8) | 2.5 | (2.7) | 1.5 | (1.5) | 3.4 | (7.0) | 55.2 | (55.1) | 1.8 | (2.2) | 0.2 | (0.3) |
| 117 | Other non-infective gastroenteritis and colitis | K52 | 20,844 | 43.3 | (20.4) | 2.7 | (2.3) | 11.1 | (12.7) | 1.8 | (2.5) | 3.3 | (5.1) | 1.1 | (1.6) | 3.1 | (6.8) | 31.5 | (45.4) | 2.0 | (3.1) | 0.1 | (0.2) |
| 118 | Irritable bowel syndrome (IBS) | K58 | 37,593 | 21.4 | (19.8) | 2.6 | (2.3) | 10.3 | (9.1) | 2.3 | (2.0) | 4.0 | (4.0) | 1.8 | (1.7) | 3.7 | (5.9) | 51.5 | (52.5) | 2.3 | (2.6) | 0.1 | (0.2) |
| 119 | Other functional intestinal disorders | K59 | 51,933 | 42.8 | (20.3) | 2.3 | (2.1) | 13.5 | (16.5) | 1.7 | (2.4) | 3.5 | (5.1) | 1.0 | (1.5) | 2.9 | (6.3) | 30.0 | (42.3) | 2.1 | (3.2) | 0.1 | (0.2) |
| 120 | Diseases of liver, biliary tract and pancreas | K71–K77; K86–K87 | 26,956 | 32.8 | (20.3) | 3.6 | (2.1) | 20.6 | (17.7) | 1.9 | (2.5) | 4.6 | (7.2) | 1.7 | (2.0) | 1.3 | (6.5) | 30.8 | (37.6) | 2.6 | (3.8) | 0.2 | (0.3) |
|  | **L – Diseases of the skin and subcutaneous tissue** | **L40** | **65,469** | **30.9** | (**19.8)** | **3.3** | (**2.3)** | **9.0** | (**8.0)** | **1.2** | (**1.5)** | **2.0** | (**3.0)** | **1.5** | (**1.7)** | **1.9** | (**6.5)** | **48.1** | (**54.4)** | **1.8** | (**2.6)** | **0.2** | (**0.2)** |
| 121 | Psoriasis ^c^ | L40 | 65,469 | 30.9 | (19.8) | 3.3 | (2.3) | 9.0 | (8.0) | 1.2 | (1.5) | 2.0 | (3.0) | 1.5 | (1.7) | 1.9 | (6.5) | 48.1 | (54.4) | 1.8 | (2.6) | 0.2 | (0.2) |
|  | **M – Diseases of the musculoskeletal system and connective tissue** | **M01–M25; M30–M36; M40–M54; M60.1–M99** | **1,032,808** | **36.7** | (**19.8)** | **3.5** | (**2.4)** | **8.4** | (**8.1)** | **1.6** | (**2.2)** | **2.6** | (**3.9)** | **1.2** | (**1.5)** | **2.5** | (**7.1)** | **41.6** | (**52.2)** | **1.8** | (**2.5)** | **0.1** | (**0.2)** |
| 122 | Infectious arthropathies | M01–M03 | 9,402 | 19.8 | (19.3) | 2.3 | (2.2) | 8.1 | (7.3) | 1.7 | (1.6) | 3.5 | (3.5) | 1.5 | (1.3) | 2.9 | (6.2) | 57.8 | (55.9) | 2.2 | (2.6) | 0.1 | (0.2) |
| 122A | Inflammatory polyarthropathies and ankylosing spondylitis ^c^ | M05–M14, M45 | 165,944 | 41.9 | (20.0) | 3.5 | (2.3) | 10.7 | (10.4) | 1.1 | (1.9) | 1.9 | (3.7) | 1.1 | (1.4) | 1.0 | (6.9) | 37.0 | (50.2) | 1.7 | (2.9) | 0.1 | (0.3) |
| 123 | Rheumatoid arthritis ^c^ | M05, M06, M07.1, M07.2, M07.3, M08, M09 | 77,345 | 34.9 | (19.7) | 3.1 | (2.0) | 12.4 | (10.2) | 1.2 | (1.7) | 2.1 | (3.4) | 1.0 | (1.2) | 1.7 | (7.7) | 41.5 | (50.9) | 2.0 | (2.8) | 0.2 | (0.3) |
| 124 | Inflammatory polyarthropathies – except rheumatoid arthritis ^c^ | M074–M079, M10–M14, M45 | 115,945 | 43.9 | (20.0) | 3.6 | (2.3) | 10.6 | (10.9) | 1.1 | (2.0) | 1.8 | (4.0) | 1.1 | (1.5) | 0.7 | (6.2) | 35.4 | (49.9) | 1.6 | (3.0) | 0.1 | (0.2) |
| 125 | Polyarthrosis [arthrosis] | M15 | 16,935 | 59.3 | (19.9) | 5.0 | (2.6) | 11.6 | (12.8) | 0.9 | (3.3) | 1.4 | (6.9) | 0.5 | (1.7) | 0.1 | (5.0) | 19.7 | (45.7) | 1.4 | (1.9) | 0.1 | (0.0) |
| 126 | Coxarthrosis [arthrosis of hip] | M16 | 104,115 | 68.6 | (20.1) | 3.3 | (2.8) | 6.0 | (11.5) | 0.5 | (1.9) | 0.8 | (5.0) | 0.4 | (1.4) | 0.2 | (5.7) | 19.4 | (48.9) | 0.7 | (2.6) | 0.0 | (0.2) |
| 127 | Gonarthrosis [arthrosis of knee] | M17 | 178,811 | 50.8 | (19.8) | 4.5 | (2.6) | 7.5 | (7.4) | 1.0 | (2.4) | 1.5 | (4.5) | 0.9 | (1.5) | 0.1 | (4.9) | 32.4 | (54.4) | 1.2 | (2.2) | 0.1 | (0.2) |
| 128 | Arthrosis of first carpometacarpal joint and other arthrosis | M18–M19 | 91,101 | 43.2 | (19.8) | 4.8 | (2.6) | 10.4 | (9.2) | 1.5 | (2.7) | 2.2 | (5.2) | 1.1 | (1.5) | 0.3 | (5.5) | 34.9 | (50.9) | 1.6 | (2.5) | 0.1 | (0.1) |
| 129 | Acquired deformities of fingers and toes | M20 | 55,730 | 33.5 | (19.6) | 4.5 | (2.5) | 8.7 | (8.1) | 1.3 | (1.6) | 1.9 | (3.1) | 1.2 | (1.5) | 1.8 | (6.3) | 45.3 | (55.0) | 1.7 | (2.2) | 0.1 | (0.2) |
| 130 | Other acquired deformities of limbs | M21 | 20,584 | 30.8 | (19.8) | 3.6 | (2.4) | 11.6 | (11.4) | 1.4 | (1.7) | 2.7 | (3.8) | 1.3 | (1.4) | 3.8 | (7.3) | 42.8 | (49.6) | 1.9 | (2.3) | 0.1 | (0.2) |
| 131 | Disorders of patella (knee cap) | M22 | 38,999 | 2.6 | (18.9) | 0.7 | (1.9) | 5.8 | (6.2) | 2.8 | (2.0) | 5.9 | (3.9) | 1.9 | (1.6) | 11.4 | (6.7) | 65.9 | (56.5) | 2.9 | (2.2) | 0.3 | (0.2) |
| 132 | Internal derangement of knee | M230, M231, M233, M235, M236, M238 | 9,192 | 5.7 | (18.9) | 1.0 | (1.7) | 3.5 | (4.0) | 2.9 | (2.4) | 4.1 | (3.3) | 1.5 | (1.3) | 11.0 | (7.7) | 67.5 | (58.6) | 2.5 | (2.0) | 0.3 | (0.2) |
| 133 | Derangement of meniscus due to old tear or injury | M232 | 36,374 | 11.3 | (18.7) | 2.6 | (2.1) | 5.0 | (3.9) | 2.2 | (2.0) | 3.0 | (3.1) | 1.5 | (1.4) | 5.1 | (7.3) | 67.1 | (59.2) | 2.1 | (2.1) | 0.2 | (0.2) |
| 134 | Internal derangement of knee, unspecified | M239 | 28,206 | 9.8 | (18.8) | 1.7 | (1.9) | 5.5 | (5.1) | 2.4 | (2.2) | 3.8 | (3.4) | 1.5 | (1.4) | 8.8 | (7.7) | 63.9 | (57.2) | 2.4 | (2.1) | 0.2 | (0.2) |
| 135 | Other specific joint derangements | M24, except M240–M241 | 5,923 | 8.8 | (19.9) | 0.8 | (1.5) | 5.0 | (6.2) | 3.2 | (2.7) | 5.3 | (4.3) | 1.5 | (1.3) | 12.5 | (7.7) | 59.5 | (54.0) | 3.0 | (2.3) | 0.4 | (0.2) |
| 136 | Other joint disorders, not elsewhere classified | M25 | 12,043 | 15.6 | (19.5) | 2.4 | (2.1) | 9.6 | (7.5) | 4.1 | (3.7) | 5.6 | (5.1) | 1.9 | (1.7) | 5.6 | (7.5) | 52.1 | (49.8) | 3.1 | (3.0) | 0.2 | (0.2) |
| 137 | Systemic connective tissue disorders | M30–M36, except M32,M34 | 42,631 | 48.9 | (20.2) | 2.2 | (2.1) | 12.1 | (13.6) | 1.3 | (1.9) | 2.6 | (4.6) | 0.8 | (1.3) | 1.6 | (6.7) | 28.4 | (46.2) | 1.8 | (3.2) | 0.1 | (0.2) |
| 138 | Systemic lupus erythematosus | M32 | 3,376 | 23.6 | (19.9) | 2.3 | (1.8) | 23.4 | (18.3) | 1.5 | (2.5) | 3.3 | (4.5) | 0.9 | (1.1) | 2.5 | (9.0) | 39.5 | (39.0) | 2.8 | (3.9) | 0.1 | (0.1) |
| 139 | Dermatopolymyositis | M33 | 1,137 | 36.7 | (19.8) | 2.5 | (1.6) | 16.7 | (15.8) | 1.2 | (2.1) | 2.6 | (5.0) | 0.7 | (0.9) | 1.8 | (6.3) | 36.6 | (46.7) | 1.4 | (1.4) | 0.0 | (0.5) |
| 140 | Systemic sclerosis | M34 | 1,675 | 37.4 | (20.4) | 3.6 | (1.8) | 22.9 | (20.3) | 1.2 | (1.9) | 1.2 | (2.2) | 0.5 | (0.8) | 1.5 | (9.1) | 29.4 | (39.3) | 2.3 | (4.4) | 0.0 | (0.0) |
| 141 | Kyphosis, lordosis | M40 | 4,160 | 18.8 | (20.3) | 2.1 | (1.7) | 16.1 | (14.0) | 2.3 | (2.3) | 5.0 | (5.3) | 1.7 | (1.5) | 3.5 | (7.1) | 47.0 | (44.0) | 3.1 | (3.4) | 0.3 | (0.4) |
| 142 | Scoliosis | M41 | 17,686 | 18.6 | (20.0) | 1.4 | (1.8) | 14.5 | (17.4) | 1.7 | (1.6) | 3.8 | (3.7) | 1.3 | (1.5) | 13.8 | (8.2) | 41.9 | (43.1) | 2.8 | (2.6) | 0.2 | (0.2) |
| 143 | Spinal osteochondrosis | M42 | 8,034 | 15.7 | (19.9) | 2.0 | (1.6) | 19.1 | (15.3) | 2.1 | (2.2) | 5.0 | (4.9) | 1.9 | (1.7) | 2.8 | (7.1) | 48.6 | (44.4) | 2.6 | (2.9) | 0.2 | (0.2) |
| 144 | Other deforming dorsopathies | M43 | 23,756 | 36.0 | (20.0) | 2.8 | (1.8) | 15.4 | (13.2) | 1.8 | (2.6) | 3.3 | (5.1) | 1.2 | (1.4) | 1.1 | (6.8) | 36.5 | (45.8) | 2.0 | (3.2) | 0.1 | (0.1) |
| 145 | Other inflammatory spondylopathies | M46 | 7,086 | 24.6 | (19.8) | 1.9 | (1.6) | 16.7 | (14.0) | 2.3 | (2.5) | 4.4 | (5.3) | 1.2 | (1.3) | 1.8 | (5.9) | 44.2 | (45.8) | 2.7 | (3.7) | 0.1 | (0.1) |
| 146 | Spondylosis | M47 | 61,999 | 42.7 | (20.1) | 3.4 | (1.9) | 16.2 | (13.9) | 1.8 | (4.2) | 2.6 | (6.8) | 0.9 | (1.4) | 0.1 | (4.2) | 30.3 | (44.3) | 1.9 | (2.9) | 0.1 | (0.2) |
| 147 | Other spondylopathies and spondylopathies in diseases classified elsewhere | M48, M49 | 50,805 | 61.0 | (20.2) | 3.1 | (2.1) | 11.9 | (15.8) | 1.2 | (3.7) | 1.8 | (6.6) | 0.5 | (1.2) | 0.1 | (4.9) | 19.1 | (41.1) | 1.3 | (4.3) | 0.1 | (0.2) |
| 148 | Cervical disc disorders | M50 | 11,476 | 12.1 | (19.4) | 2.0 | (1.5) | 11.6 | (7.6) | 5.1 | (5.7) | 5.0 | (5.9) | 1.6 | (1.3) | 0.5 | (5.3) | 58.9 | (50.3) | 3.0 | (2.7) | 0.1 | (0.3) |
| 149 | Other intervertebral disc disorders | M51 | 40,161 | 19.5 | (19.7) | 2.2 | (1.8) | 10.7 | (8.4) | 5.3 | (5.4) | 6.2 | (6.7) | 1.5 | (1.4) | 1.2 | (5.2) | 50.6 | (48.1) | 2.6 | (3.0) | 0.1 | (0.2) |
| 150 | Other dorsopathies, not elsewhere classified | M53 | 7,246 | 16.5 | (19.6) | 1.8 | (1.6) | 10.5 | (8.6) | 5.8 | (5.2) | 6.0 | (5.9) | 1.5 | (1.3) | 3.1 | (7.2) | 51.8 | (47.6) | 2.8 | (2.7) | 0.2 | (0.3) |
| 151 | Dorsalgia | M54 | 40,780 | 22.0 | (19.8) | 1.8 | (1.8) | 10.1 | (8.9) | 5.2 | (5.0) | 6.7 | (6.8) | 1.7 | (1.6) | 2.9 | (6.4) | 46.7 | (46.3) | 2.8 | (3.2) | 0.1 | (0.2) |
| 152 | Soft tissue disorders | M60–M63, except M60.0 | 13,422 | 15.7 | (20.0) | 1.7 | (2.0) | 10.2 | (10.0) | 4.2 | (3.5) | 7.3 | (6.2) | 2.1 | (2.0) | 5.8 | (6.5) | 50.0 | (46.9) | 2.9 | (2.8) | 0.2 | (0.3) |
| 153 | Synovitis and tenosynovitis | M65 | 19,104 | 20.5 | (19.4) | 3.7 | (2.5) | 7.0 | (5.3) | 2.6 | (2.7) | 3.2 | (3.6) | 1.4 | (1.4) | 4.6 | (6.9) | 54.6 | (55.6) | 2.2 | (2.3) | 0.1 | (0.2) |
| 154 | Disorders of synovium and tendon | M66–68 | 19,669 | 12.5 | (19.2) | 2.3 | (2.4) | 5.4 | (4.9) | 2.3 | (2.1) | 3.6 | (3.2) | 1.7 | (1.5) | 8.5 | (7.6) | 61.2 | (56.7) | 2.4 | (2.2) | 0.2 | (0.2) |
| 155 | Soft tissue disorders related to use, overuse and pressure | M70 | 11,090 | 27.4 | (19.8) | 3.0 | (2.4) | 8.0 | (7.0) | 2.0 | (2.2) | 2.4 | (2.9) | 1.2 | (1.3) | 4.6 | (8.4) | 49.5 | (53.9) | 1.8 | (2.0) | 0.1 | (0.1) |
| 156 | Fibroblastic disorders | M72 | 43,600 | 45.4 | (19.9) | 4.3 | (2.8) | 6.6 | (6.7) | 0.9 | (1.7) | 1.3 | (2.7) | 1.0 | (1.4) | 0.4 | (6.0) | 38.6 | (56.4) | 1.2 | (2.2) | 0.1 | (0.2) |
| 157 | Shoulder lesions | M75 | 58,112 | 18.3 | (19.4) | 3.4 | (2.3) | 7.8 | (5.5) | 4.1 | (4.0) | 3.9 | (4.4) | 1.7 | (1.6) | 1.8 | (6.4) | 56.6 | (54.0) | 2.2 | (2.3) | 0.1 | (0.1) |
| 158 | Enthesopathies of lower limb, excluding foot | M76 | 11,223 | 10.2 | (19.1) | 1.6 | (1.7) | 4.2 | (3.7) | 1.6 | (1.4) | 2.3 | (2.1) | 1.4 | (1.3) | 8.6 | (8.7) | 68.3 | (60.3) | 1.8 | (1.6) | 0.1 | (0.1) |
| 159 | Other enthesopathies | M77 | 10,500 | 11.2 | (19.4) | 1.9 | (1.7) | 6.6 | (4.9) | 4.0 | (3.3) | 3.6 | (3.2) | 1.8 | (1.5) | 3.0 | (7.3) | 65.2 | (56.2) | 2.4 | (2.2) | 0.2 | (0.2) |
| 160 | Rheumatism, unspecified | M790 | 6,852 | 20.8 | (19.9) | 2.1 | (1.6) | 37.2 | (21.3) | 1.7 | (2.2) | 5.9 | (8.0) | 0.7 | (1.0) | 0.6 | (4.0) | 26.9 | (37.5) | 4.0 | (4.4) | 0.1 | (0.1) |
| 161 | Myalgia | M791 | 10,168 | 24.6 | (20.1) | 2.4 | (1.8) | 17.3 | (13.4) | 2.4 | (2.5) | 5.6 | (6.3) | 1.7 | (1.9) | 1.3 | (4.9) | 41.3 | (44.9) | 3.3 | (4.0) | 0.1 | (0.2) |
| 162 | Other soft tissue disorders, not elsewhere classified | M792– M794; M798–M799 | 7,939 | 20.3 | (20.0) | 2.2 | (1.9) | 15.5 | (12.6) | 3.1 | (2.8) | 4.8 | (4.9) | 1.4 | (1.3) | 2.9 | (6.6) | 47.1 | (47.1) | 2.5 | (2.5) | 0.2 | (0.2) |
| 163 | Other soft tissue disorders, not elsewhere classified: pain in limb | M796 | 22,201 | 23.9 | (19.8) | 2.2 | (2.0) | 9.3 | (8.5) | 2.8 | (2.9) | 4.3 | (4.7) | 1.7 | (1.7) | 5.4 | (7.6) | 48.0 | (50.3) | 2.2 | (2.3) | 0.2 | (0.2) |
| 164 | Fibromyalgia | M797 | 3,399 | 11.1 | (19.6) | 1.1 | (1.3) | 34.2 | (23.4) | 4.4 | ((3.4) | 14.6 | (12.6) | 0.9 | (0.7) | 1.0 | (2.8) | 25.5 | (27.0) | 7.3 | (9.1) | 0.0 | (0.1) |
| 165 | Osteoporosis ^c^ | M80–M81 | 158,813 | 71.2 | (20.2) | 4.5 | (2.8) | 7.3 | (22.8) | 0.3 | (1.9) | 0.5 | (3.6) | 0.3 | (0.7) | 0.0 | (6.4) | 14.9 | (39.3) | 0.8 | (2.2) | 0.0 | (0.1) |
| 166 | Osteoporosis in diseases classified elsewhere | M82 | 1,007 | 54.3 | (20.8) | 2.4 | (1.8) | 19.7 | (28.9) | 0.9 | (1.4) | 1.3 | (3.1) | 0.0 | (0.6) | 1.1 | (6.6) | 19.2 | (34.5) | 1.0 | (2.2) | 0.0 | (0.0) |
| 167 | Adult osteomalacia and other disorders of bone density and structure | M83, M85, except M833 | 43,271 | 47.3 | (20.0) | 5.4 | (2.6) | 10.6 | (11.6) | 0.8 | (1.8) | 1.5 | (4.0) | 0.8 | (1.6) | 0.7 | (7.3) | 31.2 | (48.2) | 1.5 | (2.7) | 0.1 | (0.2) |
| 168 | Disorders of continuity of bone | M84 | 1,865 | 24.4 | (20.3) | 2.2 | (1.7) | 12.7 | (12.3) | 4.1 | (4.7) | 6.0 | (6.7) | 1.4 | (1.4) | 7.1 | (7.4) | 39.5 | (42.5) | 2.6 | (2.8) | 0.0 | (0.1) |
| 169 | Other osteopathies | M86–M90 | 24,251 | 41.7 | (20.0) | 3.3 | (2.2) | 14.0 | (14.7) | 1.3 | (2.2) | 2.6 | (4.6) | 0.9 | (1.2) | 1.5 | (6.8) | 32.7 | (45.1) | 1.8 | (2.8) | 0.1 | (0.3) |
| 170 | Other disorders of the musculoskeletal system and connective tissue | M95–M99 | 30,038 | 23.6 | (20.0) | 2.1 | (1.9) | 13.9 | (12.7) | 2.2 | (2.4) | 4.4 | (4.7) | 1.8 | (1.8) | 3.5 | (6.7) | 45.9 | (46.7) | 2.4 | (2.9) | 0.3 | (0.3) |
|  | **N – Diseases of the genitourinary system** | **N18** | **20,162** | **61.7** | **(20.5)** | **2.1** | **(1.9)** | **14.4** | **(24.6)** | **0.7** | (**1.8)** | **1.4** | (**4.2)** | **0.5** | (**1.1)** | **0.8** | (**6.4)** | **17.1** | (**36.0)** | **1.2** | (**3.4)** | **0.1** | (**0.1)** |
| 171 | Chronic renal failure (CRF) ^c^ | N18 | 20,162 | 61.7 | (20.5) | 2.1 | (1.9) | 14.4 | (24.6) | 0.7 | (1.8) | 1.4 | (4.2) | 0.5 | (1.1) | 0.8 | (6.4) | 17.1 | (36.0) | 1.2 | (3.4) | 0.1 | (0.1) |
|  | **Q – Congenital malformations, deformations and chromosomal abnormalities** | **Q00–Q56; Q60–Q99** | **124,898** | **13.0** | **(19.7)** | **1.6** | **(2.0)** | **11.7** | **(11.7)** | **2.0** | (**1.6)** | **3.9** | (**3.2)** | **1.9** | (**1.6)** | **6.7** | ((**7.0)** | **56.4** | (**50.4)** | **2.6** | (**2.5)** | **0.3** | (**0.2)** |
| 172 | Congenital malformations: of the nervous, circulatory and respiratory systems, cleft palate and cleft lip, urinary tract, bones and muscles, other and (chromosomal abnormalities not elsewhere c(lassified | Q00–Q07; Q20–Q37; Q60–Q99 | 85,534 | 12.0 | (19.7) | 1.5 | (2.0) | 13.7 | (13.7) | 2.1 | (1.6) | 4.0 | (3.2) | 1.7 | (1.5) | 7.2 | (7.0) | 54.8 | (48.6) | 2.7 | (2.4) | 0.3 | (0.2) |
| 173 | Congenital malformations of eye, ear, face and neck | Q10–Q18 | 19,689 | 9.5 | (19.7) | 1.3 | (2.1) | 7.4 | (8.0) | 2.3 | (1.6) | 4.1 | (3.1) | 2.3 | (1.9) | 8.2 | (7.3) | 61.8 | (53.4) | 2.8 | (2.5) | 0.3 | (0.2) |
| 174 | Other congenital malformations of the digestive system | Q38–Q45 | 6,481 | 34.8 | (20.0) | 2.6 | (2.2) | 11.4 | (12.2) | 1.5 | (1.8) | 3.5 | (4.6) | 1.4 | (1.6) | 2.8 | (6.3) | 39.6 | (47.8) | 2.3 | (3.2) | 0.1 | (0.2) |
| 175 | Congenital malformations of the sexual organs | Q50–Q56 | 16,192 | 12.8 | (19.3) | 1.7 | (2.2) | 7.5 | (7.3) | 1.6 | (1.4) | 3.2 | (3.1) | 2.1 | (1.8) | 3.8 | (6.3) | 64.9 | (55.9) | 2.2 | (2.4) | 0.3 | (0.3) |
|  | **F – Mental and behavioural disorders** | **F00–99** | **683,194** | **23.8** | (**20.4)** | **1.9** | (**1.9)** | **17.2** | (**17.3)** | **2.9** | (**2.9)** | **8.7** | (**9.3)** | **2.1** | (**2.1)** | **4.9** | (**6.4)** | **34.6** | (**35.3)** | **3.6** | (**4.2)** | **0.2** | (**0.2)** |
| 176 | Dementia ^c^ | F00, G30, F01, F02.0, F03.9, G31.8B, G31.8E, G31.9, G31.0B | 36,803 | 94.9 | (21.3) | 0.5 | (1.9) | 3.2 | (55.8) | 0.0 | (0.4) | 0.2 | (4.4) | 0.0 | (0.0) | 0.0 | (1.0) | 1.0 | (13.0) | 0.1 | (2.1) | 0.0 | (0.0) |
| 177 | Organic, including symptomatic, mental disorders | F04–F09 | 26,430 | 50.4 | (20.9) | 1.1 | (1.0) | 30.3 | (43.0) | 1.0 | (1.8) | 4.2 | (9.7) | 0.6 | (0.9) | 0.7 | (3.3) | 10.0 | (15.7) | 1.5 | (3.6) | 0.1 | (0.2) |
| 178 | Mental and behavioural disorders due to use of alcohol | F10 | 59,143 | 18.9 | (20.6) | 2.3 | (1.6) | 27.9 | (23.2) | 3.3 | (3.2) | 13.8 | (15.2) | 2.7 | (2.4) | 3.5 | (5.2) | 23.5 | (24.0) | 4.0 | (4.3) | 0.2 | (0.3) |
| 179 | Mental and behavioural disorders due to psychoactive substance use | F11–F19 | 53,669 | 14.1 | (20.3) | 1.5 | (1.7) | 22.5 | (21.2) | 3.0 | (2.8) | 18.2 | (16.1) | 2.3 | (2.0) | 3.9 | (3.7) | 29.0 | (27.2) | 5.2 | (4.8) | 0.2 | (0.2) |
| 180 | Schizophrenia ^c^ | F20 | 29,422 | 10.3 | (20.9) | 0.1 | (0.1) | 64.4 | (53.4) | 1.3 | (1.2) | 11.8 | (12.1) | 0.6 | (0.5) | 2.2 | (2.8) | 6.1 | (5.3) | 2.9 | (3.4) | 0.3 | (0.2) |
| 181 | Schizotypal and delusional disorders | F21–F29 | 39,694 | 14.8 | (20.8) | 0.6 | (0.6) | 46.3 | (40.5) | 1.9 | (1.7) | 12.7 | (12.8) | 1.5 | (1.2) | 3.3 | (4.3) | 14.7 | (13.3) | 3.9 | (4.5) | 0.3 | (0.3) |
| 182 | Bipolar affective disorder ^c^ | F30–F31 | 22,669 | 24.8 | (20.6) | 1.3 | (1.0) | 34.0 | (30.2) | 3.2 | (3.4) | 8.8 | (11.7) | 1.2 | (1.2) | 2.6 | (5.5) | 20.4 | (21.4) | 3.4 | (4.5) | 0.2 | (0.3) |
| 183 | Depression ^c^ | F32, F33, F34.1, F06.32 | 454,933 | 26.6 | (20.3) | 2.3 | (2.1) | 15.3 | (14.8) | 3.3 | (3.5) | 8.1 | (9.7) | 2.0 | (2.1) | 3.3 | (5.9) | 35.6 | (37.3) | 3.2 | (4.2) | 0.2 | (0.2) |
| 184 | Mood (affective) disorders | F340, F348–F349, F38–F39 | 6,887 | 21.3 | (20.7) | 1.1 | (1.1) | 33.2 | (29.5) | 2.7 | (2.7) | 11.4 | (12.8) | 1.0 | (1.0) | 3.3 | (5.8) | 22.3 | (22.2) | 3.4 | (4.1) | 0.2 | (0.2) |
| 185 | Phobic anxiety disorders | F40 | 14,324 | 3.0 | (19.9) | 0.4 | (1.1) | 20.7 | (21.7) | 5.0 | (3.4) | 21.9 | (15.6) | 2.1 | (1.7) | 7.2 | (5.4) | 32.8 | (25.0) | 6.7 | (6.0) | 0.2 | (0.2) |
| 186 | Other anxiety disorders | F41 | 38,079 | 12.4 | (20.5) | 1.1 | (1.4) | 22.9 | (21.6) | 4.3 | (3.4) | 15.3 | (13.2) | 2.0 | (1.7) | 5.1 | (5.4) | 31.9 | (27.5) | 4.9 | (5.1) | 0.2 | (0.2) |
| 187 | Obsessive compulsive disorder (OCD) ^c^ | F42 | 10,062 | 4.3 | (20.2) | 0.5 | (1.3) | 20.1 | (24.7) | 3.9 | (2.7) | 14.0 | (8.9) | 1.9 | (1.8) | 14.3 | (7.6) | 35.1 | (28.6) | 5.6 | (3.9) | 0.2 | (0.1) |
| 188 | Post-traumatic stress disorder | F431 | 16,055 | 4.0 | (19.2) | 0.5 | (0.8) | 37.4 | (27.9) | 4.8 | (3.7) | 23.7 | (20.2) | 1.6 | (1.1) | 2.0 | (3.5) | 21.6 | (18.4) | 3.9 | (4.5) | 0.5 | (0.6) |
| 189 | Reactions to severe stress and adjustment disorders | F432–F439 | 61,701 | 6.9 | (20.1) | 1.0 | (1.6) | 16.1 | (15.8) | 5.0 | (4.1) | 16.7 | (12.9) | 2.6 | (2.3) | 8.1 | (5.7) | 37.0 | (32.1) | 6.1 | (5.0) | 0.3 | (0.3) |
| 190 | Dissociative (conversion) disorders, somatoform disorders and other neurotic disorders | F44, F45, F48 | 21,420 | 14.6 | (20.3) | 1.1 | (1.2) | 30.5 | (25.5) | 3.0 | (2.5) | 10.3 | (10.0) | 1.7 | (1.5) | 3.4 | (5.8) | 31.3 | (28.9) | 3.8 | (4.3) | 0.2 | (0.2) |
| 191 | Eating disorders | F50 | 7,751 | 1.1 | (21.5) | 0.0 | (0.2) | 10.2 | (25.2) | 4.2 | (3.1) | 15.2 | (10.2) | 1.2 | (0.6) | 24.3 | (8.4) | 37.7 | (26.1) | 5.9 | (4.5) | 0.3 | (0.2) |
| 192 | Behavioural syndromes associated with physiological disturbances and physical factors | F51–F59 | 6,163 | 5.8 | (19.1) | 1.0 | (1.6) | 11.2 | (11.3) | 3.2 | (2.3) | 7.1 | (5.6) | 2.7 | (2.2) | 5.7 | (5.8) | 59.3 | (48.1) | 3.7 | (3.7) | 0.4 | (0.3) |
| 193 | Emotionally unstable personality disorder | F603 | 21,848 | 2.4 | (20.5) | 0.2 | (0.6) | 35.5 | (35.1) | 3.9 | (2.4) | 23.1 | (15.5) | 1.7 | (1.3) | 5.1 | (3.4) | 23.4 | (17.2) | 4.5 | (3.8) | 0.2 | (0.2) |
| 194 | Specific personality disorders | F602, F604–F609 | 50,415 | 7.2 | (20.4) | 0.6 | (1.0) | 32.2 | (28.3) | 3.6 | (2.6) | 17.3 | (14.3) | 2.2 | (1.7) | 4.0 | (4.2) | 28.0 | (22.2) | 4.7 | (5.1) | 0.2 | (0.2) |
| 195 | Disorders of adult personality and behaviour | F61–F69 | 17,533 | 6.1 | (20.4) | 0.5 | (0.7) | 36.1 | (30.8) | 3.8 | (2.7) | 18.3 | (14.7) | 1.9 | (1.4) | 4.2 | (4.7) | 24.6 | (19.7) | 4.4 | (4.6) | 0.3 | (0.3) |
| 196 | Mental retardation | F70–F79 | 13,822 | 10.5 | (21.0) | 0.0 | (0.0) | 73.5 | (66.9) | 0.4 | (0.3) | 5.7 | (4.3) | 0.2 | (0.2) | 4.2 | (3.0) | 3.2 | (2.6) | 2.4 | (1.8) | 0.0 | (0.0) |
| 197 | Disorders of psychological development | F80–F89 | 9,911 | 3.3 | (20.8) | 0.0 | (0.2) | 38.9 | (51.3) | 1.6 | (1.5) | 15.4 | (7.4) | 0.7 | (0.6) | 20.0 | (6.3) | 11.5 | (8.4) | 8.6 | (3.4) | 0.1 | (0.0) |
| 198 | Hyperkinetic disorders (ADHD) ^c^ | F90 | 42,908 | 2.5 | (19.6) | 0.3 | (1.1) | 15.8 | (21.0) | 3.2 | (2.9) | 20.3 | (13.7) | 1.7 | (1.6) | 18.4 | (7.1) | 29.3 | (28.0) | 8.3 | (4.8) | 0.2 | (0.2) |
| 199 | Behavioural and emotional disorders with onset usually occurring in childhood and adolescence | F91–F99 | 39,602 | 9.9 | (20.6) | 0.7 | (1.0) | 29.6 | (29.7) | 2.9 | (2.4) | 16.1 | (12.4) | 1.8 | (1.6) | 8.2 | (5.9) | 24.8 | (21.4) | 5.8 | (4.6) | 0.3 | (0.3) |
|  |  |  |  |  |  |  |  |  |  |  |  |  |  |  |  |  |  |  |  |  |  |  |  |
|  | 0 chronic conditions |  | 1,565,998 | 4.6 | (18.5) | 1.2 | (2.3) | 1.1 | (1.3) | 0.8 | (0.6) | 1.4 | (1.4) | 1.8 | (1.7) | 16.3 | (8.8) | 65.4 | (60.2) | 4.4 | (3.4) | 2.9 | (2.1) |
|  | 1 chronic condition |  | 906,365 | 10.8 | (18.9) | 2.2 | (2.5) | 2.7 | (2.5) | 1.2 | (1.0) | 2.3 | (1.8) | 2.1 | (1.8) | 7.9 | (7.8) | 67.7 | (60.6) | 2.7 | (2.5) | 0.5 | (0.4) |
|  | 2 chronic conditions |  | 601,767 | 19.0 | (19.2) | 3.2 | (2.6) | 4.9 | (4.4) | 1.5 | (1.5) | 3.1 | (3.4) | 2.0 | (1.9) | 4.5 | (7.1) | 59.3 | (56.9) | 2.4 | (2.7) | 0.3 | (0.3) |
|  | 3 chronic conditions |  | 435,614 | 26.8 | (19.4) | 3.7 | (2.7) | 7.0 | (6.8) | 1.7 | (1.9) | 3.6 | (4.9) | 1.8 | (1.9) | 2.7 | (6.4) | 50.4 | (52.7) | 2.2 | (3.0) | 0.2 | (0.2) |
|  | 4 chronic conditions |  | 306,882 | 34.6 | (19.7) | 3.9 | (2.7) | 9.3 | (9.1) | 1.8 | (2.4) | 3.8 | (6.8) | 1.5 | (1.8) | 1.7 | (5.8) | 41.3 | (47.3) | 2.0 | (3.4) | 0.1 | (0.2) |
|  | 5 chronic conditions |  | 218,183 | 41.7 | (19.9) | 4.0 | (2.6) | 11.4 | (13.8) | 1.8 | (2.9) | 4.0 | (9.0) | 1.3 | (1.7) | 1.1 | (5.0) | 32.8 | (41.2) | 1.9 | (3.8) | 0.1 | (0.1) |
|  | 6 chronic conditions |  | 155,685 | 47.5 | (20.1) | 3.7 | (2.5) | 13.3 | (18.2) | 1.8 | (3.3) | 3.9 | (10.7) | 1.0 | (1.5) | 0.7 | (4.1) | 26.2 | (35.3) | 1.8 | (4.2) | 0.1 | (0.1) |
|  | 7 chronic conditions |  | 109,688 | 52.6 | (22.5) | 3.4 | (2.4) | 14.8 | (22.5) | 1.6 | (3.2) | 3.7 | (12.3) | 0.8 | (1.3) | 0.5 | (3.9) | 20.9 | (30.0) | 1.7 | (4.0) | 0.1 | (0.1) |
|  | 8 chronic conditions |  | 77,563 | 57.0 | (20.4) | 3.0 | (2.2) | 16.2 | (27.0) | 1.5 | (3.4) | 3.4 | (13.0) | 0.6 | (1.0) | 0.4 | (3.5) | 16.5 | (25.6) | 1.5 | (4.0) | 0.1 | (0.1) |
|  | 9 chronic conditions |  | 54,429 | 60.3 | (20.6) | 2.7 | (2.1) | 17.2 | (32.1) | 1.3 | (3.2) | 3.2 | (13.9) | 0.5 | (1.0) | 0.2 | (2.4) | 13.2 | (21.1) | 1.4 | (3.7) | 0.0 | (0.1) |
|  | 10 or mor chronic conditions |  | 123,265 | 66.7 | (20.8) | 1.8 | (1.5) | 19.3 | (41.3) | 0.9 | (2.7) | 2.3 | (13.4) | 0.2 | (0.6) | 0.1 | (1.8) | 7.6 | (14.3) | 1.0 | (3.4) | 0.0 | (0.1) |
|  |  |  |  |  |  |  |  |  |  |  |  |  |  |  |  |  |  |  |  |  |  |  |  |
|  | **Having one or more chronic conditions** |  | **2,989,441** | **54.2** | (**19.7)** | **6.0** | (**2.5)** | **14.6** | (**7.1)** | **2.9** | (**1.6)** | **6.2** | (**3.8)** | **3.3** | (**1.8)** | **8.0** | (**7.2)** | **0.0** | (**53.3)** | **4.4** | (**2.8)** | **0.5** | (**0.3)** |
|  |  |  |  |  |  |  |  |  |  |  |  |  |  |  |  |  |  |  |  |  |  |  |  |
|  | **Total population** |  | **4,555,439** | **19.5** | **(19.5)** | **2.4** | (**2.4)** | **5.2** | (**5.2)** | **1.2** | (**1.2)** | **2.5** | (**2.5)** | **1.7** | (**1.7)** | **8.3** | (**8.2)** | **55.0** | (**55.0)** | **3.0** | (**3.0)** | **1.2** | (**1.2)** |
|  |  |  |  |  |  |  |  |  |  |  |  |  |  |  |  |  |  |  |  |  |  |  |  |
| 218 | Depression medicine ^c^ ** | ATC: N06A | 529,918 | 27.9 | (20.3) | 2.2 | (2.0) | 14.5 | (14.1) | 3.5 | (3.8) | 7.8 | (9.5) | 2.0 | (2.1) | 3.4 | (6.1) | 35.2 | (37.5) | 3.2 | (4.3) | 0.2 | (0.2) |
| 219 | Antipsychotic medicine ^c^ ** | ATC: N05A | 138,625 | 24.8 | (20.7) | 1.2 | (1.1) | 32.5 | (32.1) | 3.4 | (3.6) | 12.6 | (14.2) | 1.1 | (1.1) | 3.4 | (4.8) | 16.7 | (17.3) | 4.0 | (4.8) | 0.2 | (0.2) |
| 220 | Indication prescribed anxiety medicine ^c^ ** | All prescrib. w.indication codes 163 (for anxiety) or 371 (for anxiety, addictive) | 102,568 | 22.8 | (20.3) | 2.0 | (1.9) | 17.9 | (17.0) | 3.8 | (3.8) | 10.0 | (10.9) | 1.9 | (1.8) | 3.9 | (5.8) | 33.9 | (33.7) | 3.7 | (4.6) | 0.2 | (0.2) |
| 221 | Heart failure medication ^c^ ** | ATC: C01AA05, C03, C07 or C09A with indication code 430 (for heart failure) | 7,468 | 64.6 | (20.2) | 3.1 | (2.4) | 9.3 | (18.0) | 1.1 | (3.0) | 1.5 | (5.5) | 0.6 | (0.9) | 0.2 | (6.8) | 18.4 | (39.9) | 1.2 | (3.0) | 0.1 | (0.4) |
| 222 | Ischaemic heart medication ^c^ ** | ATC: C01A, C01B, C01D, C01E | 129,484 | 71.2 | (20.4) | 2.8 | 2.4) | 8.2 | (15.9) | 0.6 | (2.9) | 1.1 | (6.2) | 0.4 | (1.3) | 0.1 | (6.6) | 14.9 | (41.1) | 0.8 | (2.9) | 0.1 | (0.3) |
| **223** | **All five types of the medicine above** |  | **688,006** | **34.0** | **(20.3)** | **2.3** | (**2.0)** | **14.9** | (**15.8)** | **2.9** | (**3.5)** | **7.0** | (**9.7)** | **1.7** | (**2.0)** | **3.0** | (**6.0)** | **31.1** | (**36.1)** | **2.9** | (**4.3)** | **0.2** | (**0.2)** |
|  |  |  |  |  |  |  |  |  |  |  |  |  |  |  |  |  |  |  |  |  |  |  |  |
|  | **Extra:** |  |  |  |  |  |  |  |  |  |  |  |  |  |  |  |  |  |  |  |  |  |  |
|  | Ischaemic Heart Diseases | I05-I06; I11-I13; I20-I28; I30-I52 | 315,901 | 58.2 | (20.1) | 3.3 | (2.5) | 7.8 | (10.9) | 0.8 | (2.0) | 1.5 | (4.7) | 0.8 | (1.6) | 0.6 | (6.0) | 25.8 | (48.9) | 1.2 | (3.0) | 0.1 | (0.3) |
|  | Artritis | M01-M03; M5-M9; M7-M14; M15-M20; M45 | 505,792 | 45.6 | (19.8) | 4.1 | (2.5) | 8.5 | (8.4) | 1.1 | (2.0) | 1.8 | (4.0) | 1.0 | (1.5) | 0.7 | (6.4) | 35.7 | (52.6) | 1.4 | (2.6) | 0.1 | (0.2) |
|  | Arthrosis | M15-M19 | 338,166 | 51.8 | (19.9) | 4.3 | (2.6) | 7.9 | (8.5) | 1.0 | (2.3) | 1.6 | (5.0) | 0.9 | (1.5) | 0.2 | (5.4) | 31.0 | (52.3) | 1.2 | (2.4) | 0.1 | (0.1) |
|  | Backconditions | M32-34;M41-M43;M46-49;M50-51;M53-M54 | 212,948 | 32.1 | (20.0) | 2.6 | (1.9) | 12.8 | (11.8) | 2.7 | (3.3) | 4.0 | (5.3) | 1.3 | (1.4) | 2.3 | (7.0) | 39.9 | (46.1) | 2.2 | (2.9) | 0.1 | (0.2) |
|  | Overweight | E66 | 220,928 | 4.9 | (19.9) | 2.1 | (2.3) | 9.9 | (12.4) | 2.5 | (2.7) | 3.5 | (6.4) | 2.2 | (2.1) | 1.9 | (5.1) | 70.0 | (45.8) | 2.9 | (3.2) | 0.2 | (0.2) |
|  | Endometriosis | N80 | 29,190 | 4.9 | (9.9) | 2.1 | (0.9) | 9.9 | (10.5) | 2.5 | (1.1) | 3.5 | (1.8) | 2.2 | (0.8) | 1.9 | (2.7) | 70.0 | (26.9) | 2.9 | (1.4) | 0.2 | (0.1) |

Age and gender standardised estimates in brackets.

ICD-10 International Statistical Classification of Diseases, 10^th^ Revision.

^c^ = complex defined conditions, see reference for further details [55].

** 2-year prevalence.

***S4 Table.*** Catalogue of disease prevalence: number of patients, prevalence rates per thousand on all five levels of educational achievements for 199+ chronic conditions and disease groups in Denmark, on 1 January 2013.

| **No.** | **Name of condition** | **Total population*** | | | **Disease prevalence by educational achievement** | | | | | | | | | | | | | | | | | |
| --- | --- | --- | --- | --- | --- | --- | --- | --- | --- | --- | --- | --- | --- | --- | --- | --- | --- | --- | --- | --- | --- | --- |
|  |  |  |  |  | **No education**  **or training** | | | **Student**  **or training** | | | **Shorter**  **education** | | | **Middle (BSc or**  **equal)** | | | **Higher (MSc degree or doctorate)** | | | **Missing** | | |
|  |  | N | Per thousand | | N | Per thousand | | N | Per thousand | | N | Per thousand | | N | Per thousand | | N | Per thousand | | N | Per thousand | |
|  | **B – Viral hepatitis and human immunodeficiency virus [HIV] disease** | **8,500** | **1.9** | **(1.9)** | **3,091** | **2.7** | **(3.1)** | **73** | **0.3** | **(2.0)** | **3,162** | **1.6** | **(1.3)** | **865** | **1.3** | **(1.1)** | **488** | **1.5** | **(0.8)** | **821** | **4.1** | **(4.3)** |
| 1 | Chronic viral hepatitis | 4,584 | 1.0 | (1.0) | 2,133 | 1.9 | (2.1) | 52 | 0.2 | (1.1) | 1,323 | 0.7 | (0.5) | 385 | 0.6 | (0.4) | 160 | 0.5 | (0.1) | 531 | 2.6 | (2.8) |
| 2 | Human immunodeficiency virus [HIV] disease | 4,229 | 0.9 | (0.9) | 1,076 | 1.0 | (1.1) | 22 | 0.1 | (0.9) | 1,943 | 1.0 | (0.8) | 505 | 0.8 | (0.7) | 346 | 1.1 | (0.8) | 337 | 1.7 | (1.8) |
|  | **C – Malignant neoplasms** | **229,331** | **50.3** | **(50.4)** | **76,501** | **67.8** | **(48.5)** | **537** | **2.2** | **(47.9)** | **97,942** | **48.9** | **(51.6)** | **33,935** | **51.6** | **(52.5)** | **13,755** | **42.8** | **(52.8)** | **6,661** | **33.0** | **(40.5)** |
| 3 | Malignant neoplasms of other and unspecified localizations | 20,557 | 4.5 | (4.6) | 6,702 | 5.9 | (4.9) | 86 | 0.4 | (4.4) | 9,110 | 4.5 | (4.5) | 2,812 | 4.3 | (4.3) | 1,209 | 3.8 | (4.1) | 638 | 3.2 | (4.1) |
| 4 | Malignant neoplasms of digestive organs | 4,839 | 1.1 | (1.1) | 1,825 | 1.6 | (1.2) | 9 | 0.0 | (1.1) | 2,090 | 1.0 | (1.1) | 533 | 0.8 | (0.9) | 213 | 0.7 | (0.8) | 169 | 0.8 | (1.0) |
| 5 | Malignant neoplasm of colon | 18,826 | 4.1 | (4.1) | 7,233 | 6.4 | (4.0) | 11 | 0.0 | (4.1) | 7,687 | 3.8 | (4.3) | 2,215 | 3.4 | (4.0) | 936 | 2.9 | (4.2) | 744 | 3.7 | (3.7) |
| 6 | Malignant neoplasms of rectosigmoid junction, rectum, anus and anal canal | 10,680 | 2.3 | (2.3) | 4,011 | 3.6 | (2.5) | 0 | 0.0 | (2.4) | 4,564 | 2.3 | (2.4) | 1,259 | 1.9 | (2.2) | 531 | 1.7 | (2.2) | 315 | 1.6 | (1.9) |
| 7 | Malignant neoplasm of bronchus and lung | 14,762 | 3.2 | (3.3) | 6,254 | 5.5 | (4.2) | 6 | 0.0 | (3.4) | 6,191 | 3.1 | (3.2) | 1,425 | 2.2 | (2.3) | 482 | 1.5 | (2.1) | 404 | 2.0 | (2.6) |
| 8 | Malignant melanoma of skin | 19,636 | 4.3 | (4.3) | 4,515 | 4.0 | (3.1) | 60 | 0.2 | (3.6) | 9,109 | 4.5 | (4.6) | 3,745 | 5.7 | (5.5) | 1,778 | 5.5 | (5.8) | 429 | 2.1 | (2.8) |
| 9 | Other malignant neoplasms of skin | 15,597 | 3.4 | (3.4) | 5,209 | 4.6 | (2.8) | 0 | 0.0 | (3.3) | 6,432 | 3.2 | (3.5) | 2,118 | 3.2 | (3.7) | 1,025 | 3.2 | (4.1) | 809 | 4.0 | (4.1) |
| 10 | Malignant neoplasm of breast | 50,687 | 11.1 | (11.1) | 17,376 | 15.4 | (9.4) | 5 | 0.0 | (10.7) | 20,166 | 10.1 | (11.7) | 9,462 | 14.4 | (12.6) | 2,426 | 7.6 | (12.4) | 1,252 | 6.2 | (8.4) |
| 11 | Malignant neoplasms of female genital organs | 7,245 | 1.6 | (1.6) | 2,671 | 2.4 | (1.7) | 13 | 0.1 | (1.6) | 2,783 | 1.4 | (1.6) | 1,230 | 1.9 | (1.5) | 352 | 1.1 | (1.6) | 196 | 1.0 | (1.2) |
| 12 | Malignant neoplasm of cervix uteri, corpus uteri and part unspecified | 11,608 | 2.5 | (2.5) | 4,350 | 3.9 | (2.7) | 0 | 0.0 | (2.5) | 4,455 | 2.2 | (2.6) | 1,988 | 3.0 | (2.5) | 494 | 1.5 | (2.4) | 318 | 1.6 | (1.9) |
| 13 | Malignant tumour of male genitalia | 5,194 | 1.1 | (1.1) | 1,116 | 1.0 | (1.1) | 28 | 0.1 | (0.9) | 2,817 | 1.4 | (1.2) | 608 | 0.9 | (1.2) | 535 | 1.7 | (1.3) | 90 | 0.4 | (0.4) |
| 14 | Malignant neoplasm of prostate | 26,697 | 5.9 | (5.9) | 8,146 | 7.2 | (5.1) | 0 | 0.0 | (5.6) | 12,294 | 6.1 | (5.9) | 3,302 | 5.0 | (6.7) | 2,206 | 6.9 | (7.1) | 749 | 3.7 | (4.9) |
| 15 | Malignant neoplasms of urinary tract | 10,319 | 2.3 | (2.3) | 3,962 | 3.5 | (2.6) | 8 | 0.0 | (2.3) | 4,494 | 2.2 | (2.3) | 1,026 | 1.6 | (2.0) | 476 | 1.5 | (1.8) | 353 | 1.7 | (2.0) |
| 16 | Brain cancer ^c^ | 15,310 | 3.4 | (3.4) | 4,860 | 4.3 | (3.7) | 125 | 0.5 | (3.1) | 6,824 | 3.4 | (3.4) | 2,180 | 3.3 | (3.3) | 957 | 3.0 | (3.1) | 364 | 1.8 | (2.4) |
| 17 | Malignant neoplasms of ill–defined, secondary and unspecified sites, and of independent (primary) multiple sites | 25,619 | 5.6 | (5.6) | 8,737 | 7.7 | (5.9) | 45 | 0.2 | (5.3) | 11,028 | 5.5 | (5.7) | 3,792 | 5.8 | (5.7) | 1,429 | 4.4 | (5.4) | 588 | 2.9 | (4.0) |
| 18 | Malignant neoplasms, stated or presumed to be primary, of lymphoid, haematopoietic and related tissue | 19,712 | 4.3 | (4.3) | 6,521 | 5.8 | (4.4) | 184 | 0.8 | (3.8) | 8,443 | 4.2 | (4.3) | 2,785 | 4.2 | (4.6) | 1,276 | 4.0 | (4.6) | 503 | 2.5 | (2.9) |
|  | **D – In situ and benign neoplasms, and neoplasms of uncertain or unknown behaviour and diseases of the blood and blood–forming organs and certain disorders involving the immune mechanism** | **116,560** | **25.6** | **(25.7)** | **40,941** | **36.3** | **(29.6)** | **947** | **3.9** | **(23.7)** | **47,178** | **23.6** | **(25.1)** | **15,779** | **24.0** | **(22.5)** | **6,187** | **19.3** | **(21.5)** | **5,528** | **27.4** | **(27.4)** |
| 19 | In situ neoplasms | 19,810 | 4.3 | (4.4) | 5,309 | 4.7 | (4.0) | 23 | 0.1 | (4.0) | 8,885 | 4.4 | (4.7) | 3,772 | 5.7 | (4.6) | 1,377 | 4.3 | (4.4) | 444 | 2.2 | (2.8) |
| 20 | Haemolytic anaemias | 3,055 | 0.7 | (0.7) | 995 | 0.9 | (0.9) | 87 | 0.4 | (0.6) | 1,197 | 0.6 | (0.6) | 388 | 0.6 | (0.5) | 195 | 0.6 | (0.6) | 193 | 1.0 | (0.9) |
| 21 | Aplastic and other anaemias | 14,918 | 3.3 | (3.3) | 6,047 | 5.4 | (3.9) | 62 | 0.3 | (3.4) | 5,571 | 2.8 | (3.1) | 1,669 | 2.5 | (2.7) | 614 | 1.9 | (2.5) | 955 | 4.7 | (4.6) |
| 22 | Other anaemias | 46,613 | 10.2 | (10.3) | 20,597 | 18.3 | (13.1) | 155 | 0.6 | (10.4) | 16,364 | 8.2 | (9.3) | 4,511 | 6.9 | (7.6) | 1,478 | 4.6 | (7.0) | 3,508 | 17.4 | (16.4) |
| 23 | Coagulation defects, purpura and other haemorrhagic conditions | 25,376 | 5.6 | (5.6) | 6,842 | 6.1 | (6.1) | 491 | 2.0 | (4.1) | 11,411 | 5.7 | (5.7) | 4,119 | 6.3 | (5.5) | 1,912 | 6.0 | (5.5) | 601 | 3.0 | (3.0) |
| 24 | Other diseases of blood and blood–forming organs | 8,896 | 2.0 | (2.0) | 3,095 | 2.7 | (2.4) | 75 | 0.3 | (1.8) | 3,814 | 1.9 | (1.9) | 1,224 | 1.9 | (1.7) | 457 | 1.4 | (1.5) | 231 | 1.1 | (1.4) |
| 25 | Certain disorders involving the immune mechanism | 7,660 | 1.7 | (1.7) | 2,123 | 1.9 | (1.9) | 102 | 0.4 | (1.5) | 3,562 | 1.8 | (1.7) | 1,170 | 1.8 | (1.6) | 560 | 1.7 | (1.5) | 143 | 0.7 | (0.9) |
|  | **E – Endocrine, nutritional and metabolic diseases** | **877,433** | **192.6** | **(192.7)** | **323,816** | **286.9** | **(225.8)** | **3,416** | **14.0** | **(194.3)** | **376,398** | **188.0** | **(193.3)** | **108,995** | **165.8** | **(164.6)** | **38,834** | **120.9** | **(146.8)** | **25,974** | **128.6** | **(162.6)** |
| 26 | Diseases of the thyroid ^c^ | 131,908 | 29.0 | (29.0) | 44,216 | 39.2 | (30.7) | 733 | 3.0 | (27.8) | 52,859 | 26.4 | (28.7) | 22,248 | 33.9 | (28.5) | 7,002 | 21.8 | (27.1) | 4,850 | 24.0 | (28.0) |
| 27 | Thyrotoxicosis ^c^ | 41,374 | 9.1 | (9.0) | 16,176 | 14.3 | (11.4) | 158 | 0.6 | (8.8) | 15,964 | 8.0 | (8.8) | 5,664 | 8.6 | (7.2) | 1,879 | 5.8 | (7.4) | 1,533 | 7.6 | (7.9) |
| 28 | Diabetes type 1 ^c^ | 23,062 | 5.1 | (5.1) | 6,012 | 5.3 | (5.5) | 943 | 3.9 | (4.6) | 11,021 | 5.5 | (5.3) | 3,187 | 4.8 | (4.9) | 1,541 | 4.8 | (4.4) | 358 | 1.8 | (2.0) |
| 29 | Diabetes type 2 ^c^ | 242,177 | 53.2 | (53.3) | 102,366 | 90.7 | (73.6) | 109 | 0.4 | (57.1) | 100,099 | 50.0 | (50.6) | 22,646 | 34.5 | (35.5) | 7,735 | 24.1 | (29.1) | 9,222 | 45.7 | (55.9) |
| 30 | Diabetes others ^c^ | 1,117 | 0.2 | (0.2) | 386 | 0.3 | (0.3) | 35 | 0.1 | (0.3) | 464 | 0.2 | (0.2) | 129 | 0.2 | (0.2) | 52 | 0.2 | (0.2) | 51 | 0.3 | (0.3) |
| 31 | Disorders of other endocrine glands | 28,650 | 6.3 | (6.4) | 8,276 | 7.3 | (7.3) | 1,105 | 4.5 | (5.5) | 11,992 | 6.0 | (6.3) | 4,669 | 7.1 | (6.2) | 1,815 | 5.6 | (5.5) | 793 | 3.9 | (3.3) |
| 32 | Metabolic disorders | 23,690 | 5.2 | (5.2) | 7,872 | 7.0 | (6.4) | 338 | 1.4 | (4.3) | 9,889 | 4.9 | (5.0) | 3,460 | 5.3 | (4.7) | 1,391 | 4.3 | (4.3) | 740 | 3.7 | (4.0) |
| 33 | Disturbances in lipoprotein circulation and other lipids ^c^ | 652,242 | 143.2 | (143.1) | 252,468 | 223.7 | (169.4) | 189 | 0.8 | (147.9) | 283,459 | 141.5 | (145.1) | 73,710 | 112.1 | (118.8) | 25,487 | 79.3 | (103.7) | 16,929 | 83.8 | (114.1) |
| 34 | Cystic fibrosis ^c^ | 947 | 0.2 | (0.2) | 234 | 0.2 | (0.2) | 58 | 0.2 | (0.2) | 341 | 0.2 | (0.2) | 156 | 0.2 | (0.2) | 140 | 0.4 | 0.4) | 18 | 0.1 | (0.1) |
|  | **G – Diseases of the nervous system** | **561,054** | **123.2** | **(123.5)** | **186,070** | **164.9** | **(152.2)** | **5,128** | **21.0** | **(113.5)** | **246,350** | **123.0** | **(122.7)** | **79,664** | **121.2** | **(105.3)** | **28,699** | **89.3** | **(87.2)** | **15,143** | **75.0** | **(93.1)** |
| 35 | Inflammatory diseases of the central nervous system | 7,642 | 1.7 | (1.7) | 2,292 | 2.0 | (1.9) | 84 | 0.3 | (1.4) | 3,432 | 1.7 | (1.7) | 1,128 | 1.7 | (1.6) | 516 | 1.6 | (1.5) | 190 | 0.9 | (1.1) |
| 36 | Systemic atrophies primarily affecting the central nervous system and other degenerative diseases | 10,401 | 2.3 | (2.3) | 4,214 | 3.7 | (2.5) | 26 | 0.1 | (2.2) | 3,965 | 2.0 | (2.2) | 1,100 | 1.7 | (2.0) | 538 | 1.7 | (2.3) | 558 | 2.8 | (2.5) |
| 37 | Parkinson’s disease ^c^ | 57,583 | 12.6 | (12.6) | 27,135 | 24.0 | (20.5) | 81 | 0.3 | (12.8) | 20,602 | 10.3 | (10.6) | 5,633 | 8.6 | (8.2) | 1,899 | 5.9 | (6.9) | 2,233 | 11.1 | (12.3) |
| 38 | Extrapyramidal and movement disorders | 10,837 | 2.4 | (2.4) | 3,997 | 3.5 | (2.9) | 91 | 0.4 | (2.1) | 4,343 | 2.2 | (2.3) | 1,499 | 2.3 | (2.2) | 563 | 1.8 | (2.0) | 344 | 1.7 | (1.8) |
| 39 | Sclerosis | 13,284 | 2.9 | (2.9) | 3,535 | 3.1 | (3.2) | 26 | 0.1 | (2.7) | 6,284 | 3.1 | (3.1) | 2,354 | 3.6 | (2.7) | 925 | 2.9 | (2.5) | 160 | 0.8 | (1.5) |
| 40 | Demyelinating diseases of the central nervous system | 4,571 | 1.0 | (1.0) | 1,155 | 1.0 | (1.1) | 26 | 0.1 | (0.9) | 2,242 | 1.1 | (1.1) | 758 | 1.2 | (0.9) | 331 | 1.0 | (0.8) | 59 | 0.3 | (0.5) |
| 41 | Epilepsy ^c^ | 61,695 | 13.5 | (13.6) | 26,815 | 23.8 | (23.1) | 1,395 | 5.7 | (12.9) | 22,516 | 11.2 | (11.0) | 6,330 | 9.6 | (8.8) | 2,194 | 6.8 | (6.1) | 2,445 | 12.1 | (13.1) |
| 42 | Migraine ^c^ | 149,866 | 32.9 | (33.0) | 38,243 | 33.9 | (34.9) | 1,689 | 6.9 | (27.1) | 68,540 | 34.2 | (34.4) | 28,913 | 44.0 | (33.1) | 10,093 | 31.4 | (29.6) | 2,388 | 11.8 | (18.4) |
| 43 | Other headache syndromes | 16,469 | 3.6 | (3.6) | 4,690 | 4.2 | (4.6) | 479 | 2.0 | (2.8) | 7,594 | 3.8 | (3.7) | 2,443 | 3.7 | (3.0) | 828 | 2.6 | (2.0) | 435 | 2.2 | (2.2) |
| 44 | Transient cerebral ischaemic attacks and related syndromes and vascular syndromes of brain in cerebrovascular diseases | 43,977 | 9.7 | (9.7) | 16,484 | 14.6 | (10.4) | 18 | 0.1 | (9.7) | 18,422 | 9.2 | (9.7) | 5,271 | 8.0 | (9.0) | 2,086 | 6.5 | (8.3) | 1,696 | 8.4 | (9.3) |
| 45 | Sleep disorders | 36,806 | 8.1 | (8.1) | 9,770 | 8.7 | (8.5) | 259 | 1.1 | (7.7) | 18,764 | 9.4 | (8.5) | 5,023 | 7.6 | (7.9) | 2,229 | 6.9 | (5.7) | 761 | 3.8 | (5.8) |
| 46 | Disorders of trigeminal nerve and facial nerve disorders | 21,488 | 4.7 | (4.7) | 6,754 | 6.0 | (5.4) | 157 | 0.6 | (4.3) | 9,642 | 4.8 | (4.8) | 3,110 | 4.7 | (4.3) | 1,207 | 3.8 | (3.6) | 618 | 3.1 | (3.6) |
| 47 | Disorders of other cranial nerves, cranial nerve disorders in diseases classified elsewhere, nerve root and plexus disorders and nerve root and plexus compressions in diseases classified elsewhere | 12,429 | 2.7 | (2.7) | 3,930 | 3.5 | (3.2) | 27 | 0.1 | (2.6) | 5,906 | 2.9 | (2.9) | 1,637 | 2.5 | (2.2) | 635 | 2.0 | (1.8) | 294 | 1.5 | (2.1) |
| 48 | Mononeuropathies of upper limb | 122,395 | 26.9 | (26.9) | 43,178 | 38.3 | (35.4) | 239 | 1.0 | (27.7) | 56,608 | 28.3 | (27.9) | 15,343 | 23.3 | (18.2) | 3,900 | 12.1 | (11.1) | 3,127 | 15.5 | (21.4) |
| 49 | Mononeuropathies of lower limb, other mononeuropathies and mononeuropathy in diseases classified elsewhere | 18,627 | 4.1 | (4.1) | 5,756 | 5.1 | (4.6) | 60 | 0.2 | (3.9) | 8,787 | 4.4 | (4.3) | 2,782 | 4.2 | (3.7) | 902 | 2.8 | (2.7) | 340 | 1.7 | (2.6) |
| 50 | Polyneuropathies and other disorders of the peripheral nervous system | 30,289 | 6.6 | (6.7) | 10,937 | 9.7 | (7.9) | 90 | 0.4 | (6.6) | 13,131 | 6.6 | (6.6) | 3,658 | 5.6 | (5.8) | 1,529 | 4.8 | (5.2) | 944 | 4.7 | (5.7) |
| 51 | Diseases of myoneural junction and muscle | 5,758 | 1.3 | (1.3) | 1,898 | 1.7 | (1.6) | 101 | 0.4 | (1.1) | 2,464 | 1.2 | (1.2) | 806 | 1.2 | (1.1) | 386 | 1.2 | (1.2) | 103 | 0.5 | (0.6) |
| 52 | Cerebral palsy and other paralytic syndromes | 14,410 | 3.2 | (3.2) | 6,846 | 6.1 | (6.2) | 434 | 1.8 | (2.5) | 4,635 | 2.3 | (2.2) | 1,348 | 2.1 | (1.9) | 540 | 1.7 | (1.4) | 607 | 3.0 | (3.1) |
| 53 | Other disorders of the nervous system | 44,394 | 9.7 | (9.8) | 14,788 | 13.1 | (12.0) | 578 | 2.4 | (8.5) | 19,447 | 9.7 | (9.7) | 5,891 | 9.0 | (8.4) | 2,447 | 7.6 | (7.6) | 1,243 | 6.2 | (7.2) |
|  | **H – Diseases of the eye and adnexa and diseases of the ear and mastoid process** | **448,176** | **98.4** | **(98.6)** | **162,019** | **143.6** | **(101.4)** | **2,386** | **9.8** | **(93.4)** | **180,164** | **90.0** | **(97.1)** | **58,044** | **88.3** | **(97.8)** | **24,682** | **76.8** | **(97.1)** | **20,881** | **103.4** | **(104.1)** |
| 54 | Disorders of eyelid, lacrimal system and orbit | 13,191 | 2.9 | (2.9) | 4,338 | 3.8 | (3.0) | 70 | 0.3 | (2.8) | 5,610 | 2.8 | (2.9) | 1,988 | 3.0 | (2.8) | 736 | 2.3 | (2.7) | 449 | 2.2 | (2.7) |
| 55 | Corneal scars and opacities | 2,173 | 0.5 | (0.5) | 734 | 0.7 | (0.5) | 12 | 0.0 | (0.4) | 973 | 0.5 | (0.5) | 244 | 0.4 | (0.4) | 100 | 0.3 | (0.3) | 110 | 0.5 | (0.6) |
| 56 | Other disorders of cornea | 9,473 | 2.1 | (2.1) | 3,073 | 2.7 | (1.9) | 50 | 0.2 | (1.8) | 3,861 | 1.9 | (2.1) | 1,346 | 2.0 | (2.2) | 657 | 2.0 | (2.4) | 486 | 2.4 | (2.3) |
| 57 | Diseases of the eye lens (cataracts) | 68,009 | 14.9 | (15.1) | 29,764 | 26.4 | (16.3) | 45 | 0.2 | (14.9) | 25,162 | 12.6 | (14.8) | 7,624 | 11.6 | (14.3) | 2,743 | 8.5 | (14.3) | 2,671 | 13.2 | (11.8) |
| 58 | Disorders of the choroid and retina | 1,900 | 0.4 | (0.4) | 582 | 0.5 | (0.4) | 14 | 0.1 | (0.4) | 812 | 0.4 | (0.4) | 299 | 0.5 | (0.5) | 115 | 0.4 | (0.4) | 78 | 0.4 | (0.4) |
| 59 | Retinal vascular occlusions | 10,358 | 2.3 | (2.3) | 4,051 | 3.6 | (2.3) | 6 | 0.0 | (2.3) | 4,066 | 2.0 | (2.2) | 1,183 | 1.8 | (2.2) | 570 | 1.8 | (2.4) | 482 | 2.4 | (2.3) |
| 60 | Other retinal disorders | 68,485 | 15.0 | (15.1) | 27,739 | 24.6 | (14.3) | 66 | 0.3 | (15.0) | 24,792 | 12.4 | (14.8) | 7,701 | 11.7 | (14.7) | 3,189 | 9.9 | (15.6) | 4,998 | 24.7 | (21.9) |
| 61 | Retinal disorders in diseases classified elsewhere | 19,279 | 4.2 | (4.3) | 6,889 | 6.1 | (5.4) | 81 | 0.3 | (4.0) | 8,563 | 4.3 | (4.1) | 2,207 | 3.4 | (3.4) | 954 | 3.0 | (2.9) | 585 | 2.9 | (3.7) |
| 62 | Glaucoma ^c^ | 67,310 | 14.8 | (14.9) | 25,887 | 22.9 | (14.0) | 108 | 0.4 | (14.7) | 26,159 | 13.1 | (14.9) | 8,119 | 12.4 | (14.7) | 3,384 | 10.5 | (15.5) | 3,653 | 18.1 | (17.4) |
| 63 | Disorders of the vitreous body and globe | 7,572 | 1.7 | (1.7) | 2,016 | 1.8 | (1.4) | 43 | 0.2 | (1.4) | 3,210 | 1.6 | (1.6) | 1,347 | 2.0 | (2.0) | 741 | 2.3 | (2.5) | 215 | 1.1 | (1.4) |
| 64 | Disorders of optic nerve and visual pathways | 6,184 | 1.4 | (1.4) | 1,808 | 1.6 | (1.6) | 82 | 0.3 | (1.0) | 2,810 | 1.4 | (1.4) | 933 | 1.4 | (1.2) | 407 | 1.3 | (1.1) | 144 | 0.7 | (0.8) |
| 65 | Disorders of ocular muscles, binocular movement, accommodation and refraction | 18,247 | 4.0 | (4.0) | 3,939 | 3.5 | (3.8) | 467 | 1.9 | (2.7) | 8,332 | 4.2 | (4.1) | 3,097 | 4.7 | (4.3) | 2,026 | 6.3 | (5.9) | 386 | 1.9 | (1.8) |
| 66 | Visual disturbances | 22,232 | 4.9 | (4.9) | 7,555 | 6.7 | (5.7) | 208 | 0.9 | (4.3) | 9,245 | 4.6 | (4.7) | 3,145 | 4.8 | (4.7) | 1,384 | 4.3 | (4.6) | 695 | 3.4 | (3.7) |
| 67 | Blindness and partial sight | 6,614 | 1.5 | (1.5) | 2,855 | 2.5 | (2.0) | 57 | 0.2 | (1.3) | 2,244 | 1.1 | (1.2) | 616 | 0.9 | (1.0) | 277 | 0.9 | (1.1) | 565 | 2.8 | (2.7) |
| 68 | Nystagmus and other irregular eye movements and other disorders of eye and adnexa | 11,133 | 2.4 | (2.5) | 3,232 | 2.9 | (2.6) | 214 | 0.9 | (2.1) | 4,589 | 2.3 | (2.3) | 1,841 | 2.8 | (2.6) | 919 | 2.9 | (3.0) | 338 | 1.7 | (1.9) |
| 69 | Otosclerosis | 10,360 | 2.3 | (2.3) | 3,260 | 2.9 | (2.2) | 59 | 0.2 | (2.2) | 4,366 | 2.2 | (2.3) | 1,697 | 2.6 | (2.4) | 643 | 2.0 | (2.3) | 335 | 1.7 | (2.0) |
| 70 | Ménière’s disease ^c^ | 10,003 | 2.2 | (2.2) | 3,562 | 3.2 | (2.3) | 6 | 0.0 | (2.2) | 4,250 | 2.1 | (2.2) | 1,385 | 2.1 | (2.1) | 488 | 1.5 | (1.9) | 312 | 1.5 | (1.8) |
| 71 | Other diseases of the inner ear | 29,865 | 6.6 | (6.3) | 10,685 | 9.5 | (7.7) | 19 | 0.1 | (7.0) | 15,170 | 7.6 | (7.2) | 2,332 | 3.5 | (4.8) | 879 | 2.7 | (2.5) | 780 | 3.9 | (5.3) |
| 72 | Conductive and sensorineural hearing loss | 43,238 | 9.5 | (9.6) | 15,433 | 13.7 | (10.7) | 614 | 2.5 | (8.5) | 17,383 | 8.7 | (9.1) | 5,688 | 8.7 | (9.3) | 2,312 | 7.2 | (8.7) | 1,808 | 9.0 | (9.5) |
| 73 | Other hearing loss and other disorders of ear, not elsewhere classified | 8,306 | 1.8 | (1.8) | 2,992 | 2.7 | (2.2) | 82 | 0.3 | (1.7) | 3,400 | 1.7 | (1.7) | 1,124 | 1.7 | (1.8) | 405 | 1.3 | (1.4) | 303 | 1.5 | (1.7) |
| 74 | Presbycusis (age–related hearing loss) | 80,659 | 17.7 | (17.6) | 35,451 | 31.4 | (17.1) | 20 | 0.1 | (18.1) | 26,227 | 13.1 | (16.6) | 7,877 | 12.0 | (17.4) | 2,892 | 9.0 | (16.5) | 8,192 | 40.6 | (34.8) |
| 75 | Hearing loss, unspecified | 87,806 | 19.3 | (19.3) | 31,207 | 27.7 | (19.5) | 378 | 1.6 | (18.8) | 36,223 | 18.1 | (19.1) | 11,534 | 17.5 | (19.9) | 4,505 | 14.0 | (17.8) | 3,959 | 19.6 | (21.3) |
| 76 | Tinnitus | 40,124 | 8.8 | (8.7) | 12,187 | 10.8 | (8.9) | 87 | 0.4 | (8.5) | 17,944 | 9.0 | (8.7) | 6,146 | 9.4 | (9.5) | 2,657 | 8.3 | (8.6) | 1,103 | 5.5 | (7.5) |
| 77 | Other specified disorders of ear | 20,537 | 4.5 | (4.4) | 7,573 | 6.7 | (4.8) | 95 | 0.4 | (4.3) | 8,354 | 4.2 | (4.4) | 2,973 | 4.5 | (4.9) | 949 | 3.0 | (3.9) | 593 | 2.9 | (3.4) |
|  | **I – Diseases of the circulatory system** | **1,254,427** | **275.4** | **(275.5)** | **444,581** | **393.9** | **(308.9)** | **2,877** | **11.8** | **(268.1)** | **542,233** | **270.8** | **(279.0)** | **161,971** | **246.4** | **(244.2)** | **60,629** | **188.7** | **(221.7)** | **42,136** | **208.6** | **(248.0)** |
| 78 | Aortic and mitral valve disease ^c^ | 30,123 | 6.6 | (6.6) | 13,077 | 11.6 | (7.7) | 57 | 0.2 | (6.6) | 11,092 | 5.5 | (6.3) | 3,016 | 4.6 | (5.9) | 1,314 | 4.1 | (6.0) | 1,567 | 7.8 | (7.1) |
| 79 | Hypertensive diseases ^c^ | 1,060,046 | 232.7 | (232.7) | 391,287 | 346.7 | (263.7) | 819 | 3.4 | (234.7) | 453,910 | 226.7 | (235.6) | 130,934 | 199.2 | (200.0) | 47,086 | 146.6 | (182.6) | 36,010 | 178.3 | (214.0) |
| 80 | Heart failure ^c^ | 37,540 | 8.2 | (8.3) | 15,906 | 14.1 | (10.2) | 24 | 0.1 | (8.6) | 14,870 | 7.4 | (7.9) | 3,252 | 4.9 | (6.4) | 1,482 | 4.6 | (6.1) | 2,006 | 9.9 | (10.1) |
| 80A | Ischaemic heart diseases | 139,173 | 30.6 | (30.7) | 56,244 | 49.8 | (37.7) | 42 | 0.2 | (31.9) | 58,083 | 29.0 | (29.7) | 13,731 | 20.9 | (24.3) | 5,320 | 16.6 | (20.9) | 5,753 | 28.5 | (32.6) |
| 81 | Angina pectoris | 78,476 | 17.2 | (17.3) | 30,898 | 27.4 | (21.4) | 22 | 0.1 | (17.9) | 33,383 | 16.7 | (16.8) | 8,323 | 12.7 | (13.9) | 3,136 | 9.8 | (11.8) | 2,714 | 13.4 | (16.8) |
| 82 | Acute myocardial infarction and subsequent myocardial infarction | 36,654 | 8.0 | (8.1) | 14,841 | 13.2 | (10.2) | 7 | 0.0 | (8.5) | 15,544 | 7.8 | (7.8) | 3,316 | 5.0 | (6.1) | 1,310 | 4.1 | (4.9) | 1,636 | 8.1 | (9.2) |
| 83 | AMI complex/other | 2,969 | 0.7 | (0.7) | 1,194 | 1.1 | (0.8) | 0 | 0.0 | (0.7) | 1,224 | 0.6 | (0.6) | 317 | 0.5 | (0.5) | 97 | 0.3 | (0.4) | 135 | 0.7 | (0.8) |
| 84 | Chronic ischaemic heart disease | 84,592 | 18.6 | (18.6) | 35,570 | 31.5 | (23.3) | 12 | 0.0 | (19.6) | 34,750 | 17.4 | (18.0) | 7,447 | 11.3 | (14.3) | 3,073 | 9.6 | (12.5) | 3,740 | 18.5 | (20.2) |
| 85 | Pulmonary heart disease and diseases of pulmonary circulation | 15,352 | 3.4 | (3.4) | 6,122 | 5.4 | (4.1) | 76 | 0.3 | (3.2) | 6,193 | 3.1 | (3.3) | 1,689 | 2.6 | (2.8) | 641 | 2.0 | (2.6) | 631 | 3.1 | (3.1) |
| 86 | Acute pericarditis | 5,563 | 1.2 | (1.2) | 1,588 | 1.4 | (1.4) | 70 | 0.3 | (0.9) | 2,699 | 1.3 | (1.3) | 668 | 1.0 | (1.1) | 375 | 1.2 | (1.0) | 163 | 0.8 | (0.8) |
| 87 | Other forms of heart disease | 8,119 | 1.8 | (1.8) | 2,931 | 2.6 | (2.1) | 53 | 0.2 | (1.5) | 3,424 | 1.7 | (1.7) | 929 | 1.4 | (1.6) | 473 | 1.5 | (1.6) | 309 | 1.5 | (1.6) |
| 88 | Atrioventricular and left bundle branch block | 14,604 | 3.2 | (3.2) | 5,903 | 5.2 | (3.4) | 21 | 0.1 | (3.2) | 5,457 | 2.7 | (3.1) | 1,419 | 2.2 | (2.9) | 677 | 2.1 | (2.9) | 1,127 | 5.6 | (5.2) |
| 89 | Other conduction disorders | 11,823 | 2.6 | (2.6) | 4,182 | 3.7 | (2.9) | 111 | 0.5 | (2.4) | 5,029 | 2.5 | (2.6) | 1,289 | 2.0 | (2.3) | 662 | 2.1 | (2.3) | 550 | 2.7 | (2.7) |
| 90 | Paroxysmal tachycardia | 39,510 | 8.7 | (8.7) | 12,836 | 11.4 | (8.9) | 321 | 1.3 | (7.7) | 16,959 | 8.5 | (8.8) | 5,798 | 8.8 | (9.1) | 2,399 | 7.5 | (8.5) | 1,197 | 5.9 | (6.7) |
| 91 | Atrial fibrillation and flutter | 112,342 | 24.7 | (24.7) | 44,765 | 39.7 | (25.8) | 40 | 0.2 | (24.5) | 43,307 | 21.6 | (23.9) | 12,441 | 18.9 | (24.1) | 5,768 | 18.0 | (24.5) | 6,021 | 29.8 | (28.8) |
| 92 | Other cardiac arrhythmias | 34,418 | 7.6 | (7.6) | 11,871 | 10.5 | (7.6) | 112 | 0.5 | (6.9) | 13,963 | 7.0 | (7.4) | 4,989 | 7.6 | (8.1) | 2,086 | 6.5 | (7.7) | 1,397 | 6.9 | (7.1) |
| 93 | Complications and ill–defined descriptions of heart disease and other heart disorders in diseases classified elsewhere | 7,337 | 1.6 | (1.6) | 2,659 | 2.4 | (1.8) | 37 | 0.2 | (1.4) | 2,932 | 1.5 | (1.5) | 946 | 1.4 | (1.6) | 453 | 1.4 | (1.6) | 310 | 1.5 | (1.6) |
| 94 | Stroke | 72,606 | 15.9 | (16.0) | 29,883 | 26.5 | (19.2) | 40 | 0.2 | (16.5) | 29,511 | 14.7 | (15.7) | 7,057 | 10.7 | (12.6) | 2,783 | 8.7 | (11.7) | 3,332 | 16.5 | (17.4) |
| 95 | Cerebrovascular diseases | 17,308 | 3.8 | (3.8) | 6,554 | 5.8 | (4.4) | 45 | 0.2 | (3.7) | 7,229 | 3.6 | (3.8) | 2,034 | 3.1 | (3.3) | 901 | 2.8 | (3.4) | 545 | 2.7 | (3.1) |
| 96 | Sequelae of cerebrovascular disease | 50,952 | 11.2 | (11.2) | 22,350 | 19.8 | (14.0) | 24 | 0.1 | (11.7) | 19,676 | 9.8 | (10.8) | 4,534 | 6.9 | (8.5) | 1,660 | 5.2 | (7.8) | 2,708 | 13.4 | (13.3) |
| 97 | Atherosclerosis | 32,064 | 7.0 | (7.0) | 15,289 | 13.5 | (9.9) | 9 | 0.0 | (7.5) | 12,394 | 6.2 | (6.7) | 2,270 | 3.5 | (4.4) | 705 | 2.2 | (3.8) | 1,397 | 6.9 | (7.1) |
| 98 | Aortic aneurysm and aortic dissection | 10,296 | 2.3 | (2.3) | 3,965 | 3.5 | (2.5) | 7 | 0.0 | (2.3) | 4,544 | 2.3 | (2.4) | 1,032 | 1.6 | (2.0) | 462 | 1.4 | (1.8) | 286 | 1.4 | (1.6) |
| 99 | Diseases of arteries, arterioles and capillaries | 11,830 | 2.6 | (2.6) | 3,764 | 3.3 | (2.8) | 57 | 0.2 | (2.3) | 5,161 | 2.6 | (2.6) | 1,766 | 2.7 | (2.6) | 752 | 2.3 | (2.5) | 330 | 1.6 | (2.0) |
| 100 | Other peripheral vascular diseases | 28,508 | 6.3 | (6.3) | 12,644 | 11.2 | (8.5) | 72 | 0.3 | (6.7) | 11,930 | 6.0 | (6.2) | 2,245 | 3.4 | (4.0) | 746 | 2.3 | (3.4) | 871 | 4.3 | (4.9) |
| 101 | Phlebitis, thrombosis of the portal vein and others | 37,388 | 8.2 | (8.3) | 13,747 | 12.2 | (10.1) | 205 | 0.8 | (7.8) | 16,171 | 8.1 | (8.3) | 4,230 | 6.4 | (6.3) | 1,709 | 5.3 | (5.8) | 1,326 | 6.6 | (7.2) |
| 102 | Varicose veins of lower extremities | 23,530 | 5.2 | (5.2) | 6,589 | 5.8 | (5.4) | 67 | 0.3 | (4.8) | 11,062 | 5.5 | (5.5) | 4,072 | 6.2 | (5.1) | 1,169 | 3.6 | (3.4) | 571 | 2.8 | (3.8) |
| 103 | Haemorrhoids ^c^ | 74,285 | 16.3 | (16.3) | 18,741 | 16.6 | (16.5) | 616 | 2.5 | (11.9) | 33,957 | 17.0 | (16.9) | 13,323 | 20.3 | (17.9) | 5,238 | 16.3 | (14.5) | 2,410 | 11.9 | (12.2) |
| 104 | Oesophageal varices (chronic), varicose veins of other sites, other disorders of veins, non–specific lymphadenitis, other non–infective disorders of lymphatic vessels and lymph nodes and other and unspecified disorders of the circulatory system | 15,194 | 3.3 | (3.3) | 4,972 | 4.4 | (3.9) | 283 | 1.2 | (2.6) | 6,685 | 3.3 | (3.4) | 1,913 | 2.9 | (3.0) | 840 | 2.6 | (2.8) | 501 | 2.5 | (2.7) |
|  | **J – Diseases of the respiratory system** | **1,210,598** | **265.7** | **(266.3)** | **336,462** | **298.1** | **(278.0)** | **27,122** | **111.3** | **(217.6)** | **536,766** | **268.0** | **(271.0)** | **192,347** | **292.7** | **(274.5)** | **88,121** | **274.3** | **(275.5)** | **29,780** | **147.4** | **(159.2)** |
| 105 | Respiratory allergy ^c^ | 841,685 | 184.8 | (185.2) | 207,831 | 184.2 | (172.6) | 17,715 | 72.7 | (140.4) | 379,413 | 189.5 | (191.7) | 146,324 | 222.6 | (208.6) | 70,172 | 218.4 | (218.2) | 20,230 | 100.2 | (106.9) |
| 105A | Chronic lower respiratory diseases ^c^ | 418,120 | 91.8 | (92.0) | 136,583 | 121.0 | (110.3) | 3,035 | 12.5 | (83.3) | 177,500 | 88.6 | (89.3) | 64,207 | 97.7 | (86.6) | 27,404 | 85.3 | (83.4) | 9,391 | 46.5 | (54.1) |
| 106 | Bronchitis, not specified as acute or chronic, simple and mucopurulent chronic bronchitis  and unspecified chronic bronchitis | 12,790 | 2.8 | (2.8) | 6,448 | 5.7 | (4.5) | 12 | 0.0 | (3.0) | 4,503 | 2.2 | (2.4) | 1,017 | 1.5 | (1.6) | 277 | 0.9 | (1.4) | 533 | 2.6 | (2.8) |
| 107 | Emphysema | 5,557 | 1.2 | (1.2) | 2,489 | 2.2 | (1.8) | 8 | 0.0 | (1.3) | 2,224 | 1.1 | (1.1) | 518 | 0.8 | (0.8) | 153 | 0.5 | (0.6) | 165 | 0.8 | (1.0) |
| 108 | Chronic obstructive lung disease (COPD) ^c^ | 216,184 | 47.5 | (47.6) | 88,085 | 78.0 | (64.7) | 2,114 | 8.7 | (41.4) | 88,954 | 44.4 | (46.2) | 23,220 | 35.3 | (36.0) | 7,600 | 23.7 | (30.1) | 6,211 | 30.7 | (35.3) |
| 109 | Asthma, status asthmaticus ^c^ | 361,129 | 79.3 | (79.4) | 115,623 | 102.4 | (96.5) | 11,545 | 47.4 | (69.1) | 151,531 | 75.7 | (77.0) | 52,295 | 79.6 | (75.8) | 21,606 | 67.3 | (69.4) | 8,529 | 42.2 | (44.9) |
| 110 | Bronchiectasis | 4,362 | 1.0 | (1.0) | 1,355 | 1.2 | (0.9) | 21 | 0.1 | (0.9) | 1,814 | 0.9 | (1.0) | 781 | 1.2 | (1.2) | 273 | 0.8 | (1.0) | 118 | 0.6 | (0.8) |
| 111 | Other diseases of the respiratory system | 21,993 | 4.8 | (4.9) | 8,626 | 7.6 | (6.2) | 138 | 0.6 | (4.8) | 9,224 | 4.6 | (4.7) | 2,320 | 3.5 | (3.7) | 815 | 2.5 | (3.0) | 870 | 4.3 | (4.8) |
|  | **K – Diseases of the digestive system** | **329,337** | **72.3** | **(72.6)** | **113,926** | **100.9** | **(88.5)** | **3,790** | **15.6** | **(62.6)** | **141,162** | **70.5** | **(71.8)** | **42,201** | **64.2** | **(61.7)** | **15,438** | **48.1** | **(50.7)** | **12,820** | **63.5** | **(67.8)** |
| 112 | Ulcers ^c^ | 157,379 | 34.5 | (34.8) | 61,390 | 54.4 | (45.9) | 1,514 | 6.2 | (33.1) | 63,596 | 31.8 | (32.8) | 17,288 | 26.3 | (25.6) | 5,758 | 17.9 | (20.8) | 7,833 | 38.8 | 41.3) |
| 113 | Inguinal hernia | 25,032 | 5.5 | (5.5) | 6,910 | 6.1 | (5.4) | 157 | 0.6 | (5.0) | 12,276 | 6.1 | (5.7) | 3,073 | 4.7 | (5.7) | 1,878 | 5.8 | (5.4) | 738 | 3.7 | (4.7) |
| 114 | Ventral hernia | 7,941 | 1.7 | (1.7) | 2,962 | 2.6 | (2.3) | 31 | 0.1 | (1.8) | 3,430 | 1.7 | (1.7) | 973 | 1.5 | (1.3) | 312 | 1.0 | (1.0) | 233 | 1.2 | (1.5) |
| 115 | Crohn’s disease | 18,913 | 4.2 | (4.2) | 5,052 | 4.5 | (4.7) | 382 | 1.6 | (2.8) | 9,017 | 4.5 | (4.5) | 2,920 | 4.4 | (3.9) | 1,205 | 3.8 | (3.3) | 337 | 1.7 | (1.6) |
| 116 | Ulcerative colitis | 29,538 | 6.5 | (6.5) | 7,511 | 6.7 | (6.4) | 367 | 1.5 | (4.7) | 14,130 | 7.1 | (7.0) | 4,896 | 7.4 | (6.9) | 2,135 | 6.6 | (6.2) | 499 | 2.5 | (2.6) |
| 117 | Other non–infective gastroenteritis and colitis | 20,844 | 4.6 | (4.6) | 7,733 | 6.9 | (5.6) | 300 | 1.2 | (3.7) | 8,672 | 4.3 | (4.7) | 2,602 | 4.0 | (3.9) | 810 | 2.5 | (3.2) | 727 | 3.6 | (3.5) |
| 118 | Irritable bowel syndrome (IBS) | 37,593 | 8.3 | (8.3) | 10,531 | 9.3 | (9.3) | 469 | 1.9 | (4.9) | 17,320 | 8.6 | (8.8) | 6,521 | 9.9 | (8.5) | 2,040 | 6.4 | (6.0) | 712 | 3.5 | (3.5) |
| 119 | Other functional intestinal disorders | 51,933 | 11.4 | (11.5) | 19,528 | 17.3 | (14.5) | 801 | 3.3 | (9.4) | 20,618 | 10.3 | (11.0) | 6,341 | 9.6 | (9.5) | 2,129 | 6.6 | (8.0) | 2,516 | 12.5 | (12.1) |
| 120 | Diseases of liver, biliary tract and pancreas | 26,956 | 5.9 | (6.0) | 9,766 | 8.7 | (7.8) | 175 | 0.7 | (5.9) | 11,949 | 6.0 | (5.8) | 3,118 | 4.7 | (4.3) | 1,097 | 3.4 | (3.4) | 851 | 4.2 | (5.5) |
|  | **L – Diseases of the skin and subcutaneous tissue** | 65,469 | 14.4 | (14.5) | 18,801 | 16.7 | (14.7) | 554 | 2.3 | (12.5) | 30,052 | 15.0 | (14.9) | 9,957 | 15.1 | (14.4) | 4,593 | 14.3 | (14.4) | 1,512 | 7.5 | (9.6) |
| 121 | Psoriasis ^c^ | 65,469 | 14.4 | (14.5) | 18,801 | 16.7 | (14.7) | 554 | 2.3 | (12.5) | 30,052 | 15.0 | (14.9) | 9,957 | 15.1 | (14.4) | 4,593 | 14.3 | (14.4) | 1,512 | 7.5 | (9.6) |
|  | **M – Diseases of the musculoskeletal system and connective tissue** | **1,032,808** | **226.7** | **(227.1)** | **332,557** | **294.7** | **(246.7)** | **14,897** | **61.1** | **(211.4)** | **459,460** | **229.4** | **(234.9)** | **145,773** | **221.8** | **(214.8)** | **51,276** | **159.6** | **(179.6)** | **28,845** | **142.8** | **(165.5)** |
| 122 | Infectious arthropathies | 9,402 | 2.1 | (2.1) | 2,317 | 2.1 | (2.1) | 92 | 0.4 | (1.5) | 4,525 | 2.3 | (2.2) | 1,550 | 2.4 | (2.2) | 720 | 2.2 | (2.0) | 198 | 1.0 | (1.1) |
| 122A | Inflammatory polyarthropathies and ankylosing spondylitis ^c^ | 165,944 | 36.4 | (36.5) | 55,657 | 49.3 | (40.8) | 753 | 3.1 | (35.2) | 74,070 | 37.0 | (37.0) | 21,273 | 32.4 | (32.2) | 9,676 | 30.1 | (31.9) | 4,515 | 22.4 | (28.2) |
| 123 | Rheumatoid arthritis ^c^ | 77,345 | 17.0 | (17.0) | 24,192 | 21.4 | (18.0) | 588 | 2.4 | (15.7) | 32,885 | 16.4 | (16.9) | 12,368 | 18.8 | (16.9) | 5,714 | 17.8 | (19.1) | 1,598 | 7.9 | (10.6) |
| 124 | Inflammatory polyarthropathies – except rheumatoid arthritis ^c^ | 115,945 | 25.5 | (25.5) | 39,923 | 35.4 | (29.4) | 276 | 1.1 | (24.7) | 53,152 | 26.5 | (26.3) | 13,564 | 20.6 | (21.4) | 5,659 | 17.6 | (18.4) | 3,371 | 16.7 | (20.7) |
| 125 | Polyarthrosis [arthrosis] | 16,935 | 3.7 | (3.7) | 6,680 | 5.9 | (3.9) | 0 | 0.0 | (3.8) | 6,787 | 3.4 | (3.8) | 2,319 | 3.5 | (3.4) | 545 | 1.7 | (3.1) | 600 | 3.0 | (3.5) |
| 126 | Coxarthrosis [arthrosis of hip] | 104,115 | 22.9 | (22.7) | 44,227 | 39.2 | (26.4) | 71 | 0.3 | (22.9) | 39,709 | 19.8 | (22.3) | 12,162 | 18.5 | (21.5) | 3,824 | 11.9 | (18.8) | 4,122 | 20.4 | (20.1) |
| 127 | Gonarthrosis [arthrosis of knee] | 178,811 | 39.3 | (39.4) | 64,860 | 57.5 | (42.4) | 73 | 0.3 | (40.1) | 78,639 | 39.3 | (40.8) | 23,262 | 35.4 | (36.1) | 6,420 | 20.0 | (27.3) | 5,557 | 27.5 | (34.3) |
| 128 | Arthrosis of first carpometacarpal joint and other arthrosis | 91,101 | 20.0 | (20.1) | 32,138 | 28.5 | (22.3) | 93 | 0.4 | (20.2) | 41,042 | 20.5 | (20.9) | 12,070 | 18.4 | (17.5) | 3,149 | 9.8 | (12.6) | 2,609 | 12.9 | (17.3) |
| 129 | Acquired deformities of fingers and toes | 55,730 | 12.2 | (12.3) | 16,302 | 14.4 | (11.8) | 488 | 2.0 | (10.7) | 25,441 | 12.7 | (13.3) | 9,762 | 14.9 | (12.7) | 2,630 | 8.2 | (10.0) | 1,107 | 5.5 | (8.0) |
| 130 | Other acquired deformities of limbs | 20,584 | 4.5 | (4.5) | 6,564 | 5.8 | (5.1) | 424 | 1.7 | (3.8) | 9,156 | 4.6 | (4.7) | 3,168 | 4.8 | (4.4) | 857 | 2.7 | (3.1) | 415 | 2.1 | (2.5) |
| 131 | Disorders of patella (knee cap) | 38,999 | 8.6 | (8.6) | 8,826 | 7.8 | (10.0) | 2,452 | 10.1 | (3.0) | 19,314 | 9.6 | (9.7) | 5,728 | 8.7 | (8.3) | 2,245 | 7.0 | (6.3) | 434 | 2.1 | (0.1) |
| 132 | Internal derangement of knee | 9,192 | 2.0 | (2.0) | 1,772 | 1.6 | (1.9) | 579 | 2.4 | (1.2) | 4,508 | 2.3 | (2.2) | 1,463 | 2.2 | (2.4) | 680 | 2.1 | (2.0) | 190 | 0.9 | (0.7) |
| 133 | Derangement of meniscus due to old tear or injury | 36,374 | 8.0 | (8.0) | 8,064 | 7.1 | (7.6) | 1,148 | 4.7 | (6.9) | 18,285 | 9.1 | (8.7) | 5,769 | 8.8 | (8.5) | 2,467 | 7.7 | (7.1) | 641 | 3.2 | (4.2) |
| 134 | Internal derangement of knee, unspecified | 28,206 | 6.2 | (6.2) | 6,488 | 5.7 | (6.4) | 1,798 | 7.4 | (6.9) | 13,726 | 6.9 | (6.7) | 4,109 | 6.3 | (6.1) | 1,551 | 4.8 | (4.4) | 534 | 2.6 | (2.7) |
| 135 | Other specific joint derangements | 5,923 | 1.3 | (1.3) | 1,325 | 1.2 | (1.3) | 440 | 1.8 | (0.7) | 2,829 | 1.4 | (1.4) | 782 | 1.2 | (1.3) | 401 | 1.2 | (1.3) | 146 | 0.7 | (0.5) |
| 136 | Other joint disorders, not elsewhere classified | 12,043 | 2.6 | (2.7) | 3,345 | 3.0 | (3.1) | 427 | 1.8 | (2.6) | 5,629 | 2.8 | (2.8) | 1,770 | 2.7 | (2.2) | 591 | 1.8 | (1.7) | 281 | 1.4 | (1.6) |
| 137 | Systemic connective tissue disorders | 42,631 | 9.4 | (9.4) | 15,606 | 13.8 | (10.5) | 307 | 1.3 | (8.6) | 16,872 | 8.4 | (9.4) | 6,444 | 9.8 | (9.1) | 1,965 | 6.1 | (7.8) | 1,437 | 7.1 | (6.9) |
| 138 | Systemic lupus erythematosus | 3,376 | 0.7 | (0.7) | 1,040 | 0.9 | (0.9) | 32 | 0.1 | (0.7) | 1,401 | 0.7 | (0.7) | 648 | 1.0 | (0.7) | 181 | 0.6 | (0.6) | 74 | 0.4 | (0.5) |
| 139 | Dermatopolymyositis | 1,137 | 0.2 | (0.2) | 381 | 0.3 | (0.3) | 8 | 0.0 | (0.2) | 490 | 0.2 | (0.2) | 157 | 0.2 | (0.2) | 72 | 0.2 | (0.2) | 29 | 0.1 | (0.2) |
| 140 | Systemic sclerosis | 1,675 | 0.4 | (0.4) | 611 | 0.5 | (0.5) | 12 | 0.0 | (0.4) | 712 | 0.4 | (0.4) | 258 | 0.4 | (0.3) | 65 | 0.2 | (0.3) | 17 | 0.1 | (0.2) |
| 141 | Kyphosis, lordosis | 4,160 | 0.9 | (0.9) | 1,327 | 1.2 | (1.2) | 88 | 0.4 | (0.8) | 1,710 | 0.9 | (0.8) | 649 | 1.0 | (0.9) | 291 | 0.9 | (0.8) | 95 | 0.5 | (0.6) |
| 142 | Scoliosis | 17,686 | 3.9 | (3.9) | 5,287 | 4.7 | (4.8) | 1,475 | 6.1 | (2.9) | 7,016 | 3.5 | (3.8) | 2,491 | 3.8 | (3.9) | 1,059 | 3.3 | (3.8) | 358 | 1.8 | (1.1) |
| 143 | Spinal osteochondrosis | 8,034 | 1.8 | (1.8) | 2,616 | 2.3 | (2.4) | 118 | 0.5 | (1.6) | 3,724 | 1.9 | (1.7) | 996 | 1.5 | (1.5) | 423 | 1.3 | (1.0) | 157 | 0.8 | (1.0) |
| 144 | Other deforming dorsopathies | 23,756 | 5.2 | (5.3) | 8,159 | 7.2 | (6.1) | 119 | 0.5 | (5.1) | 10,515 | 5.3 | (5.3) | 3,296 | 5.0 | (4.7) | 1,031 | 3.2 | (3.5) | 636 | 3.1 | (4.0) |
| 145 | Other inflammatory spondylopathies | 7,086 | 1.6 | (1.6) | 2,144 | 1.9 | (1.8) | 54 | 0.2 | (1.4) | 3,287 | 1.6 | (1.6) | 1,028 | 1.6 | (1.4) | 409 | 1.3 | (1.1) | 164 | 0.8 | (1.0) |
| 146 | Spondylosis | 61,999 | 13.6 | (13.6) | 23,358 | 20.7 | (16.9) | 30 | 0.1 | (14.2) | 27,273 | 13.6 | (13.7) | 7,452 | 11.3 | (10.8) | 2,108 | 6.6 | (7.9) | 1,778 | 8.8 | (11.9) |
| 147 | Other spondylopathies and spondylopathies in diseases classified elsewhere | 50,805 | 11.2 | (11.2) | 19,916 | 17.6 | (12.3) | 18 | 0.1 | (11.3) | 21,285 | 10.6 | (11.5) | 5,980 | 9.1 | (10.0) | 1,990 | 6.2 | (8.9) | 1,616 | 8.0 | (8.8) |
| 148 | Cervical disc disorders | 11,476 | 2.5 | (2.5) | 3,049 | 2.7 | (2.8) | 13 | 0.1 | (2.5) | 5,873 | 2.9 | (2.7) | 1,698 | 2.6 | (2.1) | 607 | 1.9 | (1.4) | 236 | 1.2 | (1.9) |
| 149 | Other intervertebral disc disorders | 40,161 | 8.8 | (8.9) | 11,706 | 10.4 | (10.4) | 199 | 0.8 | (8.3) | 19,725 | 9.8 | (9.4) | 5,590 | 8.5 | (7.3) | 1,921 | 6.0 | (4.6) | 1,020 | 5.0 | (6.4) |
| 150 | Other dorsopathies, not elsewhere classified | 7,246 | 1.6 | (1.6) | 2,057 | 1.8 | (1.9) | 157 | 0.6 | (1.7) | 3,533 | 1.8 | (1.7) | 988 | 1.5 | (1.2) | 334 | 1.0 | (0.8) | 177 | 0.9 | (1.0) |
| 151 | Dorsalgia | 40,780 | 9.0 | (9.0) | 12,850 | 11.4 | (11.2) | 719 | 3.0 | (8.6) | 18,848 | 9.4 | (9.3) | 5,141 | 7.8 | (6.7) | 1,853 | 5.8 | (4.9) | 1,369 | 6.8 | (7.2) |
| 152 | Soft tissue disorders | 13,422 | 2.9 | (3.0) | 4,102 | 3.6 | (3.9) | 470 | 1.9 | (2.2) | 5,933 | 3.0 | (3.0) | 1,862 | 2.8 | (2.2) | 602 | 1.9 | (1.6) | 453 | 2.2 | (2.2) |
| 153 | Synovitis and tenosynovitis | 19,104 | 4.2 | (4.2) | 5,362 | 4.8 | (4.6) | 585 | 2.4 | (4.0) | 8,961 | 4.5 | (4.5) | 2,855 | 4.3 | (3.8) | 969 | 3.0 | (3.1) | 372 | 1.8 | (2.4) |
| 154 | Disorders of synovium and tendon | 19,669 | 4.3 | (4.3) | 4,662 | 4.1 | (4.5) | 1,137 | 4.7 | (4.3) | 9,534 | 4.8 | (4.7) | 2,890 | 4.4 | (4.1) | 1,059 | 3.3 | (3.1) | 387 | 1.9 | (1.9) |
| 155 | Soft tissue disorders related to use, overuse and pressure | 11,090 | 2.4 | (2.4) | 3,275 | 2.9 | (2.7) | 331 | 1.4 | (2.6) | 5,184 | 2.6 | (2.6) | 1,620 | 2.5 | (2.3) | 511 | 1.6 | (1.6) | 169 | 0.8 | (1.1) |
| 156 | Fibroblastic disorders | 43,600 | 9.6 | (9.6) | 13,106 | 11.6 | (9.4) | 81 | 0.3 | (9.3) | 20,653 | 10.3 | (10.1) | 6,264 | 9.5 | (10.0) | 2,501 | 7.8 | (8.1) | 995 | 4.9 | (7.1) |
| 157 | Shoulder lesions | 58,112 | 12.8 | (12.7) | 16,538 | 14.7 | (14.6) | 573 | 2.4 | (12.0) | 29,266 | 14.6 | (13.9) | 8,092 | 12.3 | (10.7) | 2,428 | 7.6 | (6.3) | 1,215 | 6.0 | (9.0) |
| 158 | Enthesopathies of lower limb, excluding foot | 11,223 | 2.5 | (2.5) | 2,003 | 1.8 | (2.0) | 592 | 2.4 | (2.3) | 5,294 | 2.6 | (2.6) | 2,120 | 3.2 | (3.2) | 1,063 | 3.3 | (3.2) | 151 | 0.7 | (0.8) |
| 159 | Other enthesopathies | 10,500 | 2.3 | (2.3) | 2,600 | 2.3 | (2.5) | 176 | 0.7 | (2.1) | 5,302 | 2.6 | (2.5) | 1,584 | 2.4 | (2.0) | 644 | 2.0 | (1.7) | 194 | 1.0 | (1.3) |
| 160 | Rheumatism, unspecified | 6,852 | 1.5 | (1.5) | 2,786 | 2.5 | (2.4) | 11 | 0.0 | (1.5) | 2,861 | 1.4 | (1.4) | 852 | 1.3 | (0.7) | 188 | 0.6 | (0.6) | 154 | 0.8 | (1.2) |
| 161 | Myalgia | 10,168 | 2.2 | (2.2) | 3,661 | 3.2 | (3.1) | 66 | 0.3 | (2.2) | 4,513 | 2.3 | (2.2) | 1,241 | 1.9 | (1.5) | 352 | 1.1 | (0.9) | 335 | 1.7 | (2.0) |
| 162 | Other soft tissue disorders, not elsewhere classified | 7,939 | 1.7 | (1.7) | 2,299 | 2.0 | (2.1) | 72 | 0.3 | (1.3) | 3,630 | 1.8 | (1.8) | 1,227 | 1.9 | (1.5) | 514 | 1.6 | (1.4) | 197 | 1.0 | (1.1) |
| 163 | Other soft tissue disorders, not elsewhere classified: pain in limb | 22,201 | 4.9 | (4.9) | 6,978 | 6.2 | (5.9) | 858 | 3.5 | (5.3) | 9,900 | 4.9 | (5.0) | 2,933 | 4.5 | (4.1) | 904 | 2.8 | (2.8) | 628 | 3.1 | (3.4) |
| 164 | Fibromyalgia | 3,399 | 0.7 | (0.7) | 1,271 | 1.1 | (1.2) | 12 | 0.0 | (0.8) | 1,494 | 0.7 | (0.7) | 490 | 0.7 | (0.3) | 65 | 0.2 | (0.1) | 67 | 0.3 | (0.5) |
| 165 | Osteoporosis ^c^ | 158,813 | 34.9 | (34.8) | 68,692 | 60.9 | (36.0) | 20 | 0.1 | (34.8) | 56,592 | 28.3 | (34.7) | 21,704 | 33.0 | (35.0) | 5,055 | 15.7 | (33.2) | 6,750 | 33.4 | (32.3) |
| 166 | Osteoporosis in diseases classified elsewhere | 1,007 | 0.2 | (0.2) | 401 | 0.4 | (0.3) | 8 | 0.0 | (0.2) | 404 | 0.2 | (0.2) | 129 | 0.2 | (0.2) | 42 | 0.1 | (0.2) | 23 | 0.1 | (0.1) |
| 167 | Adult osteomalacia and other disorders of bone density and structure | 43,271 | 9.5 | (9.5) | 14,803 | 13.1 | (9.2) | 114 | 0.5 | (8.9) | 17,949 | 9.0 | (9.8) | 7,555 | 11.5 | (10.4) | 1,932 | 6.0 | (8.8) | 918 | 4.5 | (6.4) |
| 168 | Disorders of continuity of bone | 1,865 | 0.4 | (0.4) | 648 | 0.6 | (0.5) | 87 | 0.4 | (0.3) | 789 | 0.4 | (0.4) | 219 | 0.3 | (0.4) | 70 | 0.2 | (0.3) | 52 | 0.3 | (0.3) |
| 169 | Other osteopathies | 24,251 | 5.3 | (5.3) | 9,059 | 8.0 | (6.6) | 194 | 0.8 | (5.2) | 10,288 | 5.1 | (5.3) | 3,175 | 4.8 | (4.6) | 894 | 2.8 | (3.4) | 641 | 3.2 | (3.7) |
| 170 | Other disorders of the musculoskeletal system and connective tissue | 30,038 | 6.6 | (6.6) | 9,686 | 8.6 | (8.4) | 426 | 1.7 | (5.2) | 13,821 | 6.9 | (6.8) | 3,911 | 6.0 | (5.7) | 1,401 | 4.4 | (3.9) | 793 | 3.9 | (4.1) |
|  | **N – Diseases of the genitourinary system** | **20,162** | **4.4** | **(4.5)** | **8,621** | **7.6** | **(5.9)** | **81** | **0.3** | **(4.5)** | **7,956** | **4.0** | **(4.2)** | **1,770** | **2.7** | **(3.2)** | **713** | **2.2** | **(2.8)** | **1,021** | **5.1** | **(5.1)** |
| 171 | Chronic renal failure (CRF) ^c^ | 20,162 | 4.4 | (4.5) | 8,621 | 7.6 | (5.9) | 81 | 0.3 | (4.5) | 7,956 | 4.0 | (4.2) | 1,770 | 2.7 | (3.2) | 713 | 2.2 | (2.8) | 1,021 | 5.1 | (5.1) |
|  | **Q – Congenital malformations, deformations, and chromosomal abnormalities** | **124,898** | **27.4** | **(27.5)** | **33,012** | **29.3** | **(32.6)** | **4,671** | **19.2** | **(19.1)** | **54,447** | **27.2** | **(27.1)** | **20,474** | **31.2** | **(27.9)** | **9,581** | **29.8** | **(26.4)** | **2,713** | **13.4** | **(11.3)** |
| 172 | Congenital malformations: of the nervous, circulatory and respiratory systems, cleft palate and cleft lip, urinary tract, bones and muscles, other and chromosomal abnormalities not elsewhere classified | 85,534 | 18.8 | (18.9) | 23,361 | 20.7 | (23.3) | 3,460 | 14.2 | (12.5) | 36,389 | 18.2 | (18.3) | 14,026 | 21.3 | (18.7) | 6,333 | 19.7 | (17.6) | 1,965 | 9.7 | (8.3) |
| 173 | Congenital malformations of eye, ear, face and neck | 19,689 | 4.3 | (4.3) | 4,569 | 4.0 | (4.8) | 918 | 3.8 | (3.1) | 8,808 | 4.4 | (4.4) | 3,426 | 5.2 | (4.6) | 1,621 | 5.0 | (4.4) | 347 | 1.7 | (1.0) |
| 174 | Other congenital malformations of the digestive system | 6,481 | 1.4 | (1.4) | 2,257 | 2.0 | (1.8) | 106 | 0.4 | (1.2) | 2,699 | 1.3 | (1.4) | 858 | 1.3 | (1.2) | 367 | 1.1 | (1.2) | 194 | 1.0 | (1.0) |
| 175 | Congenital malformations of the sexual organs | 16,192 | 3.6 | (3.6) | 3,704 | 3.3 | (3.7) | 268 | 1.1 | (2.5) | 7,742 | 3.9 | (3.6) | 2,687 | 4.1 | (4.0) | 1,509 | 4.7 | (3.9) | 282 | 1.4 | (1.3) |
|  | **F – Mental and behavioural disorders** | **683,194** | **150.0** | **(150.7)** | **254,454** | **225.5** | **(222.3)** | **19,635** | **80.6** | **(121.4)** | **269,957** | **134.8** | **(136.6)** | **85,741** | **130.5** | **(118.7)** | **28,936** | **90.1** | **(85.4)** | **24,471** | **121.2** | **(117.6)** |
| 176 | Dementia ^c^ | 36,803 | 8.1 | (8.1) | 18,319 | 16.2 | (8.8) | 0 | 0.0 | (8.5) | 10,766 | 5.4 | (7.4) | 2,811 | 4.3 | (6.8) | 1,066 | 3.3 | (7.5) | 3,840 | 19.0 | (15.3) |
| 177 | Organic, including symptomatic, mental disorders | 26,430 | 5.8 | (5.9) | 12,791 | 11.3 | (9.4) | 113 | 0.5 | (5.5) | 8,481 | 4.2 | (4.5) | 2,219 | 3.4 | (3.7) | 890 | 2.8 | (3.3) | 1,936 | 9.6 | (9.2) |
| 178 | Mental and behavioural disorders due to use of alcohol | 59,143 | 13.0 | (13.2) | 24,641 | 21.8 | (22.5) | 1,323 | 5.4 | (9.3) | 24,285 | 12.1 | (11.1) | 4,884 | 7.4 | (7.8) | 1,701 | 5.3 | (3.9) | 2,309 | 11.4 | (13.4) |
| 179 | Mental and behavioural disorders due to psychoactive substance use | 53,669 | 11.8 | (11.9) | 26,234 | 23.2 | (25.0) | 1,392 | 5.7 | (4.2) | 19,286 | 9.6 | (9.3) | 3,671 | 5.6 | (5.2) | 956 | 3.0 | (1.6) | 2,130 | 10.5 | (9.9) |
| 180 | Schizophrenia ^c^ | 29,422 | 6.5 | (6.5) | 15,920 | 14.1 | (15.3) | 321 | 1.3 | (3.2) | 9,369 | 4.7 | (4.2) | 1,789 | 2.7 | (2.2) | 776 | 2.4 | (0.8) | 1,247 | 6.2 | (6.0) |
| 181 | Schizotypal and delusional disorders | 39,694 | 8.7 | (8.8) | 18,976 | 16.8 | (17.8) | 645 | 2.6 | (5.6) | 13,270 | 6.6 | (6.3) | 3,431 | 5.2 | (4.4) | 1,513 | 4.7 | (3.2) | 1,859 | 9.2 | (9.0) |
| 182 | Bipolar affective disorder ^c^ | 22,669 | 5.0 | (5.0) | 8,109 | 7.2 | (7.0) | 216 | 0.9 | (3.9) | 8,834 | 4.4 | (4.4) | 3,531 | 5.4 | (4.7) | 1,326 | 4.1 | (3.9) | 653 | 3.2 | (3.7) |
| 183 | Depression ^c^ | 454,933 | 99.9 | (100.2) | 161,524 | 143.1 | (136.8) | 6,768 | 27.8 | (77.3) | 189,274 | 94.5 | (96.4) | 63,077 | 96.0 | (83.8) | 19,825 | 61.7 | (59.3) | 14,465 | 71.6 | (72.2) |
| 184 | Mood (affective) disorders | 6,887 | 1.5 | (1.5) | 2,790 | 2.5 | (2.5) | 100 | 0.4 | (1.3) | 2,565 | 1.3 | (1.3) | 940 | 1.4 | (1.2) | 271 | 0.8 | (0.7) | 221 | 1.1 | (1.1) |
| 185 | Phobic anxiety disorders | 14,324 | 3.1 | (3.2) | 5,804 | 5.1 | (6.1) | 431 | 1.8 | (1.0) | 5,457 | 2.7 | (2.7) | 1,768 | 2.7 | (2.0) | 630 | 2.0 | (1.1) | 234 | 1.2 | (0.4) |
| 186 | Other anxiety disorders | 38,079 | 8.4 | (8.4) | 14,581 | 12.9 | (14.2) | 854 | 3.5 | (5.0) | 14,638 | 7.3 | (7.3) | 5,269 | 8.0 | (6.3) | 1,686 | 5.2 | (3.8) | 1,051 | 5.2 | (4.4) |
| 187 | Obsessive compulsive disorder (OCD) ^c^ | 10,062 | 2.2 | (2.2) | 3,294 | 2.9 | (3.5) | 762 | 3.1 | (0.9) | 3,922 | 2.0 | (2.0) | 1,332 | 2.0 | (1.9) | 578 | 1.8 | (1.6) | 174 | 0.9 | (0.1) |
| 188 | Post–traumatic stress disorder | 16,055 | 3.5 | (3.6) | 5,724 | 5.1 | (5.9) | 153 | 0.6 | (2.9) | 6,296 | 3.1 | (2.8) | 1,867 | 2.8 | (2.1) | 592 | 1.8 | (0.7) | 1,423 | 7.0 | (7.3) |
| 189 | Reactions to severe stress and adjustment disorders | 61,701 | 13.5 | (13.7) | 23,214 | 20.6 | (23.5) | 3,171 | 13.0 | (6.0) | 23,952 | 12.0 | (11.9) | 7,364 | 11.2 | (10.1) | 2,086 | 6.5 | (5.2) | 1,914 | 9.5 | (7.9) |
| 190 | Dissociative (conversion) disorders, somatoform disorders and other neurotic disorders | 21,420 | 4.7 | (4.7) | 7,912 | 7.0 | (7.5) | 326 | 1.3 | (4.0) | 8,773 | 4.4 | (4.3) | 2,836 | 4.3 | (3.1) | 978 | 3.0 | (2.2) | 595 | 2.9 | (3.2) |
| 191 | Eating disorders | 7,751 | 1.7 | (1.7) | 2,039 | 1.8 | (2.5) | 946 | 3.9 | (-0.2) | 3,228 | 1.6 | (1.8) | 1,089 | 1.7 | (1.6) | 339 | 1.1 | (1.3) | 110 | 0.5 | (-0.3) |
| 192 | Behavioural syndromes associated with physiological disturbances and physical factors | 6,163 | 1.4 | (1.3) | 1,354 | 1.2 | (1.5) | 100 | 0.4 | (0.4) | 2,620 | 1.3 | (1.3) | 1,267 | 1.9 | (1.8) | 660 | 2.1 | (1.7) | 162 | 0.8 | (0.6) |
| 193 | Emotionally unstable personality disorder | 21,848 | 4.8 | (4.9) | 10,738 | 9.5 | (11.3) | 542 | 2.2 | (1.3) | 7,237 | 3.6 | (3.6) | 2,245 | 3.4 | (1.8) | 587 | 1.8 | (0.3) | 499 | 2.5 | (1.4) |
| 194 | Specific personality disorders | 50,415 | 11.1 | (11.2) | 21,873 | 19.4 | (22.0) | 834 | 3.4 | (6.9) | 18,633 | 9.3 | (8.9) | 5,921 | 9.0 | (6.4) | 1,876 | 5.8 | (2.6) | 1,278 | 6.3 | (5.2) |
| 195 | Disorders of adult personality and behaviour | 17,533 | 3.8 | (3.9) | 7,571 | 6.7 | (7.7) | 292 | 1.2 | (2.6) | 6,510 | 3.3 | (3.0) | 1,939 | 3.0 | (2.1) | 665 | 2.1 | (0.8) | 556 | 2.8 | (2.4) |
| 196 | Mental retardation | 13,822 | 3.0 | (3.1) | 11,095 | 9.8 | (10.6) | 547 | 2.2 | (0.9) | 549 | 0.3 | (0.1) | 81 | 0.1 | (0.0) | 39 | 0.1 | N/A | 1,511 | 7.5 | (7.4) |
| 197 | Disorders of psychological development | 9,911 | 2.2 | (2.2) | 5,937 | 5.3 | (5.9) | 1,700 | 7.0 | (2.0) | 1,400 | 0.7 | (0.8) | 192 | 0.3 | (0.9) | 119 | 0.4 | (0.7) | 563 | 2.8 | (2.1) |
| 198 | Hyperkinetic disorders (ADHD) ^c^ | 42,908 | 9.4 | (9.5) | 19,548 | 17.3 | (19.7) | 6,703 | 27.5 | (12.2) | 11,812 | 5.9 | (6.1) | 2,712 | 4.1 | (5.6) | 1,048 | 3.3 | (3.6) | 1,085 | 5.4 | (2.9) |
| 199 | Behavioural and emotional disorders with onset usually occurring in childhood and adolescence | 39,602 | 8.7 | (8.8) | 17,990 | 15.9 | (17.6) | 2,279 | 9.4 | (5.7) | 13,185 | 6.6 | (6.5) | 3,501 | 5.3 | (5.0) | 1,295 | 4.0 | (3.1) | 1,352 | 6.7 | (5.5) |
|  |  |  |  |  |  |  |  |  |  |  |  |  |  |  |  |  |  |  |  |  |  |  |
|  | **Having one or more chronic conditions** | **2,989,441** | **656.2** | **(657.2)** | **867,345** | **768.5** | **(713.2)** | **67,277** | **276.0** | **(560.2)** | **1,333,904** | **666.1** | **(667.9)** | **445,255** | **677.4** | **(646.5)** | **193,164** | **601.3** | **(604.2)** | **82,496** | **408.4** | **(452.2)** |
|  |  |  |  |  |  |  |  |  |  |  |  |  |  |  |  |  |  |  |  |  |  |  |
|  | **Total population** | **4,555,439** | **1000** | **N/A** | **1,128,588** | **1,000** | **(1000)** | **243,726** | **1,000** | **(1000)** | **2,002,633** | **1,000** | **(1000)** | **657,252** | **1,000** | **(1000)** | **321,254** | **1,000** | **(1000)** | **201,986** | **1,000** | **(1000)** |
|  |  |  |  |  |  |  |  |  |  |  |  |  |  |  |  |  |  |  |  |  |  |  |
|  | Depression medicine ^c^ ** | 529,918 | 116.3 | (116.7) | 191,590 | 70.9 | (160.4) | 8,919 | 36.6 | (94.9) | 218,564 | 109.1 | (111.4) | 69,794 | 106.2 | (93.6) | 22,768 | 169.8 | (70.0) | 18,283 | 90.5 | (92.5) |
|  | Antipsychotic medicine ^c^ ** | 138,625 | 30.4 | (30.6) | 64,514 | 13.2 | (56.7) | 3,176 | 13.0 | (22.8) | 47,132 | 23.5 | (23.4) | 12,914 | 19.6 | (17.9) | 4,225 | 57.2 | (11.8) | 6,664 | 33.0 | (33.1) |
|  | Indication prescribed anxiety medicine ^c^ ** | 102,568 | 22.5 | (22.6) | 37,917 | 13.7 | (33.3) | 1,792 | 7.4 | (16.1) | 41,687 | 20.8 | (21.1) | 13,503 | 20.5 | (17.4) | 4,400 | 33.6 | (12.7) | 3,269 | 16.2 | (16.3) |
|  | Heart failure medication ^c^ ** | 7,468 | 1.6 | (1.7) | 3,127 | 1.0 | (2.0) | 7 | 0.0 | (1.7) | 3,027 | 1.5 | (1.6) | 667 | 1.0 | (1.3) | 311 | 2.8 | (1.2) | 329 | 1.6 | (1.7) |
|  | Ischaemic heart medication ^c^ ** | 129,484 | 28.4 | (28.5) | 57,571 | 13.5 | (35.2) | 36 | 0.1 | (30.0) | 47,700 | 23.8 | (26.4) | 11,785 | 17.9 | (22.5) | 4,324 | 51.0 | (20.9) | 8,068 | 39.9 | (39.4) |
|  | **All of the five types of medicine above** | **688,006** | **151.0** | **(151.6)** | **262,536** | **88.1** | **(92.4)** | **10,846** | **44.5** | **(128.4)** | **275,162** | **137.4** | **(141.4)** | **83,969** | **127.8** | **(118.8)** | **28,310** | **232.6** | **(211.0)** | **27,183** | **134.6** | **(137.0)** |
|  |  |  |  |  |  |  |  |  |  |  |  |  |  |  |  |  |  |  |  |  |  |  |
|  | **Extra** |  |  |  |  |  |  |  |  |  |  |  |  |  |  |  |  |  |  |  |  |  |
|  | Ischaemic Heart Diseases | 315,901 | 69.3 | (69.3) | 120,791 | 107.0 | (78.8) | 824 | 3.4 | (67.9) | 129,207 | 64.5 | 67.8) | 36,025 | 54.8 | (62.5) | 15,151 | 47.2 | (58.6) | 13,903 | 68.8 | (73.0) |
|  | Artritis | 505,792 | 111.0 | (111.0) | 175,514 | 155.5 | (120.9) | 1,528 | 6.3 | (108.4) | 222,089 | 110.9 | 114.2) | 68,505 | 104.2 | (103.2) | 23,321 | 72.6 | (87.3) | 14,835 | 73.4 | (90.7) |
|  | Arthrosis | 338,166 | 74.2 | (74.2) | 125,325 | 111.0 | (81.8) | 237 | 1.0 | (75.0) | 145,512 | 72.7 | 76.2) | 43,695 | 66.5 | (68.1) | 12,528 | 39.0 | (53.3) | 10,869 | 53.8 | (64.9) |
|  | Back conditions | 212,948 | 46.7 | (46.7) | 70,622 | 62.6 | (55.8) | 2,798 | 11.5 | (45.0) | 95,642 | 47.8 | 47.8) | 28,098 | 42.8 | (39.9) | 9,814 | 30.5 | (31.1) | 5,974 | 29.6 | (34.7) |
|  | Overweight | 220,928 | 48.5 | (48.5) | 68,152 | 60.4 | (67.3) | 1,763 | 7.2 | (42.3) | 101,339 | 50.6 | 50.9) | 35,644 | 54.2 | (34.8) | 8,875 | 27.6 | (14.7) | 5,155 | 25.5 | (25.1) |
|  | Endometriosis | 29,290 | 6.4 | (6.4) | 6,205 | 5.5 | (6.7) | 154 | 0.6 | (5.4) | 13,694 | 6.8 | 6.9) | 6,689 | 10.2 | (6.5) | 2,100 | 6.5 | (5.1) | 448 | 2.2 | (3.3) |
|  |  |  |  |  |  |  |  |  |  |  |  |  |  |  |  |  |  |  |  |  |  |  |

Age and gender standardised estimates in brackets.

ICD-10 International Statistical Classification of Diseases, 10^th^ Revision.

^c^ = complex defined conditions, see reference for further details [55].

* Total population frequencies and prevalence adapted from Hvidberg et al. 2019 [56].

** 2-year prevalence.
